# Proteomic Portrait of Degranulation Program in Human Circulating Neutrophils Upon Multi-Inflammatory and Infectious Activation

**DOI:** 10.1016/j.mcpro.2025.101078

**Published:** 2025-09-29

**Authors:** Ying Hua, Ziqi Zhou, Can Zhang, Hai Fang, Mingxing Wu, Zhiyong Chen, Xin Ku, Wei Yan

**Affiliations:** 1 Key Laboratory of Systems Biomedicine (Ministry of Education), Shanghai Center for Systems Biomedicine, State Key Laboratory of Medical Genomics, Shanghai Jiao Tong University, Shanghai, China; 2 Shanghai Institute of Hematology, State Key Laboratory of Medical Genomics, National Research Center for Translational Medicine at Shanghai, Ruijin Hospital, Shanghai Jiao Tong University School of Medicine, Shanghai, China; 3 State Key Laboratory of Ophthalmology, Zhongshan Ophthalmic Center, Sun Yat-sen University, Guangdong Provincial Key Laboratory of Ophthalmology and Vision Science, Guangdong Provincial Clinical Res CN, Guangdong, China; 4 Department of Rheumatology & Immunology, Shanghai Jiao Tong University School of Medicine Affiliated Sixth People’s Hospital, Shanghai, China

**Keywords:** neutrophils, multistate proteomics, degranulation, ectodomain shedding, secretome

## Abstract

Neutrophils respond rapidly to inflammation and infection *via* defense mechanisms, including degranulation, reactive oxygen species production, and neutrophil extracellular trap formation (known as “NETosis”). As the most abundant neutrophil components, granule proteins constitute the major mediators of neutrophil effector functions and likely orchestrate their functional diversity. However, a systematic profile of these proteins, particularly their temporal release dynamics during inflammatory responses, remains uncharacterized. Here, we performed a “multistate” proteomic study to explore circulating neutrophils’ dynamic responses to diverse infectious and inflammatory signals over time. Circulating neutrophils exhibited both conserved and stimulus-specific protein expression programs. Through integrated characterization of the cellular and secretory proteome landscapes, we delineated the release patterns of canonical granule proteins and identified inflammatory mediators, including soluble membrane receptors. Notably, granule membrane receptors were translocated to the cell surface and shed *via* proteolytic cleavage, highlighting their dynamic regulation and diversity. These findings revealed the complexity of the neutrophil degranulation program, demonstrating its stimulus-dependent and temporally layered nature. Our study provides a functional atlas of neutrophil degranulation upon inflammation, which would strengthen our understanding of neutrophil activation in inflammation and facilitate the exploration of inflammation management therapies.

## Highlights

• Neutrophils exhibit distinct proteome features upon different activations.
• A suite of neutrophil-released inflammatory products are identified.
• Neutrophils display stimuli-specific degranulation programs.
• Membrane proteins are proteolytically cleaved by metalloproteinases/a disintegrin and metalloproteinases in inflamed neutrophils.

## Graphical Abstract

**Figure:**
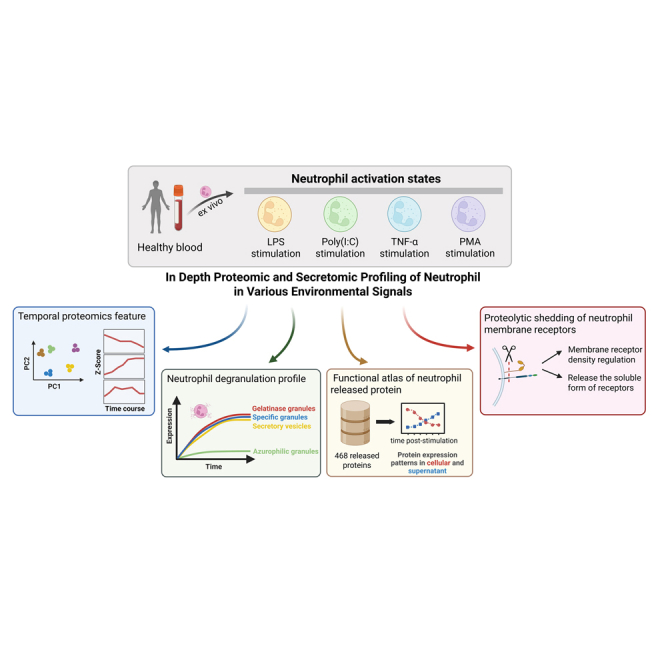

## In Brief

Neutrophils are essential for inflammation and antimicrobial defense. Granule proteins dictate neutrophil effector functions. A deep understanding of their functional release in various contexts is important. In this study, we portrayed the proteomic landscape with a significant focus on the neutrophil secretome across multiple inflammatory and infectious states. Through integrated characterization of the cellular and secretory proteomes, we delineated granule protein release patterns and revealed the complexity of the neutrophil degranulation program, demonstrating its stimulus-dependent and temporally layered nature.

Neutrophils, the most abundant leukocytes in peripheral blood, play a substantial role in immune response to microbial and sterile challenges (1). Upon infection, neutrophils counteract pathogens through diverse mechanisms, including reactive oxygen species release, degranulation, and neutrophil extracellular trap (NET) formation (2, 3, 4). Unlike other circulating leukocyte subtypes (*e.g.*, T cells, B cells, and monocytes), peripheral neutrophils exhibit a relatively short lifespan (less than 1 day) and terminal differentiation properties (5). Therefore, their effector functions need to be tightly orchestrated by granule proteins stored in distinct subsets (6, 7). These subsets, namely azurophilic (primary), specific (secondary), gelatinase (tertiary) granules, and secretory vesicles (SVs), are well defined by stage-specific protein compositions acquired during granulopoiesis (8). Packed with a repertoire of innate immunity proteins such as microbicides, proteases, and membrane proteins, different granule subsets exhibit varying propensities to precisely release these granule proteins in response to various inflammatory and/or infectious signals (6). Previous proteomic analyses on the neutrophil granule subsets isolated by subcellular fractionation *via* density gradient centrifugation have reported identification of numerous proteins. For example, Lominadze *et al*. (9) identified 286 proteins from purified neutrophil azurophil, specific, and gelatinase granules. Rørvig *et al*. (10) analyzed the purified neutrophil azurophil granules, specific granules, gelatinase granules, SVs, and cell membranes and quantified 1292 proteins, of which 126 prominent proteins known as granule proteins or with a high likelihood of being granule proteins were chosen for comparative analysis with the mRNA expression profile. However, how these granule proteins are functionally discharged by neutrophils under different physiological states, for example, in response to numerous immunological and inflammatory stimulating signals, has not been systematically characterized at the proteome level.

There is accumulating evidence indicating a wide spectrum of neutrophil states in both homeostasis and disease, marked by differences in density, surface receptors, granule composition, transcript expression, and associated functional diversity (11, 12, 13, 14). Mature neutrophils appear to be highly heterogeneous and plastic when challenged by different signals from local or systemic origin. For example, circulating neutrophils rapidly acquired tissue-restricted properties upon recruitment into tissue, despite limited residence times (15). Similarly, protumor or antitumor neutrophil phenotypes were developed in different tumor niches (16). In addition, a neutrophil proteomic study was reported to display oscillations following circadian rhythm, especially in granule content (17). These findings underscore that a systematic dissection of the neutrophil proteome requires not only a snapshot investigation under a stand-alone cell state (9, 18) but also a holistic dissection of neutrophils with multiple functional states in a time-dependent manner.

In this study, we employed mass spectrometry (MS)–based quantitative proteomics to profile circulating neutrophils under multi-inflammatory and infectious activation states. As a proof of principle, four classical infectious/inflammatory stimuli were selected to activate neutrophils, with time-course analyses capturing proteome dynamics across activation states. We found that neutrophils exhibited distinct and dynamic protein expression patterns, with granule exocytosis emerging as a key determinant of their activation state. To establish a comprehensive understanding of the function release of neutrophil granule proteins, we also performed time-resolved secretome profiling of the neutrophil under the same multiple stimulation states. By integrating the cellular proteome and secretome data, we depicted an inflammation-mediated exocytosis pattern for each protein functionally released by the inflammatory neutrophil, including those previously identified in the isolated granules (9, 10) and those with the signature pattern that are potentially novel granule proteins. On further examination of the membrane proteins in these functionally released protein datasets, we found that many membrane granule proteins can be proteolytically cleaved after fusion with the plasma membrane and shed soluble compartments into the extracellular space. Such portraits of the degranulation program will provide significant insights on understanding the functional and phenotypic plasticity of neutrophils resulting from granule exocytosis upon different inflammatory activations.

## Experimental Procedures

### Neutrophil Isolation

Neutrophils were isolated from fresh blood of healthy donors with written consent of each donor. This study was approved by the Ethics Committee of Shang Hai Jiao Tong University (B20240362I) and abided by the Declaration of Helsinki principles. Whole blood (collected within 1 h) was layered at a 1:1 volume ratio on the top of 5 ml of Polymorphprep solution (Serumwerk Bernburg) and centrifuged for 40 min at 600*g* without brake. Neutrophils were carefully collected from the second buffy coat layer according to the manufacturer’s instructions and washed in Dulbecco’s PBS (Ca^2+^/Mg^2+^ free) containing 5% homemade human serum. Residual erythrocytes were removed by incubating with red blood cell lysis buffer (Sangon Biotech) for 5 min. The isolated and washed neutrophils were resuspended in an appropriate volume of RPMI1640 medium (Gibco; Thermo Fisher Scientific, Inc).

### Cell Culture and Time-Course Stimulation

Neutrophils (1 × 10^6^ cells/ml) were cultured in RPMI1640, supplemented with 10% human serum, under 5% CO_2_ at 37 °C with a water vapor-saturated atmosphere. After resting for 30 min at 37 °C, neutrophils were incubated in one of the four specific stimulants: (a) 100 ng/ml lipopolysaccharide (LPS) (Sigma–Aldrich); (b) 10 ng/ml phorbol 12-myristate 13-acetate (PMA; InvivoGen); (c) 50 μg/ml Poly(I:C) (InvivoGen); (d) 20 ng/ml tumor necrosis factor alpha (TNF-α) (Beyotime) for 0 min, 15 min, 30 min, 1 h, 2 h, and 4 h. For each time point and each stimulation condition in the time-course experiment, three independent biological replicates were applied. Following incubation under these four conditions, neutrophils were collected, washed twice in PBS, and stored at −80°C until further use.

### Cell Lysate Preparation

Cell pellets were lysed in 0.2% homemade acid-labile surfactant (19) in 50 mM Hepes buffer containing 1× EDTA-free protease inhibitor mixture (Roche). The lysate was centrifuged at 15,000*g* for 30 min at 4 °C. The standard BCA (Thermo Fisher Scientific, Inc) assay was applied to measure protein concentrations of all samples.

After lysis, proteins were denatured with 6 M urea at 37 °C for 30 min, reduced with 5 mM Tris(2-carboxyethyl) phosphine (Sangon Biotech) at 56 °C for 30 min, and alkylated with 25 mM iodoacetamide (Sangon Biotech) for 30 min at room temperature in the dark. Digestion was performed by adding trypsin (Enzyme & Spectrum) in an enzyme-to-substrate ratio of 1:30, followed by incubation for 14 h at 37 °C. Next, samples were acidified with phosphoric acid and desalted using Sep-Pak columns (96-well plate; Thermo Fisher). The peptides were eluted with 0.1% TFA/50% acetonitrile and dried with a vacuum concentrator.

### Conditioned Media Sample Preparation

Neutrophils were seeded in 12-well plates at 1 × 10^6^ cells/ml in a serum-free medium, and equal numbers of neutrophils were seeded for each condition. Cells were then incubated in PBS (unstimulated) and one of the three specific stimulants: (a) 100 ng/ml LPS; (b) 20 ng/ml TNF-α; (c) 10 ng/ml PMA for 0 min, 5 min, 15 min, 30 min, 1 h, and 2 h at 37 °C. The cell cultures were collected, and protease inhibitor (Roche) was added immediately. The collected media were centrifuged at 400*g* for 5 min, followed by centrifugation at 1800*g*, 4 °C for 10 min and at 10,000*g*, and 4 °C for 15 min to pellet cell debris. Then, equal volumes of media from different conditions were prepared; protein A (GenScript) was added in equal amounts to each sample as an external standard in order to eliminate the possible technical variation. Next, these media samples were used for downstream peptide preparation and LC–MS/MS analysis. The peptide preparation was performed as described previously.

### MS Data Acquisition

Samples were analyzed using an Ultimate 3000 HPLC system (Thermo Fisher Scientific) coupled to a Thermo Orbitrap Fusion in data-independent acquisition (DIA) mode. For proteome analysis of the cell lysate, 1 μg peptides were loaded. For conditioned media samples, peptides prepared from the same equivalent volume (1/3 of the total volume of the cultured medium) of conditioned media were loaded. Tryptic peptides were loaded onto a 15 cm column with a 75 μM inner diameter, packed in-house with 1.9 μM C-18 ReproSil particles (Dr Maisch GmbH), and separated using a binary buffer system of 0.1% formic acid (CNW) in water (buffer A) and 0.1% formic acid in 100% acetonitrile (buffer B), at a flow rate of 300 nl/min. The following gradient was applied: 80 min from 5% B to 31% B, 20 min from 31% B to 44% B, 1 min from 44% B to 95% B, 10 min hold at 95% B, 1 min from 95% B to 5% B, and an equilibration at 5% B for 5 min.

For the MS1 scan, the Orbitrap resolution was set to 60,000. A scan range of 350 to 1550 *m/z* was applied, and the automatic gain control target was set to 3 × 10^5^ with a maximum injection time of 100 ms. For the MS2 scan, the Orbitrap resolution was set to 30,000, with 30 fixed windows (21 *m/z* isolation window and 1 *m/z* overlap). MS/MS scans were activated by higher-energy collisional dissociation at 30% normalized collision energy. MS/MS scan range was defined as 400 to 1000 *m/z*. The automatic gain control target was set to 3 × 10^4^ with a maximum injection time of 22 ms. All data were acquired with Xcalibur software (Thermo Fisher Scientific).

### MS Data Process

After MS acquisition, raw files were processed with the DIA-NN (1.8.1) (20) in library-free mode. The predicted library was generated using the SwissProt sequence database for *Homo sapiens*, which was downloaded from UniProt (www.uniprot.org) in October 2022 with 20,423 canonical sequence entries.

For the cell proteome data analyses in DIA-NN, we set the peptide length range to 7 to 30 and the precursor charge range to 1 to 4. The precursor *m/z* range was set to 300 to 1800, and the fragment ion *m/z* range was set to 200 to 1800. The “Robust LC (high accuracy)” was selected for precursor quantification. Trypsin/P was selected as the protease, allowing cleavage at the N-terminal region of proline and between aspartic acid and proline. Maximum missed cleavages were set to one, carbamidomethylated cysteine as fixed modifications, and oxidation of methionine as variable modifications. The precursor false discovery rate was set to 1%. The match-between-run function was enabled, and the cross-run normalization was set as retention time (RT) dependent. Automatic mass and RT correction were performed.

For the secretome data analyses in DIA-NN, the cross-run normalization was disabled. Other search parameters were set as the same as that in the cell proteome data analysis.

### Experimental Design and Statistical Rationale

#### Mass Spectrometry Analysis

The neutrophil proteome data were performed with three biological replicates for each experimental condition. To minimize batch effects, the sample acquisition order was randomized using a block randomization approach based on experimental groups (*e.g.*, control *versus* treatment). Protein A was spiked into neutrophil supernatant sample preparation (0.1 μg protein A in each equal-volume supernatant sample). No RT standards or protein/peptide standards were spiked in the preparation of neutrophil cell lysate or peptide samples. MS1 spectra were acquired with (resolution at “60,000 at *m/z* 200”) over (*m/z* range of “350–1500”) prior to fragmentation. And for the MS/MS acquisition, 30 fixed windows (21 *m/z* isolation window and 1 *m/z* overlap) were applied. Total cycle time was set as 3 s. A total of 72 cell proteome data were generated and analyzed. Raw files were processed with the DIA-NN (1.8.1) (20) in library-free mode. The predicted library was generated using the SwissProt sequence database for *H. sapiens,* which was downloaded from UniProt (www.uniprot.org) in October 2022. MS-DAP (The Mass Spectrometry Downstream Analysis Pipeline) package was used to perform data filtering, normalization, and differential testing on the DIA-NN output report file (report.tsv file) (21). For DIA data analysis, recommended settings by Koopmans *et al.* (21) were used. “Protein.Group” column in the DIA-NN report was used to identify the protein group, and PG.MaxLFQ label-free quantification (LFQ) was used to obtain the normalized value. Proteins quantified with at least one unique peptide are included. The normalization algorithm was set as “vsn,” and “deqms” algorithm was used to identify sets of differentially expressed proteins at each condition or time point. Proteins were considered significantly upregulated or downregulated based on the criteria that the Benjamin–Hochberg adjusted *p* ≤ 0.01 and absolute fold change (FC) ≥1.5. For the differential detection, proteins with at least two peptides are included.

For each stimulation condition, we prepared and analyzed a single neutrophil secretome sample per time point in the time-course experiment, which generated six secretome files for each stimulation condition and 24 secretome files in total. Given we set multiple continuous time points and we mainly accessed the trend of expression levels over time, we prepared only one sample per time point. Statistical analysis was performed in R (version 4.3.1). The DIA-NN output was filtered at a *q* value <1% for precursors and proteins. The quantification data generated from DIA-NN were first normalized using the median normalization and then normalized based on the relative abundance of the external standard protein A in different samples.

### Bioinformatic Analysis

To capture the coregulated protein modules and temporal patterns of protein expression, the time-course cell proteome data were analyzed using the fuzzy c-means algorithm (MFUZZ), which was integrated into the R ClusterGvis package (https://github.com/junjunlab/ClusterGVis). The optimal number of clusters was automatically inferred. Briefly, to identify the most representative clusters, the differentially expressed proteins from MS-DAP analysis are subjected to the MFUZZ analysis.

Gene Ontology enrichment analysis was performed using the web-based tool, Metascape (https://metascape.org/) (22). The total identified proteins of the neutrophil proteomics in this study were set as the background genes. Thresholds were set at Benjamin–Hochberg adjusted *p* < 0.05, minimum count of 3, and enrichment factor >1.5.

### Integration of Time-Dependent Cellular and Supernatant Expression Profiles of Released Granule Proteins

For time-dependent secretome data obtained from conditioned media, FCs at each time point were calculated by comparing protein LFQ values between treatment and control conditions. A linear curve was then fitted to model the time-dependent FCs. Proteins meeting the following criteria were recognized as released proteins with increased expression patterns: (1) the slope of the fitted curve >0 and (2) FC >1.5 in at least one time point.

For time-dependent cell proteome data, the relative percentage of each protein was calculated by comparing LFQ values between stimulated and control neutrophils at baseline (0 min).

For visualization, we plotted line graphs showing: (1) cellular expression levels (“Relative percentage”) and (2) supernatant expression levels (“FC”) for both known granule proteins and all identified released proteins.

### Metalloproteinases/A Disintegrin and Metalloproteinase–Mediated Shedding Identification

GM6001 (Bide Pharm) was used to inhibit metalloproteinase (MMP)/a disintegrin and metalloproteinase (ADAM)-mediated shedding events. Neutrophils were pretreated with 2 μM GM6001 for 30 min at 37 °C and subsequently stimulated with one of the three specific stimulants: (a) 10 ng/ml PMA; (b) 100 ng/ml LPS; and (c) 20 ng/ml TNF-α for 1 h at 37 °C. After stimulation, cell culture media were collected and centrifuged to remove the cell debris. Each media sample was prepared with three biological replicates. The media samples were subsequently subjected to peptide preparation and LC–MS/MS analysis. Peptide sample preparation and MS data analysis procedures were same as for the neutrophil secretome.

After quantification by MS, the soluble levels of membrane proteins in the supernatants from TNF-α/LPS/PMA-treated condition and the TNF-α/LPS/PMA + GM6001-treated conditions were compared using a two-tailed *t* test. Membrane proteins exhibiting statistically significant differences (*p* < 0.05) and reduced production upon GM6001 (|log2 FC| > 0.6) were identified as candidate protein substrates for MMP/ADAMs.

### Bioinformatics Prediction of Secreted Pathways

The potential secretion pathways of nongranular-released proteins were predicted using the SignalP 6.0 server (https://services.healthtech.dtu.dk/services/SignalP-6.0/) and the SecretomeP 2.0 server (https://services.healthtech.dtu.dk/services/SecretomeP-2.0/) to predict the classical or nonclassical secretion pathways. Protein sequences were retrieved from the UniProt database and uploaded onto the SignalP 6.0 and SecretomeP 2.0 servers for the prediction. Potential exosomal and microvesicle release of the proteins was assessed by manual annotation on the Vesiclepedia database. Transmembrane domains were predicted using the TMHMM database.

### Flow Cytometry

Neutrophils were stimulated with LPS (100 ng/ml), PMA (10 ng/ml), Poly(I:C) (50 μg/ml), TNF-α (20 ng/ml), or PBS for 1 h prior to flow cytometry analysis. Cytometric analyses were performed using an LSR Fortessa Analyzer (BD Biosciences). Cells were stained with conjugated antibodies (anti-CD45-FITC [#304006, HI30, BioLegend], anti-CD66b-APC [#305118, G10F5, BioLegend], anti-CD16-PE [#360703, B73.1, BioLegend], anti-CD11b-PerCP/Cy5.5 [#301327, IRCF44, BioLegend], anti-OLR1-PE [#358603, 15C4, BioLegend]) for 30 min at 4 °C in the dark. Cells were gated by dot-plot analysis, and 10,000 cells were acquired per sample. Analysis was conducted using FlowJo (BD Biosciences), version 0.8.1.

### Immunofluorescent Staining

Isolated neutrophils were fixed with 4% (w/v) paraformaldehyde (Sangon Biotech) for 10 min and coated onto slides, permeabilized with 0.1% (w/v) Triton X-100 (Sangon Biotech) solution diluted in PBS for 10 min, and then incubated in blocking solution (PBS with 0.25% Triton X-100 and 1% bovine serum albumin [Share Bio]) for 1 h at room temperature. Primary antibodies (mouse anti-LTF [#44072; Signalway antibody], mouse anti-MMP9 [#49188; Signalway antibody], rabbit anti–quiescin sulfhydryl oxidase 1 (QSOX1) [#12713-1-AP; Proteintech], rabbit anti–tissue inhibitor of metalloproteinase 2 [TIMP-2] [#AF8166; Beyotime]) were diluted in blocking solution (1:200 dilution), added to the slides, and incubated overnight at 4 °C. The next day, the slides were washed three times with PBS and incubated with fluorescently labeled secondary antibodies (Alexa 488-conjugated goat anti-rabbit IgG [1:500 dilution; #A0428; Beyotime], Alexa 647-conjugated goat anti-mouse IgG [1:500 dilution; #A0468; Beyotime]) for 1 h at room temperature in the dark, followed by incubation with the nuclear stain 4,’6-diamidino-2-phenylindole (Sangon Biotech) for 10 min at room temperature. The slides were then observed using a confocal laser microscope (Nikon A1Si), and composite images were created using ImageJ software (National Institutes of Health).

## Result

### Comprehensive Proteomic Analysis Reveals Distinct Features of Human Circulating Neutrophils Across Multiple Inflammatory States

The known features of the relatively short lifespan, terminal differentiation, and limited transcriptional regulation in circulating neutrophils suggest that protein components play extraordinarily important roles for neutrophil function. As neutrophils often show multifaceted adaptabilities in complex immune environments, we are interested in dissecting the proteome of human neutrophils in different activation states. In this regard, neutrophils were isolated from healthy donor blood using standard density gradient separation (Supplemental Fig. S1*A*), with a purity of CD45+CD66b+ populations of more than 95% as analyzed by flow cytometry (Supplemental Fig. S1*B*). Further nuclear morphology evaluation revealed two to five segments, indicating a mature stage of these neutrophils (Supplemental Fig. S1*C*). As a proof-of-principle study, we selected four states of neutrophils stimulated by bacterial LPS, double-stranded RNA analog Poly(I:C), TNF-α, and PKC activator PMA *ex vivo*, respectively, mimicking multiple inflammatory or infectious states induced by bacteria, viruses, proinflammatory cytokines, and potent NETosis activators.

**Fig. 1:**
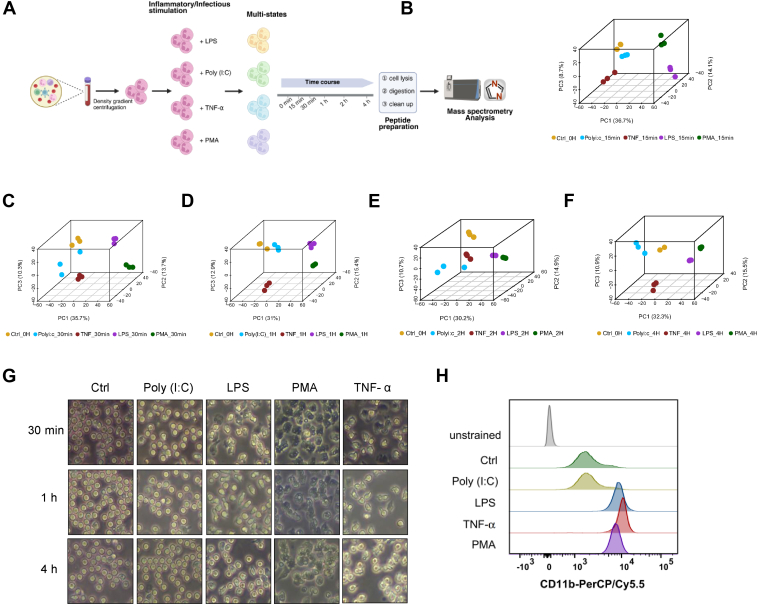
**Schematic workflow of neutrophil activation and proteomic analysis.***A*, neutrophils were freshly isolated from human peripheral blood using density gradient centrifugation and subsequently treated with multiple stimuli (LPS, Poly(I:C), TNF-α, and PMA) in a time-dependent manner. Activated neutrophils were subsequently collected and analyzed by LC–MS/MS. *B*–*F*, three-dimensional PCA of time-resolved neutrophil proteomes under different stimulation. (*B*) 15 min, (*C*) 30 min, (*D*) 1 h, (*E*) 2 h, and (*F*) 4 h. *G*, morphology of activated neutrophils at 30 min, 1 h, and 4 h. Poly(I:C)-treated neutrophils exhibited a round shape similar to that of untreated neutrophils. In contrast, stimulation with LPS, TNF-α, and PMA resulted in an altered, deformed morphology of the neutrophils. *H*, neutrophils were stimulated with LPS, PMA, Poly(I:C), TNF-α or PBS for 1 h and then subjected to evaluation of the cell surface level of CD11b expression by flow cytometry analysis. A histogram plot showing the representative expression levels of the activation marker CD11b in control and stimulus-treated neutrophils. LPS, lipopolyssacharide; PMA, phorbol 12-myristate 13-acetate; TNF-α, tumor necrosis factor alpha.

To depict a longitudinal draft map of the neutrophil proteome across different states, time-course experiments were conducted. Freshly isolated neutrophils were treated with LPS, PMA, Poly(I:C), or TNF-α for 0 min, 15 min, 30 min, 1 h, 2 h, and 4 h, respectively (Fig. 1*A*). Time-course assays were only executed up to 4 h based on our preliminary observation that the neutrophils underwent significant cell death when cultured *ex vivo* for 8 h after the PMA treatment. Even without any treatment, neutrophils cultured for 20 h exhibited a significant downregulation of CD66b (a neutrophil marker) expression compared with freshly isolated neutrophils. Consequently, a total of 24 states were collected, processed, and analyzed by high-resolution MS using a quadrupole Orbitrap instrument. Three biological replicates were collected for each proteome state, generating a total of 72 proteome files. Raw LC–MS/MS data were processed using the DIA-NN software platform (20). With a peptide and protein false discovery rate set at 1%, over 3800 proteins were quantified (Supplemental Table S1).

Three-dimensional principal component analysis was conducted on the acquired neutrophil proteome data (based on LFQ values) for each of the four states at each time point (Fig. 1, *B*–*F*). As shown in Figure 1, *B*–*F*, the three biological replicates of neutrophils within each state clustered together closely, whereas neutrophils from different states deviated markedly from each other, indicating that the different treatments induced dimensionally distinct remodeling of the neutrophil proteome. Significant alterations in the neutrophil proteome were observed as early as 15 min poststimulation with TNF-α, LPS, and PMA, indicating the neutrophils were sensitive and responded rapidly to bacterial infection, proinflammatory cytokine, and PKC activator. At 15 min, PCA separation (PC1/PC2/PC3) was driven by acute stress proteins (*e.g.*, LAMTOR4, MAPKAPK5, EIF1, PSMB5) and inflammatory proteins (*e.g.*, LAMTOR4, ALOX15, C1R), whereas later time responses (2–4 h) shifted toward neutrophil degranulation (*e.g.*, DEFA, PRG3), antibacterial effectors (*e.g.*, CXCL8, IL1B), and reactive oxygen species production (*e.g.*, COX4I1, NDUFB4, VDAC1), reflecting neutrophil reprogramming from rapid sensing to pathogen eradication. Overall, the neutrophil proteome exhibited a great and distinguished change under TNF-α, LPS, PMA, and Poly(I:C) stimulation through time course.

This observation was consistent with the morphology phenotypes of these neutrophils in each stimulated state. Neutrophils treated with LPS, TNF-α, or PMA showed a significant deformed shape along with an increased tendency for adhesion, as demonstrated by their morphology in 30 min, 1 h, and 4 h of treatment (Fig. 1*G*). While Poly(I:C) elicited very minor morphological changes on neutrophils, which maintained round and phase-lucent throughout the 4 h of treatment. We further investigated the neutrophil activation phenotypes by examining the surface levels of a classical neutrophil activation marker, CD11b. As shown in Figure 1*H*, after 1 h of stimulation, the LPS-, TNF-α-, and PMA-treated neutrophils exhibited increased cell surface levels of CD11b, as analyzed by flow cytometry, suggesting canonical activations in these neutrophils. No increase in the surface expression of CD11b was detected in the Poly(I:C)-treated neutrophils as compared with the untreated control, indicating that neutrophils were not significantly activated under the Poly(I:C) treatment in our experiment. These data highlighted that the circulating neutrophils have distinguished proteome profiles and different phenotypes in response to different inflammatory stimuli.

### Temporal Analysis of Expression Patterns Identifies Neutrophil Signature Protein Clusters Rich in Biological Information

**Fig. 2:**
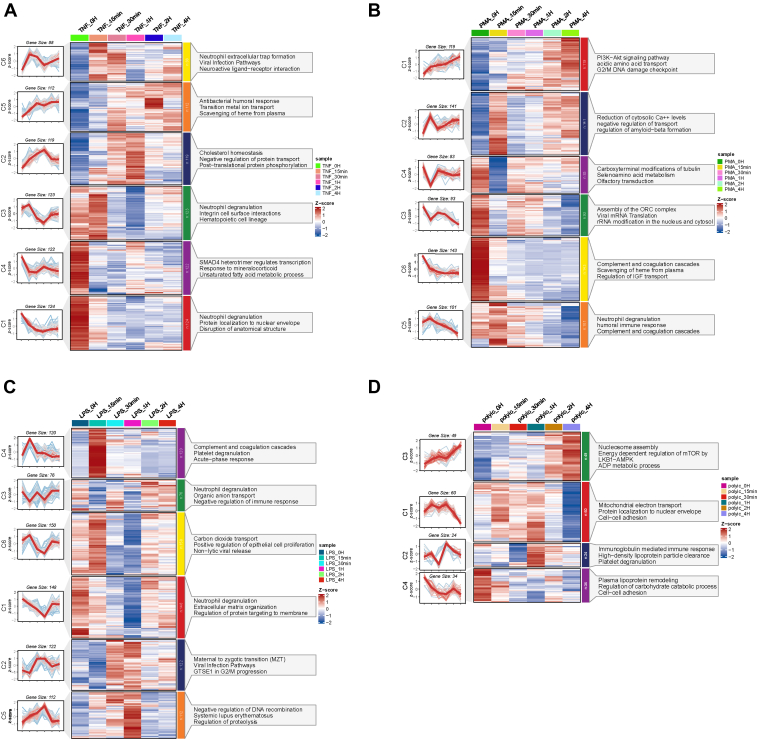
**Temporal proteomic clustering and functional enrichment under inflammatory stimulation.** Heatmap of z-scored temporal expression profiles for each protein in the representative clusters identified by the fuzzy c-means clustering algorithm (MFUZZ). Line plot on the *left* showing the temporal patterns for individual clusters with cluster size labeled. The top three enriched terms from Gene Ontology Biological Pathways (GO-BP) annotation enrichment for each cluster are shown on the *right*. (adjusted *p* value <0.01). *A*, TNF-α condition, (*B*) PMA condition, (*C*) LPS condition, and (*D*) Poly(I:C) condition. LPS, lipopolyssacharide; PMA, phorbol 12-myristate 13-acetate; TNF-α, tumor necrosis factor alpha.

To gain orchestrated insights into neutrophil responses to different inflammatory stimuli over time, we performed a time-course proteomic analysis on cells in each state. A temporal expression pattern was obtained for each identified protein during the 4 h exposure to the inflammatory stimuli. We employed the MFUZZ algorithm (23), a soft clustering approach based on fuzzy c-means clustering principles, to capture coregulated protein modules with similar temporal expression profiles; the optimal cluster numbers were automatically inferred. Proteins within each cluster were annotated in terms of biological pathways, molecular functions, and underlying mechanisms. Using this approach, the most representative protein clusters with their annotation Gene Ontology terms among different conditions were generated (Fig. 2 and Supplemental Table S2). Six clusters representing the regulatory patterns across the timeline of the TNF-α, PMA, and LPS treatments and four clusters in the Poly(I:C)-treated neutrophils were generated and presented. Of these clusters, temporal cluster 2 and cluster 5 in the TNF-α treatment (Fig. 2*A*), cluster 1 in the PMA treatment (Fig. 2*B*), cluster 2 and cluster 5 in the LPS treatment (Fig. 2*C*), and cluster 3 in the Poly(I:C) treatment (Fig. 2*D*) all demonstrated an upregulated trend throughout the time course. Specifically, in the TNF-α stimulation, clusters featuring an upregulated trend (C2, C5 in Fig. 2*A*) were enriched for terms such as “cholesterol hemostasis” and “antibacterial humoral response.” In the PMA stimulation, clusters with an upregulated pattern (C1 in Fig. 2*B*) were functionally associated with the “PI3K-Akt signaling pathway.” In the LPS stimulation, clusters with upregulated trends (C2, C5 in Fig. 2*C*) were enriched for the terms “negative regulation of DNA recombination” and “infection pathways.” The term “nucleosome assembly” was illustrated in cluster 3 of the Poly(I:C) stimulation. All these observations reflected that different biological functions are initiated in neutrophil response to different stimulation.

Several clusters featured downregulated trends throughout the time course, including clusters 1, 3, and 4 in the TNF-α treatment (Fig. 2*A*); clusters 3, 5, and 6 in the PMA treatment (Fig. 2*B*); clusters 1 and 6 in the LPS treatment (Fig. 2*C*); and cluster 3 in the Poly(I:C) treatment (Fig. 2*D*). Among these downregulated clusters in the TNF-α, LPS, and PMA stimulations, multiple of them (*i.e.*, C1 and C3 in Fig. 2*A*; C5 in Fig. 2*B*; and C1 in Fig. 2*C*) were functionally annotated as “neutrophil degranulation,” suggesting that the degranulation program as an essential response can be widely induced during the inflammatory stimulations. However, their detailed temporal patterns throughout the time course in each stimulation state were not identical, suggesting a complex protein discharge process of neutrophil granules. In the Poly(I:C) stimulation, the clusters featuring downregulated trends (C1, C4 in Fig. 2*D*) were enriched for the terms “mitochondrial electron transport” and “plasma lipoprotein remodeling.”

In addition to several overall continuous upregulation or downregulation trends, many proteins also exhibited oscillated expressions over time, such as cluster 6 in the TNF-α stimulation (Fig. 2*A*), with enriched annotation for “neutrophil extracellular trap formation.” Cluster 2 and cluster 4 in the PMA stimulation (Fig. 2*B*) were annotated as “reduction of cytosolic Ca++ levels” and “carboxyterminal modifications of tubulin.” In the LPS stimulation, clusters featuring oscillated patterns (C3 and C4 in Fig. 2*C*) were enriched for “neutrophil degranulation” and “complement and coagulation cascades.” In the Poly(I:C) stimulation (Fig. 2*D*), cluster 2 displayed an oscillated pattern and was functionally annotated as “immunoglobulin-mediated immune response.”

All these data suggested a complicated and dynamic process of protein regulation underlying neutrophil responses to various stimuli. A holistic and longitudinal characterization of the perturbed proteome could provide rich information for dissecting well-tuned neutrophil signaling and plasticity over a stand-alone snapshot analysis. Systematic analysis of the similarities and differences with temporal information of neutrophil protein expression patterns in various inflammatory contexts broadly expanded our understanding of the neutrophil activation process and provided rich data resources for further investigation into neutrophil activity and function.

### Circulating Neutrophils Have Conserved Core Regulatory Mechanisms in Response to Major Inflammatory Stimulation

Among these enriched biological pathways of different neutrophil activation conditions illustrated in Figure 2, we noticed that several of the top enriched biological pathways were annotated to the same functional categories, such as “neutrophil degranulation” and “infection pathways,” which were shared under the TNF-α, LPS, and PMA treatments but not the Poly(I:C) treatment. These mutual signaling events thus proposed that a common core regulatory program might exist in the neutrophil upon inflammation conditions induced by TNF-α, LPS, and PMA.

**Fig. 3:**
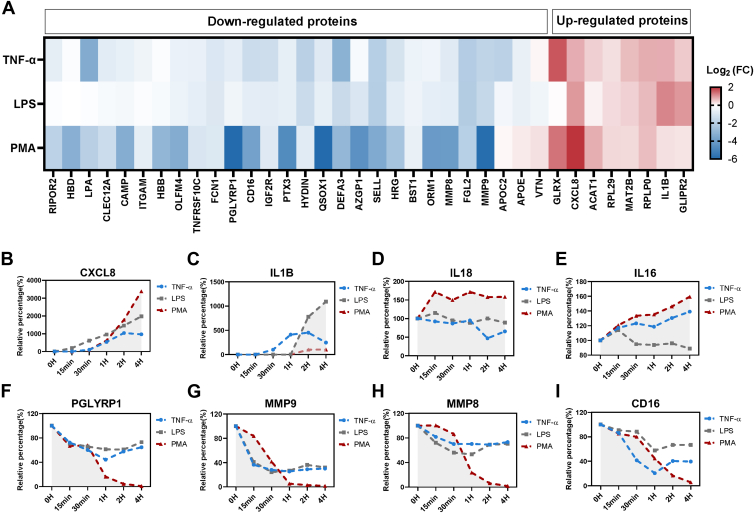
**Neutrophils display conserved protein expression features under inflammatory stress.***A*, heatmap showed 36 proteins with consistent patterns across all three experimental conditions. Shown are the log2 fold changes across comparisons of proteins upregulated and downregulated in the activation of 4 h time point. Rows represent conditions, and columns represent significant proteins (adjusted *p* value <0.05). *B* and *C*, line plot showing the increased pattern of proinflammatory mediator, (*B*) CXCL8, (*C*) IL1B, (*D*) IL-18, and (*E*) IL-16. *F*–*I*, line plot showing the decreased pattern of neutrophil granule proteins, (*F*) PGLYRP1, (*G*) MMP9, (*H*) MMP8, and (*I*) CD16. *Y-*axis: relative percentage (%), the relative percentage of granule proteins in the cellular compartment was calculated by comparing the protein MS signals (label-free quantification [LFQ] intensity) between the stimulated neutrophil and the control neutrophil at baseline (0-min time point). MMP, metalloproteinase; MS, mass spectrometry.

Next, we specifically focused on proteins with similar expression patterns shared across the TNF-α, PMA, and LPS treatments, which could provide useful information on the potential conserved core program. A total of 36 proteins were screened out with consistent expression patterns across all three experimental conditions (Fig. 3*A*). For illustration of the cellular expression for each protein, the relative percentage of its cellular expression during the time course was calculated by dividing the protein MS signal (LFQ intensity) at each time point by its LFQ intensity at 0 min. Figure 3, *B–F* showed the plots of the relative percentages *versus* time points for several representative proteins. As shown in Figure 3, *B* and *C*, there are several proteins that showed conserved upregulated patterns, including the classical proinflammatory mediators CXCL8 and IL1B. CXCL8 and IL1B appeared to be at very low constitutive levels in the untreated neutrophils. Upon stimulation with TNF-α, PMA, or LPS, both cytokines exhibited steadily increasing expression over time (Fig. 3, *B* and *C*), suggesting that mature neutrophils can be boosted to generate needed cytokines upon stimulation. Consistently, neutrophils were previously reported to promote mRNA expression along with synthesis of numerous cytokines in response to inflammatory activating signals (24, 25, 26). Furthermore, we observed that neutrophils could induce the cytokines at different levels depending on the applied stimuli. For example, PMA-activated neutrophils could produce significantly higher levels of CXCL8 than the LPS- and TNF-α-treated neutrophils (Fig. 3*B*). In addition, IL1B was significantly induced under the treatment of LPS but not in the PMA treatment (Fig. 3*C*). These results denoted that a fine-tuning mechanism might exist in neutrophils in response to different inflammatory stimuli. Unlike the CXCL8 and IL1B that were almost not expressed at the constitutive level but could be rapidly induced in response to the inflammatory signals, other cytokines, such as IL-18 and IL-16, were expressed constitutively in circulating neutrophils. This is consistent with previous reports that the resting neutrophils could store IL-18 and IL-16 in the cytosol (27, 28). However, in our proteomic analysis, IL-18 expression was barely changed upon stimulation by the LPS and TNF-α signals, whereas only a mild induced pattern was indeed observed under the PMA treatment condition (Fig. 3*D*). IL-16 displayed the typical upregulation pattern under the TNF-α and PMA treatments but not under the LPS treatment (Fig. 3*E*). Therefore, these two cytokines were not considered as part of the conserved core inflammatory protein network.

Interestingly, most of these shared differentially regulated proteins exhibited downregulated trends in the activated neutrophils (Fig. 3*A*). Many of these proteins are well-known canonical neutrophil granule proteins, including PGLYRP1, MMP9, MMP8, and CD16 (Fig. 3, *F–I*). Following activation with TNF-α, LPS, and PMA, the cellular levels of these neutrophil granule proteins were significantly reduced as quickly as at 15 min of the treatments, suggesting the degranulation process is initiated very rapidly in the activated neutrophils. Neutrophil degranulation is a key effector mechanism by which neutrophils fight against various invading pathogens (1). Such inflammation-mediated reduction of the granule proteins was a prominent feature under TNF-α, LPS, and PMA conditions.

Collectively, these data indicated that the circulating neutrophils have conserved important responsive mechanisms involving the induction of proinflammatory cytokines and reduction of intracellular granule proteins to cope with different inflammatory signals. These substantial changes in the neutrophil proteome under different states provided valuable information on neutrophils’ diverse cellular functional outputs in various microenvironments.

### Integrated Secretome and Cellular Proteome Delineate the Neutrophil Degranulation Process Upon Inflammation

The dramatic decline at the cellular levels of the canonical granule proteins in the inflammatory neutrophils prompted us to dissect whether these granule proteins are indeed released under different activation states.

**Fig. 4:**
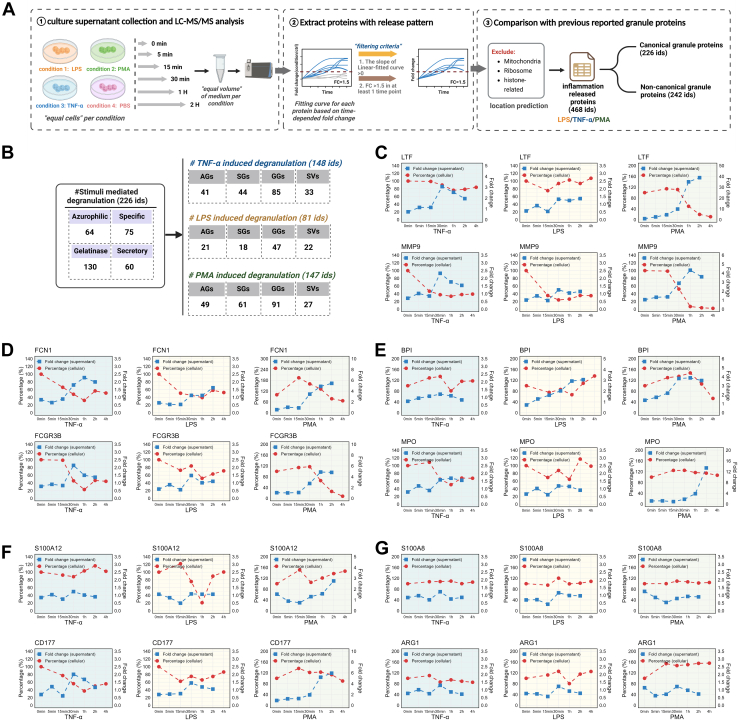
**Quantitative proteomic analysis of conditioned media identifies neutrophil-derived protein release.***A*, schematic description of neutrophil secretome to evaluate granule protein release by neutrophils. Equal numbers of neutrophils were cultured in a serum-free system with different treatments. The supernatants were collected for LC–MS/MS analysis. For each protein, the fold changes (FCs) were calculated by comparing the protein LFQ values between the stimulated and the control conditions at each time point. Time-dependent FCs were fitted to curves, with proteins meeting the following criteria identified as released proteins: (1) the slope of the fitted curve >0 and (2) FC >1.5 in at least one time point. Next, proteins annotated as mitochondrial, ribosomal, or histone related were excluded. *B*, the number of released granule proteins in TNF-α, LPS, and PMA conditions. *C*, a line graph showing the temporal secretion profiles of granule proteins LTF and MMP9 following stimulation with TNF-α (*blue background*), LPS (*yellow background*), and PMA (*green background*). Left *Y*-axis: the relative percentage of protein in the cellular proteome. Right *Y*-axis: the FC of proteins in the supernatant. *X*-axis: time poststimulation. *D*, a line graph showing the cellular and supernatant expression patterns of granular proteins FCN1 and FCGR3B. *E*, the cellular and supernatant expression patterns of azurophilic granule proteins BPI and MPO. *F*, the cellular and supernatant expression patterns of granular proteins S100A12 and CD177. *G*, the cellular and supernatant expression patterns of granular proteins S100A8 and ARG1. AG, azurophilic granule; GG, gelatinase granule; LFQ, label-free quantitation; LPS, lipopolyssacharide; PMA, phorbol 12-myristate 13-acetate; SG, specific granule; SV, secretory vesicle; TNF-α, tumor necrosis factor alpha.

We then performed the proteomic analysis on conditioned medium derived from a serum-free culture system to quantify the extent of protein release from the stimulated neutrophils. Considering the Poly(I:C)-treated neutrophils did not reveal the typical cellular down-regulatory patterns of granule proteins (Supplemental Fig. S1*D*), we only conducted the LPS, TNF-α, and PMA treatments to examine the protein release from such stimulated neutrophils. Equal numbers of the neutrophils were seeded at each condition, and equal volumes of the conditioned media were collected and analyzed at 0 min, 5 min, 15 min, 30 min, 1 h, and 2 h after treatment with LPS, PMA, TNF-α, or PBS (control), respectively, as this culture period enabled the detection of secreted proteins with managed cell death (Fig. 4*A*). Over 1100 proteins were quantified at nearly all time points in the conditioned media (Supplemental Table S3). To specifically characterize the stimulation-mediated protein release, we calculated FCs at each time point by comparing the protein MS signals (LFQ intensity) between the stimulated and the control conditions and fitted the linear curve for each protein based on their time-dependent FCs. Proteins meeting the following criteria, (1) the slope of the fitted linear curve >0 and (2) FC >1.5 in at least one time point, were recognized as “released” proteins in the neutrophil supernatant. Next, proteins annotated as mitochondrial, ribosomal, or histone related were excluded. Filtered proteins from LPS, TNF-α, and PMA conditions were subsequently combined, resulting in a list of 468 neutrophil-released proteins (Supplemental Table S4). Then we compared this “released” protein list with previous neutrophil granule proteomics studies and biochemical studies (9, 10, 29) (Fig. 4*A*). Of these 468 released proteins, 226 proteins were previously reported granule proteins, and 242 proteins were unclassified released proteins (non-canonical granule proteins).

Among these 226 stimuli-inducible released granule proteins, 64 of them were in the azurophilic granules, 75 proteins were in the specific granules, 130 proteins were in the gelatinase granules, and 60 proteins were in the SVs according to the previously published neutrophil granule proteomic studies (9, 10) (Fig. 4*B*). Many granule proteins were assigned into more than one granule subset based on the published data. In summary, 148, 81, and 147 granule proteins showed their release in the TNF-α, LPS, and PMA treatments, respectively (a detailed protein list is shown in Supplemental Fig. S2).

Since we also have the cellular expression data for each of these granule proteins, we then set out to plot and compare the cellular and supernatant expression through the time course for each released granule protein under each inflammatory condition. Temporal dynamics of several representative canonical granule proteins are illustrated in Figure 4, *C–G*, showing cellular and supernatant expression profiles under the TNF-α (*blue background*), LPS (*yellow background*), and PMA (*green background*) stimulations. Several granule proteins exhibited an “increase” of the pattern in the supernatant (blue line) and a concordant “decrease” of the pattern in the cellular (red line) among all three conditions, such as LTF, MMP9, FCN1, FCGR3B, QPCT, and OLFM4 (Fig. 4, *C* and *D* and Supplemental Fig. S3*A*). Notably, a rapid secretion phase was observed between 15 and 30 min poststimulation for all conditions among many granule proteins, accompanied by corresponding intracellular reduction at their cellular levels, suggesting the 15 to 30 min might be the turning point of the inflammatory response under our tested conditions.

We also noticed that there were several granule proteins that were released specifically depending on the inflammatory signals applied. For instance, the azurophilic protein BPI showed a release pattern in the supernatant of the LPS and PMA conditions (Fig. 4*E*) but not in the TNF-α condition. MPO, another well-known azurophilic protein, showed an “increase” of the pattern in the supernatant of the TNF-α- and PMA-treated cells but not in the LPS condition. In addition, S100A12, the neutrophil activation marker protein with increased serum level in sepsis patients (30), exhibits a PMA-specific release pattern from neutrophils (Fig. 4*F*). The gelatinase protein CD177, specifically expressed in neutrophils, exhibited a higher and sustained release level in PMA-treated neutrophils than in LPS and TNF-α treatment (Fig. 4*F*). CD177 was recently reported to have significantly elevated serum levels in severe coronavirus disease 2019 patients compared with moderate cases (31). The correlation between the inflammation-mediated release of the neutrophil granule proteins and their increased serum levels in patients of inflammations is of particular interest for further investigations in the future toward potential clinical consequences.

However, it is also worthy of notification that not all granule proteins were released with a concordant decreasing pattern of cellular expression, as in the case of LTF, MMP9, FCN1, and FCGR3B (Fig. 4, *C* and *D*). For example, the cellular expression of MPO remained unchanged during the time course in the PMA-treated neutrophils (Fig. 4*E*). Similar situations were also observed in other proteins such as azurophilic granule proteins, BPI, AZU1, and ELANE (Fig. 4*E* and Supplemental Fig. S3*B*), suggesting complicated regulatory mechanisms governing the degranulation program in the neutrophil.

In the evaluation of the expression patterns of other canonical granule proteins, we noticed that several proteins did not show any “increase” in the pattern of release under any of the tested stimulation conditions although they were identified in the isolated granule fractions in the previous proteomic studies (9, 10), including S100A8, S100A9, and ARG1 (Fig. 4*G* and Supplemental Fig. S3*C*). It remains to be characterized whether these proteins could be released upon other stimulation conditions in future studies.

To distill a consensus profile for each granule type, we also compared the cellular/secreted patterns of known azurophilic granule, specific granule, gelatinase granule, and SV markers. As shown in Supplemental Figure S4, given that PMA-treated samples resulted in the most complete spectrum of granule protein release, we analyzed the cellular/secreted patterns of known granule marker proteins in PMA-treated neutrophils. Azurophilic granule marker proteins (*e.g.*, AZU1, MPO, ELANE, and PRTN3) are significantly released around 30 min to 1 h poststimulation (Supplemental Fig. S4*A*), whereas specific granule markers (*e.g.*, LCN2, LTF, MMP8, CHI3L1, CRISP3, TCN1, LRG1, and OLFM4), gelatinase granule markers (*e.g.*, FCN1, FGL2, MMP9, CAMP, CHIT1, CD93, and PGLYRP1), and SV markers (*e.g.*, FCGR3B, CD14, and CD11b) are significantly released 15 min to 30 min poststimulation (Supplemental Fig. S4, *B*–*D*). These findings demonstrate that specific granules, gelatinase granules, and SVs undergo prioritized secretion compared with azurophilic granules in neutrophil responses to stimulus, in consistent with the neutrophil degranulation model. Furthermore, specific granules, gelatinase granules, and SVs exhibited an “increase” pattern in their secreted levels with a coordinated “decrease” pattern in cellular level, whereas azurophilic granule marker proteins showed secretion without substantial depletion of cellular pools in the PMA condition.

On the other hand, two previously reported granule proteins, QSOX1 and TIMP-2, showed the typical granule exocytosis pattern with the time-resolved “increase” in the supernatant and “decrease” in the cell (Supplemental Fig. S5, *A* and *B*). Their exocytosis patterns were very similar to that of LTF (known specific granule protein) and MMP9 (known gelatinase granule protein) (Fig. 4*C*). Considering the localizations of the canonical granule proteins were mainly inferred from the subcellular proteomics data, we conducted an immunomicroscopy assay to validate their subcellular localizations. As shown in Supplemental Figure S5, *C* and *D*, QSOX1 and TIMP-2 both showed clear colocalization with LTF and MMP9, suggesting their possible comobilization with the known granule marker proteins. Proteins exhibiting similar secretion kinetic profiles are likely localized within the same granule subtypes. Based on this recognition, we identified additional proteins exhibiting patterns similar to known granule markers as granule protein candidates (Supplemental Table S5). Further immunomicroscopic studies will be highly demanded to validate their precise subcellular localization.

Taken together, our integrated proteomics approach has delineated the specific release profiles of neutrophil granule proteins by characterizing their time-dependent expression in both cellular and supernatant compartments. These data are in line with the subcellular proteomics data reported previously. Furthermore, for the first time, these reported neutrophil granule proteins were systematically evaluated by their functional release properties under multiple inflammatory conditions. Here, we reported a dataset of all 468 neutrophil proteins with varied release patterns that highly correlated with inflammatory environments (Supplemental Dataset), which would shed light on these valuable molecules that are well-dictating neutrophil function.

### Neutrophil Release Diversified Granule Proteins to Cope With Different Inflammatory Conditions

**Fig. 5:**
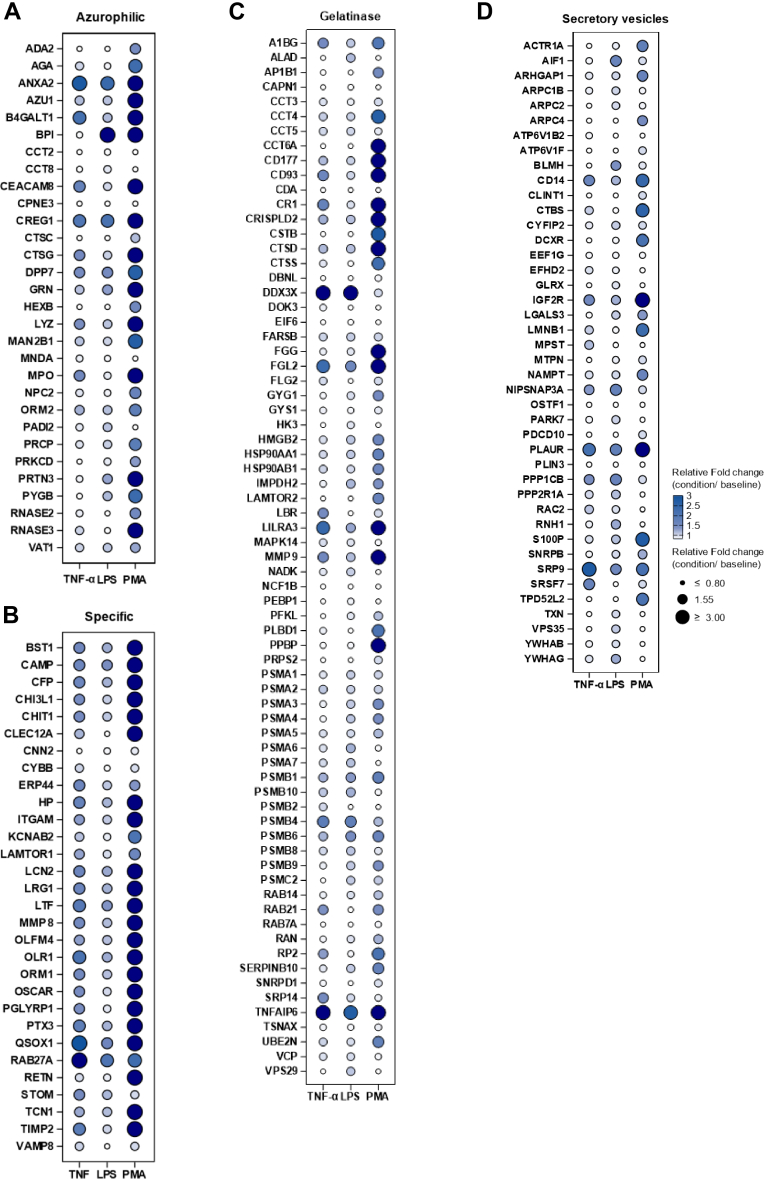
**Stimulus-specific exocytosis pattern of proteins from neutrophil azurophil granules, specific granules, gelatinase granules, and secretory vesicles.***Dot* plots display fold changes (stimulated condition *versus* baseline at 2 h) of the granule proteins in different inflammatory conditions. *A*, azurophil granule proteins, (*B*) specific granule proteins, (*C*) gelatinase granule proteins, and (*D*) secretory vesicles. *Circle* size/color gradient corresponds to the relative fold change. Proteins localized to multiple granule subsets are excluded from this visualization.

To provide a holistic and quantitative understanding of the functional release capabilities of these granule proteins in our dataset, we summarized their exocytosis patterns by presenting their “release level” in the supernatant of neutrophils under different inflammatory conditions (Fig. 5). Proteins with ambiguous and controversial distributions across granule subtypes in previous proteomic studies are not included here. The relative FCs (stimulated *versus* control condition) at 2 h (the longest time point to ensure significant accumulation of granule proteins in our assay) were used to represent the quantitative release level of each granule protein. As shown in Figure 5, *A*–*D*, well-known abundant granule proteins in azurophilic granules (*e.g.*, AZU1, BPI, CTSG, and MPO), specific granules (*e.g.*, CHI3L1, LCN2, LTF, and OLFM4), and gelatinase granules (*e.g.*, CD177, FCN1, and MMP9) were mobilized to exocytosis in varying amounts. We observed significantly higher levels of protein release in the majority of studied granules under the PMA treatment compared with the LPS and TNF-α treatments. This is consistent with the role of PMA, a PKC activator, as a potent stimulator in various cell types, including monocytes, neutrophils, and cancer cells (32, 33). In a comparison of the LPS and TNF-α treatments, several granule proteins displayed a stimuli-dependent release manner. For example, azurophilic protein BPI was induced in the LPS-treated neutrophils but not the TNF-α-treated neutrophils. In contrast, MPO and CTSG exhibited higher release levels in the TNF-α-treated neutrophils compared with the LPS-treated neutrophils. These results indicated that neutrophils feature distinct exocytosis patterns for granule proteins across different inflammatory stimulations. Our proteomics data serve as a valuable resource for investigating the detailed exocytosis patterns of individual neutrophil granule proteins under different inflammatory environments.

### The Secretion of Non-canonical Released Proteins by the Stimulated Neutrophils

**Fig. 6:**
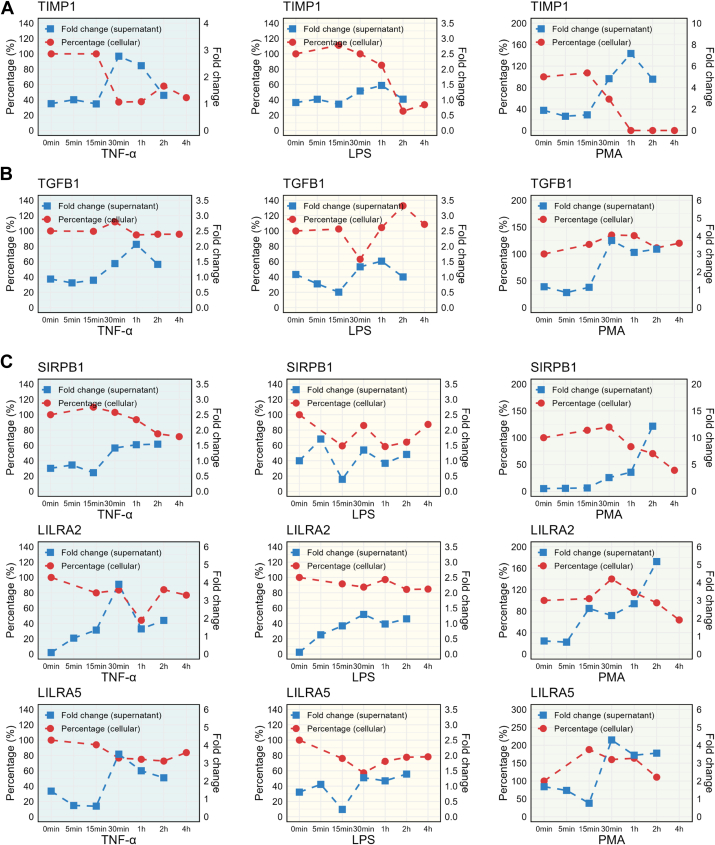
**The secretion of noncanonical granule proteins in stimulus-activated neutrophils.** Line graph showing the cellular and supernatant expression patterns of novel neutrophil-secreted proteins in the TNF-α condition (*blue background*), LPS condition (*yellow background*), and PMA condition (*green background*). *A*, anti-proteinase TIMP-1, (*B*) TGF-β1, and (*C*) immune receptors: SIRPB1, LILRA2, and LILRA5. PMA, phorbol 12-myristate 13-acetate; TNF-α, tumor necrosis factor alpha.

In addition to the 226 canonical granule proteins reported previously, we also identified 242 non-canonical released proteins, of which 20.4% were predicted to contain a signal peptide (Supplemental Fig. S6*A*), suggesting their potential secretion *via* the classical pathway. Another 33.1% of them were predicted to be secreted through nonclassical pathways (Supplemental Fig. S6*B*). In addition, 10.7% were predicted to contain transmembrane domains (Supplemental Fig. S6*C*), and 59.1% were previously reported to be secreted extracellularly *via* vesicles (Supplemental Fig. S6*D*). In total, 177 of these 242 proteins (73%) were predicted to be secreted from neutrophils (Supplemental Table S4). These potential secreted proteins were dominated by endopeptidase inhibitors (Supplemental Fig. S6*E*). For example, the TIMP-1, a natural inhibitor of metallomatrix proteases, exhibited typical release pattern, particularly upon the PMA and TNF-α stimulations (Fig. 6*A*). Unlike its granule-localized family member TIMP-2, TIMP-1 was predominantly found in vesicles lacking classical granule markers (34). TIMP-1 was reported as a multifunctional cytokine that can modulate the activity of most innate and adaptive immune cell subtypes (35). Several clinical studies have reported elevated serum levels of TIMP-1 in patients of colorectal cancer and lung cancer, suggesting TIMP-1 as a useful serum biomarker for the diagnosis and prognosis of cancers and inflammatory disorders (36, 37, 38). Our identification of the inflammation-stimulated TIMP-1 secretion by neutrophils suggested that the observed elevation in the patient serum might also have a neutrophil-mediated mechanism.

In addition to the protease inhibitors, we also observed the inducible exocytosis of TGF-β1 in the activated neutrophils (Fig. 6*B*). TGF-β1 has profound effects on many other immune cells, including all classes of lymphocytes, macrophages, and dendritic cells (39). Monocytes/macrophage-derived TGF-β1 were reported to interact with intestinal epithelial cells and promote intestinal regeneration (40). Therefore, the activated neutrophils may utilize the secreted TGF-β1 to interact with other effector cells, including themselves, as TGF-β1 was reported as a pleiotropic cytokine to promote neutrophil migration (35). This potential TGF-β1-mediated autocrine mechanism in the activated human neutrophils may be in line with a previous study in which mouse peritoneal neutrophils triggered TGF-β1 production for self-regulation of neutrophil inflammatory function (41).

Furthermore, we also detected three immune receptors, SIRPB1, LILRA2, and LILRA5, with increased release levels induced by all three inflammatory signals (Fig. 6*C*). These immune receptors were reported to be widely expressed on various immune cells (such as monocytes, macrophages, and dendritic cells) and are able to regulate multiple cellular functions *via* their activating and inhibitory signal motifs (42). For example, activation of LILRA2 on monocytes could impair the granulocyte–macrophage colony-stimulating factor–induced monocyte differentiation into immature dendritic cells (43). Crosslinking and activation of LILRA5 could induce monocyte activation and cytokine release (44, 45). Monotherapy targeting SIRPB1 was reported to promote the cytotoxic and phagocytic activity of macrophages against tumor cells (46, 47). Detection of these membrane proteins in the supernatant of the stimulated neutrophils in our study serves as a surprise. While further evaluation of their cellular expression indicated varied levels depending on different stimulation conditions, their inducible release across all three tested inflammatory stimulations suggested that perhaps these membrane receptors, at least their soluble forms, might play functional roles in inflammatory responses.

Altogether, applying the integrated proteomic approach has led to identifying a suite of key inflammatory mediators that were released by neutrophils but were not identified as the canonical granule proteins in the previous studies (9, 10). Whether these noncanonical released proteins are candidates for novel granule proteins awaits validation in the future. However, their functional release patterns upon various inflammatory stimulations will extend our understanding of neutrophil-derived proteins as regulators of immune modulation.

### Inflamed Neutrophils Release Their Granule Membrane Proteins Into the Extracellular Compartment Through MMP/ADAM-Mediated Proteolytic Shedding

**Fig. 7:**
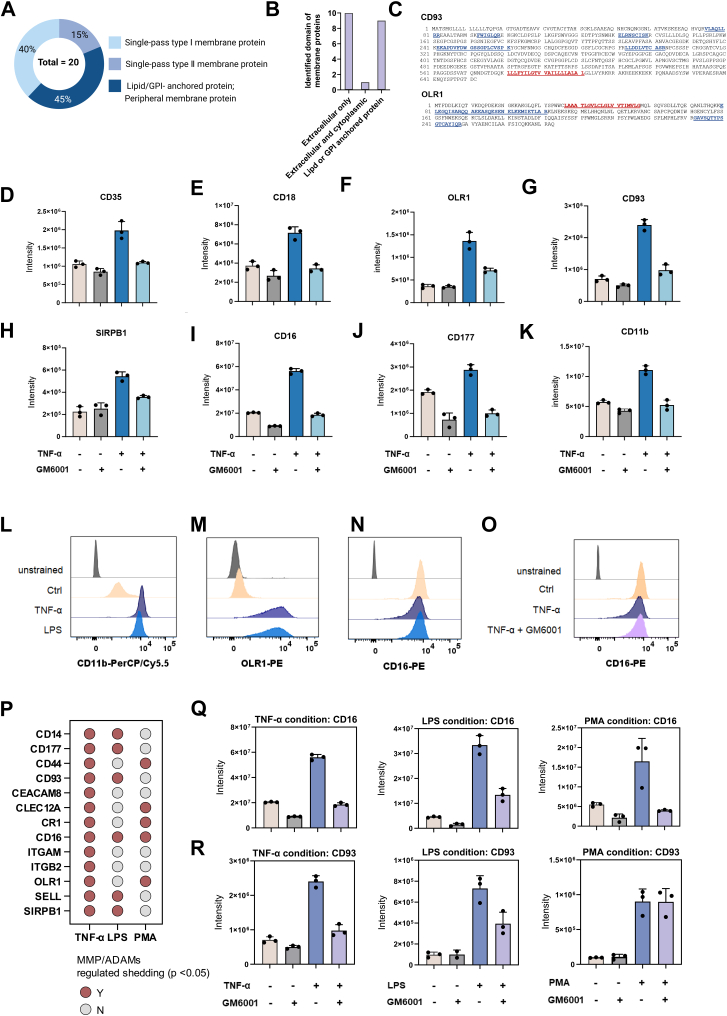
**Multiple granule membrane proteins are released into extracellular space *via* ectodomain shedding.***A*, the interaction types of released membrane proteins with lipid bilayers. *B*, the domain topology of these membrane proteins identified in MS/MS. Histogram showing that the majority of the released membrane proteins are identified by their extracellular domain in MS/MS. *C*, the identified sequence of the single-pass type I membrane protein CD93 and single-pass type Ⅱ membrane protein OLR1, as determined by MS/MS analysis. The transmembrane domains were marked in *red*, and the sequence detected by MS was highlighted in *blue*. *D*–*K*, human neutrophils (1 × 10^6^) in 200 μl medium were treated with/without TNF-α (final concentration: 20 ng/ml) in the presence of GM6001 (2 μM) or not, for 1 h. Shown is the average ± SD of the CD35 (*D*), CD18 (*E*), OLR1 (*F*), CD93 (*G*), SIRPB1 (*H*), CD16 (*I*), CD177 (*J*), and CD11b (*K*) expression in the medium under different conditions. *L*–*N,* neutrophils were stimulated with LPS/TNF-α/PBS for 1 h at 37 °C prior to evaluation of CD11b, OLR1, and CD16 surface expression by flow cytometry. Histogram plot showing the expression levels of CD11b, OLR1, and CD16 on the surface of neutrophils activated by TNF-α, LPS, and untreated (PBS). *L*, CD11b expression in the cell surface. *M*, OLR1 expression in the cell surface. *N*, CD93 expression in the cell surface. *O*, neutrophils were pretreated with or without GM6001 (2 μM) for 30 min, followed by stimulation with TNF-α in the presence of GM6001 or not for 1 h at 37 °C. Neutrophils were then stained with anti-CD16 antibody for flow cytometry analysis. Histogram plot showing CD16 expression on the surface of TNF-α-activated neutrophils with and without GM6001 presence. *P*, *dot* plots showing the sensitivity of protein substrates to the MMP/ADAM sheddase in different conditions. *Yellow color*: substrates of MMP/ADAM sheddase (yes) (*p* < 0.05). *Gray color*: not the substrate of MMP/ADAM sheddase (no). *Q*, a histogram plot showing TNF-α, LPS, or PMA induced the increase of CD16 can be inhibited by GM6001 treatment. *R,* a histogram plot showing LPS/TNF-α-induced CD93 shedding events could be inhibited by GM6001. PMA-induced CD93 shedding could not be inhibited by GM6001. ADAM, a disintegrin and metalloproteinase; LPS, lipopolysaccharide; MMP, metalloproteinase; PMA, phorbol 12-myristate 13-acetate; TNF-α, tumor necrosis factor alpha.

In further examination of these neutrophil-released granule proteins in Figure 5, we noticed that 20 of them were membrane proteins (Fig. 7*A*). These proteins include known granule membrane proteins in the specific granules (*i.e.*, CD11b, CD18), gelatinase granules (*i.e.*, CD11b, CD18, CD177, CD66b, CD35, CD93, CD16), SVs (*i.e.*, CD16, CD14), and plasma membrane proteins (*i.e.*, OLR1, CD62L/SELL). According to the current neutrophil degranulation model, these membrane structures in neutrophil granules, such as SVs, are presumably mobilized from internal storage and quickly fuse with the plasma membrane upon activation, leading to rapid alteration of neutrophil plasma membrane composition (6). Our proteomics analyses revealed that these granule membrane proteins are indeed released into the conditioned media upon inflammation. Surprisingly, the majority of the MS-detected peptides of these membrane proteins in the conditioned media samples were derived from their extracellular domain (Fig. 7*B*, Supplemental Table S6). Figure 7*C* shows the MS-detected peptide sequences of representative membrane proteins such as single-pass type I membrane protein CD93 and type Ⅱ membrane protein OLR1. This observation led us to hypothesize that neutrophils might release the granule membrane proteins *via* ectodomain shedding after these proteins were mobilized and fused with the plasma membrane upon the inflammatory activation.

Ectodomain shedding is the proteolytic cleavage of cell surface proteins, resulting in the release of part of the extracellular domains from the cell into extracellular space. It has been reported that shedding extensively existed in numerous leukocytes such as monocytes, macrophages, and NK cells, particularly during inflammation (48, 49). Ectodomain shedding is mediated by various proteases, including MMPs (such as MMP8 and MMP9) and serine proteases (such as CTSG and ELANE) (50), which were also released through granule exocytosis in the activated neutrophils (Supplemental Fig. S2). Therefore, to examine whether the release of the granule membrane proteins was through the ectodomain shedding mechanism, we applied GM6001, a broad-spectrum inhibitor of MMPs and ADAMs, in the conditioned media of neutrophils during inflammatory activations. We first selected the TNF-α stimulated condition to explore the inhibitory effects of GM6001 on ectodomain shedding activity. As shown in Figure 7, *D*–*G*, levels of CD35, CD18, OLR1, and CD93 were all increased in the conditioned media of the neutrophils following the TNF-α stimulation. And the increase of these proteins in the conditioned media was dramatically suppressed in the presence of GM6001. Similar observations were found in other membrane proteins such as immune receptors SIRPB1 and CD16 and adhesion molecules CD177 and CD11b (Fig. 7, *H*–*K*). A previous study reported that the shedding of CD11b and L-selectin (CD62L) in neutrophils facilitates their migration along blood vessels (51). The discovery of novel shedding molecules such as complement receptor (CD35), immune receptor (SIRPB1 and CLEC12A), low-density lipoprotein receptor (OLR1) extended the increasing shedding events reported in the multifaced neutrophils that would trigger further investigations on their functions and mechanisms.

The cell membrane mediated the interaction of the neutrophils with the extracellular environment *via* the various expressed receptors on the surface. We then sought to characterize the cell surface levels of these neutrophil granule membrane proteins following inflammatory activation using flow cytometry analysis. After 1 h of stimulation, we observed variable surface levels of the membrane proteins in the activated neutrophils. For example, levels of CD11b were increased under both the TNF-α and LPS conditions (Fig. 7*L*). OLR1, which had very low constitutive surface levels in the unstimulated neutrophils, also showed a significant increase in response to the stimulations (Fig. 7*M*). This suggested that for CD11b and OLR1, the replenishment *via* translocation is greater than proteolytic loss. On the other hand, the surface levels of CD16 remained unchanged following either the TNF-α or LPS stimulation, which is inconsistent with the degranulation model (Fig. 7*N*). And the surface levels of CD16 remained unchanged in the TNF-α-activated neutrophils regardless of the presence of GM6001 to inhibit the ectodomain shedding event (Fig. 7*O*). Apparently, although with ectodomain shedding occurring, the granule membrane proteins exhibited complicated translocation behavior on the cell surface, suggesting different regulatory mechanisms may exist in neutrophils to maintain the homeostasis of the surface levels of these critical membrane proteins.

Next, we investigated whether this MMP/ADAM-mediated proteolytic cleavage also occurred in the LPS and PMA conditions. We systematically compared the shedding properties of key membrane proteins under the three different conditions (Fig. 7*P*). Interestingly, these membrane proteins exhibited different sensitivity to GM6001 in neutrophils from different activations. For example, CD16, the FCγ receptor, which is found in neutrophil SVs, exhibited an MMP/ADAM-dependent shedding in all three conditions (Fig. 7*Q*). However, the induced shedding of CD93 (Fig. 7*R*), a complement receptor involved in enhancing phagocytosis, was only inhibited by GM6001 under the treatment conditions of LPS and TNF-α, but not PMA, suggesting PMA might have different mechanisms to induce CD93 release. Similar circumstances occurred with CD18, OLR1, CD177, and others (Supplemental Fig. S7, *A*–*C*). These data suggested that neutrophils can shed membrane-associated proteins through different mechanisms under various conditions, potentially involving distinctive inflammatory processes.

Taken together, our findings demonstrated that neutrophil granule membrane proteins undergo varying degrees of ectodomain shedding in response to inflammatory stimuli, resulting in the release of multiple bioactive molecules into the surrounding environment. Underlying the involvement of the internal receptor translocation, ectodomain shedding, and other unknown mechanisms in the regulation of membrane receptor expression, the density of neutrophil surface receptors could be heterogeneous and remarkably flexible for responding to the complex endogenous environment.

## Discussion

Neutrophil degranulation represents a unique mechanism efficiently utilized by the neutrophil to fight against infection and inflammation (2, 3, 4). Proteins are the major granule components released by the neutrophil as effector molecules to conduct various defense functions (6). Systematic analysis of the exocytosis profiles of neutrophil granule proteins, particularly under diverse activation conditions, would be greatly important to elucidate their functional contributions to neutrophil biology. Our time-resolved secretome analysis of neutrophils upon LPS/TNF-α/PMA stimulations has revealed 468 proteins with an inflammation-induced release pattern, including 226 annotated granule proteins based on previous neutrophil subcellular proteomic analysis on fractionated granule organelles. By integrating our secretome and cellular proteome data, we generated a valuable dataset presenting the dynamic cellular and extracellular expression patterns for each of the 468 proteins released by circulating neutrophils under various stimulation conditions. It is useful not only for reviewing and analyzing the dynamic degranulation or secretion process of individual neutrophil effector proteins upon inflammations but also could provide deep phenotypic information *via* holistic data mining. For example, by comparing the cellular and secretion kinetics of granule marker proteins, we identified multiple distinct secretion patterns. Specific gelatinase granule marker proteins (such as LTF, MMP9, FCN1, QPCT, and OLFM4) commonly showed an “increased” pattern in supernatant level with a consistent “decreased” pattern in cellular level; these proteins also exhibited a rapid protein release starting at 15 to 30 min poststimulation of either TNF-α, LPS, or PMA (Fig. 4, *C* and *D* and Supplemental Fig. S3*A*). Under PMA condition, azurophilic granule marker proteins (such as AZU1, ELANE, and MPO) showed an “increased” pattern in the supernatant level (30 min-1 h poststimulation), whereas without a “decreased” pattern in the cellular level (Supplemental Fig. S4*A*). Furthermore, several proteins exhibited a stimulus-dependent secretion pattern: azurophilic granule protein BPI only showed a release pattern- under LPS or PMA stimulation (Fig. 4*E*). S100A12 exhibits a PMA-specific release pattern from neutrophils (Fig. 4*F*). Such phenomena would have been missed if not dissecting such a large proteomic dataset systematically. These observations might also suggest that beyond the canonical classification system (biogenesis timing and cargo composition), granule subtypes may be further defined by their stimulus-specific secretion profile. Additional studies in addition to our proteomics design are required to establish such a refined classification framework. As a proof-of-principle study, we applied three infectious/inflammatory stimuli to investigate neutrophil secretion patterns. More stimuli conditions in future studies would extend to fully elucidate the stimulus-dependent release patterns of neutrophil granule proteins.

Further examination of the released granule proteins revealed 20 membrane proteins annotated in the UniProt or the Human Protein Atlas. Our study demonstrated that these membrane proteins undergo extracellular domain shedding *via* MMP/ADAM-mediated proteolytic cleavage. These include adhesion molecules (*i.e.*, CD93 and CD177), activation receptors (CD16), and immune inhibition receptors (*i.e.*, CLEC12A and SIRPB1). Ectodomain shedding of membrane proteins can generate various soluble forms that are released into surroundings, where they may interact with immune cells or soluble factors to modulate immune responses. For example, neutrophil-derived soluble CD16 has been shown to induce cytokine production in monocytes by engaging complement receptors on the cell surface (52). Similarly, soluble CD93 binds directly to the proinflammatory mediator HMGB1, blocking its receptor interactions and alleviating inflammation (53). Functional roles of other shed proteins, such as CD177, CLEC12A, and SIRPB1, remain to be elucidated in future studies.

Besides generating and releasing soluble protein “stub”, the ectodomain shedding can also modulate surface levels of cell membrane proteins to regulate cell-cell interaction and receptor-mediated signal transduction (54). Upon ectodomain shedding, the surface levels of cell membrane proteins are presumably reduced after proteolytic cleavage. This is exemplified by the reduced level of surface L-selectin (also known as CD62L) observed in stimulated neutrophils because of the ectodomain shedding (55, 56). Interestingly, we did observe variable surface levels of membrane receptors in the inflammation-activated neutrophils. Specifically, the surface levels of CD11b and OLR1 were shown to be increased in the activated neutrophils, whereas the CD16 surface expression remained at a consistent level, although ectodomain shedding of these three receptors was observed. These results imply that certain regulatory mechanisms might exist in the activated neutrophils to maintain homeostasis of the surface receptors by replenishing the proteolytic loss on the cell surface from the intracellular pool.

The other released granule proteins without a predefined membrane structure are probably located in the granule lumen. A majority of these intragranular proteins are reported proteases with diversified substrate specificities and different functions. For example, CTSG and AZU1 are serine proteases that were reported directly against their microbial substrates (6, 57). Other proteases, such as MMP8 and MMP9, were shown to facilitate neutrophil extravasation and migration by degrading extracellular matrix proteins (58). In addition, neutrophil proteases ELANE and PRTN3 were reported to proteolytically modify both mature and immature forms of cytokines (*e.g.*, IL-1β and IL-6) and chemokines (*e.g.*, CXCL8, CXCL9, and CCL2), resulting in activation or inactivation of these important cytokines/chemokines (6, 59). Therefore, *via* the regulated degranulation program, neutrophils may release substantial proteases to interacting with cytokines/chemokines to exert a significant regulatory effect in immune responses. In addition to interact with the inflammatory cells, several neutrophil-derived proteases also exhibited a direct and effective cytotoxic activity on tumor cells (60). Besides the proteases, their inhibitors are colocated inside the granule and can be induced to be exocytosed as well, such as TIMP-2. These neutrophil-borne proteases and antiproteases both play important roles in the normal functioning of the neutrophil but need to be elegantly orchestrated (61). Other granule lumen proteins also showed similar or distinct biological functions. For example, BPI showed antimicrobial activity against gram-negative organisms (62), and FCN1 can function as a pattern-recognition receptor in innate immunity (63).

Based on our observation, we summarized a model in which the neutrophil releases their granule lumen proteins and granule membrane proteins through different mechanisms for their regulatory function. As shown in Supplemental Figure S8, the granule lumen proteins were directly discharged into the extracellular compartment following the fusion of the granule membrane with the plasma membrane. Simultaneously, the granule membrane proteins were translocated to the cell surface during the membrane fusion. Considering the ectodomain shedding can occur in the plasma membrane or throughout the cellular organelles of the secretory pathway (50), three potential pathways could be involved in granule membrane protein secretion: (i) post-fusion cleavage: granule membrane proteins might be cleaved by intramembrane sheddase or soluble sheddase after membrane fusion and then released into the environment, (ii) pre-fusion cleavage: the granule membrane proteins might be cleaved on the granule prior to fusion, and (iii) vesicle-mediated pathway: secreted through vesicle-mediated pathway followed by extracellular proteolytic processing.

In our study, we also identified 242 released proteins that are not canonical granule proteins but have potential secretory capacity in neutrophils. These include the key immune regulators TIMP-1 and TGF-β1 as well as the immune receptors LILRA2, LILRA5, and SIRPB1. These neutrophil-released proteins were not reported in the previous proteomic studies on the fractionated neutrophil granules. Therefore, it is unclear whether they were released by the inflammatory neutrophils through the traditional degranulation program or other secretion mechanisms. Notably, TIMP-1 and TGF-β1, together with the granule proteins S100A12, CD177, and MMP9, were all reported with abnormal levels in the blood of patients with various dysfunctions (36, 38, 64, 65, 66). Release of the key inflammatory mediators into serum is part of neutrophil defense mechanisms in many diseases associated with infections and inflammations. Investigation of these neutrophil-released proteins can be a valuable resource to develop novel serum biomarkers of numerous diseases mediated by neutrophil activation.

Circulating neutrophils represent the class of cells with remarkable plasticity and diversity that would benefit them to cope with various complex physiological and pathological conditions (67, 68). Our integrated proteomics profiling analysis under the four representative inflammatory states also proved that the neutrophils could respond to different inflammatory signals on their differential protein expression and exocytosis patterns, as shown by the different cellular proteome and secretome in the neutrophils exposed to different environmental cues. For example, we observed that TNF-α and LPS induced relatively moderate granule protein release in comparison with the robust exocytosis triggered by the PMA treatment. Even for the granule proteins from the same granule subtype, such as the azurophilic granule proteins, BPI and MPO, they were released in a different manner in response to different stimulation signals. BPI was released by the LPS and PMA treatment but not by TNF-α. On the other hand, MPO was released by the TNF-α and PMA treatment but not by LPS. This suggests the existence of a sophisticated regulatory mechanism in the neutrophils to enable the selective exocytosis from the preformed granules. In addition to the differential granule protein exocytosis, we also observed distinct cellular expression patterns among interferon-related proteins (*i.e.*, ISG15, ISG20, IFI35, and IFI44) in activated neutrophils. Interferon-stimulated proteins are known to be activated in monocytes or macrophages by infectious stimuli (69, 70, 71). In our data, treatment of circulating neutrophils with the LPS, TNF-α, or Poly(I:C) led to significant upregulation of ISG15, ISG20, and IFI35. In the meanwhile, the PMA treatment did not induce their expressions (Supplemental Fig. S7*D*). On the other hand, IFI44 was induced in the TNF-α-, Poly(I:C)-, and PMA-stimulated neutrophils but not in the LPS-stimulated neutrophils. These findings indicate that neutrophils may possess stimulus-dependent protein expression programs that enable tailored responses to different inflammatory signals. Taken together, to gain a comprehensive proteomic map for neutrophils under different local microenvironments, such as in different tissues *in vivo*, it is strongly recommended to investigate the neutrophils (as a representative cell type) in multiple states as possible. Our “multistate proteomics” approach presented here is only a starting point as a proof-of-principle study. Further analysis on neutrophils in other contexts, including tumor and other disease conditions, will definitely provide more insights into the full spectrum of the neutrophil.

In our secretome experimental design, we monitored the protein secretion profiles across six time points, capturing the secretion dynamics of 226 granule-associated proteins in the supernatant proteome. While this temporal resolution allowed us to identify distinct release patterns and characterize secretion profiles of canonical granule markers, several limitations should be noted. The sensitivity of MS remained insufficient to detect certain low-abundance granule proteins, and very early secretion events (<5 min poststimulation) likely occurred prior to our first sampling time point and were thus left uncharacterized. As the MS detection limit advances, the time-course design of proteomic analysis will be expected to benefit more from finer tuning on sampling (≤15-min intervals) to detect ultrafast events as well as extended durations to assess sustained protein release.

To minimize cell perturbation during the neutrophil isolation procedure, several measures were implemented, including reduced handling time (<30 min from blood draw to neutrophil isolation) and the use of autologous serum-contained wash buffers. A 30-min preculture period at 37 °C was also included to stabilize neutrophils after isolation. To characterize stimulus-induced secretion patterns, PBS-treated neutrophil secretome samples (control) were included at all time points to establish baseline secretion profiles. Even under unstimulated conditions, neutrophils exhibited a low-level granule secretion, as shown by the detectable release of classical granule proteins (such as MMP9, MMP8, OLFM4, and LCN2, Supplemental Fig. S7*E*) in the control condition. This constitutive secretion suggests that neutrophils maintain basal secretory activity, which may contribute to immune homeostasis. A subset of proteins in the secretome data displayed a relatively unchanged pattern across all time points and stimulation conditions (Supplemental Table S7). These proteins may have been completely released during the preculture period and were not further inducible by any of the tested stimuli, or they might originate from nonviable cells. Several granule proteins, such as S100A8, S100A9, and ARG1 (Fig. 4*G* and Supplemental Fig. S3*C*), also showed a nonsecreted pattern, suggesting that alternative stimuli might need to be explored to examine their secretion potential.

Furthermore, the proteomic profiling analysis in this study was carried out on neutrophils of a large population. In consideration of the cellular heterogeneity among the neutrophils, a single-cell approach would be ideal to reveal the diversification in individual neutrophils in response to different environmental cues. It is awaiting the single-cell proteomic technology that is emerging and expected to be available for general practice in the near future. In addition, in this study, the neutrophil isolation from whole blood was performed using density gradient centrifugation, which exploits density differences between mononuclear leukocytes and granulocytes. However, because of the similar density between neutrophils and eosinophils, it is challenging to separate them solely based on the density-based fractionation approach. Thus, there is a limitation that the neutrophil populations in this study contain an unavoidable contamination of eosinophils (approximately 1–2%). Caution needs to be taken in careful data interpretation. For more precise functional comparisons specifically between these granulocyte subsets, antibody-based affinity purification methods would be a more suitable alternative.

To measure the proteome dynamics of the neutrophils under stimulations in a time-dependent manner, we conducted our proteomics surveys in time-course experiments with the longest evaluated time point at 4 h. While rich information on the neutrophil degranulation program has been gained and discussed here, certain later stage events during the neutrophil activation, such as the NET formation (72) and resolution of the inflammation (73, 74), would likely need longer time points to be examined poststimulation in the circulating neutrophils. It awaits specific investigation on these interesting phenomena in the future. In addition, in our experiments, the Poly(I:C)-treated neutrophils did not exhibit a significant degranulation response or the upregulation of CD11b on the cell surface. While we used the same Poly(I:C) concentration that effectively activates monocytes, these results suggest that complete neutrophil activation may require either higher stimulus doses or longer exposure durations.

To our knowledge, this is the first proteomic study profiling the temporal exocytosis pattern of known granule proteins from neutrophils in response to multiple inflammatory and infectious stimuli. This integrated cellular proteome and secretome analysis of neutrophils has driven the discovery of many crucial neutrophil signaling molecules involved in the process of degranulation and ectodomain shedding. Considering there is massive production of short-lived neutrophils in peripheral circulation (5), it is conceivable to speculate that these enormous proteases and soluble receptors degranulated from the inflamed neutrophils would play crucial roles in fighting against inflammation and infection. Therefore, characterizing the release pattern of neutrophil-borne proteins, especially the degranulation program, would shed significant light on understanding the multifaceted role of the circulating neutrophils in the human body and efficient inflammation management in therapy.

## Data Availability

The MS proteomics data have been deposited to the ProteomeXchange Consortium (http://proteomecentral.proteomexchange.org) *via* the iProX partner repository (75, 76) with the dataset identifier PXD059138. The access link for these data in ProteomeXchange is: http://proteomecentral.proteomexchange.org/cgi/GetDataset?ID=PXD059138.

## Supplemental Data

This article contains supplemental data (9, 10).

## Conflict of Interest

The authors declare no competing interests.

## References

[1] Burn G.L., Foti A., Marsman G., Patel D.F., Zychlinsky A. (2021). The neutrophil. Immunity.

[2] Mollinedo F. (2019). Neutrophil degranulation, plasticity, and cancer metastasis. Trends Immunol..

[3] Papayannopoulos V. (2018). Neutrophil extracellular traps in immunity and disease. Nat. Rev. Immunol..

[4] Rawat K., Syeda S., Shrivastava A. (2021). Neutrophil-derived granule cargoes: paving the way for tumor growth and progression. Cancer Metastasis Rev..

[5] Hidalgo A., Chilvers E.R., Summers C., Koenderman L. (2019). The neutrophil life cycle. Trends Immunol..

[6] Cassatella M.A., Östberg N.K., Tamassia N., Soehnlein O. (2019). Biological roles of neutrophil-derived granule proteins and cytokines. Trends Immunol..

[7] Faurschou M., Borregaard N. (2003). Neutrophil granules and secretory vesicles in inflammation. Microbes Infect..

[8] Borregaard N., Sørensen O.E., Theilgaard-Mönch K. (2007). Neutrophil granules: a library of innate immunity proteins. Trends Immunol..

[9] Lominadze G., Powell D.W., Luerman G.C., Link A.J., Ward R.A., McLeish K.R. (2005). Proteomic analysis of human neutrophil granules. Mol. Cell. Proteomics.

[10] Rørvig S., Østergaard O., Heegaard N.H.H., Borregaard N. (2013). Proteome profiling of human neutrophil granule subsets, secretory vesicles, and cell membrane: correlation with transcriptome profiling of neutrophil precursors. J. Leukoc. Biol..

[11] Montaldo E., Lusito E., Bianchessi V., Caronni N., Scala S., Basso-Ricci L. (2022). Cellular and transcriptional dynamics of human neutrophils at steady state and upon stress. Nat. Immunol..

[12] Ng L.G., Ostuni R., Hidalgo A. (2019). Heterogeneity of neutrophils. Nat. Rev. Immunol..

[13] Pillay J., Kamp V.M., van Hoffen E., Visser T., Tak T., Lammers J.-W. (2012). A subset of neutrophils in human systemic inflammation inhibits T cell responses through Mac-1. J. Clin. Invest..

[14] Silvestre-Roig C., Hidalgo A., Soehnlein O. (2016). Neutrophil heterogeneity: implications for homeostasis and pathogenesis. Blood.

[15] Ballesteros I., Rubio-Ponce A., Genua M., Lusito E., Kwok I., Fernández-Calvo G. (2020). Co-option of neutrophil fates by tissue environments. Cell.

[16] Hedrick C.C., Malanchi I. (2022). Neutrophils in cancer: heterogeneous and multifaceted. Nat. Rev. Immunol..

[17] Adrover J.M., Aroca-Crevillén A., Crainiciuc G., Ostos F., Rojas-Vega Y., Rubio-Ponce A. (2020). Programmed ‘disarming’ of the neutrophil proteome reduces the magnitude of inflammation. Nat. Immunol..

[18] Aquino E.N., Neves A.C., Santos K.C., Uribe C.E., Souza P.E., Correa J.R. (2016). Proteomic analysis of neutrophil priming by PAF. Protein Pept. Lett..

[19] Ross A.R.S., Lee P.J., Smith D.L., Langridge J.I., Whetton A.D., Gaskell S.J. (2002). Identification of proteins from two-dimensional polyacrylamide gels using a novel acid-labile surfactant. Proteomics.

[20] Demichev V., Messner C.B., Vernardis S.I., Lilley K.S., Ralser M. (2020). DIA-NN: neural networks and interference correction enable deep proteome coverage in high throughput. Nat. Methods.

[21] Koopmans F., Li K.W., Klaassen R.V., Smit A.B. (2023). MS-DAP platform for downstream data analysis of label-free proteomics uncovers optimal workflows in benchmark data sets and increased sensitivity in analysis of alzheimer’s biomarker data. J. Proteome Res..

[22] Zhou Y., Zhou B., Pache L., Chang M., Khodabakhshi A.H., Tanaseichuk O. (2019). Metascape provides a biologist-oriented resource for the analysis of systems-level datasets. Nat. Commun..

[23] Kumar L., E. Futschik M. (2007). Mfuzz: a software package for soft clustering of microarray data. Bioinformation.

[24] Altstaedt J., Kirchner H., Rink L. (1996). Cytokine production of neutrophils is limited to interleukin-8. Immunology.

[25] Cassatella M.A., Dixon F.J. (1999). Advances in Immunology.

[26] Tecchio C., Micheletti A., Cassatella M.A. (2014). Neutrophil-derived cytokines: facts beyond expression. Front. Immunol..

[27] Roth S., Agthe M., Eickhoff S., Möller S., Karsten C.M., Borregaard N. (2015). Secondary necrotic neutrophils release interleukin-16C and macrophage migration inhibitory factor from stores in the cytosol. Cell Death Discov..

[28] Silliman C.C., Kelher M.R., Gamboni-Robertson F., Hamiel C., England K.M., Dinarello C.A. (2010). Tumor necrosis factor-α causes release of cytosolic interleukin-18 from human neutrophils. Am. J. Physiol. Cell Physiol..

[29] Druhan L.J., Lance A., Li S., Price A.E., Emerson J.T., Baxter S.A. (2017). Leucine rich α-2 glycoprotein: a novel neutrophil Granule protein and modulator of myelopoiesis. PLoS One.

[30] Ahmed A., Dirk F., Thomas V., Jan W.O., van T., Pierre-François L. (2013). S100A12 and soluble receptor for advanced glycation end.... Shock.

[31] Yves L., Aurélie W., Boris P.H., Mélany M., Cécile L., Mathieu S. (2021). CD177, a specific marker of neutrophil activation, is associated with coronavirus disease 2019 severity and death. iScience.

[32] Besson A., Davy A., Robbins S.M., Yong V.W. (2001). Differential activation of ERKs to focal adhesions by PKC ε is required for PMA-induced adhesion and migration of human glioma cells. Oncogene.

[33] Chang Z.L., Beezhold D.H. (1993). Protein kinase C activation in human monocytes: regulation of PKC isoforms. Immunology.

[34] Price B., Dennison C., Tschesche H., Elliott E. (2000). Neutrophil tissue inhibitor of matrix metalloproteinases-1 occurs in novel vesicles that do not fuse with the phagosome. J. Biol. Chem..

[35] Schoeps B., Frädrich J., Krüger A. (2023). Cut loose TIMP-1: an emerging cytokine in inflammation. Trends Cell Biol..

[36] Meng C., Yin X., Liu J., Tang K., Tang H., Liao J. (2018). TIMP-1 is a novel serum biomarker for the diagnosis of colorectal cancer: A meta-analysis. PLoS One.

[37] Kehusmaa A., Tuomisto A., Sirniö P., Karjalainen H., Kastinen M., Tapiainen V.V. (2025). Associations of serum and tissue TIMP1 with host response and survival in colorectal cancer. Sci. Rep..

[38] Dantas E., Murthy A., Ahmed T., Ahmed M., Ramsamooj S., Hurd M.A. (2023). TIMP1 is an early biomarker for detection and prognosis of lung cancer. Clin. Transl. Med..

[39] Letterio J.J., Roberts A.B. (1998). Regulation of immune responses by TGF-β. Annu. Rev. Immunol..

[40] Chen L., Qiu X., Dupre A., Pellon-Cardenas O., Fan X., Xu X. (2023). TGFB1 induces fetal reprogramming and enhances intestinal regeneration. Cell Stem Cell.

[41] Steiger S., Harper J.L. (2013). Neutrophil cannibalism triggers transforming growth factor β1 production and self regulation of neutrophil inflammatory function in monosodium urate monohydrate crystal–induced inflammation in mice. Arthritis Rheum..

[42] van Rees D.J., Szilagyi K., Kuijpers T.W., Matlung H.L., van den Berg T.K. (2016). Immunoreceptors on neutrophils. Semin. Immunol..

[43] Lee D.J., Sieling P.A., Ochoa M.T., Krutzik S.R., Guo B., Hernandez M. (2007). LILRA2 activation inhibits dendritic cell differentiation and antigen presentation to T cells1. J. Immunol..

[44] Lewis Marffy, McCarthy A.J. (2020). Leukocyte Immunoglobulin-Like Receptors (LILRs) on Human Neutrophils: Modulators of Infection and Immunity. Front. Immunol..

[45] Borges L., Kubin M., Kuhlman T. (2003). LIR9, an immunoglobulin-superfamily–activating receptor, is expressed as a transmembrane and as a secreted molecule. Blood.

[46] Sakamoto M., Murata Y., Tanaka D., Kakuchi Y., Okamoto T., Hazama D. (2022). Anticancer efficacy of monotherapy with antibodies to SIRPα/SIRPβ1 mediated by induction of antitumorigenic macrophages. Proc. Natl. Acad. Sci..

[47] Hayashi A., Ohnishi H., Okazawa H., Nakazawa S., Ikeda H., Motegi S. (2004). Positive Regulation of Phagocytosis by SIRPβ and Its Signaling Mechanism in Macrophages. J. Biol. Chem..

[48] Bohlson S.S., Silva R., Fonseca M.I., Tenner A.J. (2005). CD93 is rapidly shed from the surface of human myeloid cells and the soluble form is detected in human plasma. J. Immunol..

[49] Morgan H.J., Rees E., Lanfredini S., Powell K.A., Gore J., Gibbs A. (2022). CD200 ectodomain shedding into the tumor microenvironment leads to NK cell dysfunction and apoptosis. J. Clin. Invest..

[50] Lichtenthaler S.F., Lemberg M.K., Fluhrer R. (2018). Proteolytic ectodomain shedding of membrane proteins in mammals—hardware, concepts, and recent developments. EMBO J..

[51] Zen K., Guo Y.-L., Li L.-M., Bian Z., Zhang C.-Y., Liu Y. (2011). Cleavage of the CD11b extracellular domain by the leukocyte serprocidins is critical for neutrophil detachment during chemotaxis. Blood.

[52] Galon J., Gauchat J.F., Mazières N., Spagnoli R., Storkus W., Lötze M. (1996). Soluble Fcgamma receptor type III (FcgammaRIII, CD16) triggers cell activation through interaction with complement receptors. J. Immunol..

[53] Huang S.-E., Kuo C.-H., Shiao S.-Y., Shen C.-R., Lee F.-T., Chang B.-I. (2023). Soluble CD93 lectin-like domain sequesters HMGB1 to ameliorate inflammatory diseases. Theranostics.

[54] Arribas J., Borroto A. (2002). Protein ectodomain shedding. Chem. Rev..

[55] Kuhns D.B., Gallin J.I., Long Priel D.A. (1995). Loss of L-Selectin (CD62L) on human neutrophils following exudation in vivo. Cell. Immunol..

[56] Li Y., Brazzell J., Herrera A., Walcheck B. (2006). ADAM17 deficiency by mature neutrophils has differential effects on L-selectin shedding. Blood.

[57] Pham C.T. (2006). Neutrophil serine proteases specific regulators of inflammation. Nat. Rev. Immunol..

[58] Lin M., Jackson P., Pearlman E. (2008). Matrix metalloproteinase-8 facilitates neutrophil migration through the corneal stromal matrix by collagen degradation and production of the chemotactic peptide Pro-Gly-Pro. Am. J. Pathol..

[59] Clancy D.M., Sullivan G.P., Moran H.B.T., Henry C.M., Reeves E.P., McElvaney N.G. (2018). Extracellular neutrophil proteases are efficient regulators of IL-1, IL-33, and IL-36 cytokine activity but poor effectors of microbial killing. Cell Rep..

[60] Cui C., Chakraborty K., Tang X.A., Zhou G., Schoenfelt K.Q., Becker K.M. (2021). Neutrophil elastase selectively kills cancer cells and attenuates tumorigenesis. Cell.

[61] Janciauskiene S., Wrenger S., Immenschuh S., Olejnicka B., Greulich T., Welte T. (2018). The multifaceted effects of Alpha1-Antitrypsin on neutrophil functions. Front Pharmacol..

[62] Levy O. (2000). A neutrophil-derived anti-infective molecule: bactericidal/permeability-increasing protein. Antimicrob. Agents Chemother..

[63] Honoré C., Rørvig S., Munthe-Fog L., Hummelshøj T., Madsen H.O., Borregaard N. (2008). The innate pattern recognition molecule Ficolin-1 is secreted by monocytes/macrophages and is circulating in human plasma. Mol. Immunol..

[64] Lévy Y., Wiedemann A., Hejblum B.P., Durand M., Lefebvre C., Surénaud M. (2021). CD177, a specific marker of neutrophil activation, is associated with coronavirus disease 2019 severity and death. iScience.

[65] Ayub S., Shafi T., Rasool R., Dangroo M.A., Bindroo M.A., Gull A. (2024). Evaluating the role of active TGF-β1 as inflammatory biomarker in Kashmiri (North-Indian) patients with systemic sclerosis: a case-control study. Adv. Rheumatol..

[66] Wittkowski H., Sturrock A., Van Zoelen, Viemann D., Van Der Poll T., Hoidal J.R., Roth J. (2007). Neutrophil-derived S100A12 in acute lung injury and respiratory distress syndrome. Crit. Care Med..

[67] Jaillon S., Ponzetta A., Di Mitri D., Santoni A., Bonecchi R., Mantovani A. (2020). Neutrophil diversity and plasticity in tumour progression and therapy. Nat. Rev. Cancer.

[68] Qu J., Jin J., Zhang M., Ng L.G. (2023). Neutrophil diversity and plasticity: implications for organ transplantation. Cell. Mol. Immunol..

[69] Bogunovic D., Boisson-Dupuis S., Casanova J.-L. (2013). ISG15: leading a double life as a secreted molecule. Exp. Mol. Med..

[70] Song R., Gao Y., Dozmorov I., Malladi V., Saha I., McDaniel M.M. (2021). IRF1 governs the differential interferon-stimulated gene responses in human monocytes and macrophages by regulating chromatin accessibility. Cell Rep..

[71] Orecchioni M., Ghosheh Y., Pramod A.B., Ley K. (2019). Macrophage polarization: different gene signatures in M1(LPS+) vs. classically and M2(LPS–) vs. alternatively activated macrophages. Front. Immunol..

[72] Brinkmann V., Reichard U., Goosmann C., Fauler B., Uhlemann Y., Weiss D.S. (2004). Neutrophil extracellular traps kill bacteria. Science.

[73] Greenlee-Wacker M.C. (2016). Clearance of apoptotic neutrophils and resolution of inflammation. Immunol. Rev..

[74] Serhan C.N., Savill J. (2005). Resolution of inflammation: the beginning programs the end. Nat. Immunol..

[75] Chen T., Ma J., Liu Y., Chen Z., Xiao N., Lu Y. (2022). iProX in 2021: connecting proteomics data sharing with big data. Nucleic Acids Res..

[76] Ma J., Chen T., Wu S., Yang C., Bai M., Shu K. (2019). iProX: an integrated proteome resource. Nucleic Acids Res..

