# Supplementary material for: Proteomic Portrait of Degranulation Program in Human Circulating Neutrophils Upon Multi-Inflammatory and Infectious Activation
Source: Mol Cell Proteomics. 2025 Sep 29;25(1):101078. doi: 10.1016/j.mcpro.2025.101078 (PMC12804025; doi:10.1016/j.mcpro.2025.101078)

Supplemental Figure 1

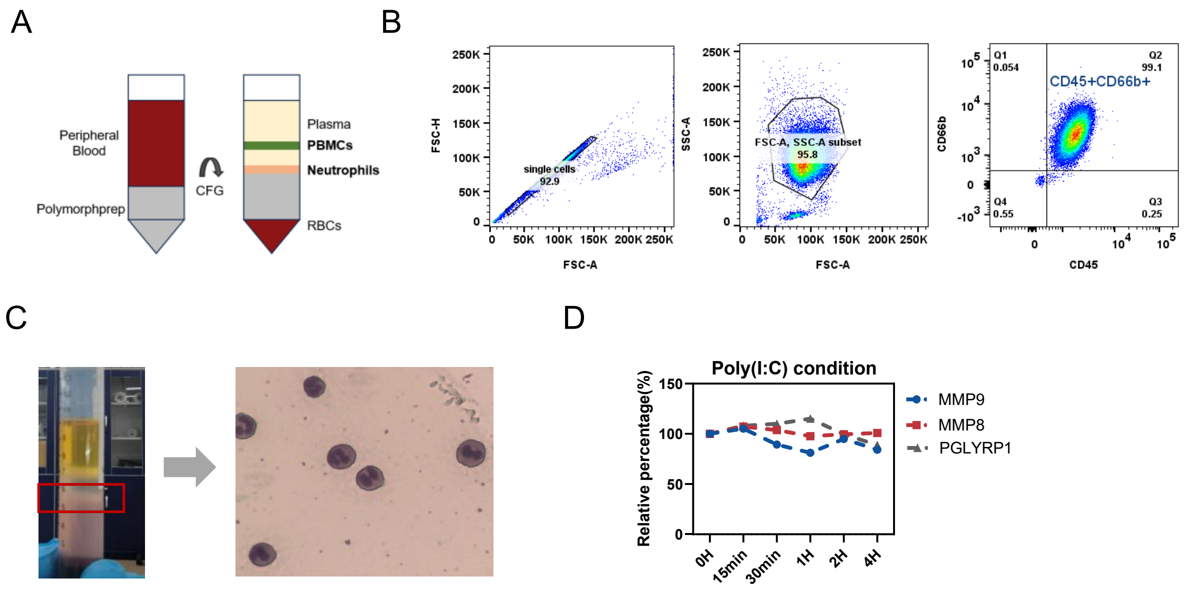

Supplemental Figure 2

TNF-α induced degranulation

148

Azurophilic: ADA2, ANXA2, AZU1, B4GALT1, CALR, CAMP, CAP1, CCT2, CEACAM8, CHIT1, CPNE3, CRISP3, CTSG, CTSZ, DPP7, ELANE, GCA, GRN, HEXB, HSPA5, LCN2, LTF, LYZ, MAN2B1, MMP9, MNDA, MPO, MYH9, OLFM4, ORM2, PDIA3, PRTN3, PYGB, RETN, RNASE2, RNASE3, SDCBP, STX7, VAMP8, VAT1, VNN1

Specific: ALB, B2M, BST1, CALR, CAMP, CFP, CHI3L1, CHIT1, CRISP3, CTSZ, EHD1, ERP44, FCN1, GCA, HBB, HP, HSPA5, ITGB2, LAMTOR1, LCN2, LRG1, LTF, MGAM, MMP8, MMP9, MYH9, OLFM4, OLR1, ORM1, OSCAR, PDIA3, PGLYRP1, PTX3, QPCT, QSOX1, RETN, SDCBP, STX7, TCN1, TIMP2, TLN1, VAMP8, VNN1, YWHAZ

Gelatinase: A1BG, ALAD, ALB, AP1B1, B2M, CA4, CALR, CAMP, CAPN1, CCT3, CCT5, CD177, CD93, CDA, CHIT1, CORO7, CR1, CRISPLD2, CSTB, CTSZ, DBNL, DDX3X, DOK3, EIF6, ELANE, FCGR3B, FCN1, FERMT3, FGG, FGL2, GCA, GYS1, HBB, HP, HSP90AA1, HSP90AB1, HSPA5, ITGB2, LBR, LCN2, LILRA3, LTF, MGAM, MMP9, MYH9, MYL6, NADK, NCF1B, OLFM4, PDIA3, PEBP1, PFKL, PGLYRP1, PRPS2, PSMA1, PSMA2, PSMA4, PSMA5, PSMA6, PSMA7, PSMB1, PSMB10, PSMB2, PSMB4, PSMB6, PSMB8, PSMB9, PSMC2, QPCT, RAB11B, RAB14, RAB21, RAB7A, RAN, RETN, SDCBP, SNRPD1, SRP14, STX7, TLN1, TSNAX, VAMP8, VCP, VNN1, VPS29, YWHAZ

Secretory: ARHGAP1, ARPC1B, ATP6V1B2, CA4, CAP1, CD14, CLINT1, CORO7, CTBS, EEF1G, EFHD2, FCGR3B, FERMT3, IGF2R, MPST, MYH9, MYL6, OSTF1, PARK7, PDCD10, PLAUR, PLIN3, PPP1CB, PPP2R1A, RAB11B, RNH1, SRP9, SRSF7, STX7, TLN1, TPD52L2, VPS35, YWHAB, YWHAG

LPS induced degranulation

81

Azurophilic: ANXA2, AZU1, BPI, CALR, CCT8, CDC42, CREG1, CTSC, DPP7, GNAI2, MAN2B1, OLFM4, PADI2, PRKCD, PRTN3, PYGB, RAB27A, STOM, TUBA1A, VAMP8, VAT1

Specific: CALR, CDC42, CLTC, CNN2, FCN1, GNAI2, HBB, OLFM4, PADI4, QPCT, QSOX1, RAB27A, STOM, TCN1, TLN1, TUBA1A, TUBA4A, VAMP8

Gelatinase: ALAD, AP1B1, CALR, CDC42, CORO7, CR1, CTSD, DDX3X, FARSB, FCN1, FERMT3, FGL2, FLG2, GNAI2, HBB, HK3, HSP90AA1, IMPDH2, LBR, NADK, OLFM4, PADI4, PFKL, PRPS2, PSMA1, PSMA2, PSMA4, PSMA5, PSMA6, PSMA7, PSMB10, PSMB4, PSMB6, PSMB8, PSMB9, PSMC2, QPCT, RAB27A, RAN, RP2, STOM, TLN1, TSNAX, TUBA1A, VAMP8, VCP, VPS29

Secretory: AIF1, ARPC2, ATP6V1F, BLMH, CORO7, CTBS, CYFIP2, FCGR3B, FERMT3, GLRX, MTPN, NIPSNAP3A, PLAUR, PPP1CB, RAC2, SNRPB, TLN1, TPD52L2, TUBA1A, TUBA4A, TXN, YWHAG

PMA induced degranulation

147

Azurophilic: AGA, ANXA2, AZU1, B4GALT1, BPI, CAMP, CANX, CCT2, CEACAM8, CHIT1, CREG1, CRISP3, CTSC, CTSG, CTSZ, CYBB, DPP7, ELANE, GCA, GGH, GLIPR2, GRN, HEXB, HRNR, HSPA5, LCN2, LTF, LYZ, MAN2B1, MMP9, MPO, NPC2, OLFM4, ORM2, PDIA3, PRCP, PRTN3, PYGB, RAB27A, RAP1B, RETN, RNASE2, RNASE3, S100A9, SDCBP, STX7, TOLLIP, TUBA1A, VIM

Specific: ALB, ANXA3, B2M, BST1, CAMP, CANX, CAPZA1, CFP, CHI3L1, CHIT1, CLEC12A, CLTC, CRISP3, CTSZ, CYBB, DSP, ERP44, FCN1, FGB, GCA, GGH, GLIPR2, HP, HRNR, HSPA5, ICAM3, ITGAM, ITGB2, KCNAB2, LCN2, LRG1, LTF, MGAM, MMP8, MMP9, MVP, OLFM4, OLR1, ORM1, OSCAR, P4HB, PDIA3, PGLYRP1, PTX3, QPCT, QSOX1, RAB27A, RAP1B, RETN, S100A12, S100A9, SDCBP, STX7, TCN1, THBS1, TIMP2, TLN1, TOLLIP, TUBA1A, VIM, VNN2

Gelatinase: AP1B1, B2M, CA4, CAMP, CANX, CAPZA1, CCT4, CCT6A, CD177, CD93, CHIT1, CLEC12A, CRISPLD2, CSTB, CTSD, CTSS, CTSZ, CYBB, DSP, ELANE, FARSB, FCGR3B, FCN1, FGB, FGG, FGL2, GCA, GYG1, HMGB2, HP, HRNR, HSP90AA1, HSP90AB1, HSPA5, ICAM3, ITGAM, ITGB2, LAMTOR2, LCN2, LILRA3, LTF, MAPK14, MGAM, MMP9, MVP, OLFM4, P4HB, PDIA3, PEBP1, PGLYRP1, PLBD1, PPBP, PSMA1, PSMA2, PSMA3, PSMA4, PSMA5, PSMA6, PSMA7, PSMB1, PSMB10, PSMB2, PSMB4, PSMB6, PSMB8, PSMB9, QPCT, RAB11B, RAB14, RAB27A, RAP1B, RETN, S100A9, SDCBP, SERPINB10, SNAP23, SNRPD1, SRP14, STX7, THBS1, TLN1, TNFAIP6, TOLLIP, TUBA1A, UBE2N, VIM, VNN2, VPS29

Secretory: ACTR1A, ANXA3, ARHGAP1, ARPC4, CA4, CAPZA1, CTBS, DCXR, FCGR3B, IGF2R, LGALS3, LMNB1, NAMPT, PLAUR, RAB11B, S100A12, S100A9, S100P, SNAP23, SNRPB, STX7, TLN1, TPD52L2, TUBA1A, VIM, YWHAG

Supplemental Figure 3

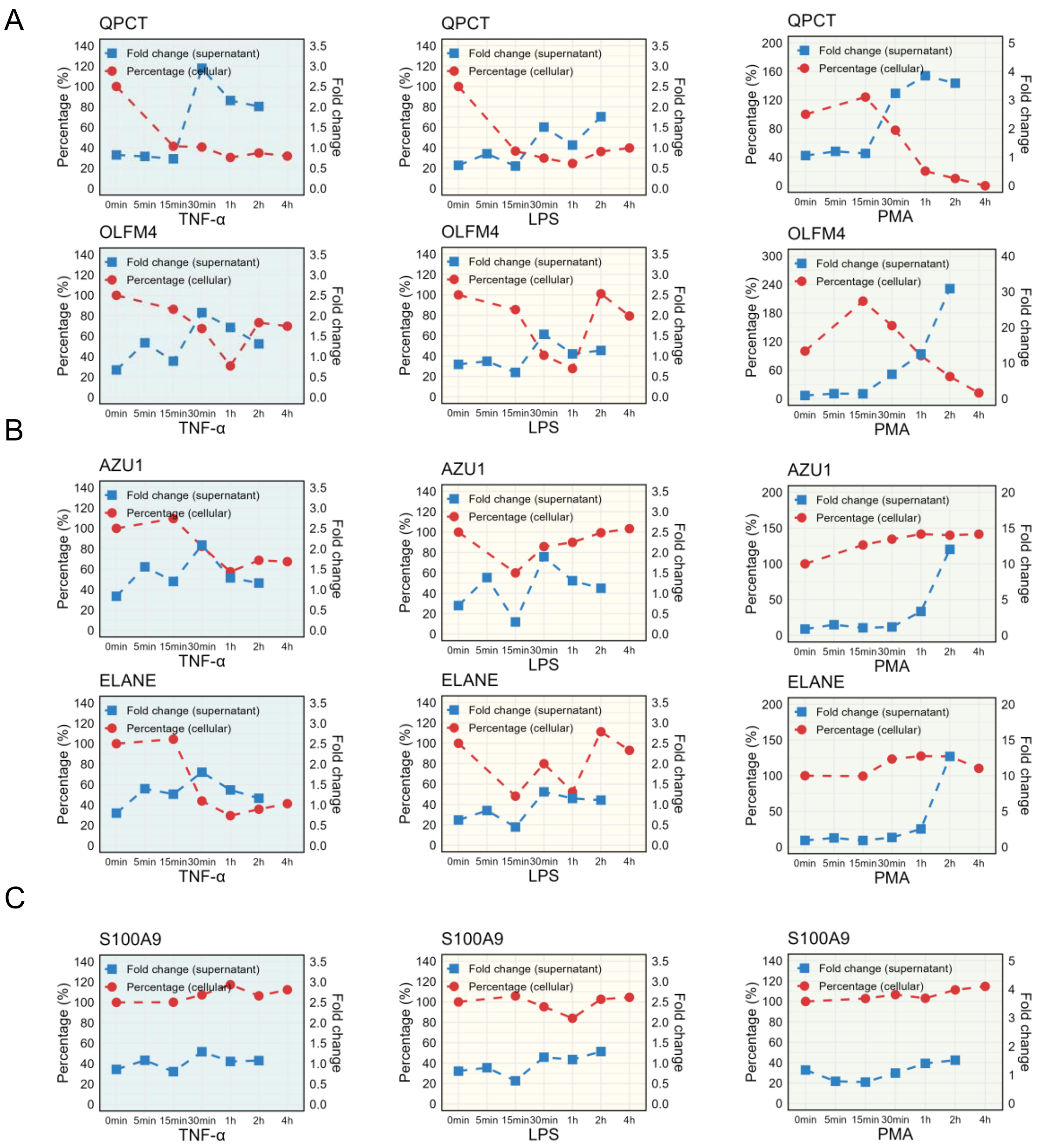

Supplemental Figure 4

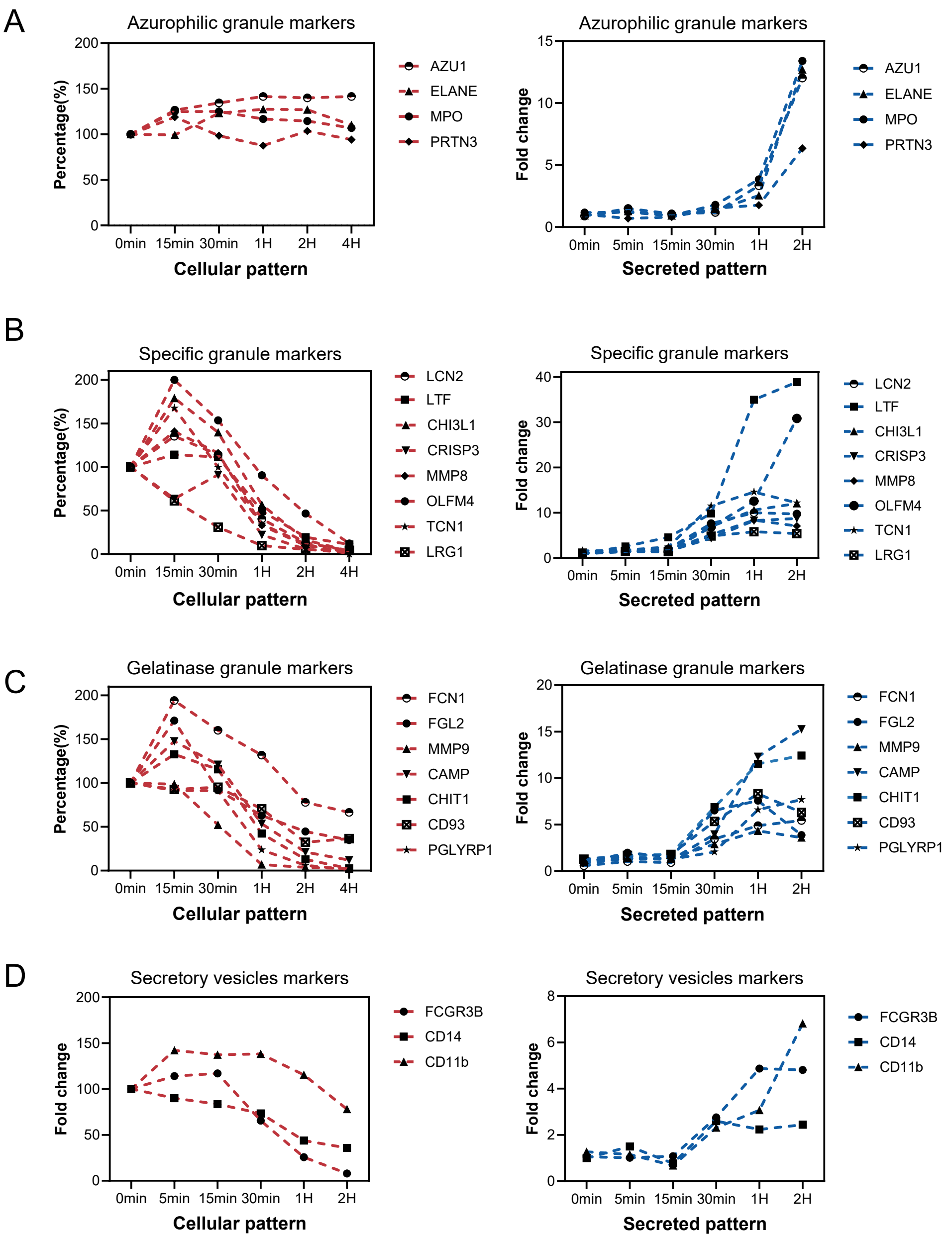

Supplemental Figure 5

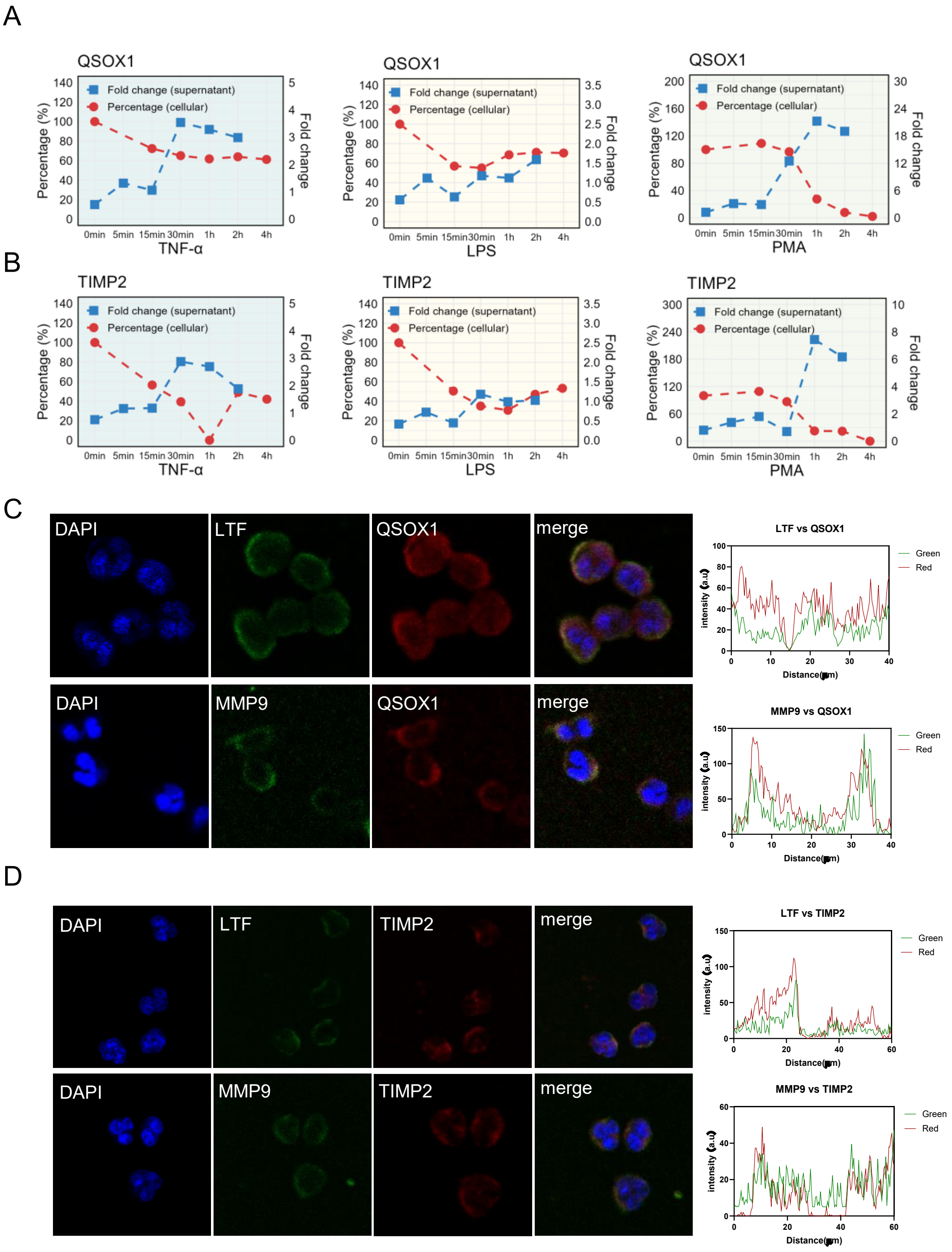

Supplemental Figure 6

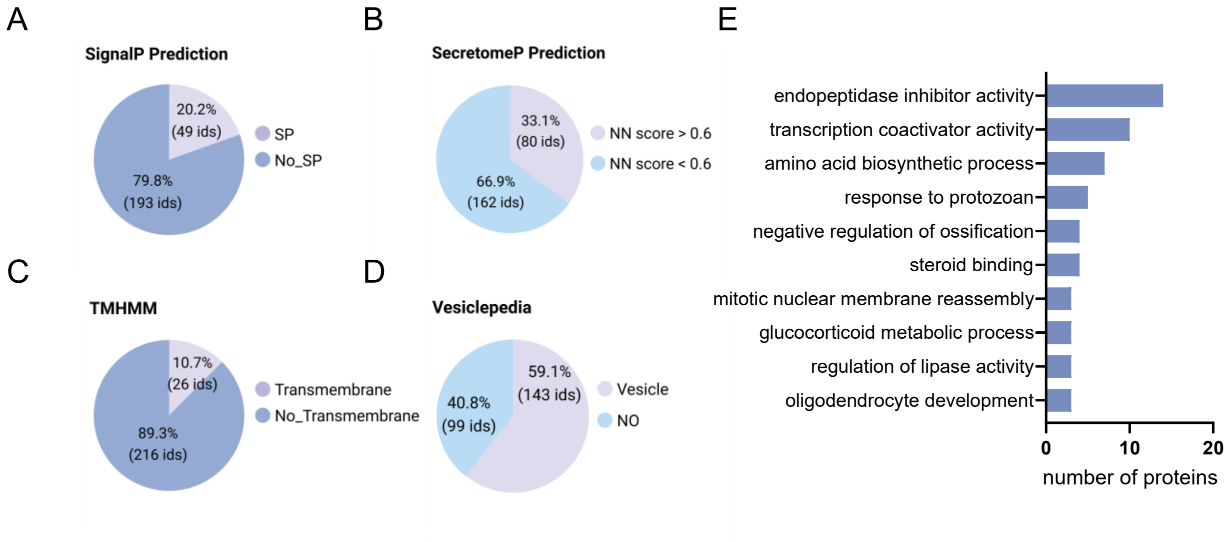

Supplemental Figure 7

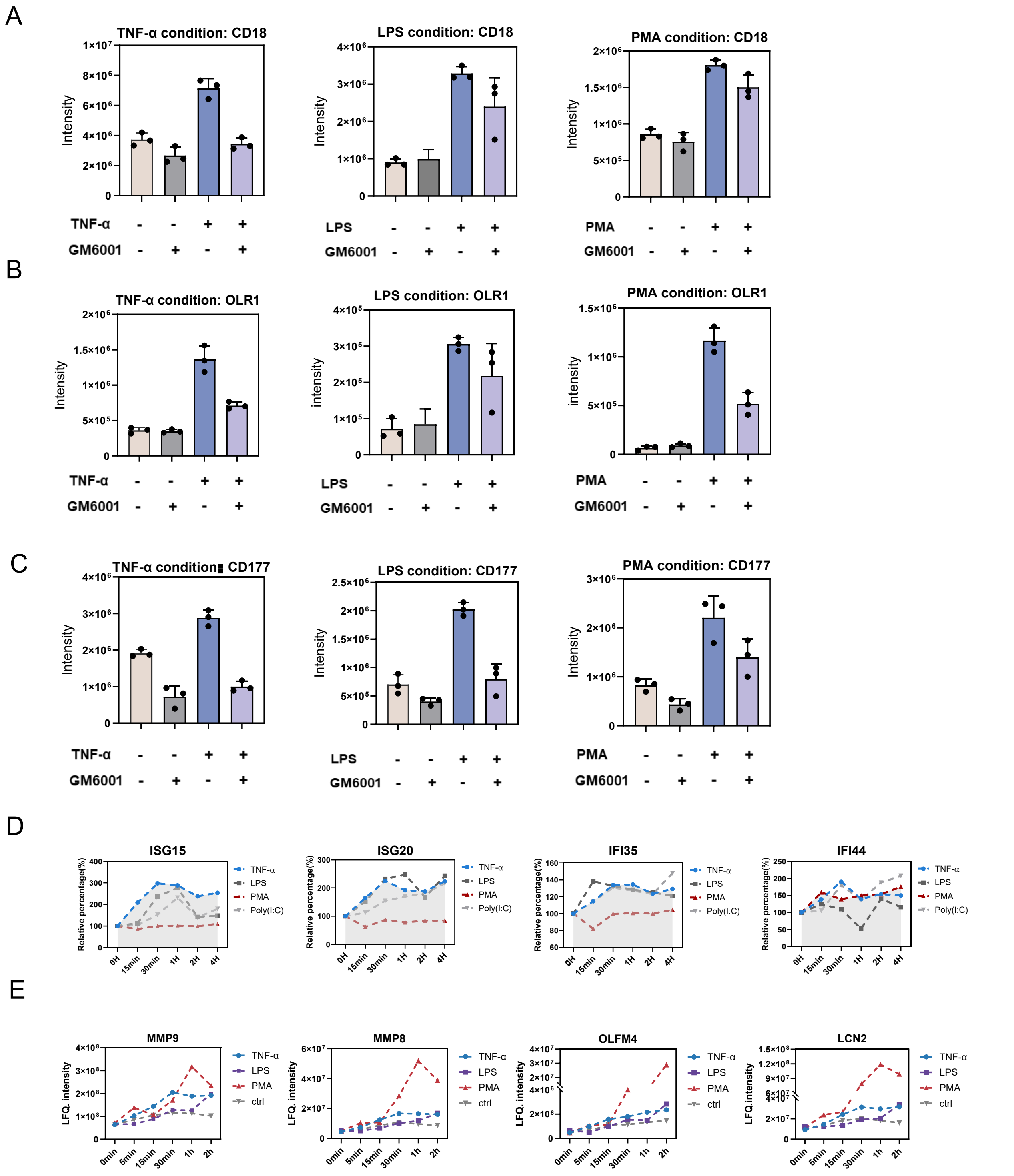

Supplemental Figure 8

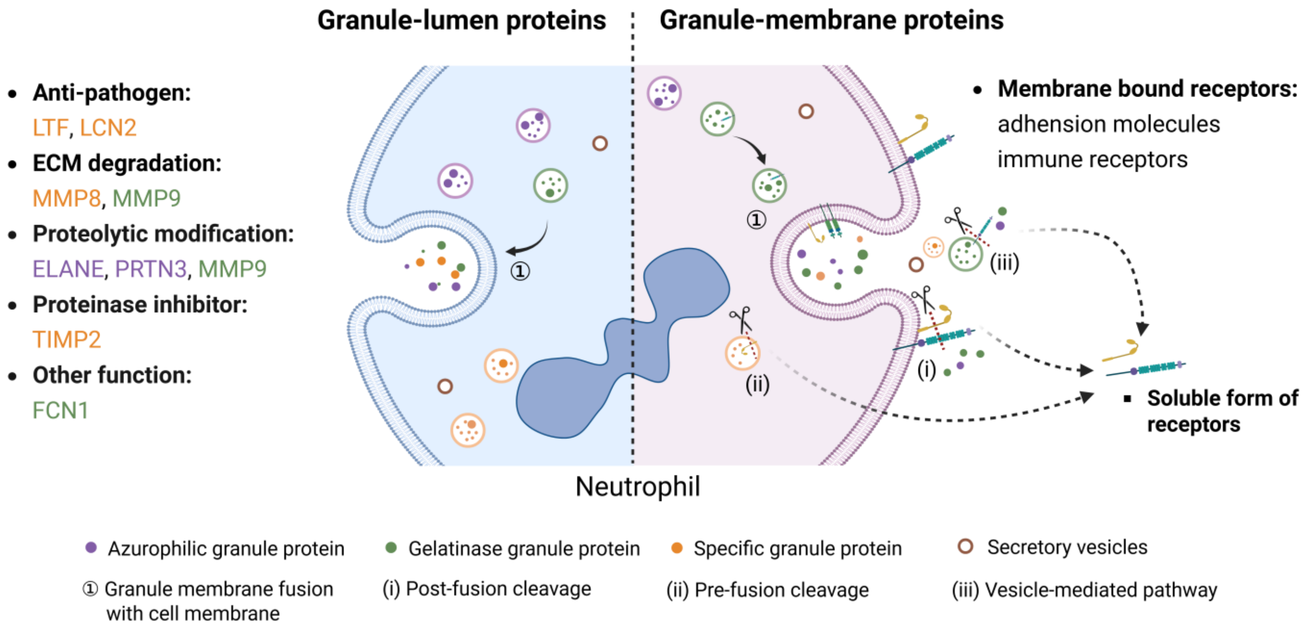

Supplement: Supplemental Figures [file mmc9.pdf]
