# Supplementary figures and images for: Proteomic Portrait of Degranulation Program in Human Circulating Neutrophils Upon Multi-Inflammatory and Infectious Activation
Source: Mol Cell Proteomics. 2025 Sep 29;25(1):101078. doi: 10.1016/j.mcpro.2025.101078 (PMC12804025; doi:10.1016/j.mcpro.2025.101078)

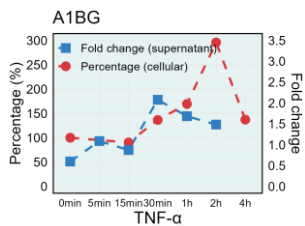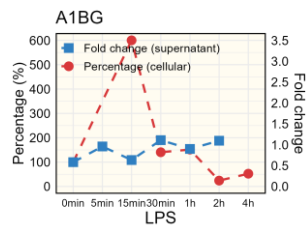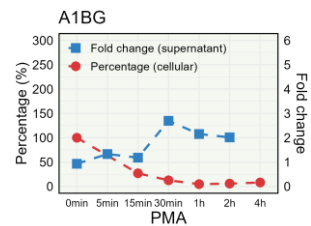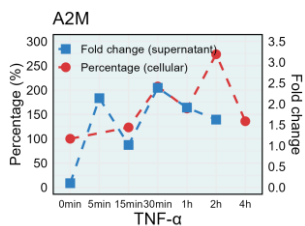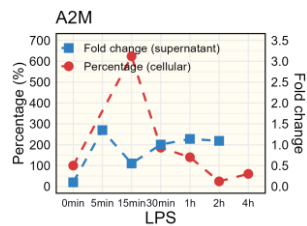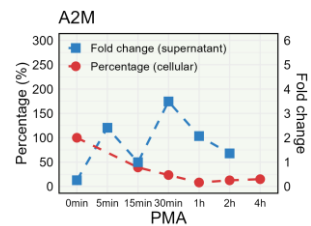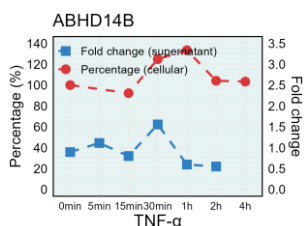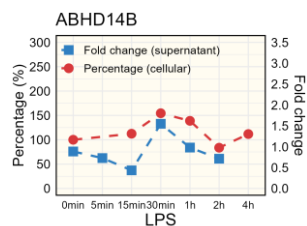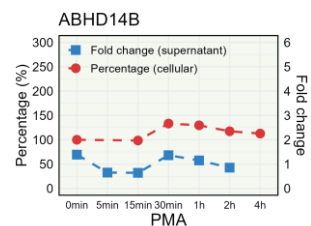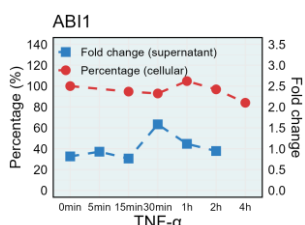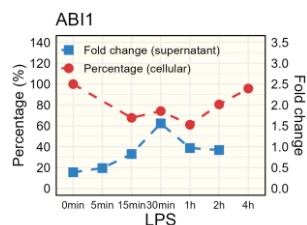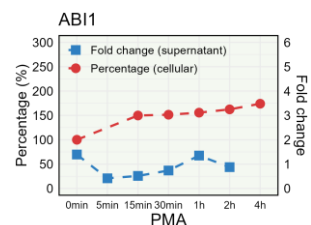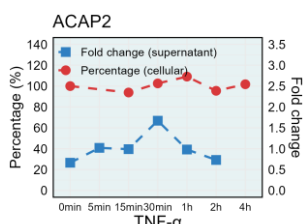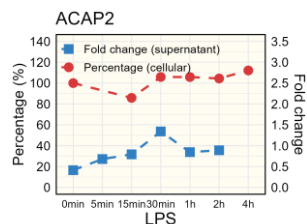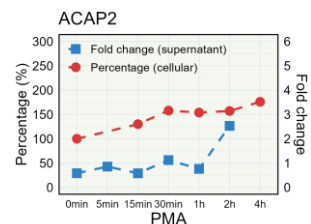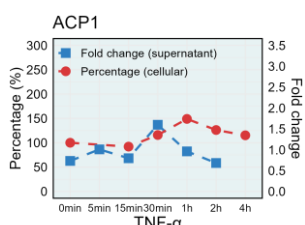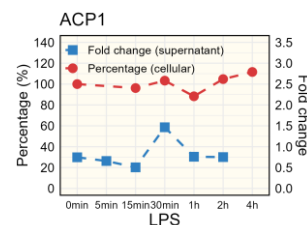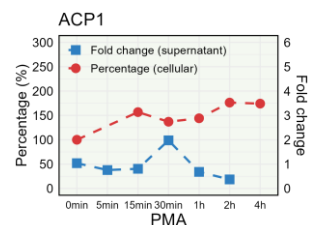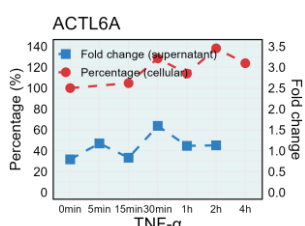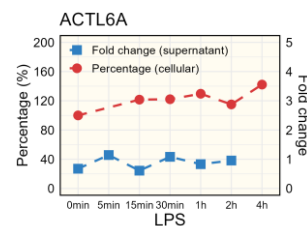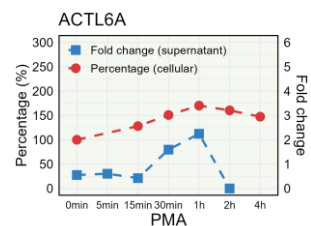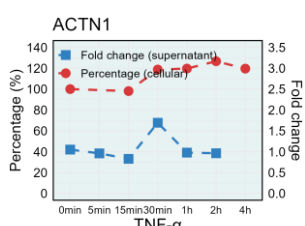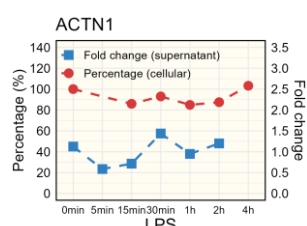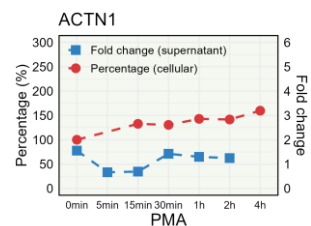

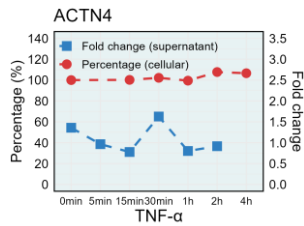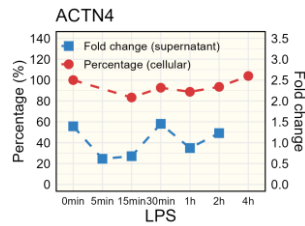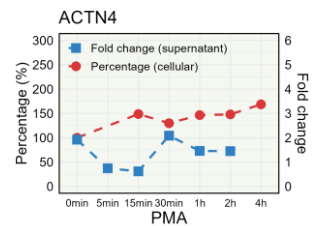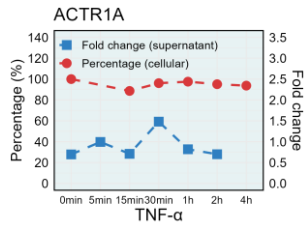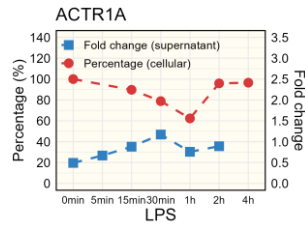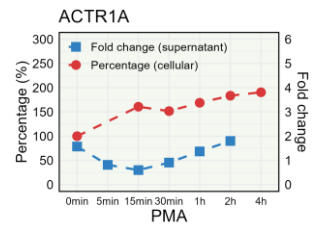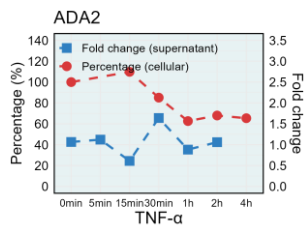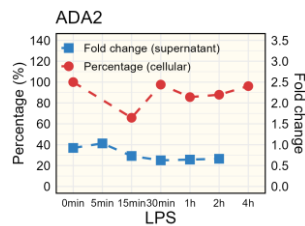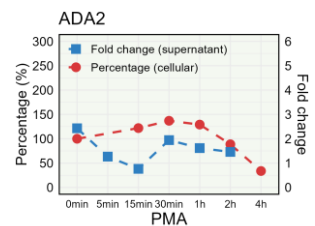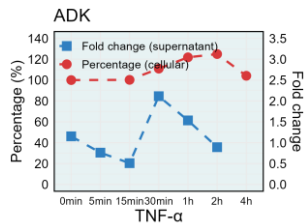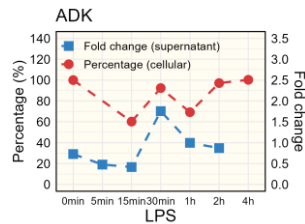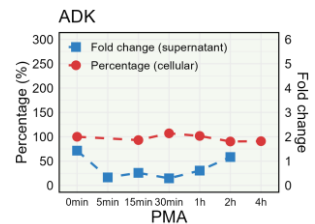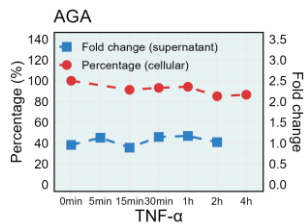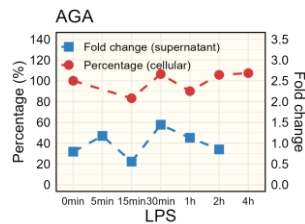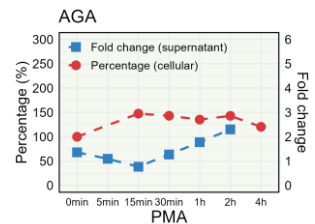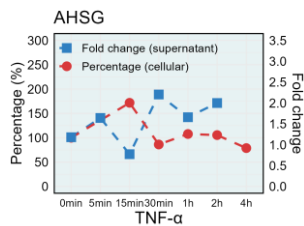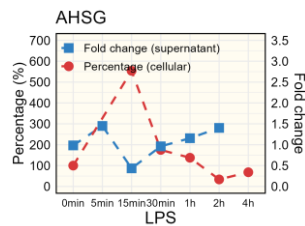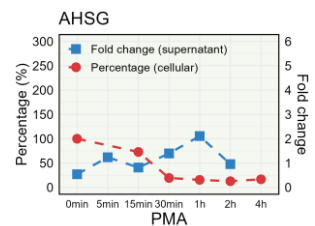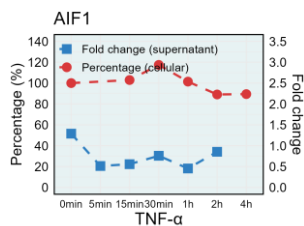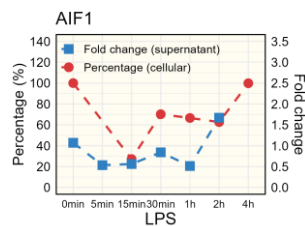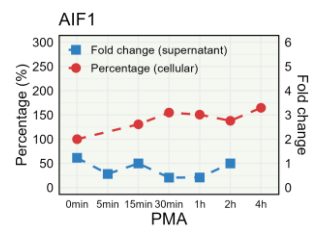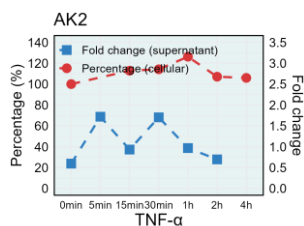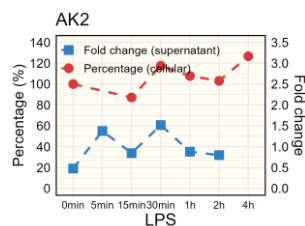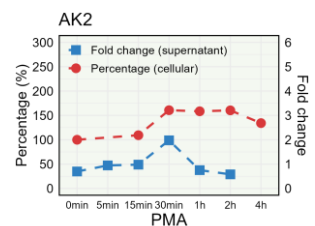

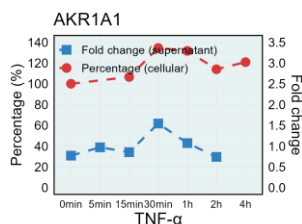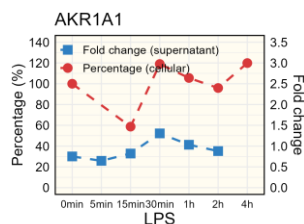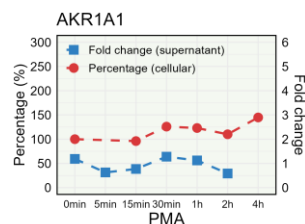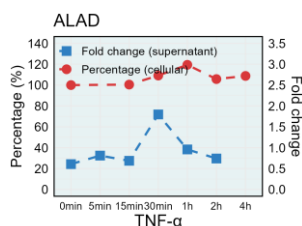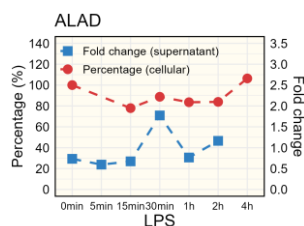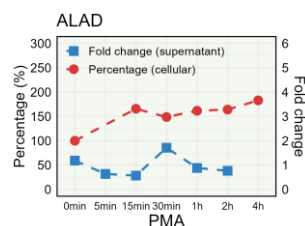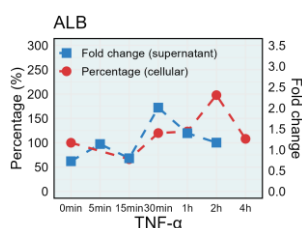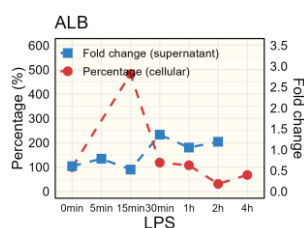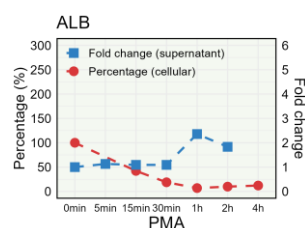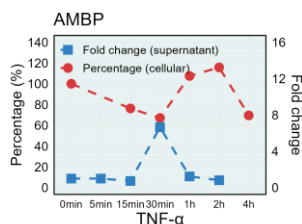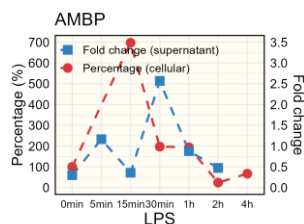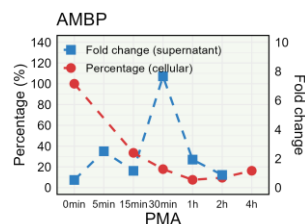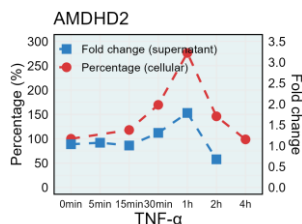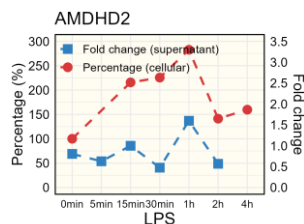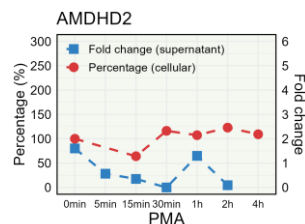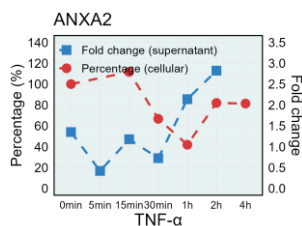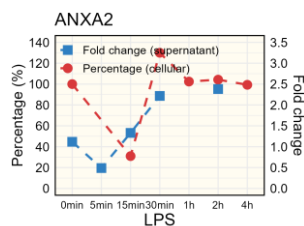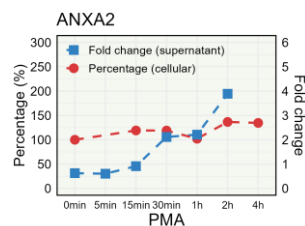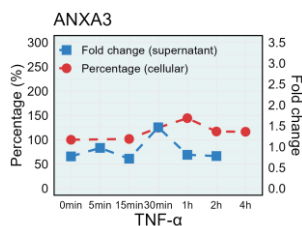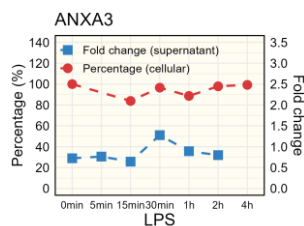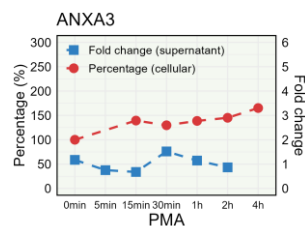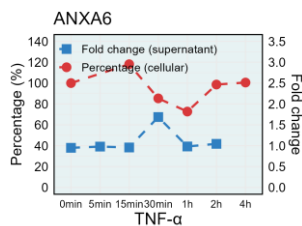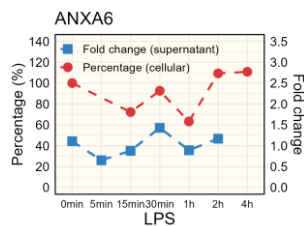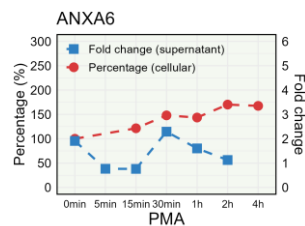

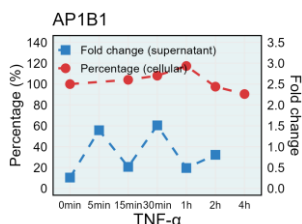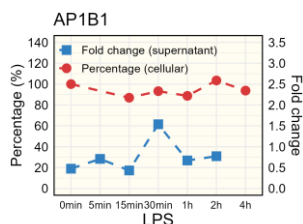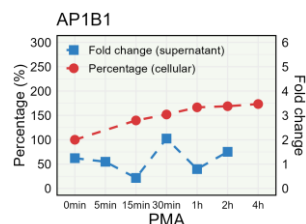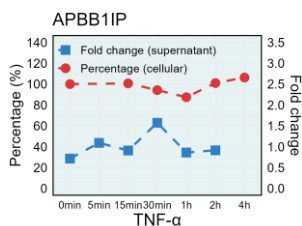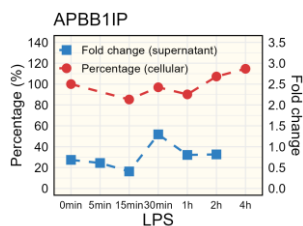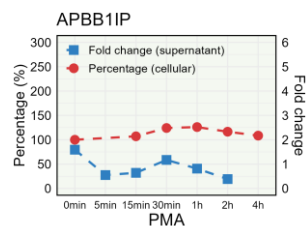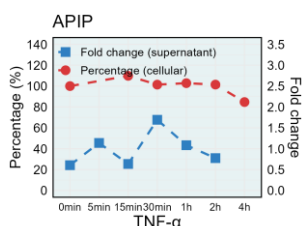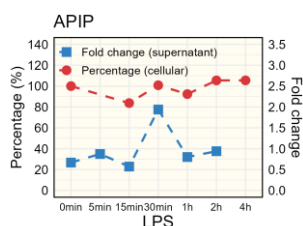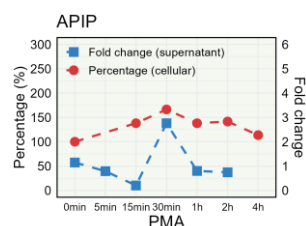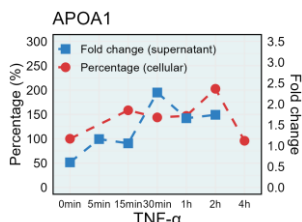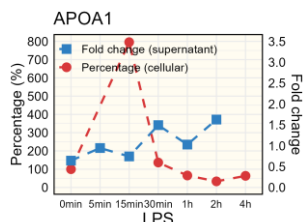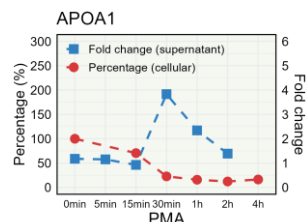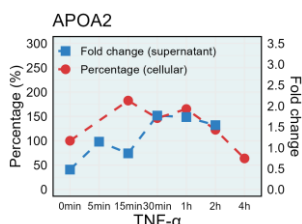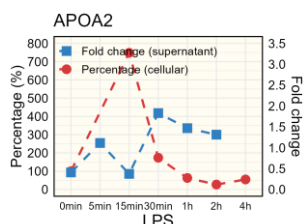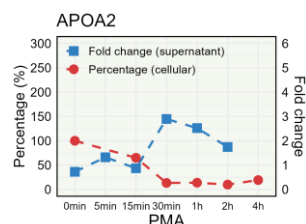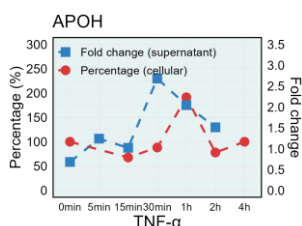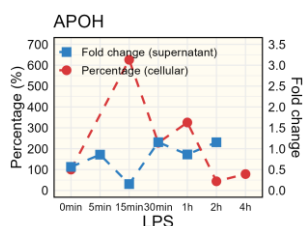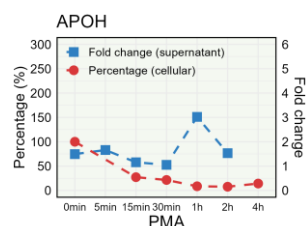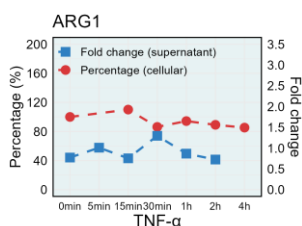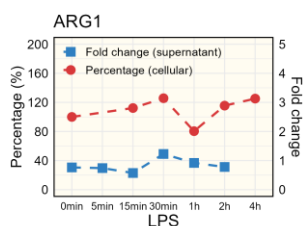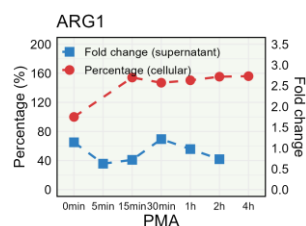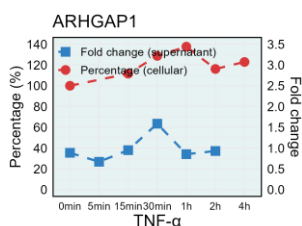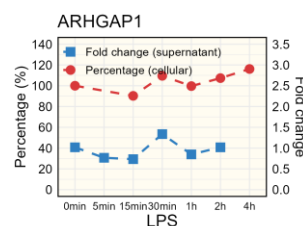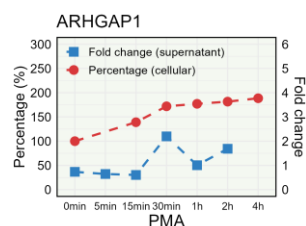

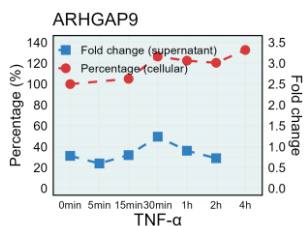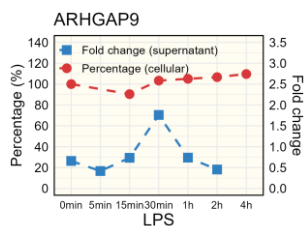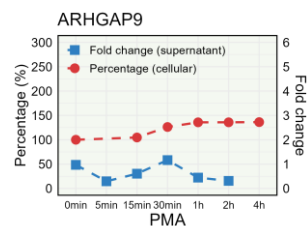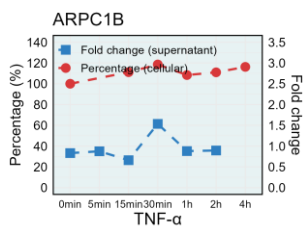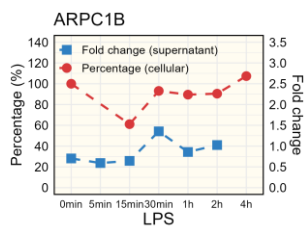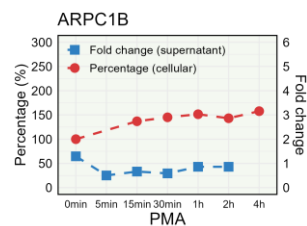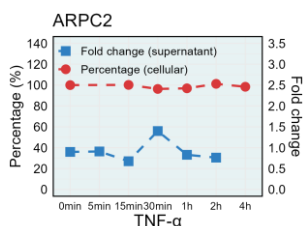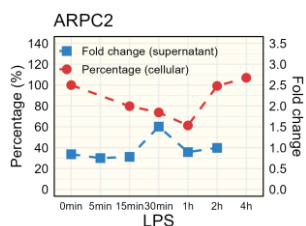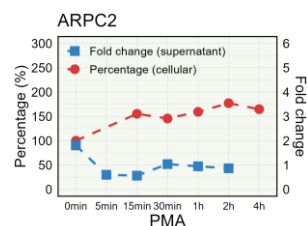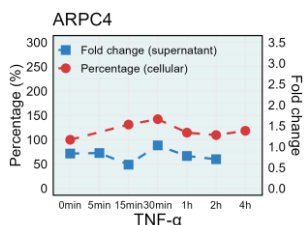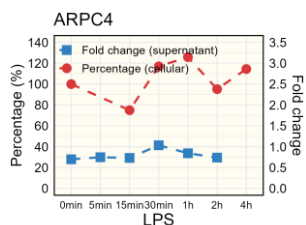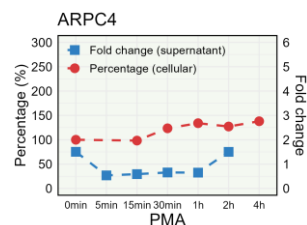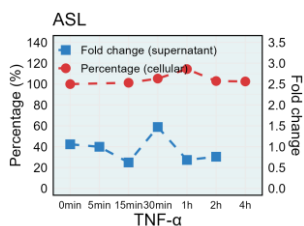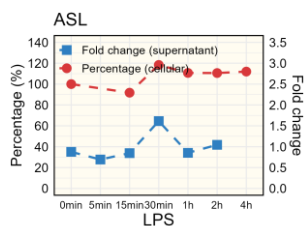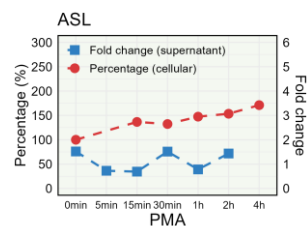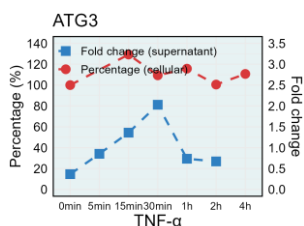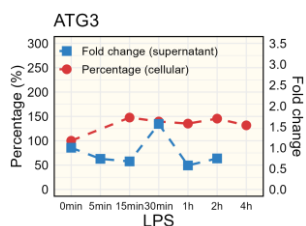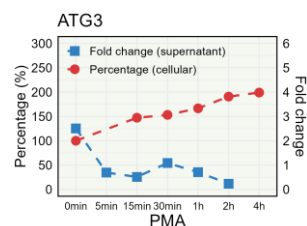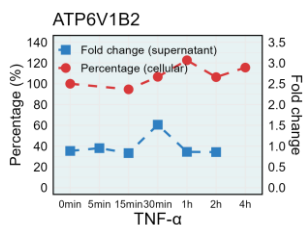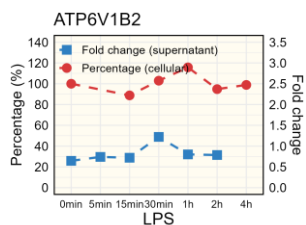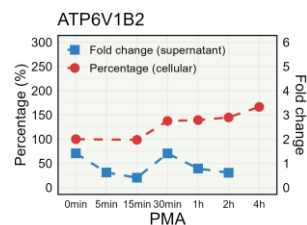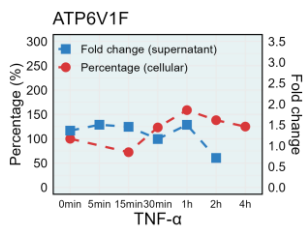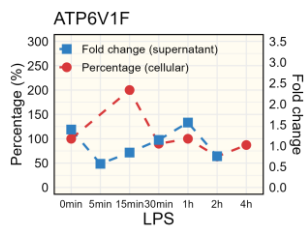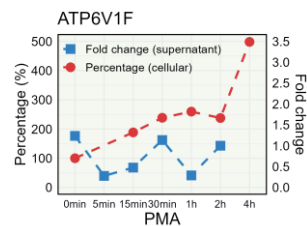

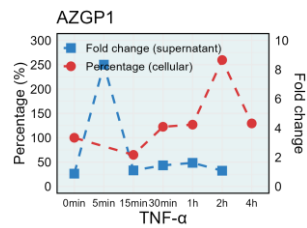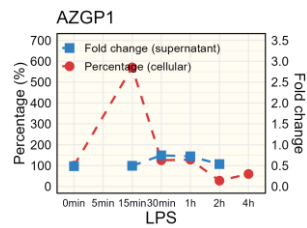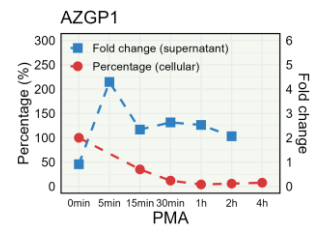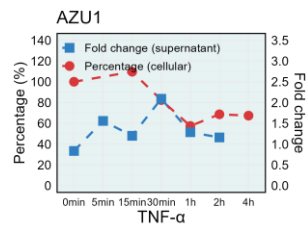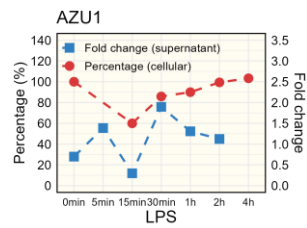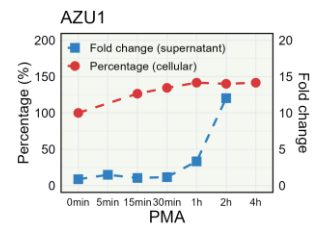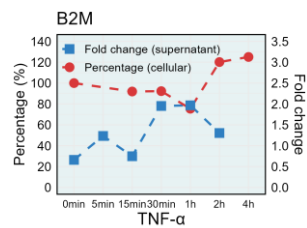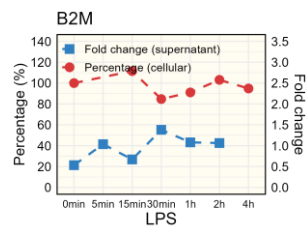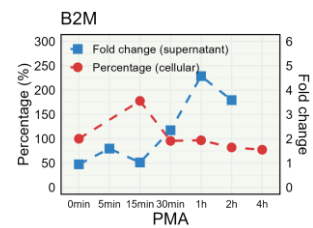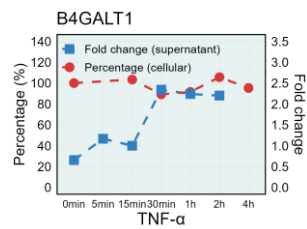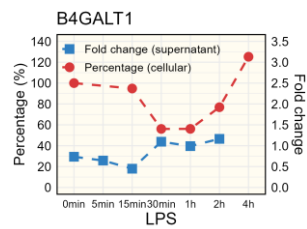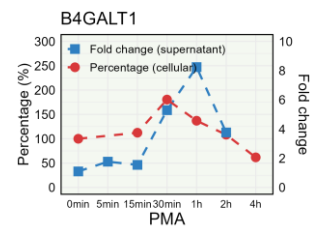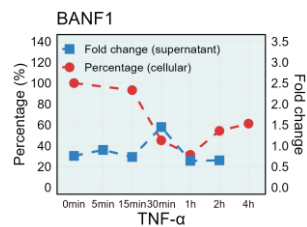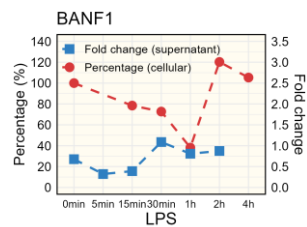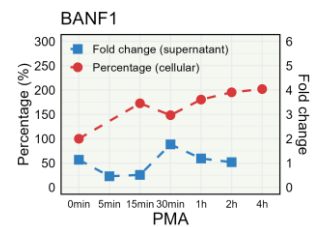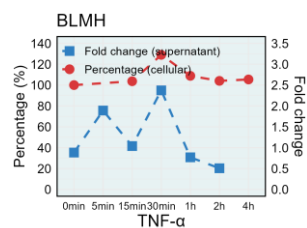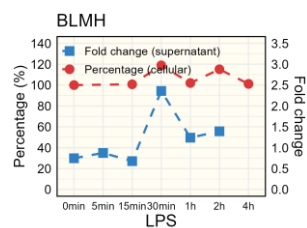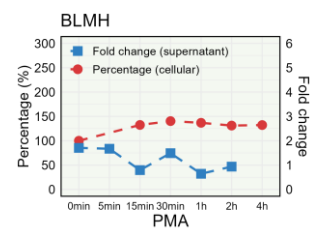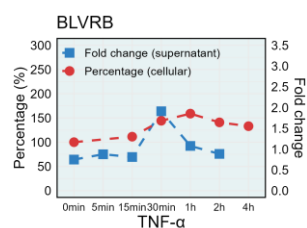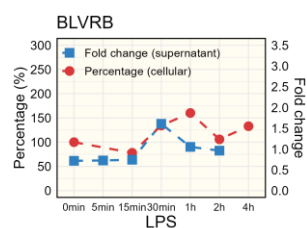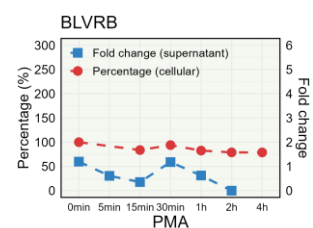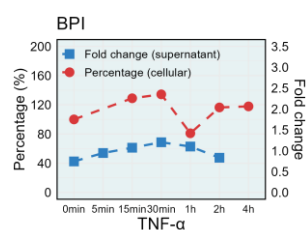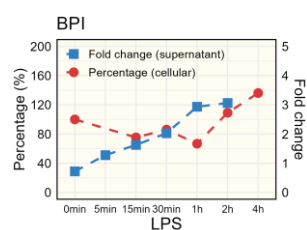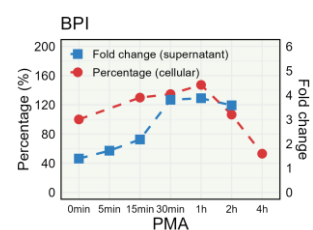

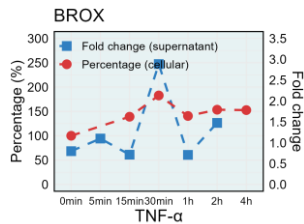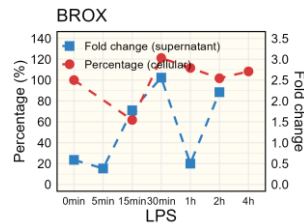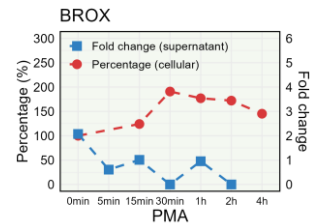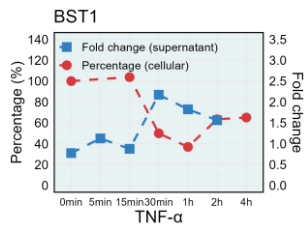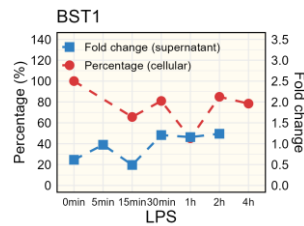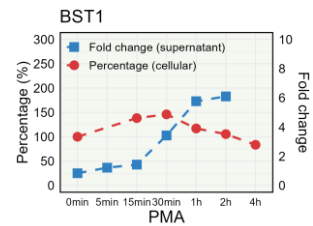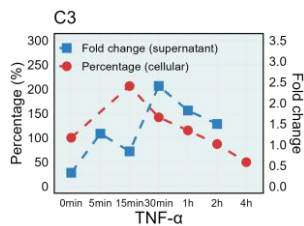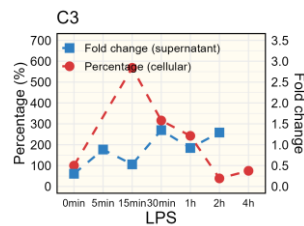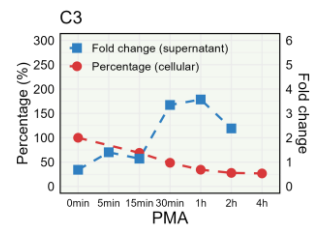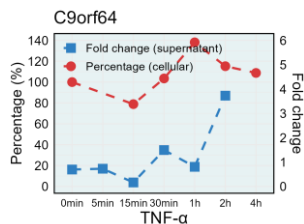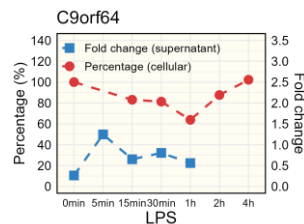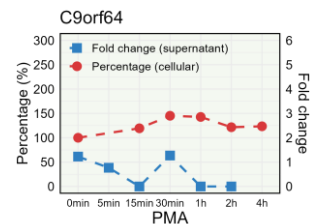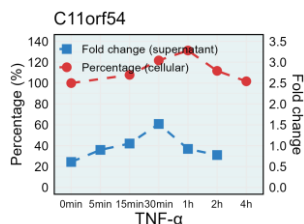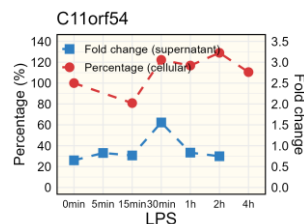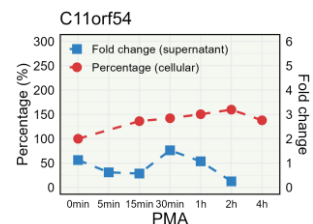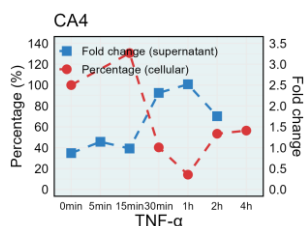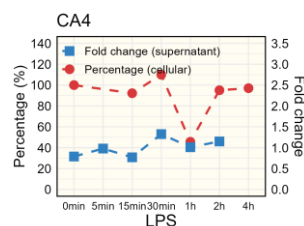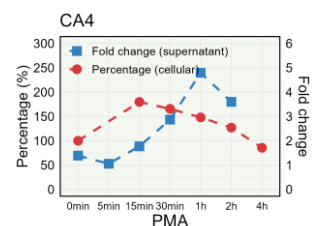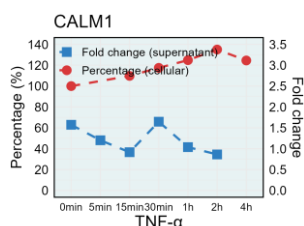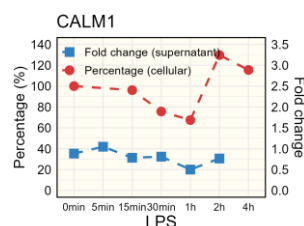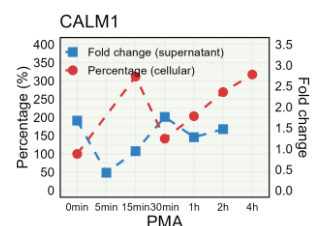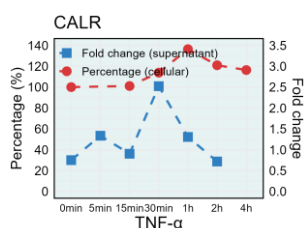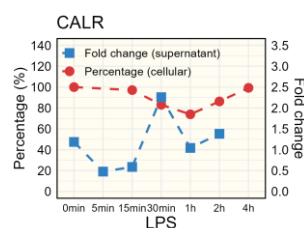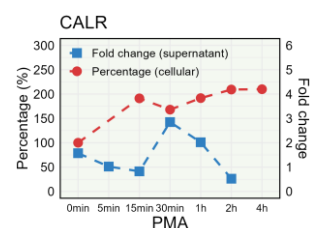

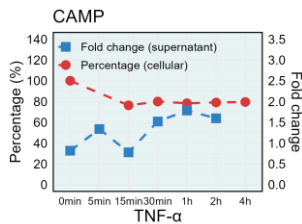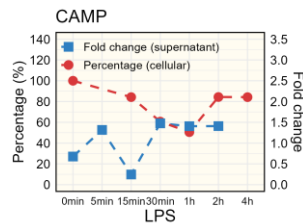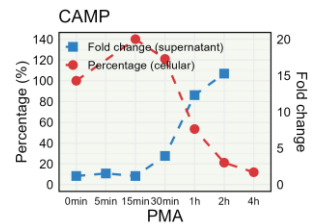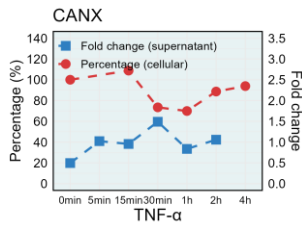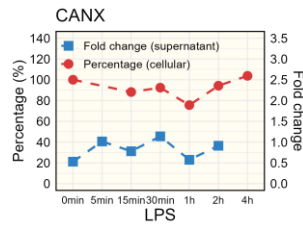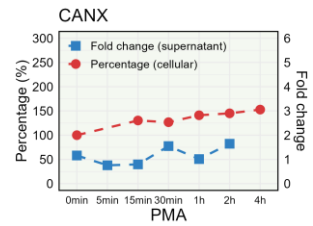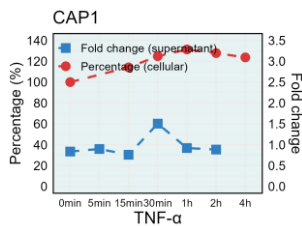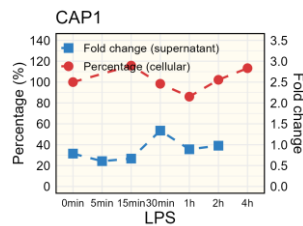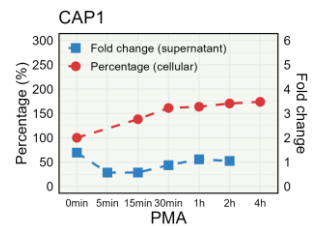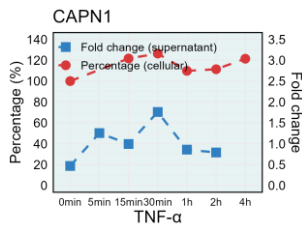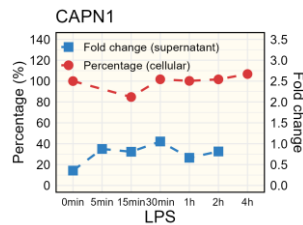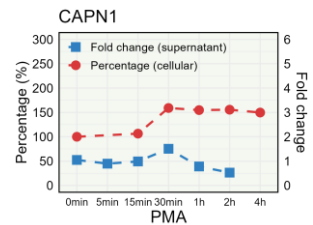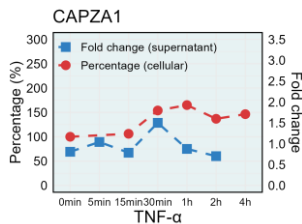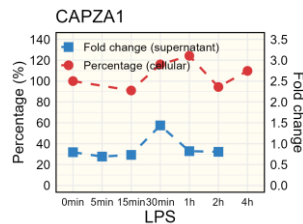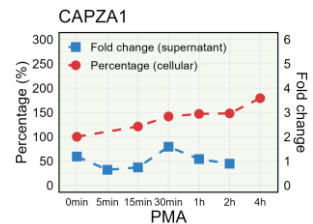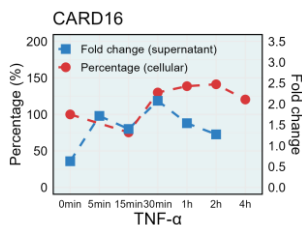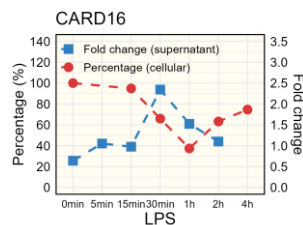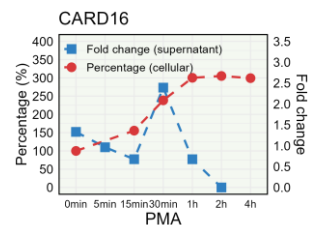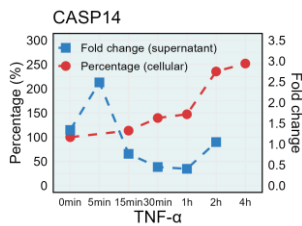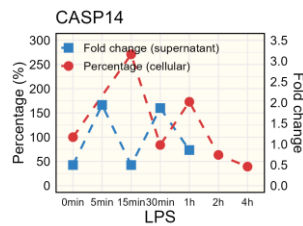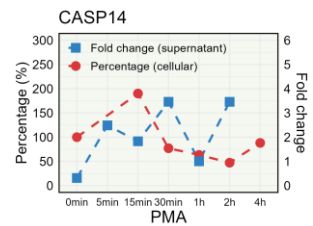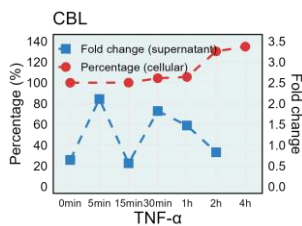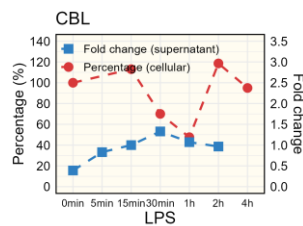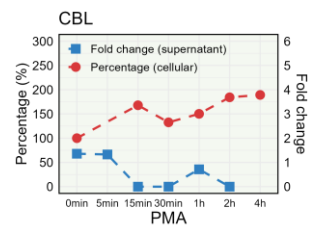

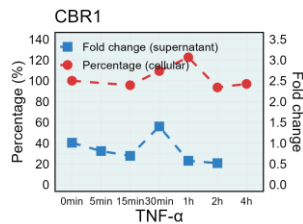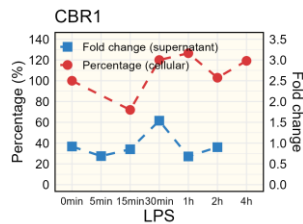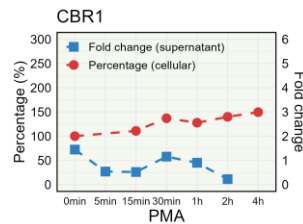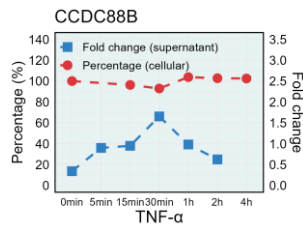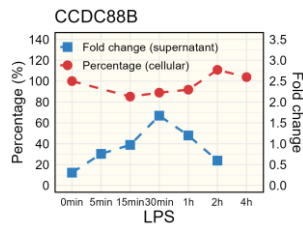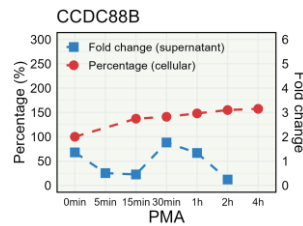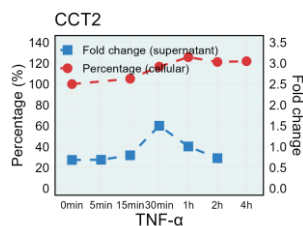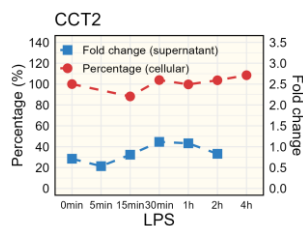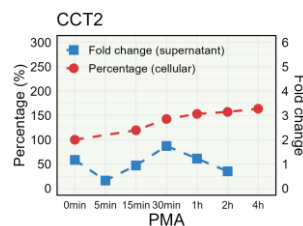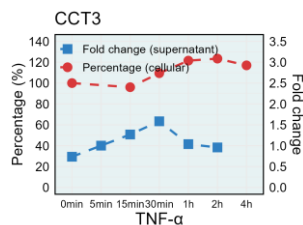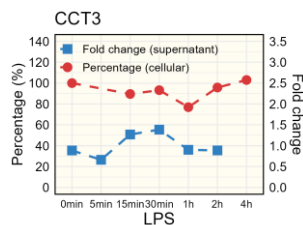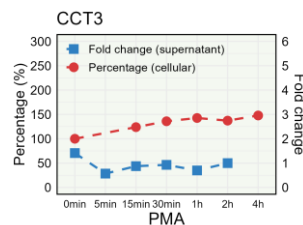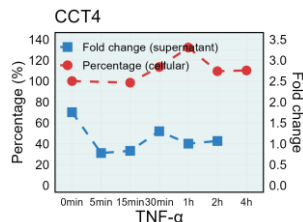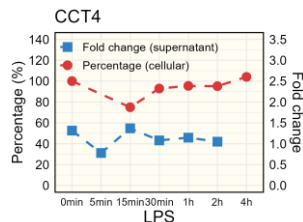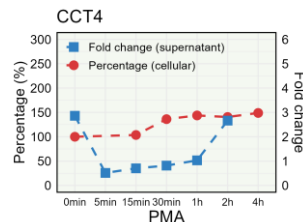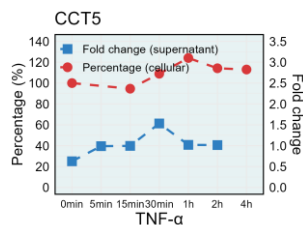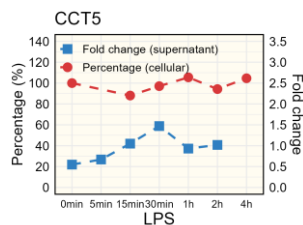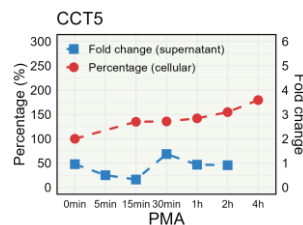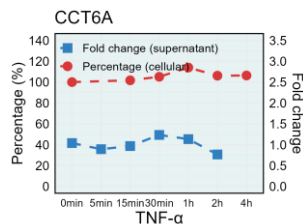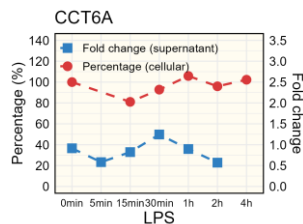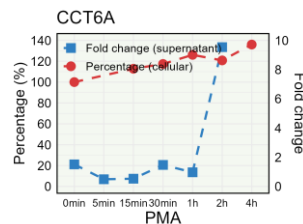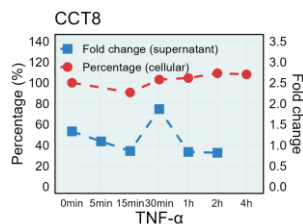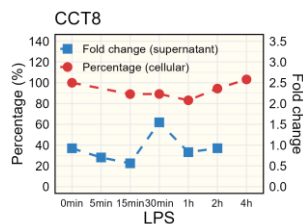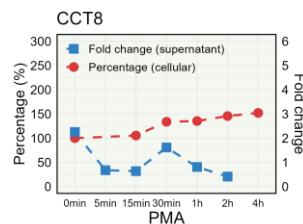

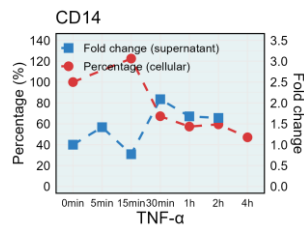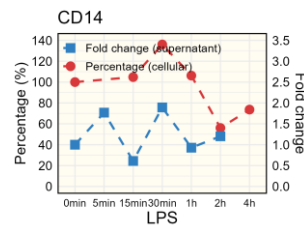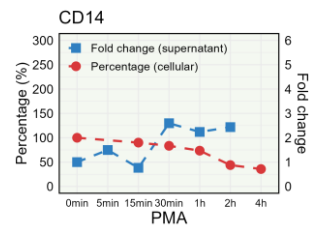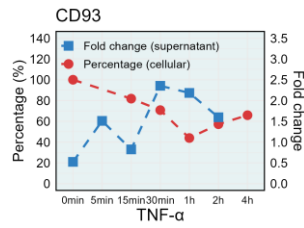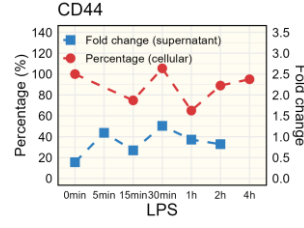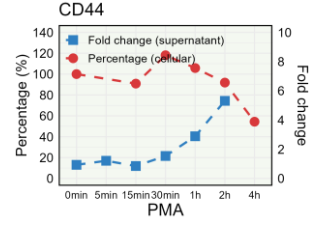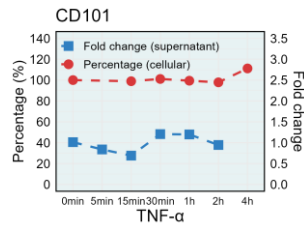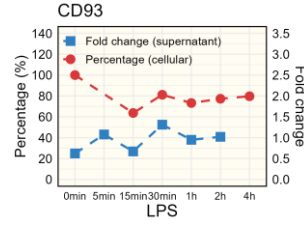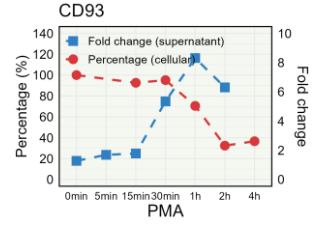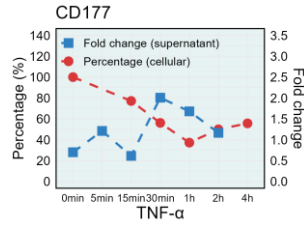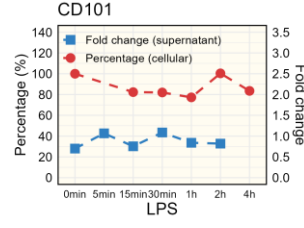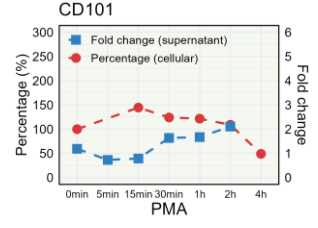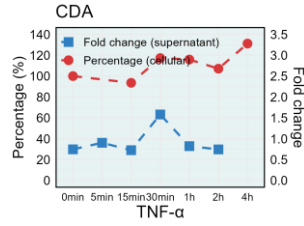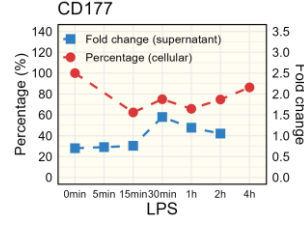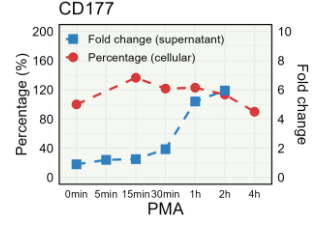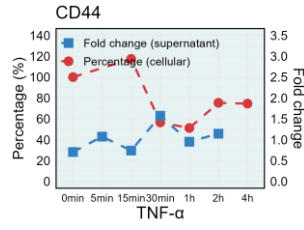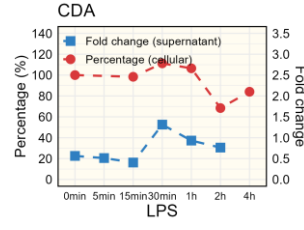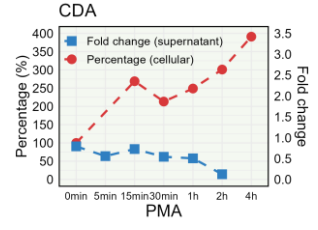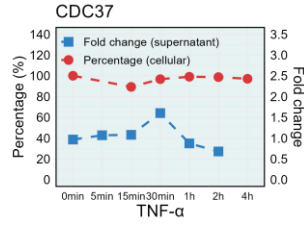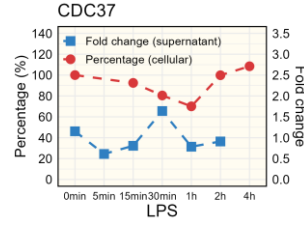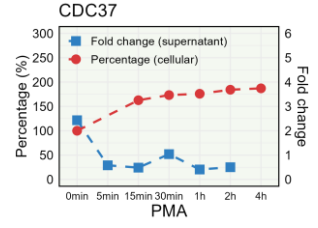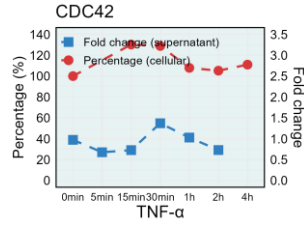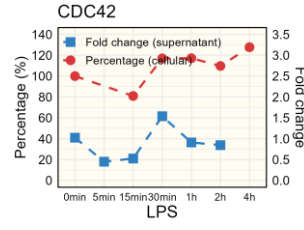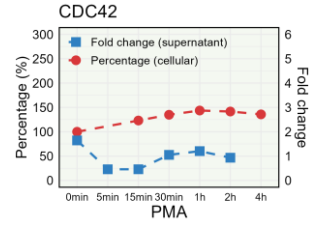

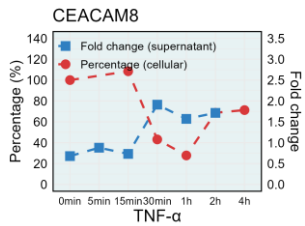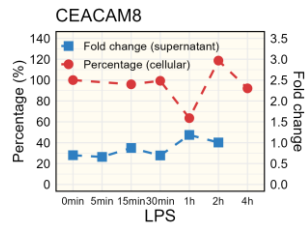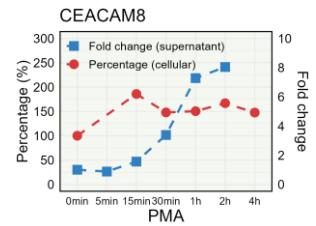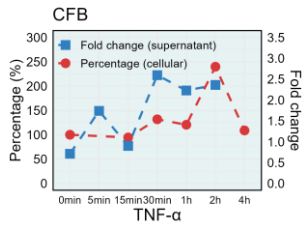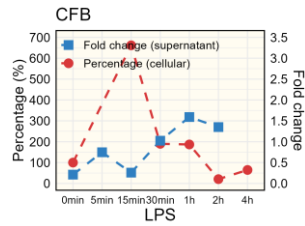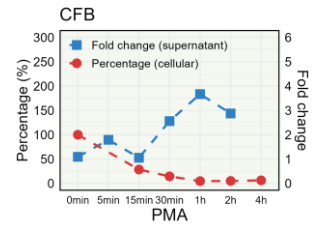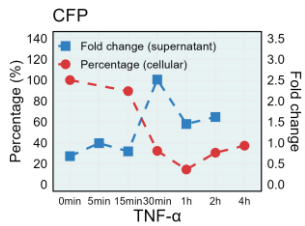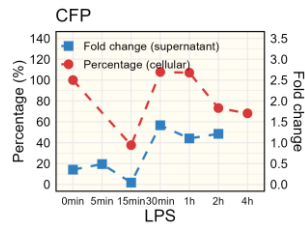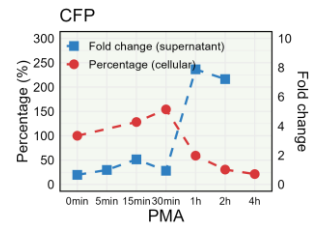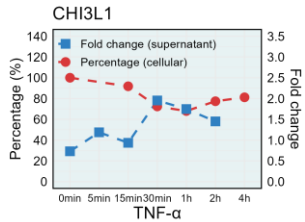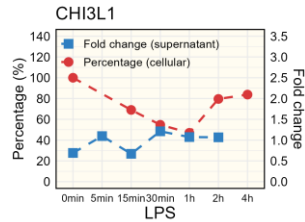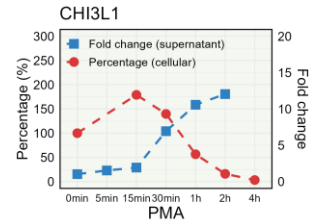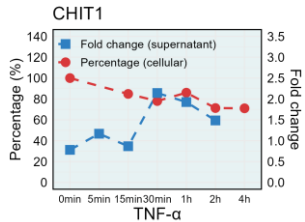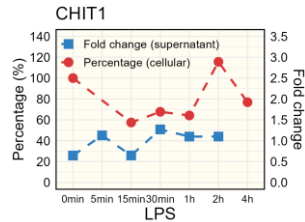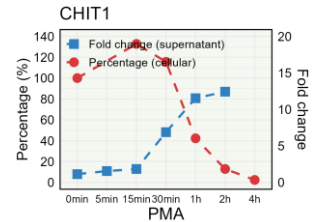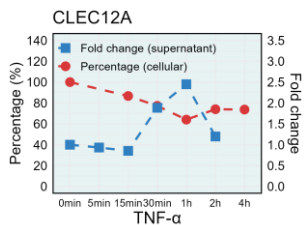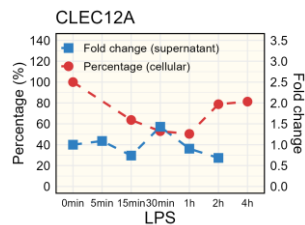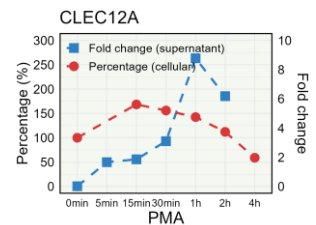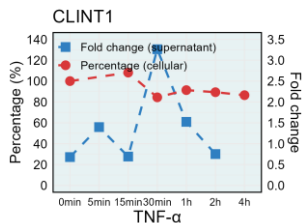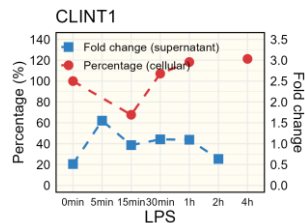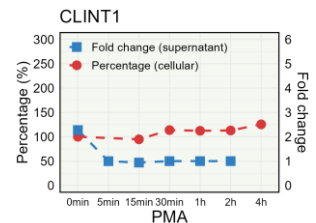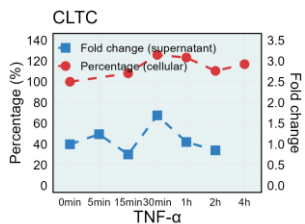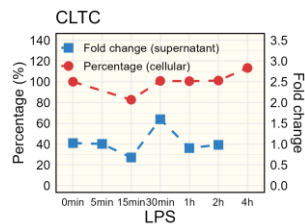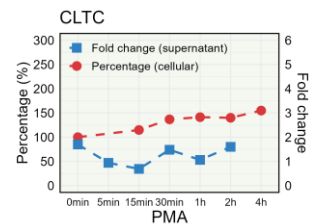

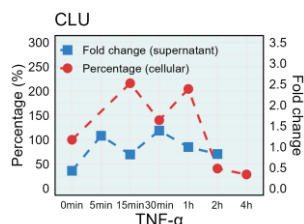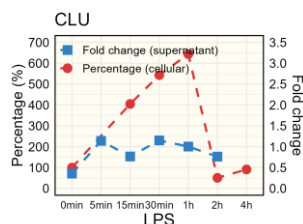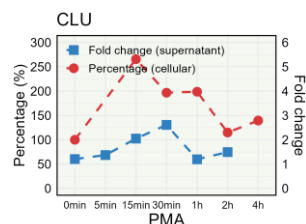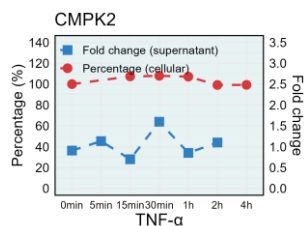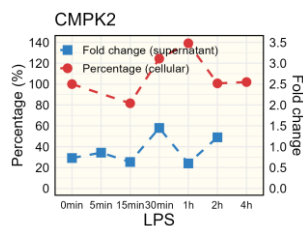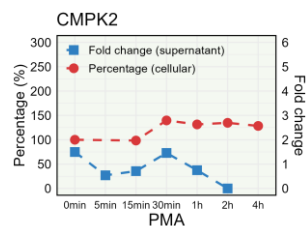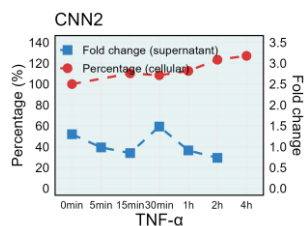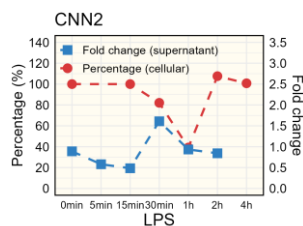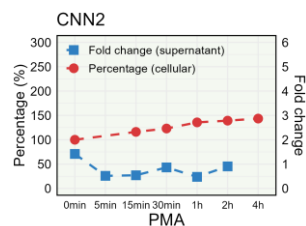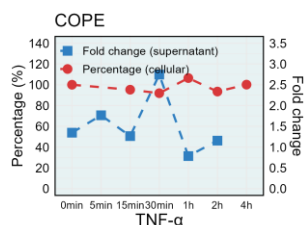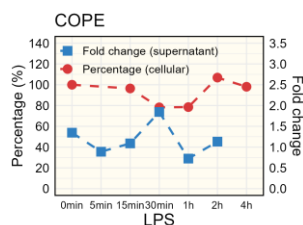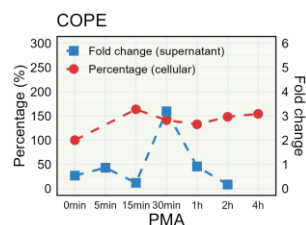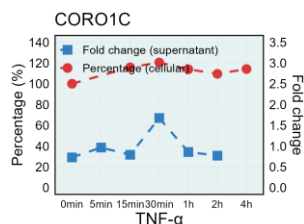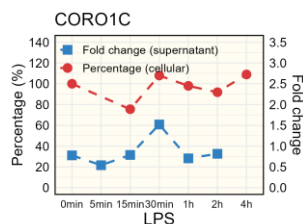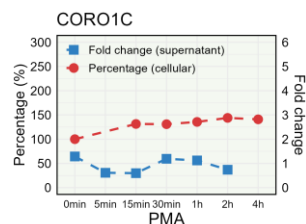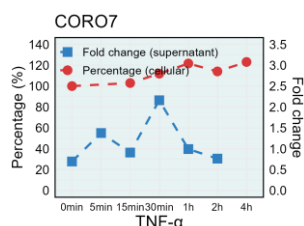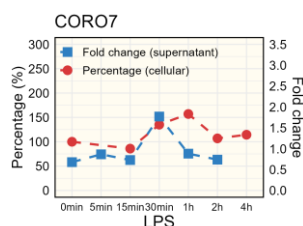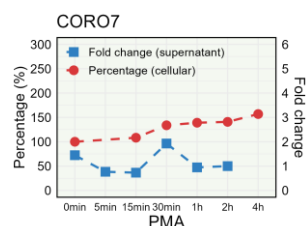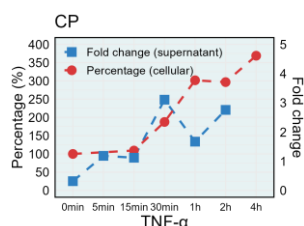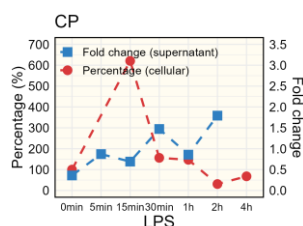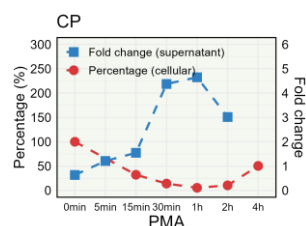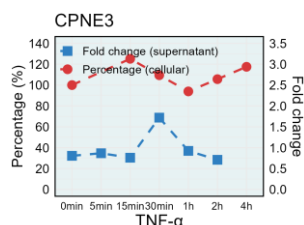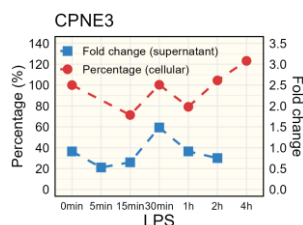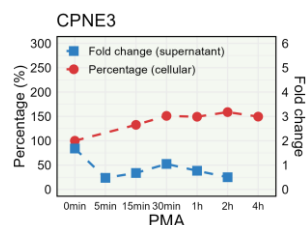

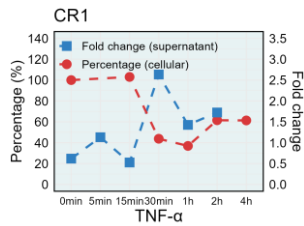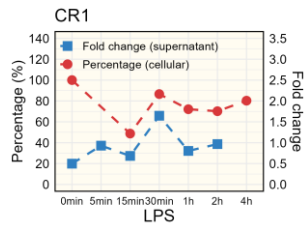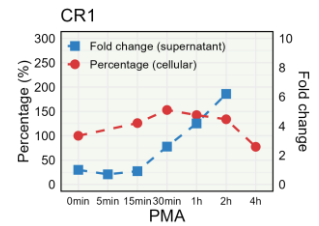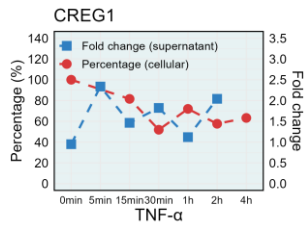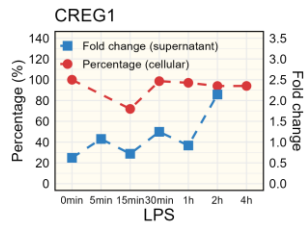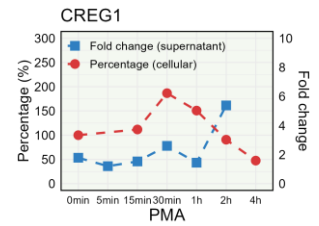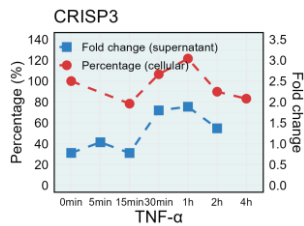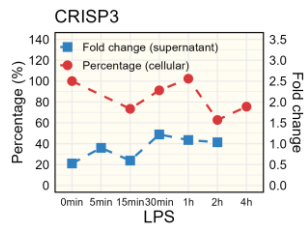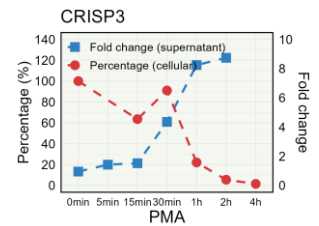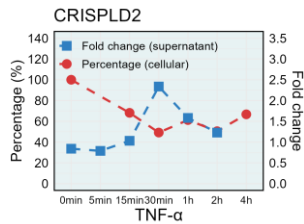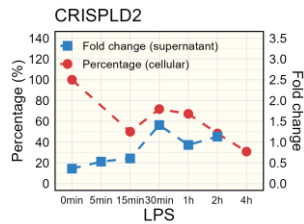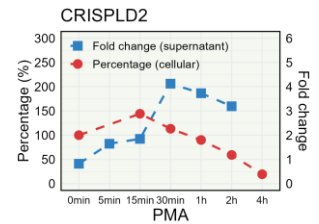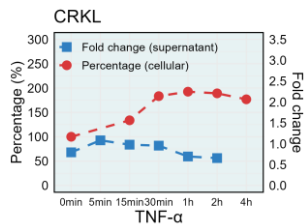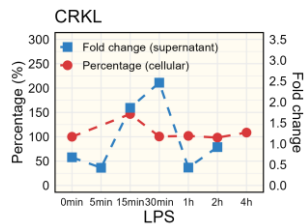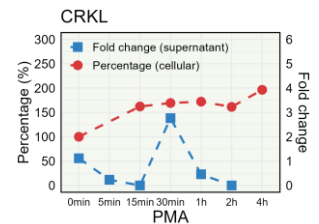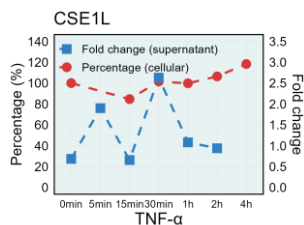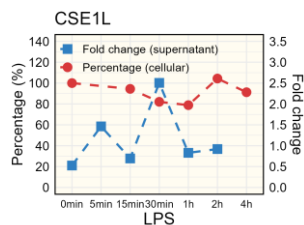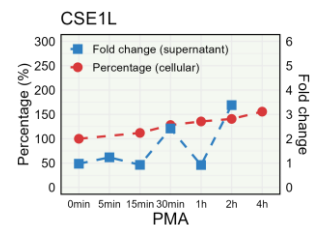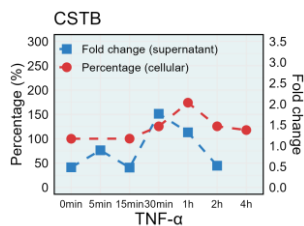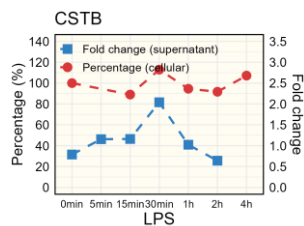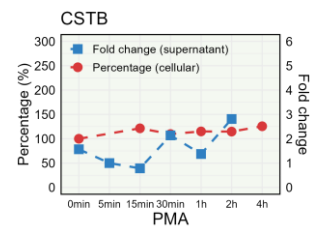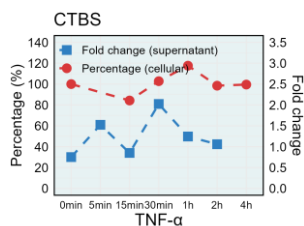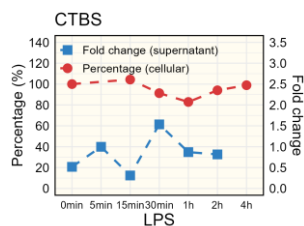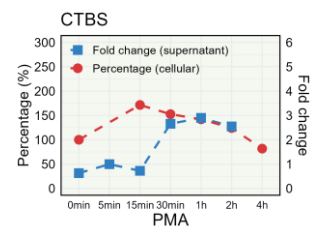

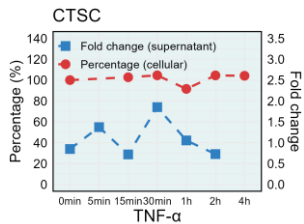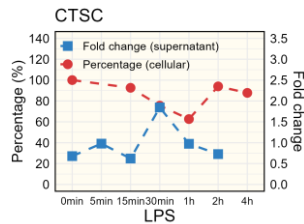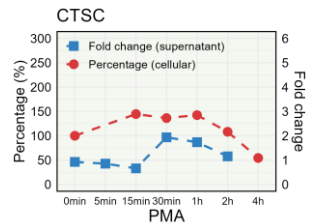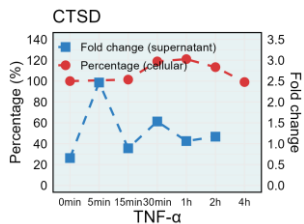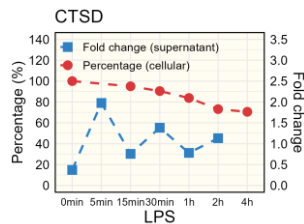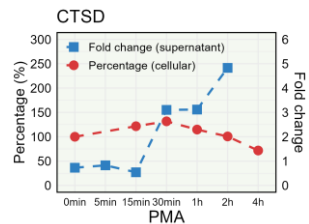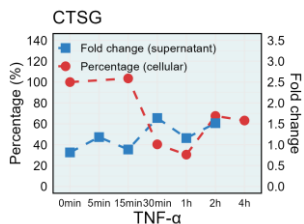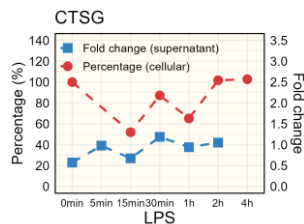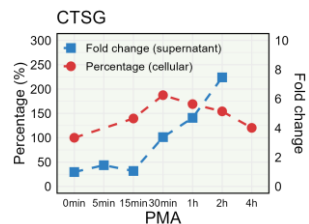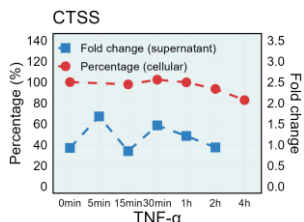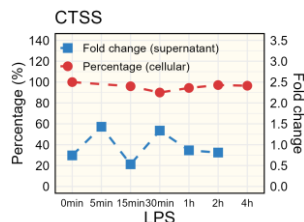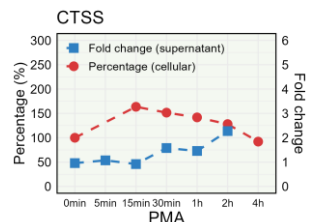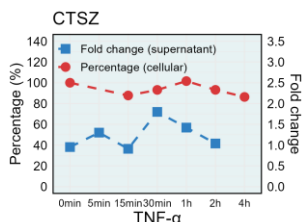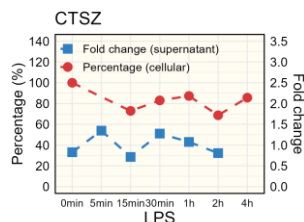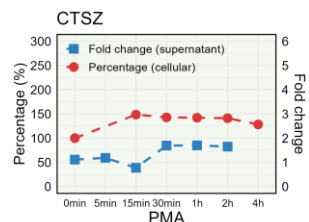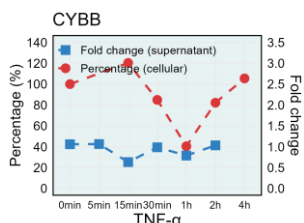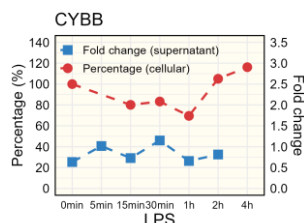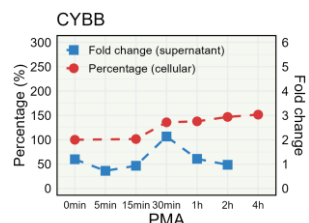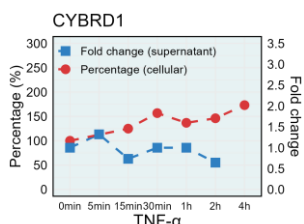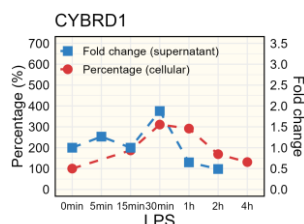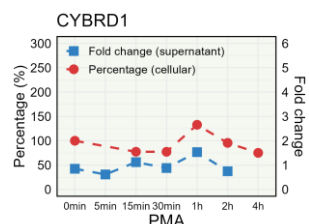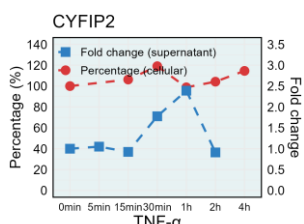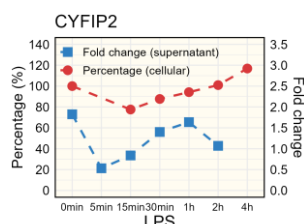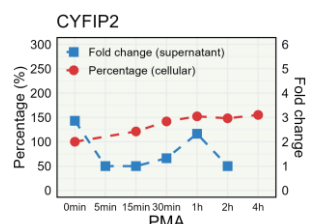

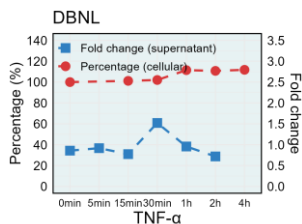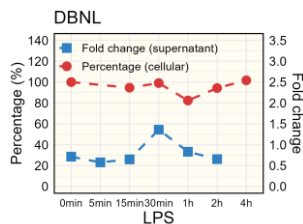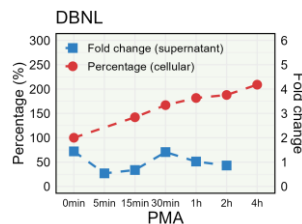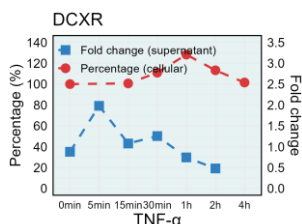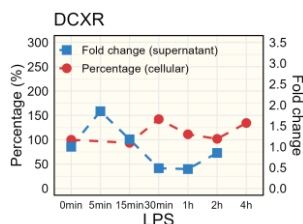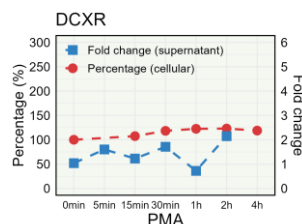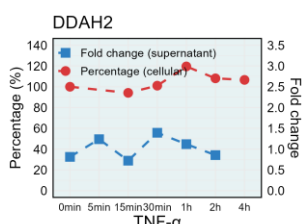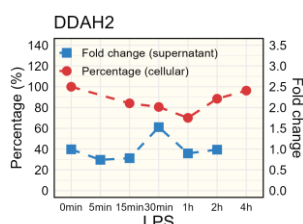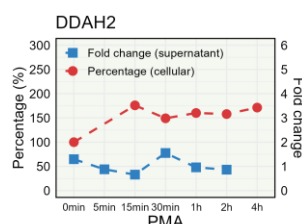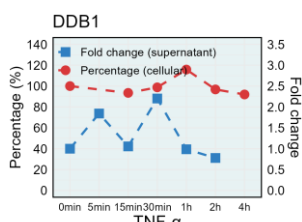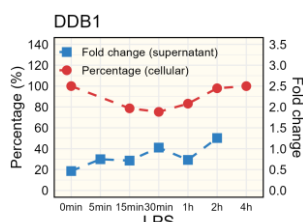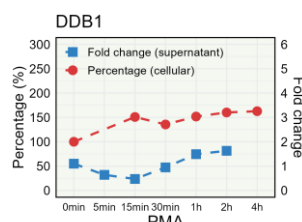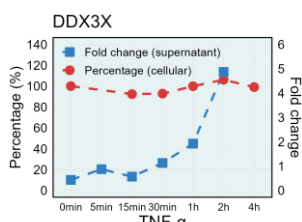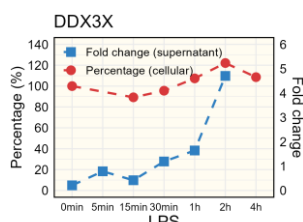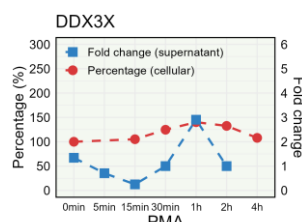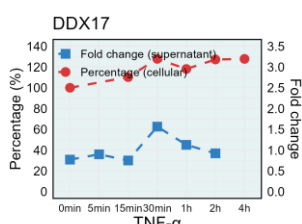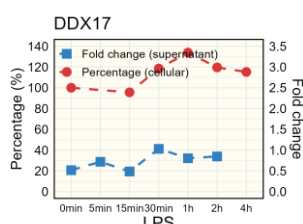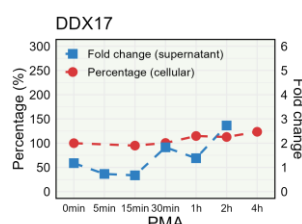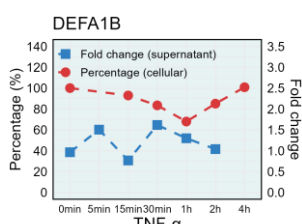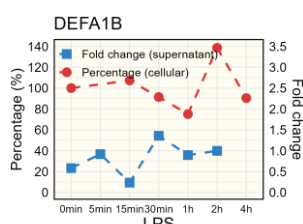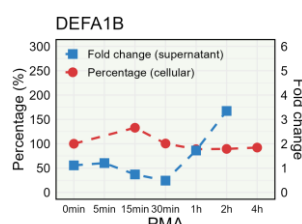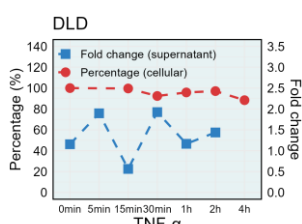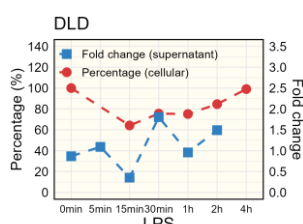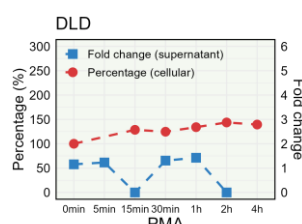

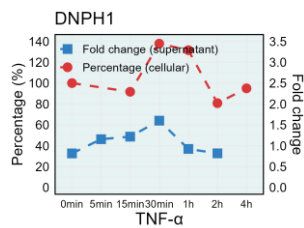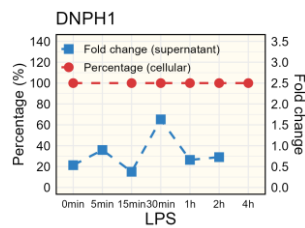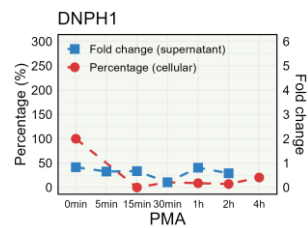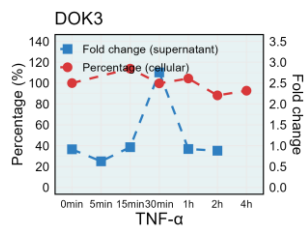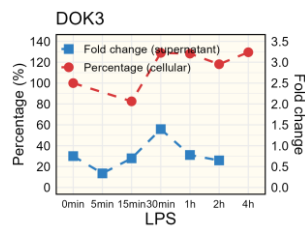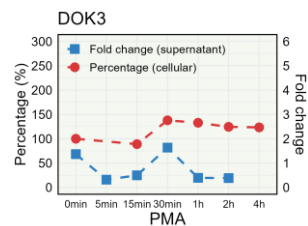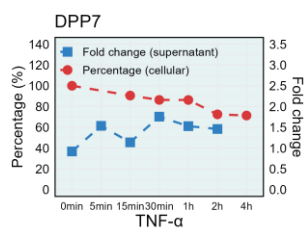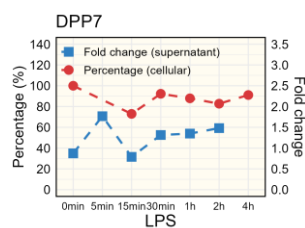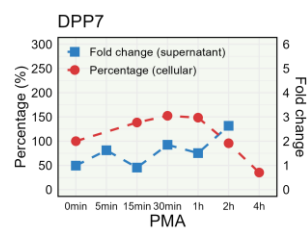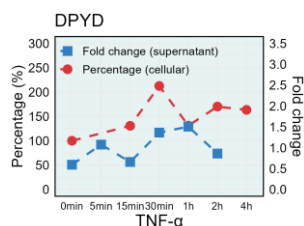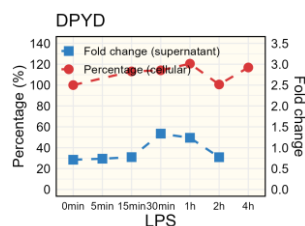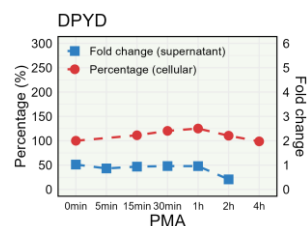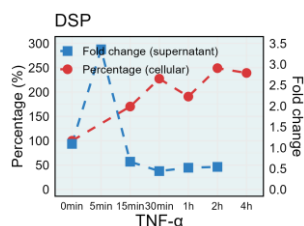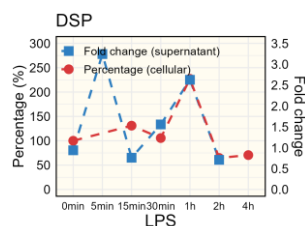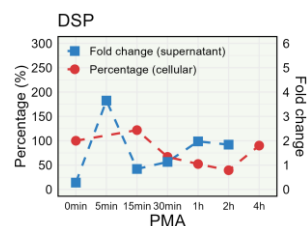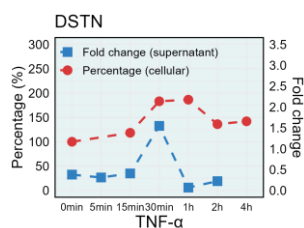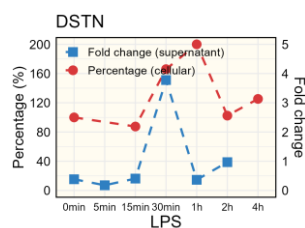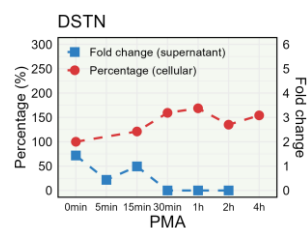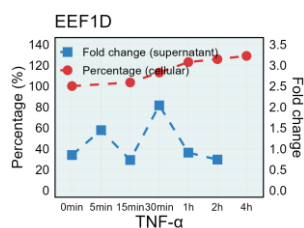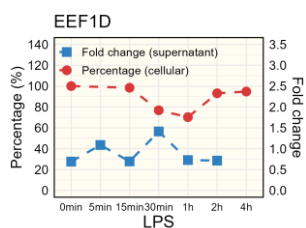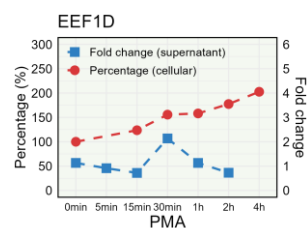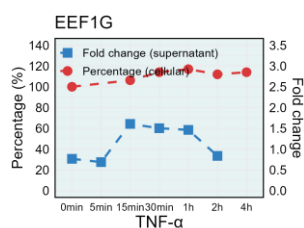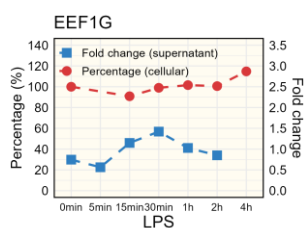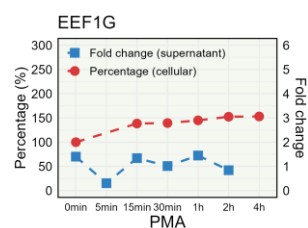

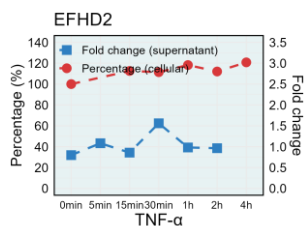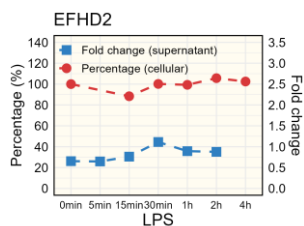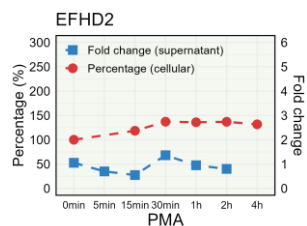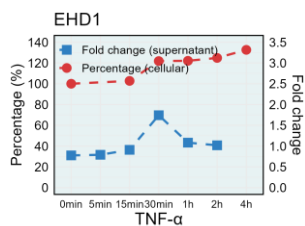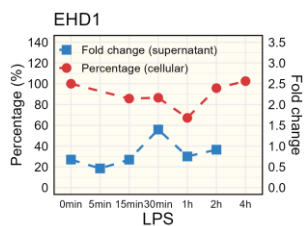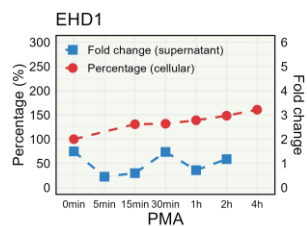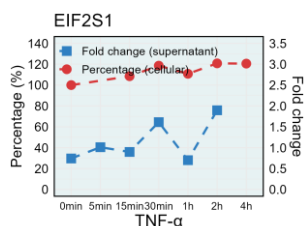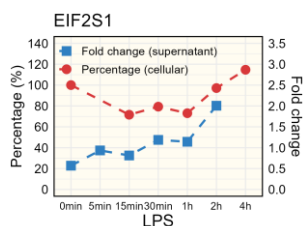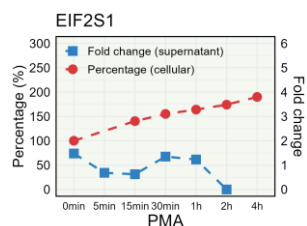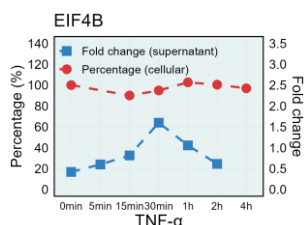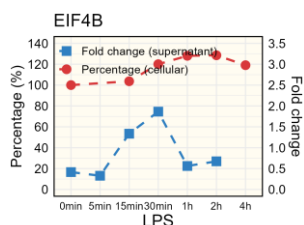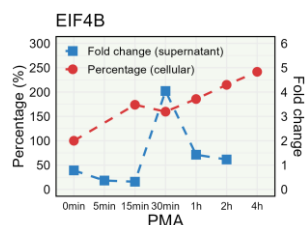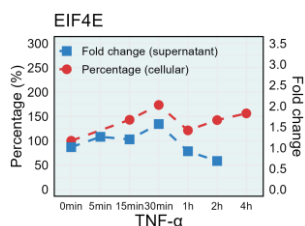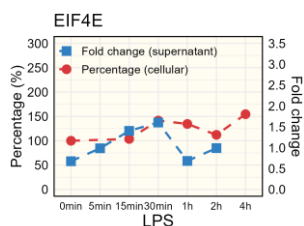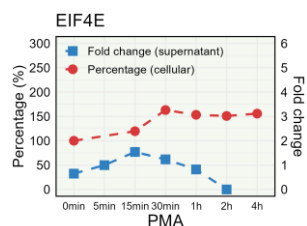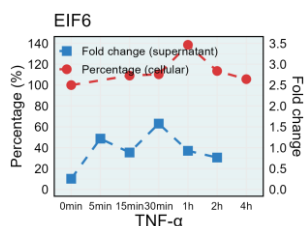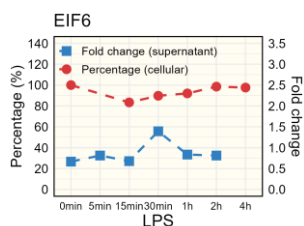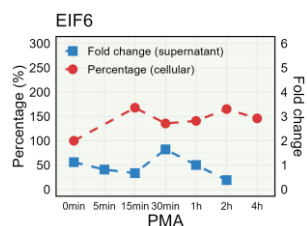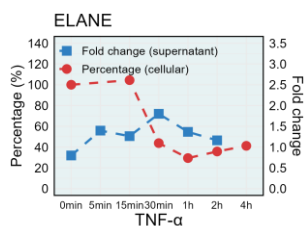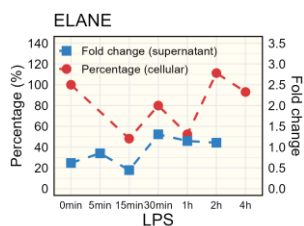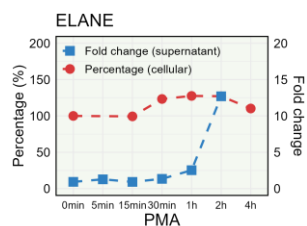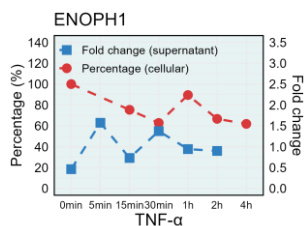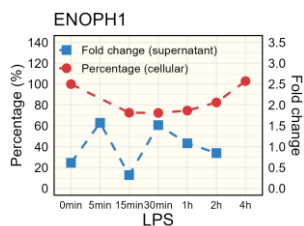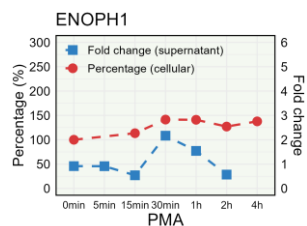

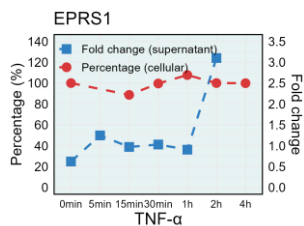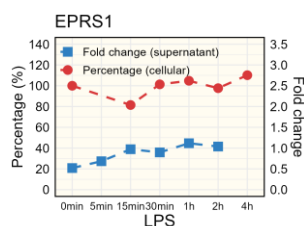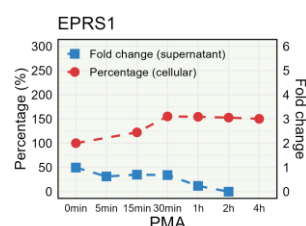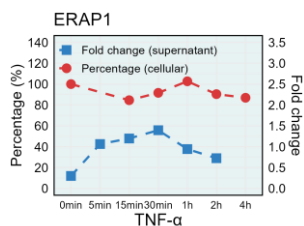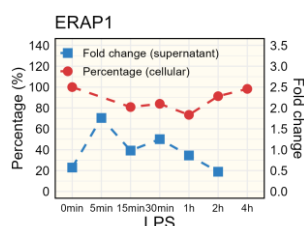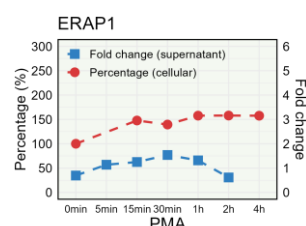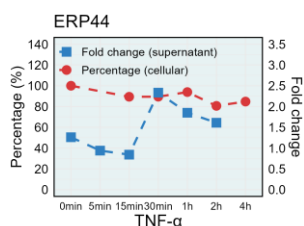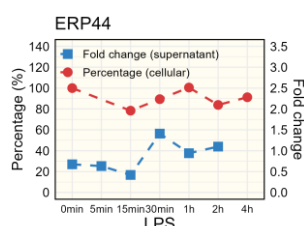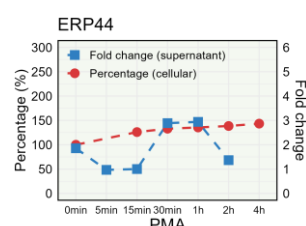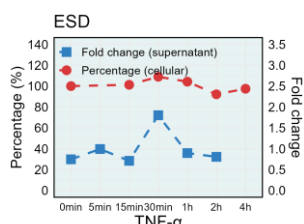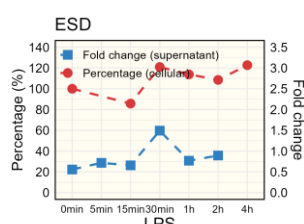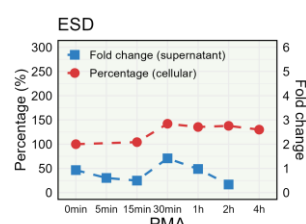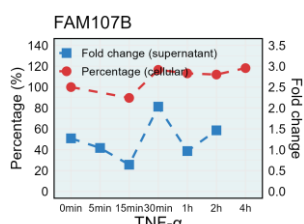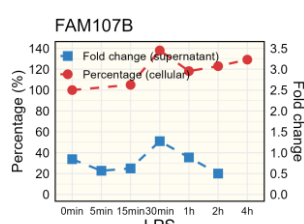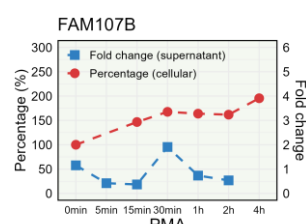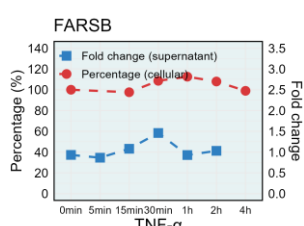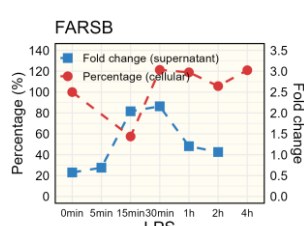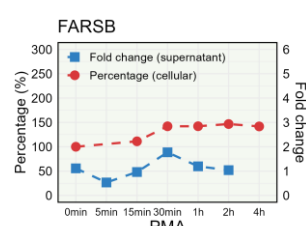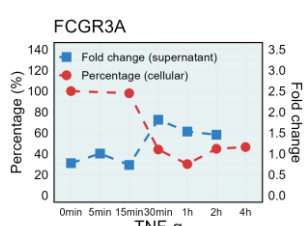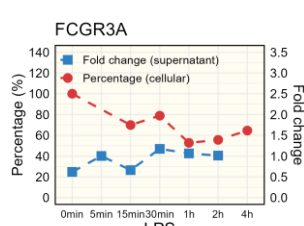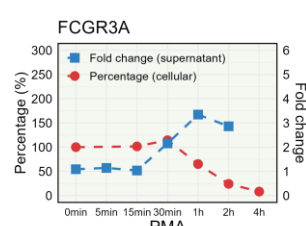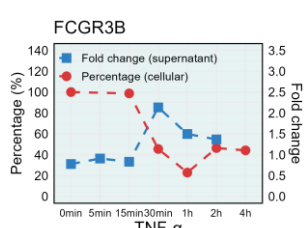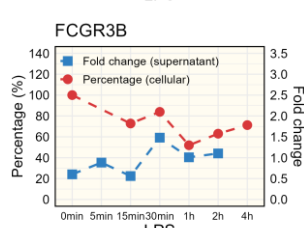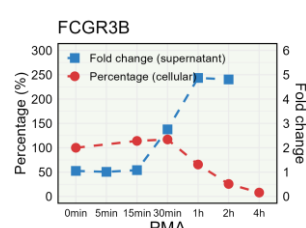

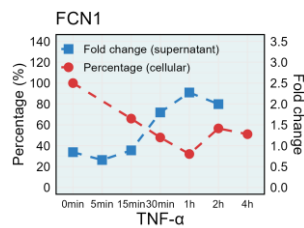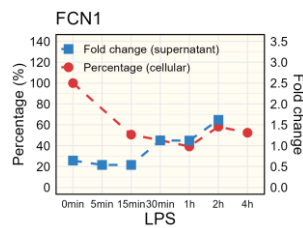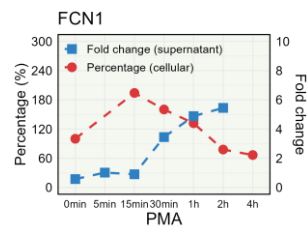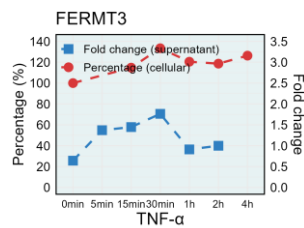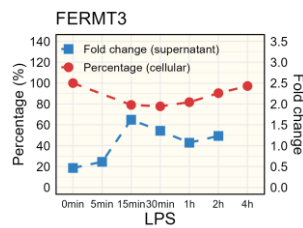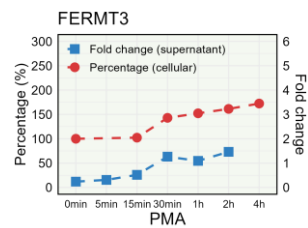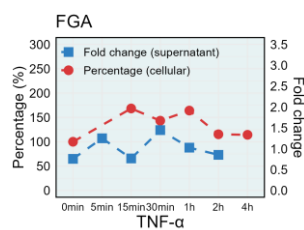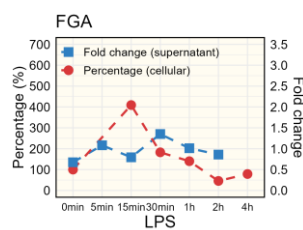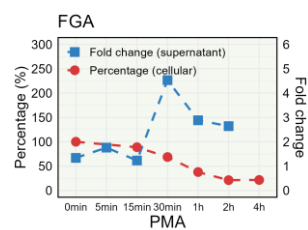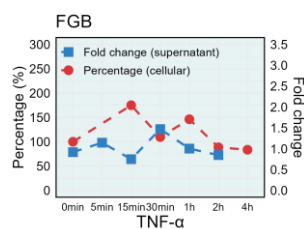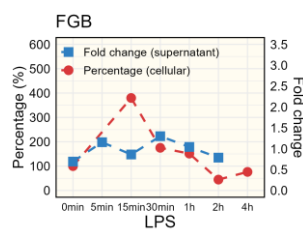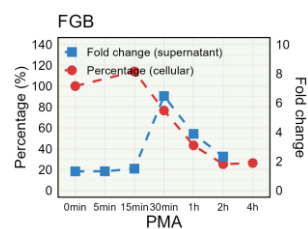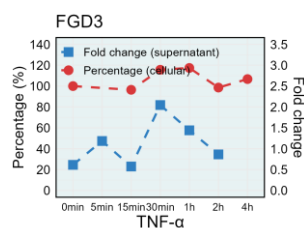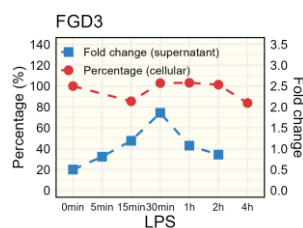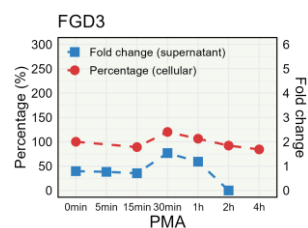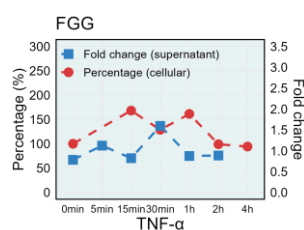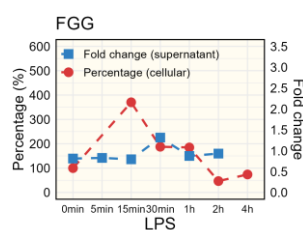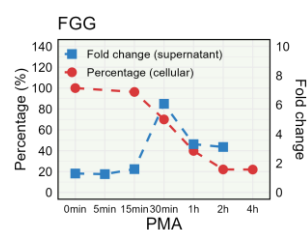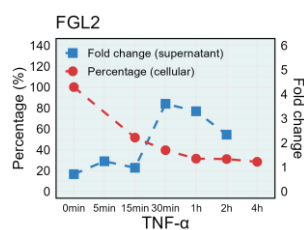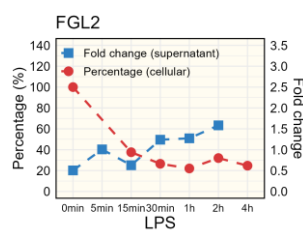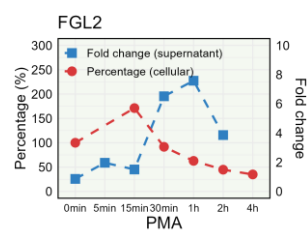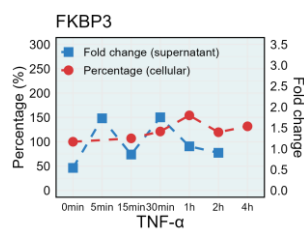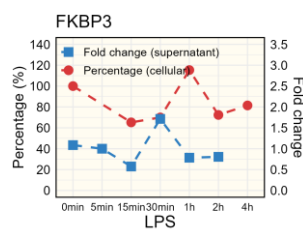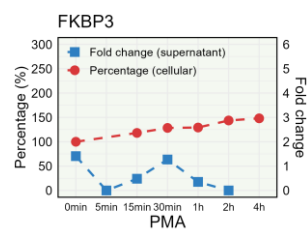

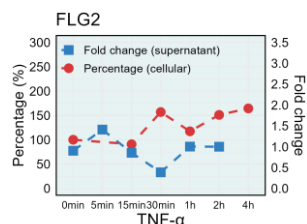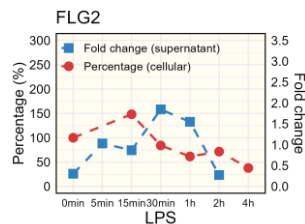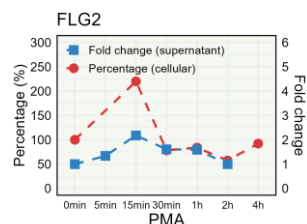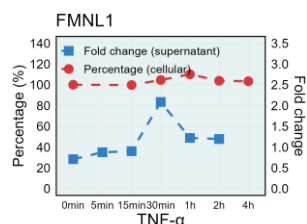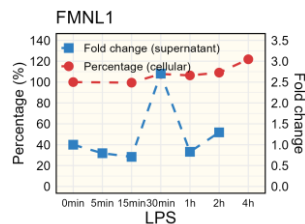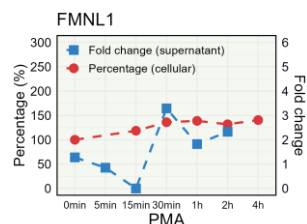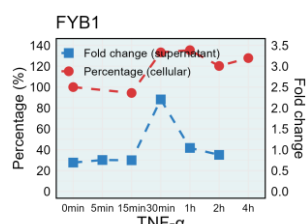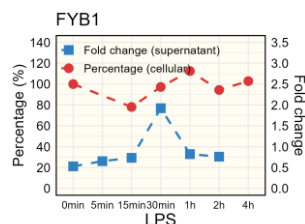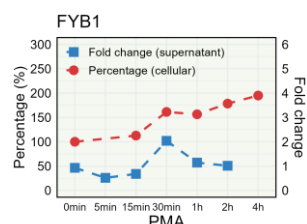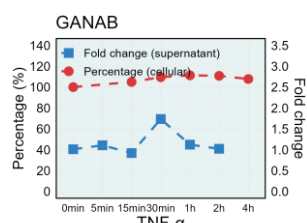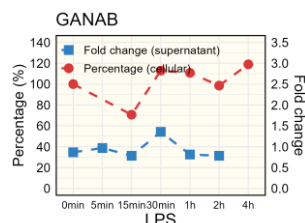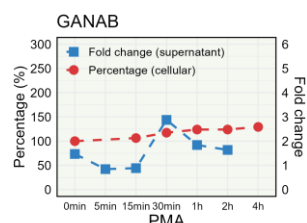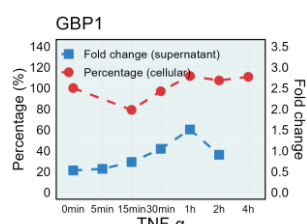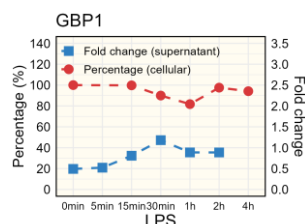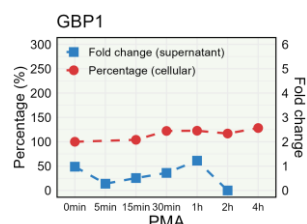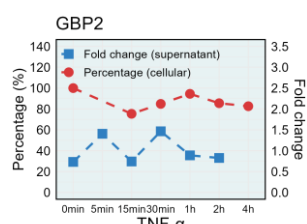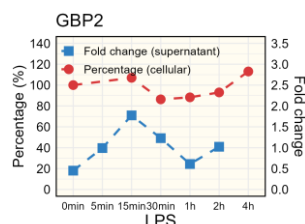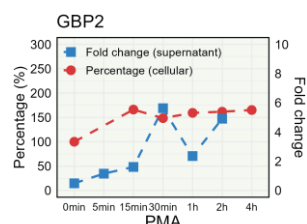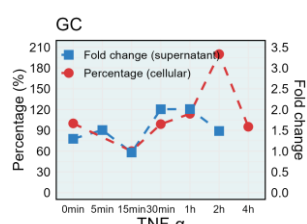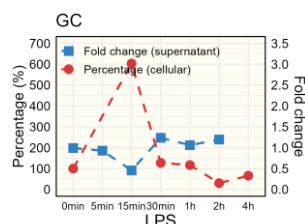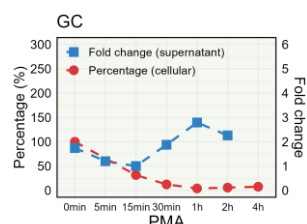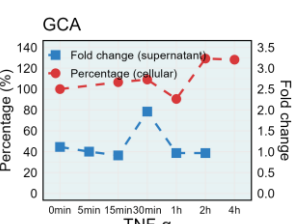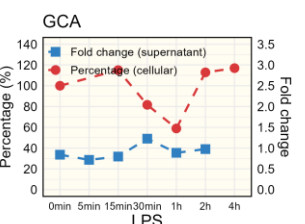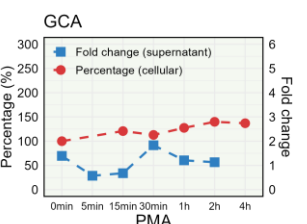

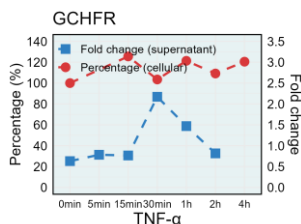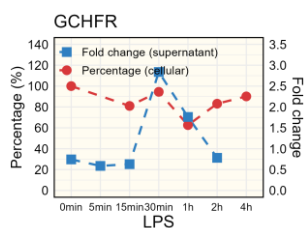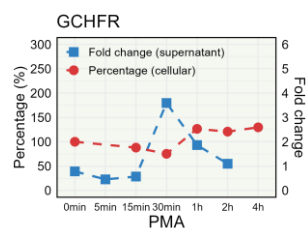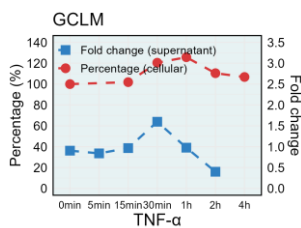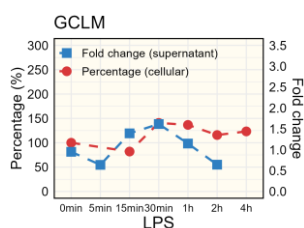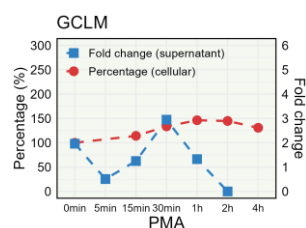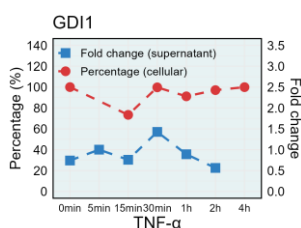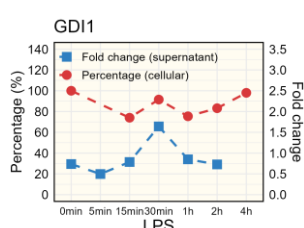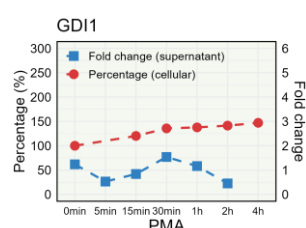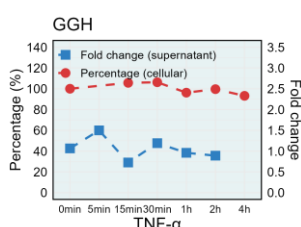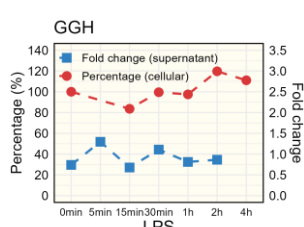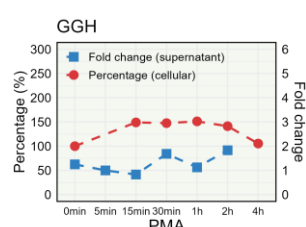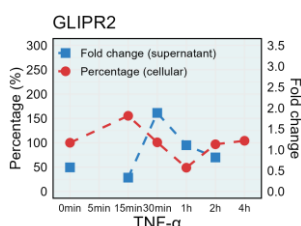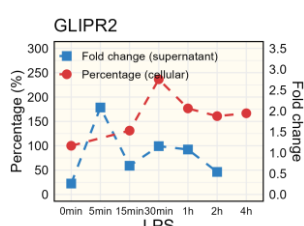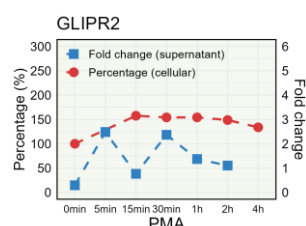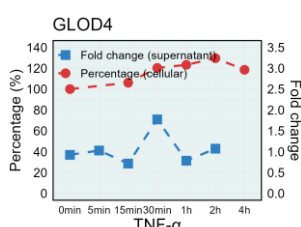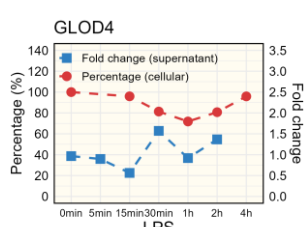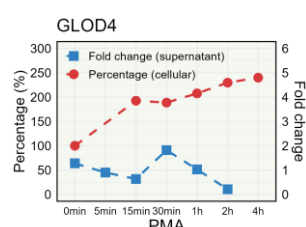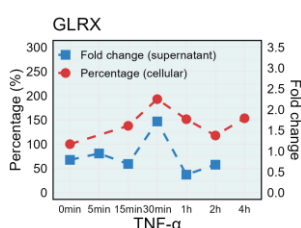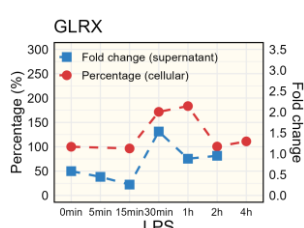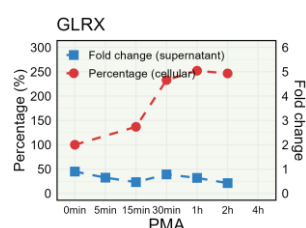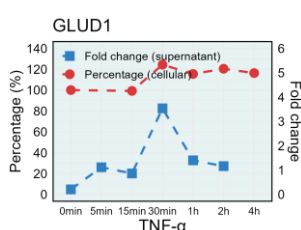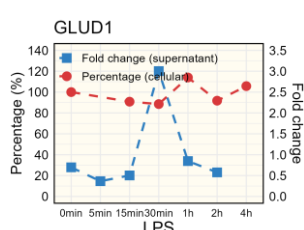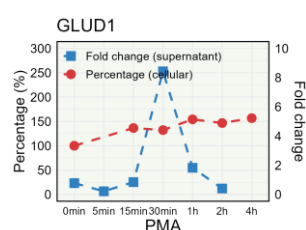

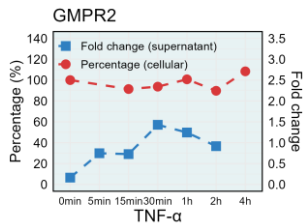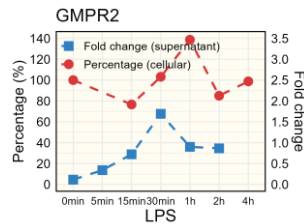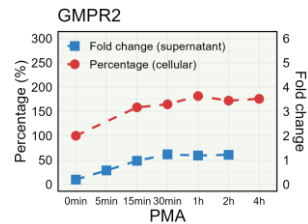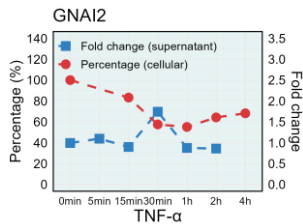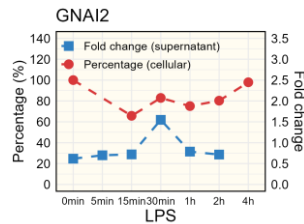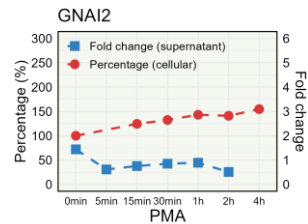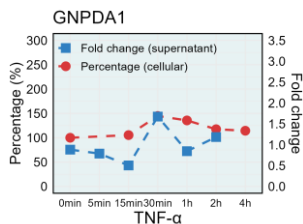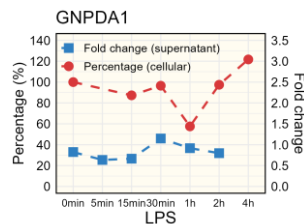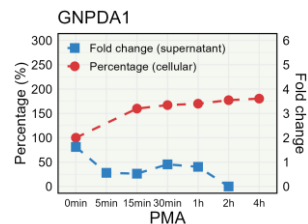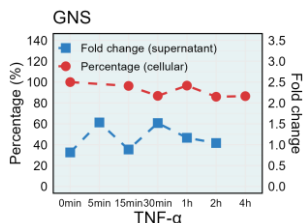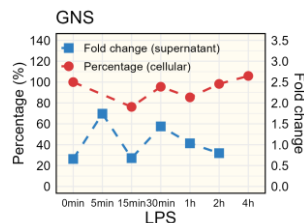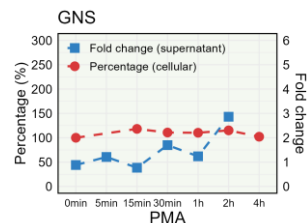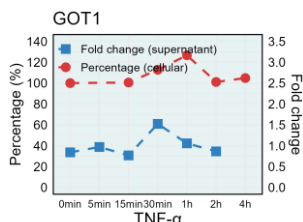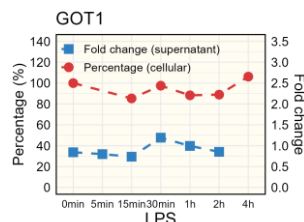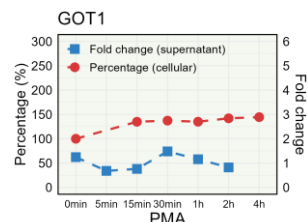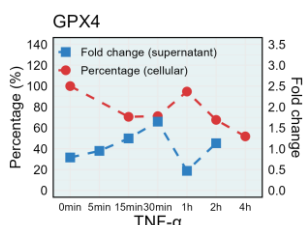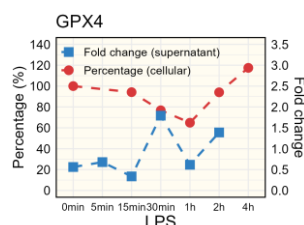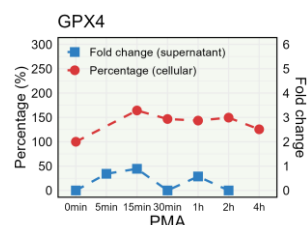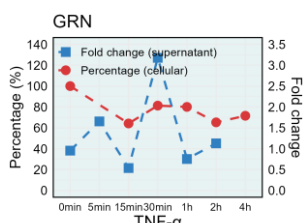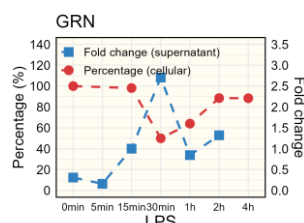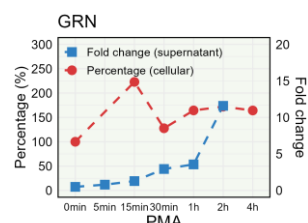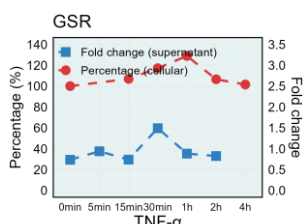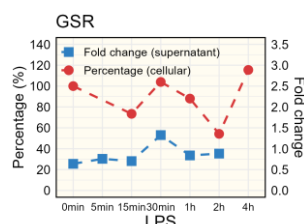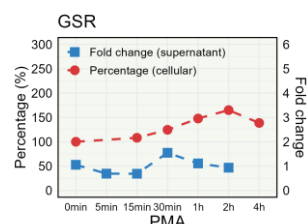

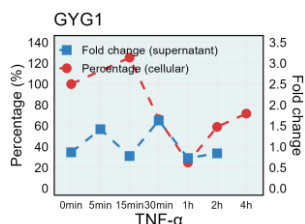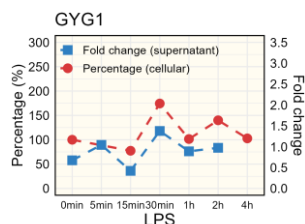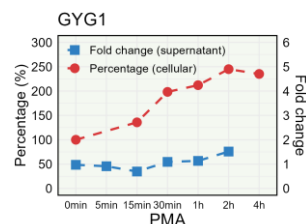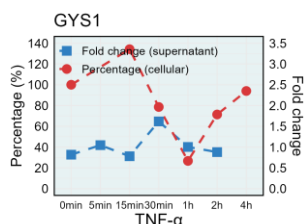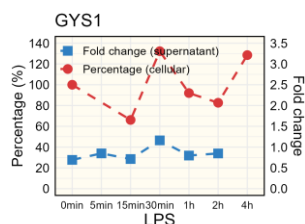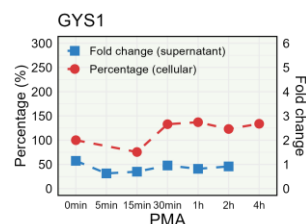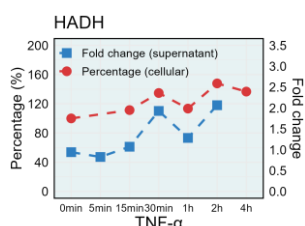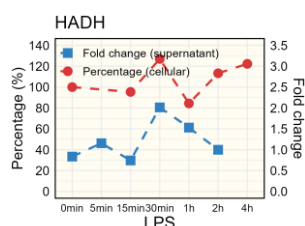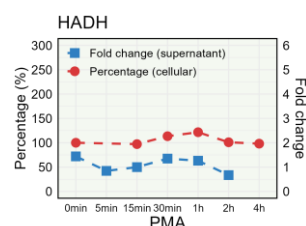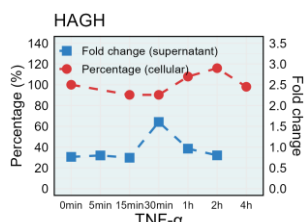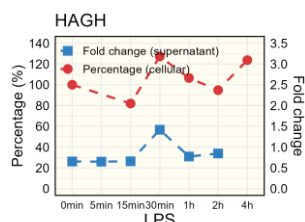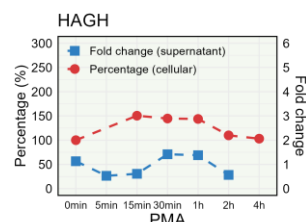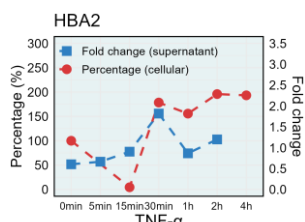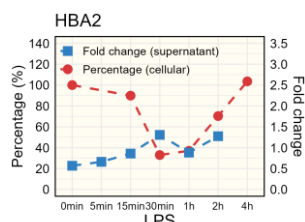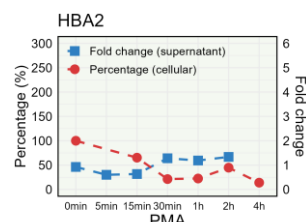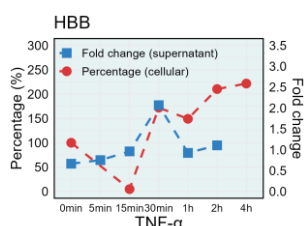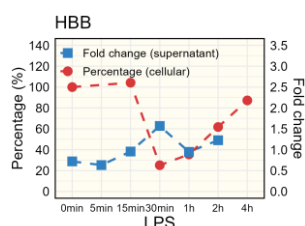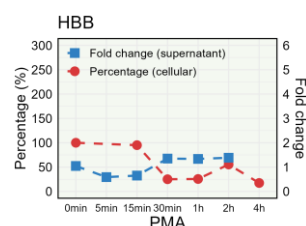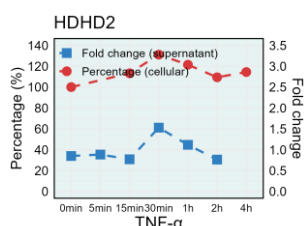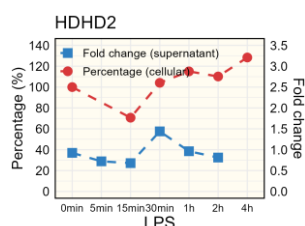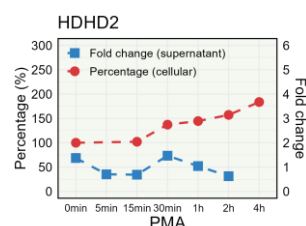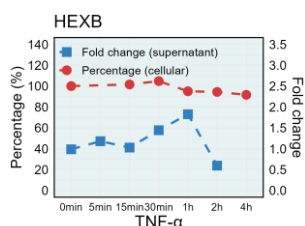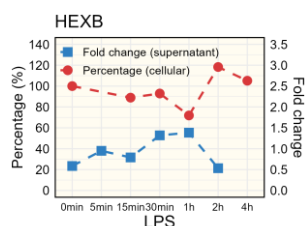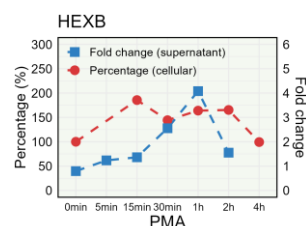

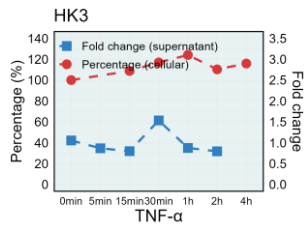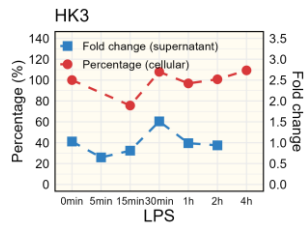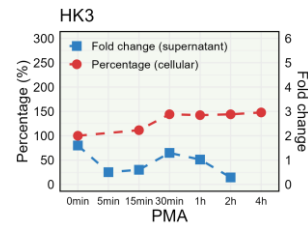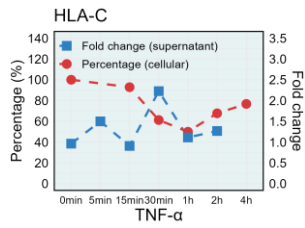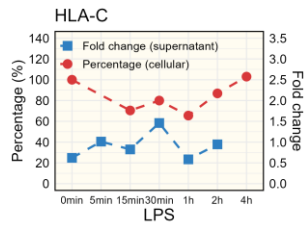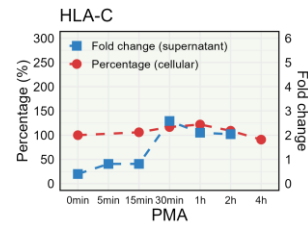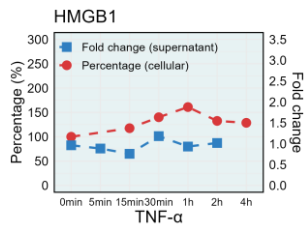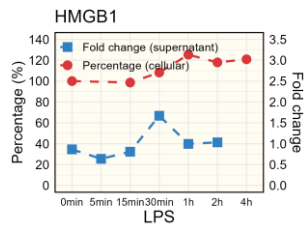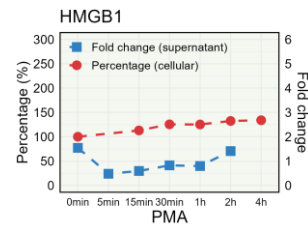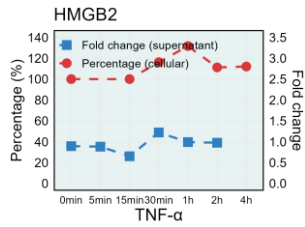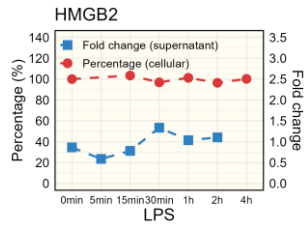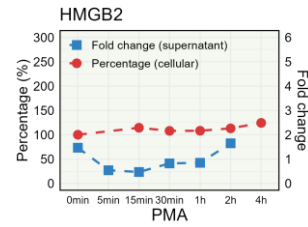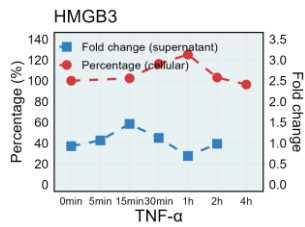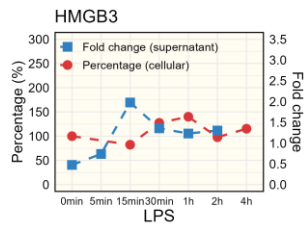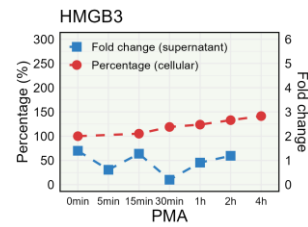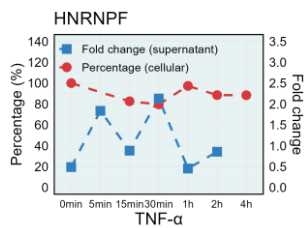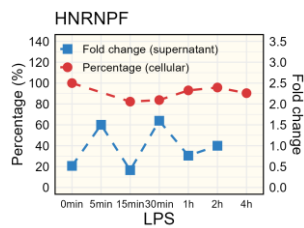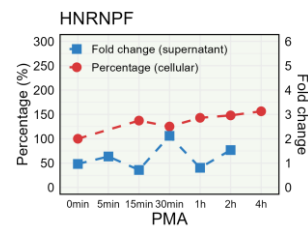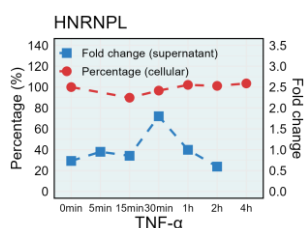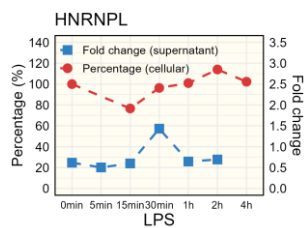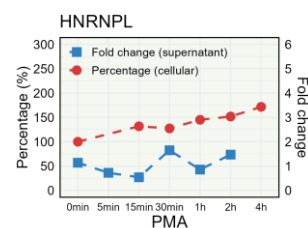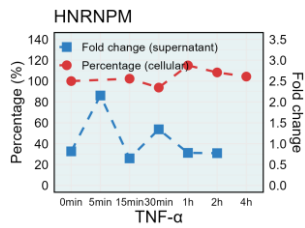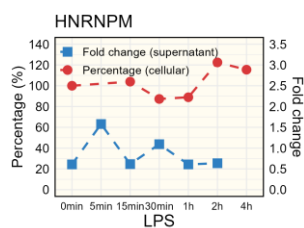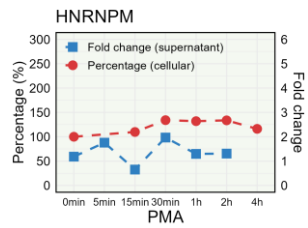

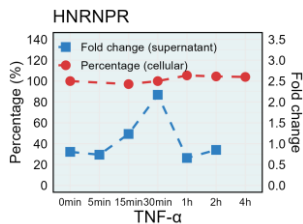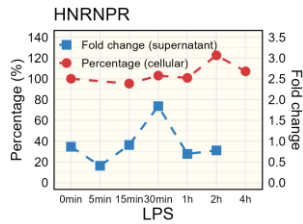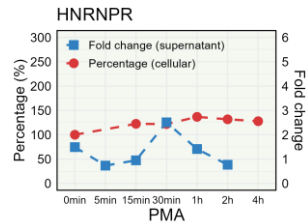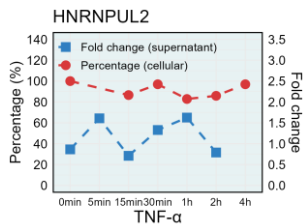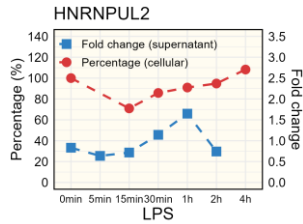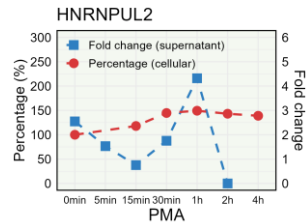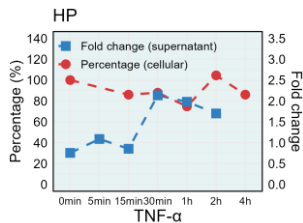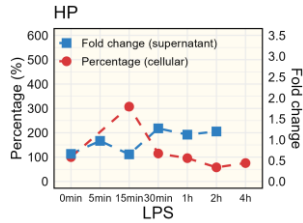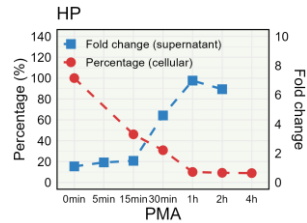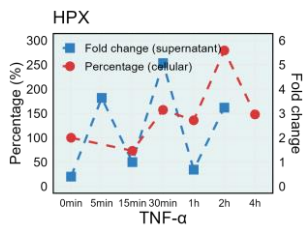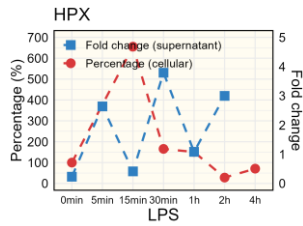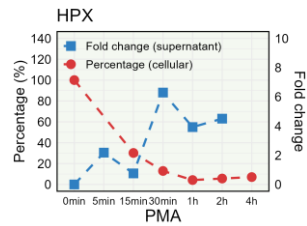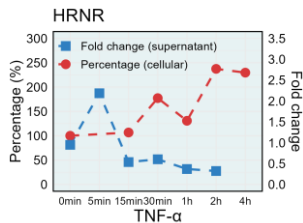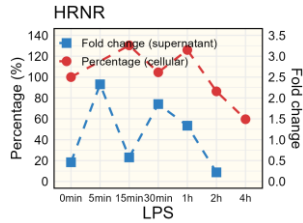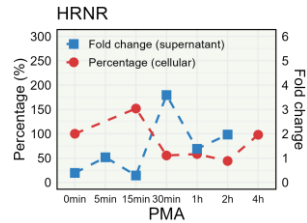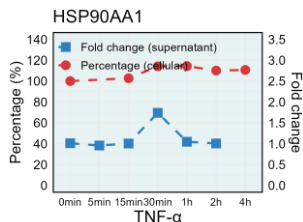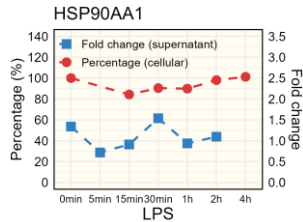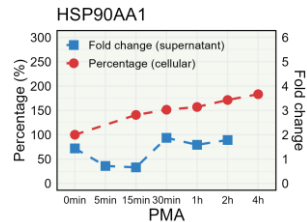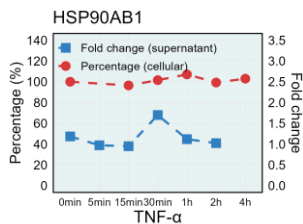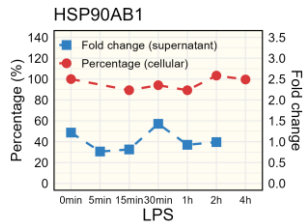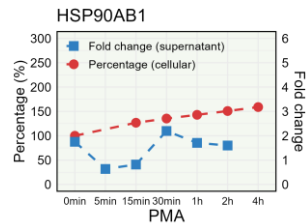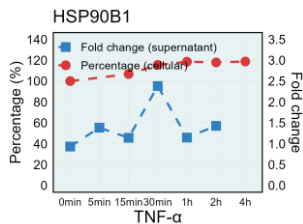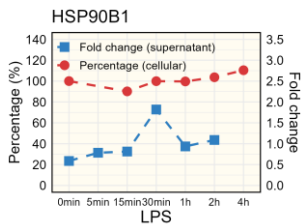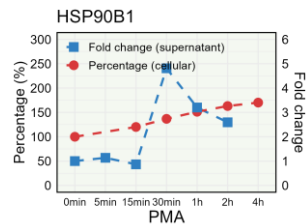

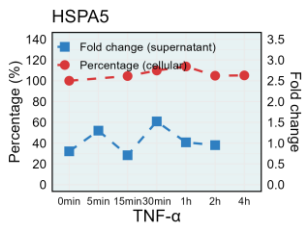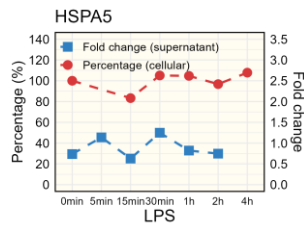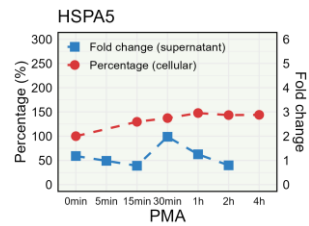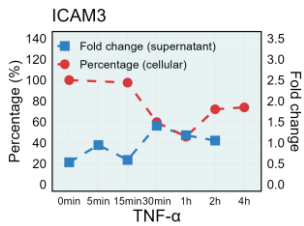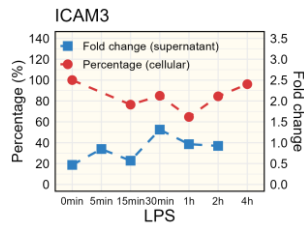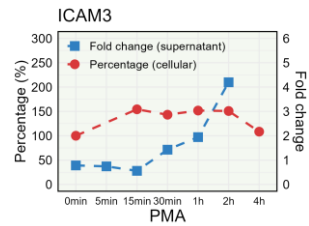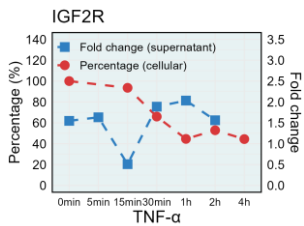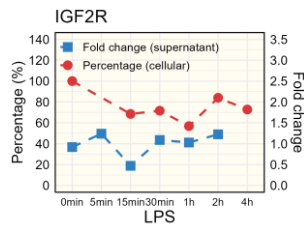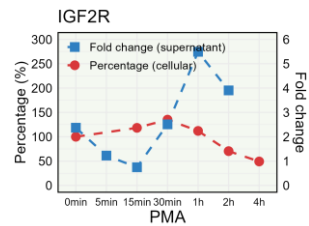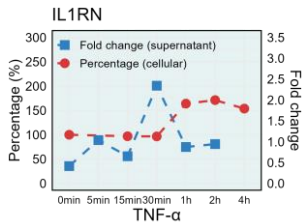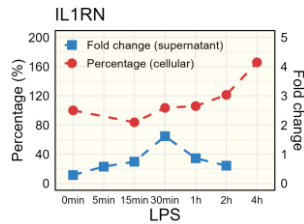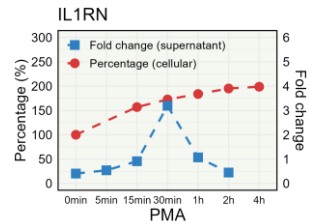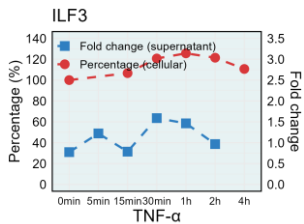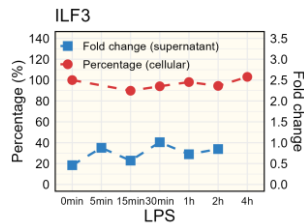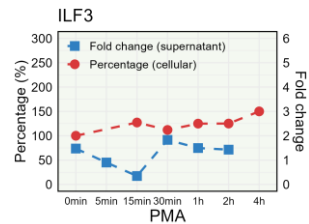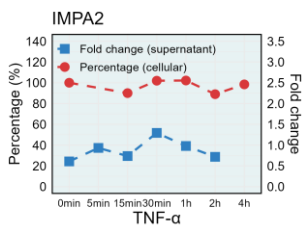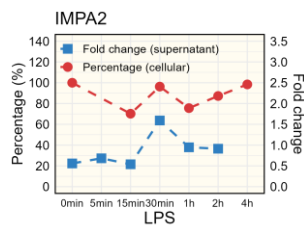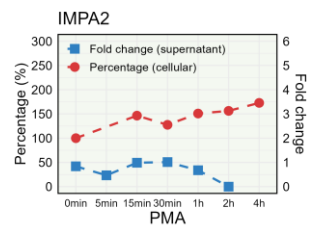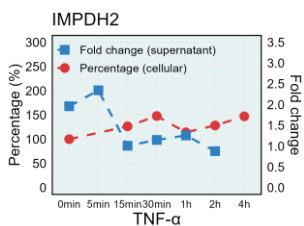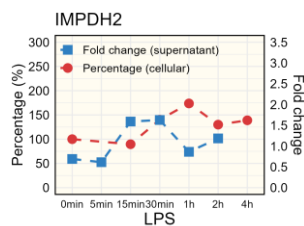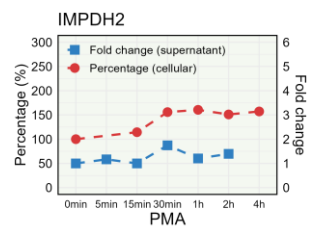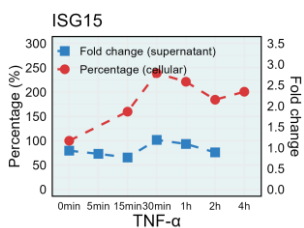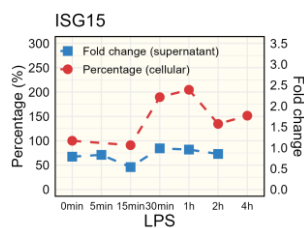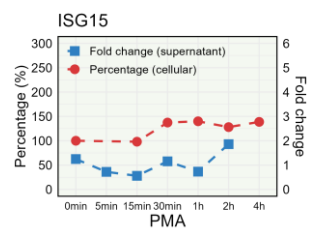

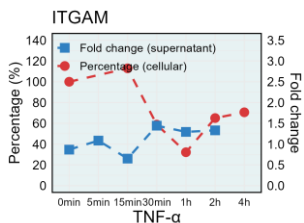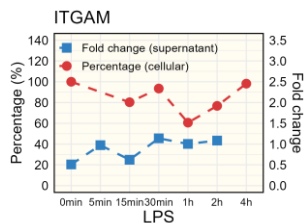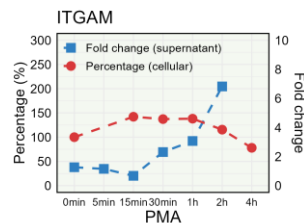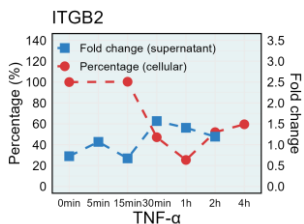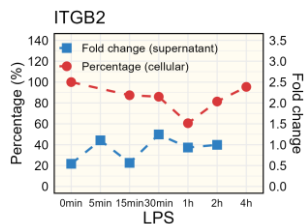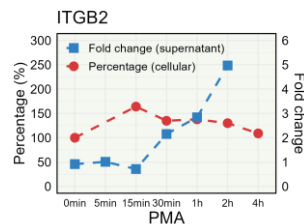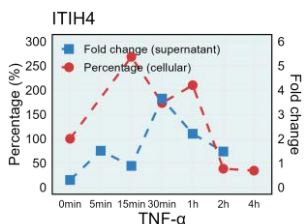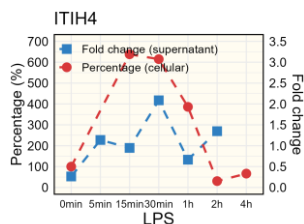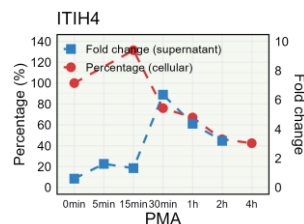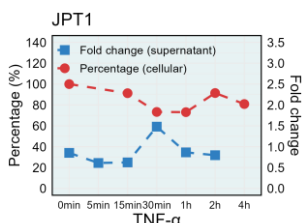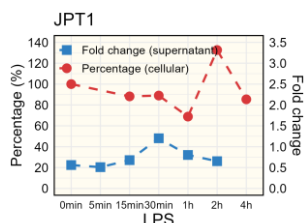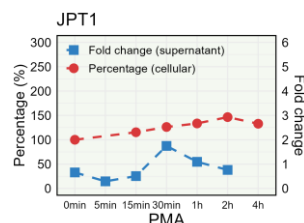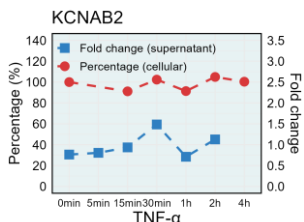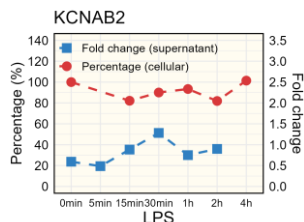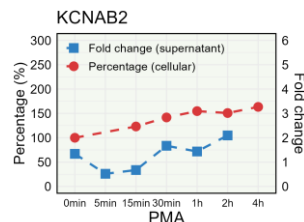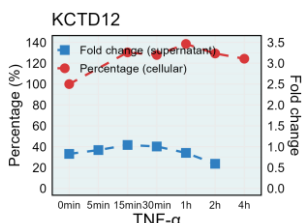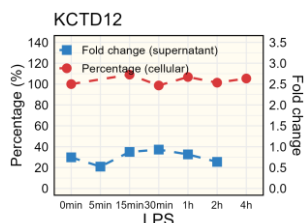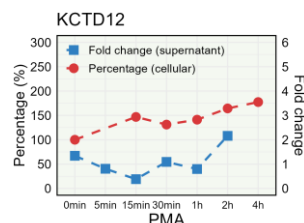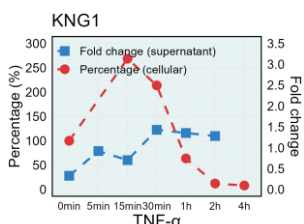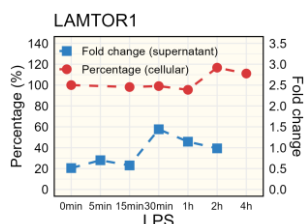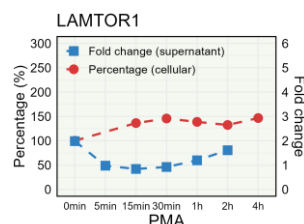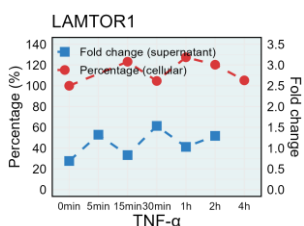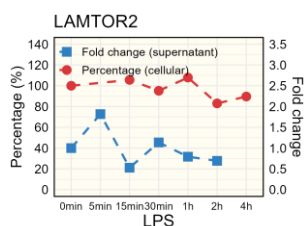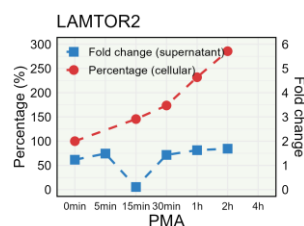

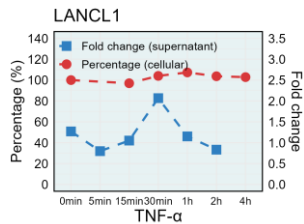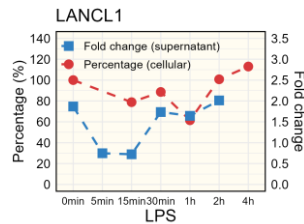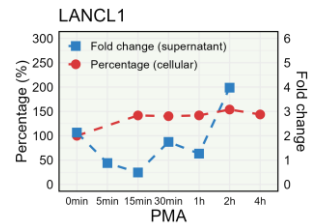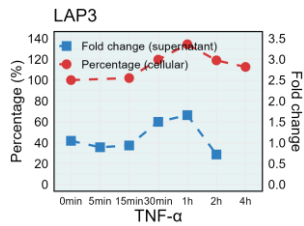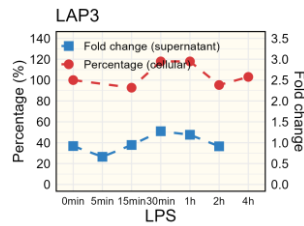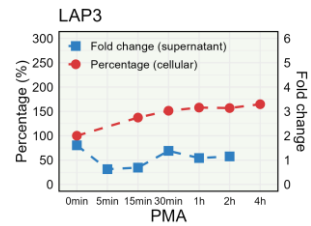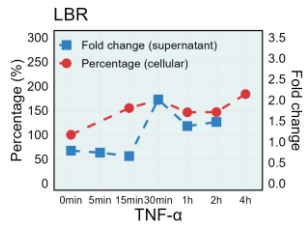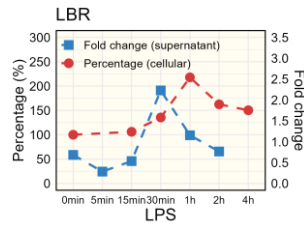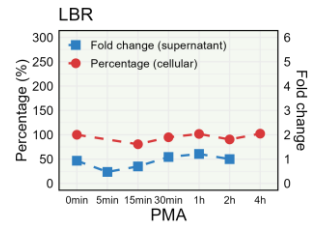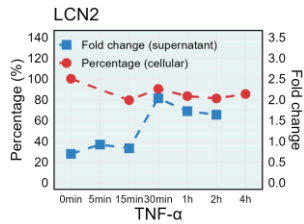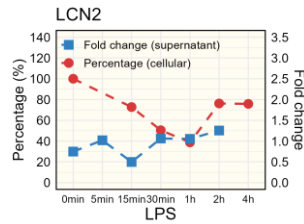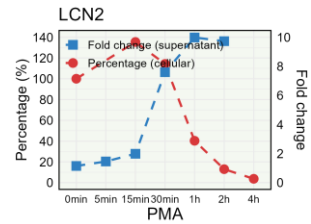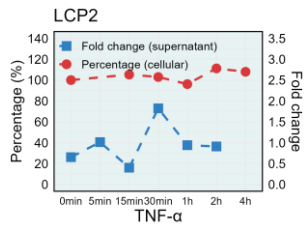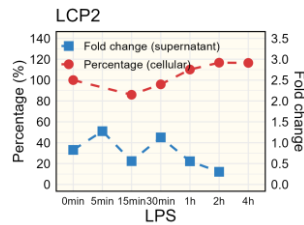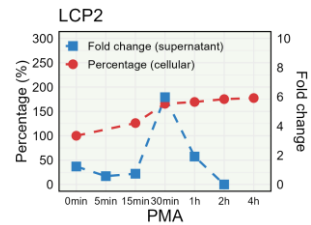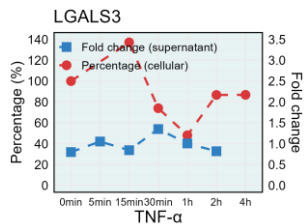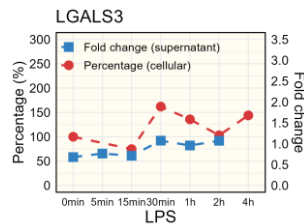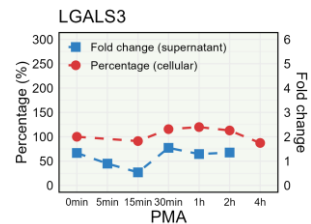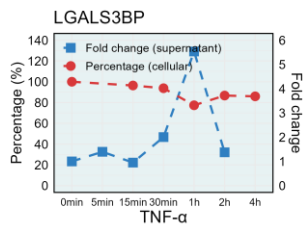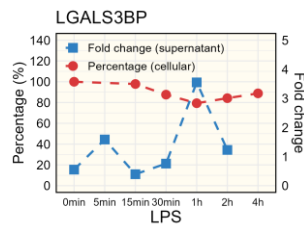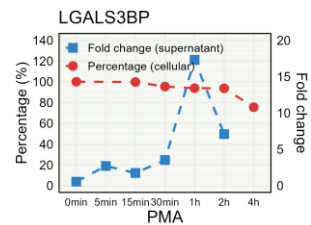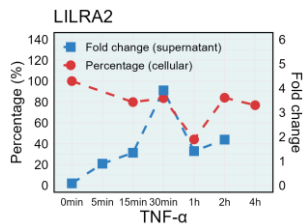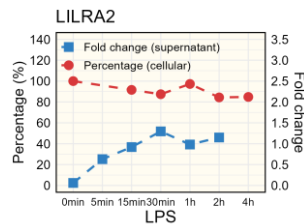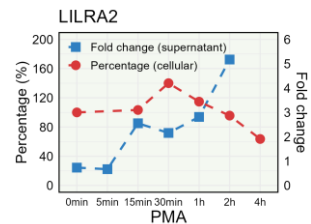

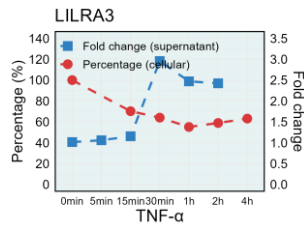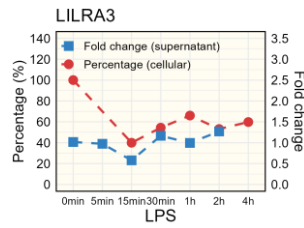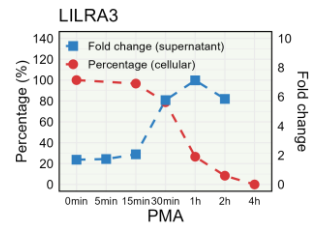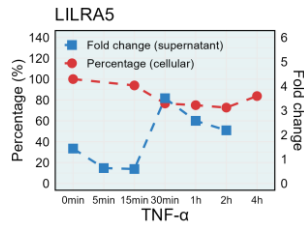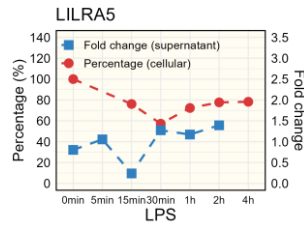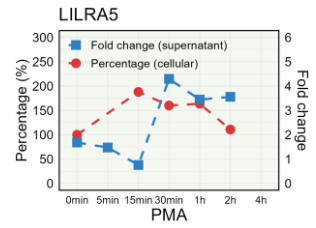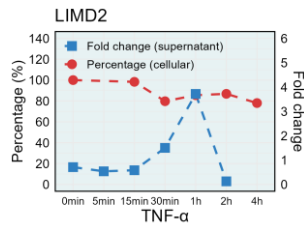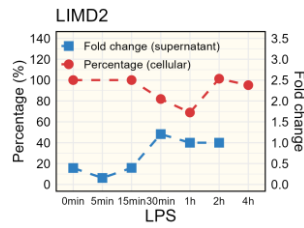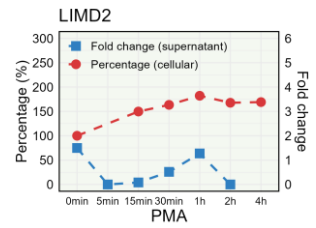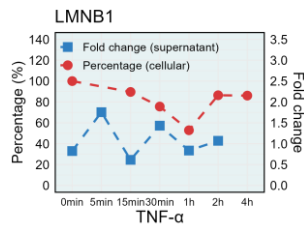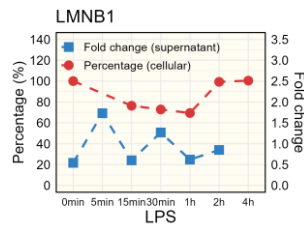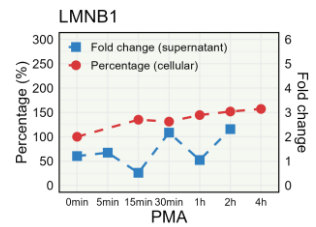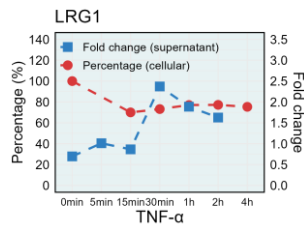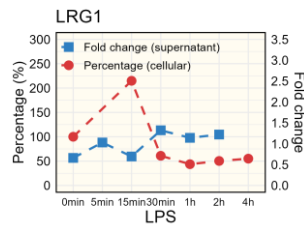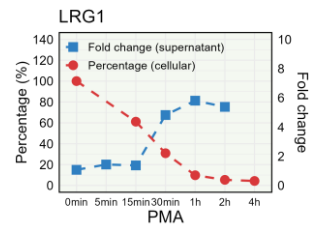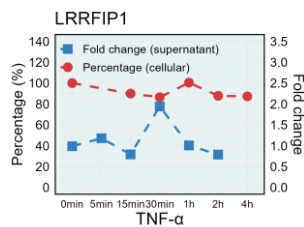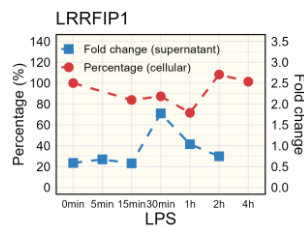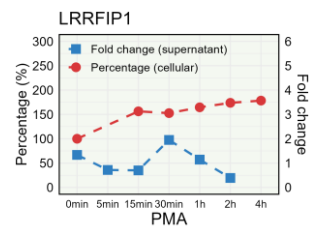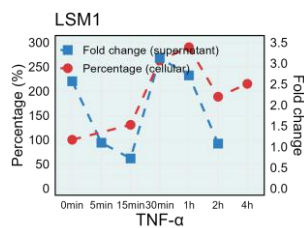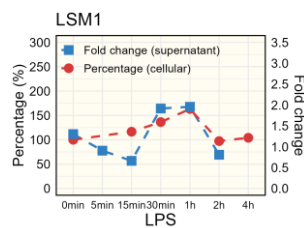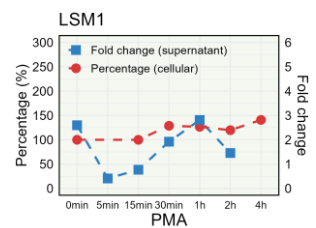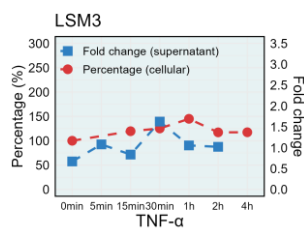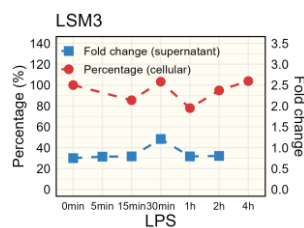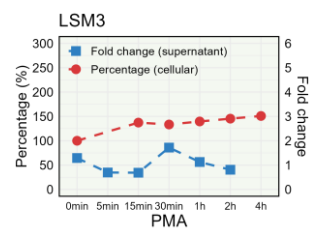

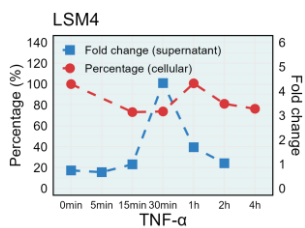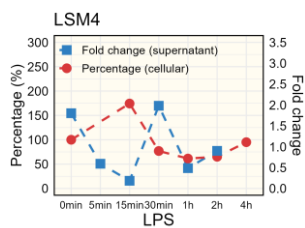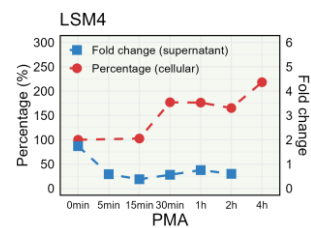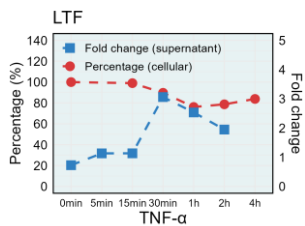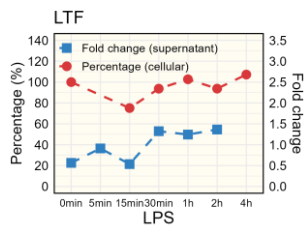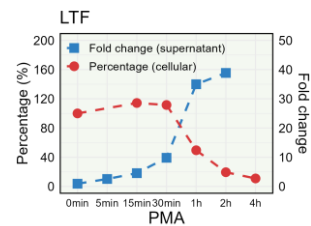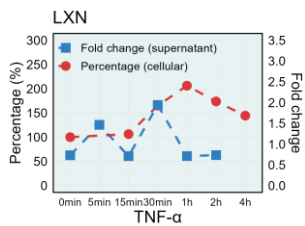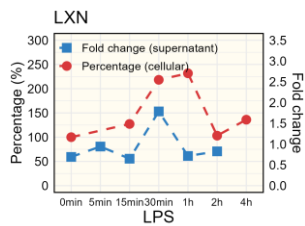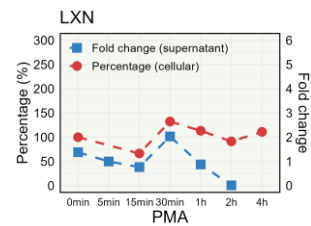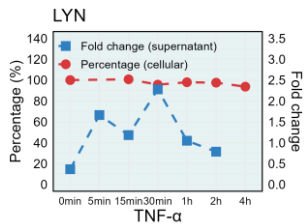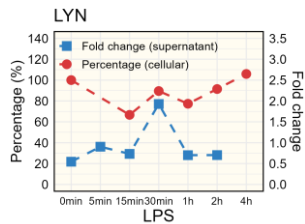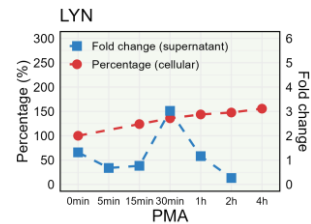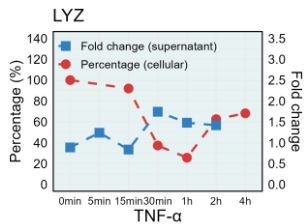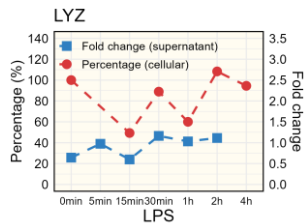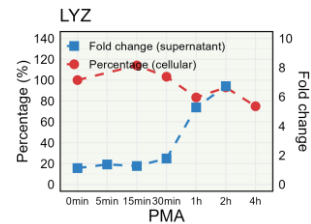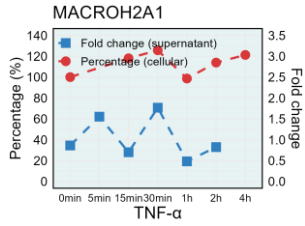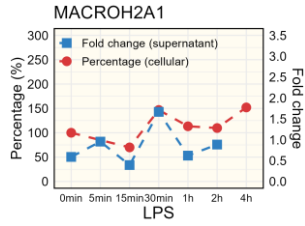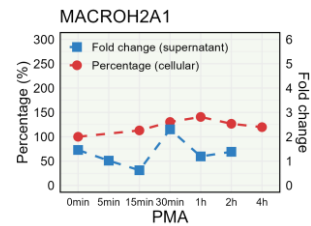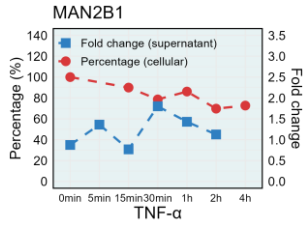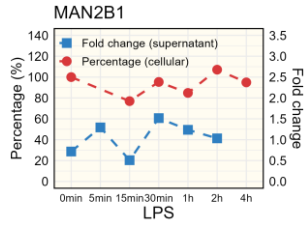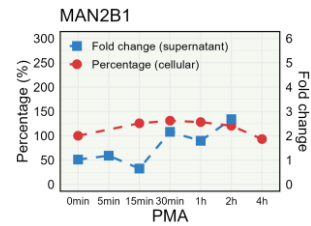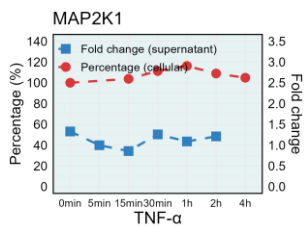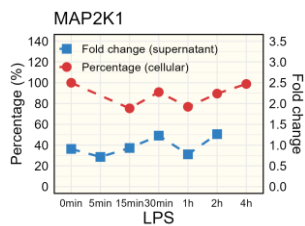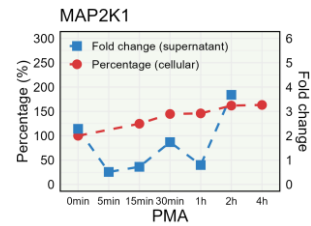

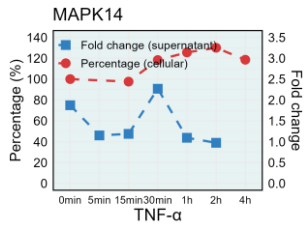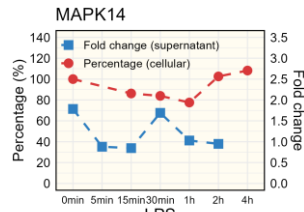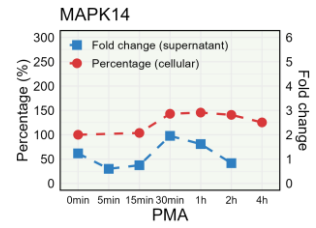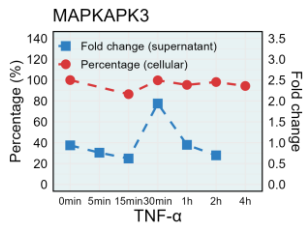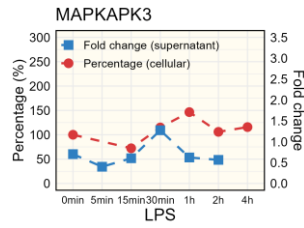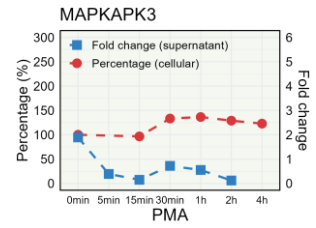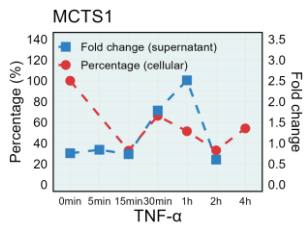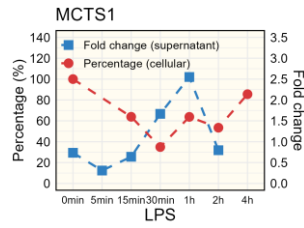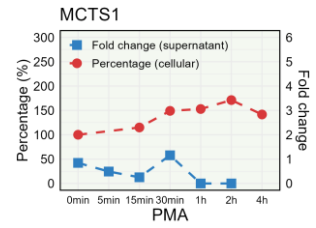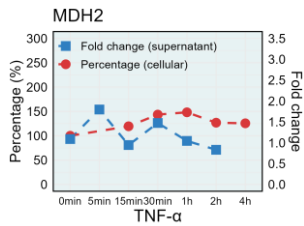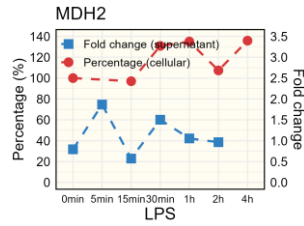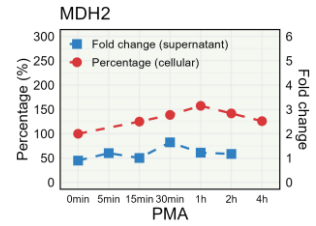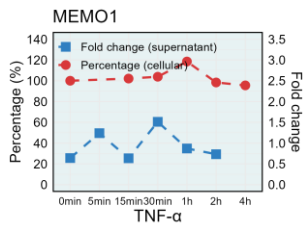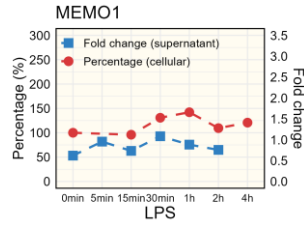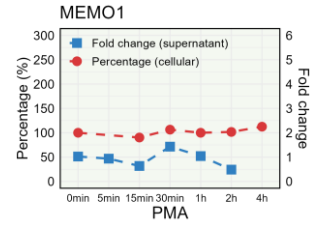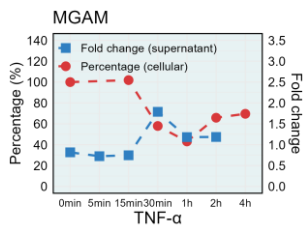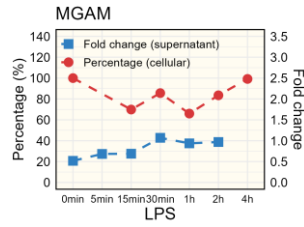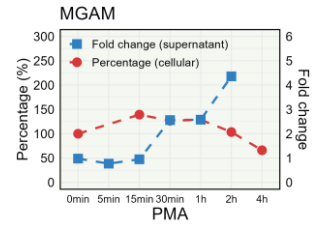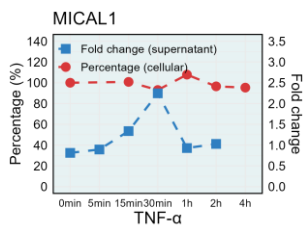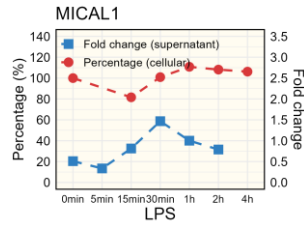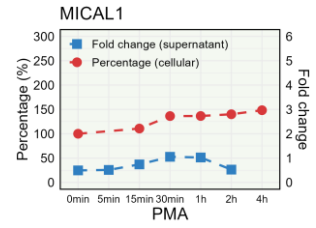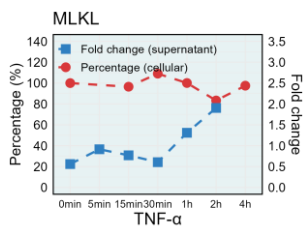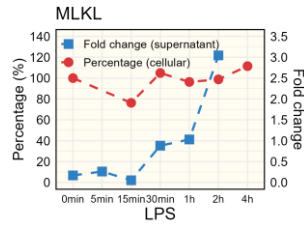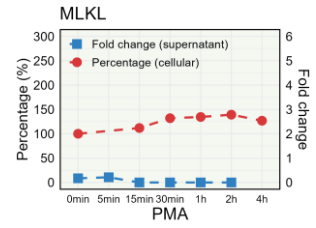

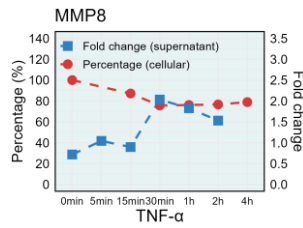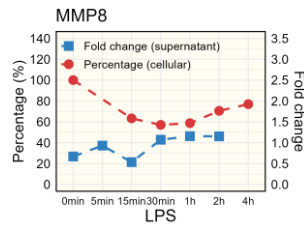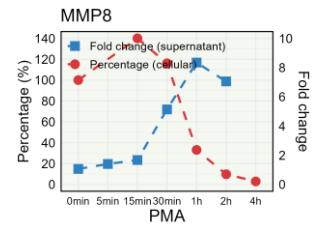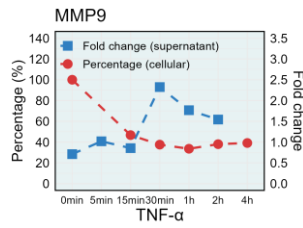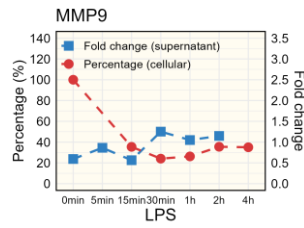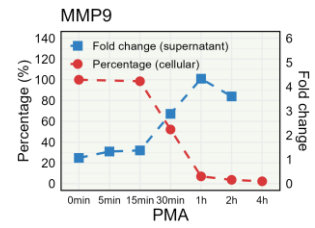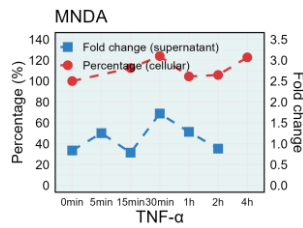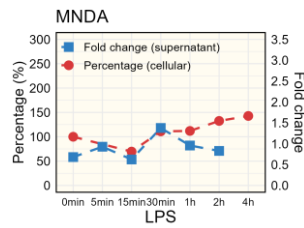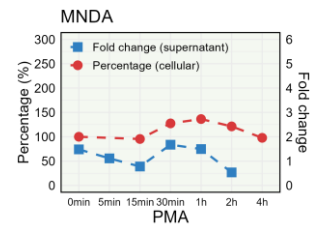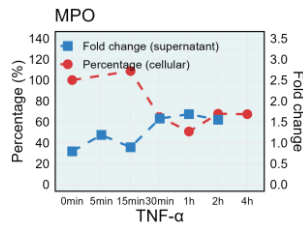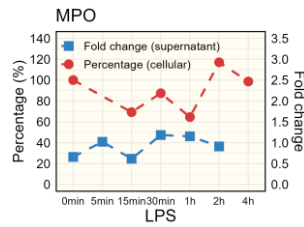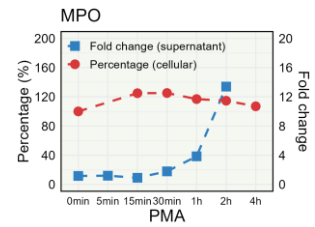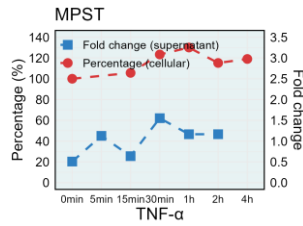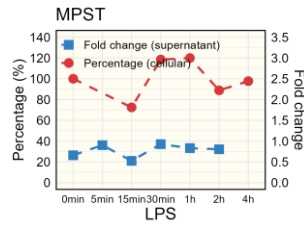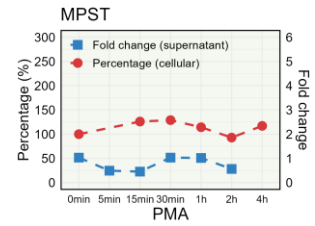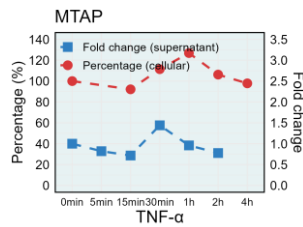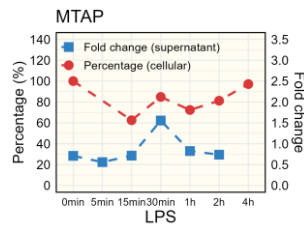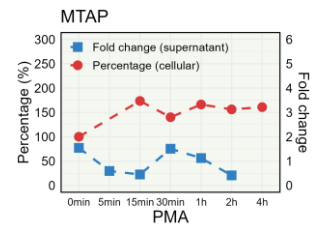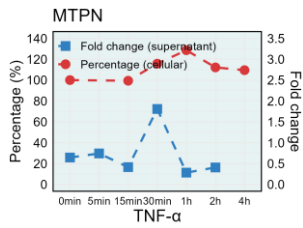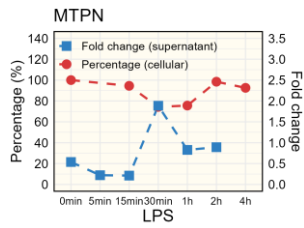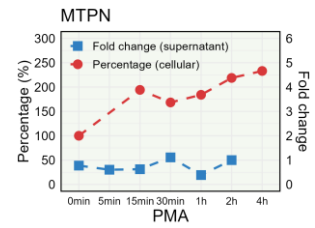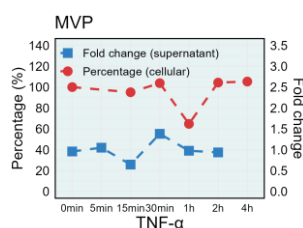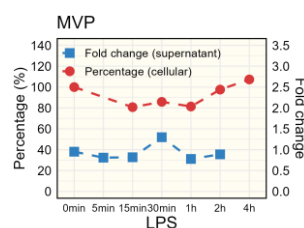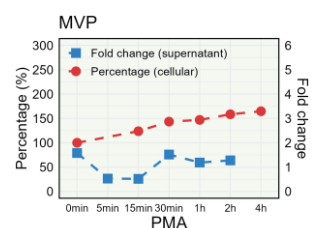

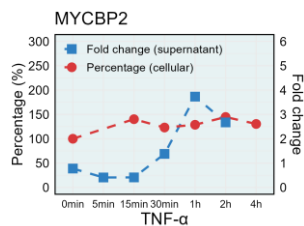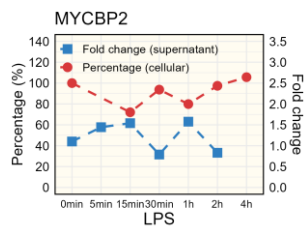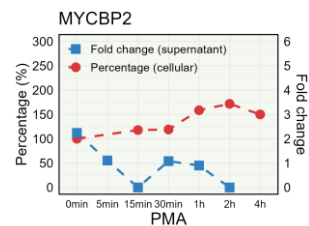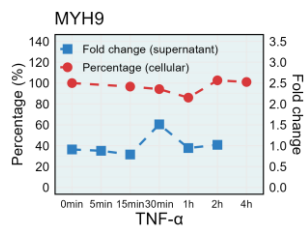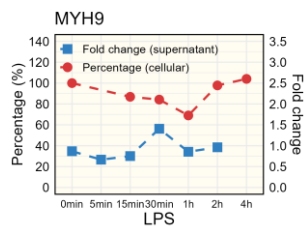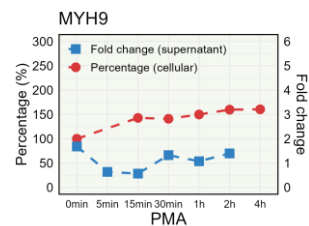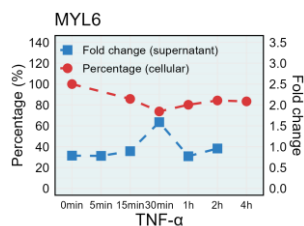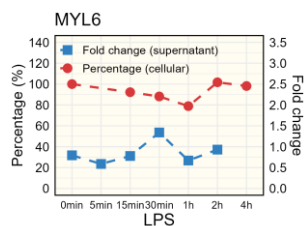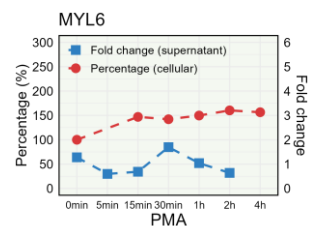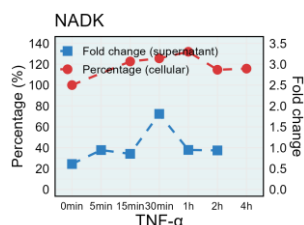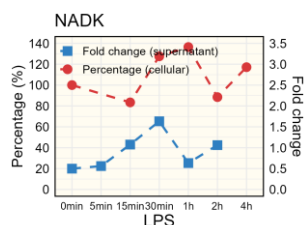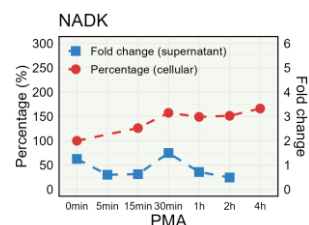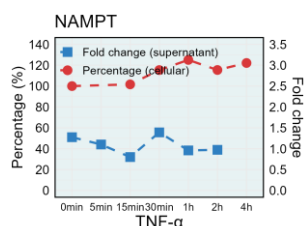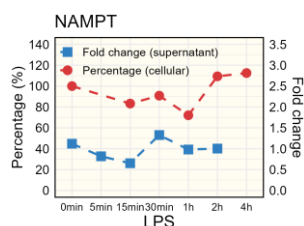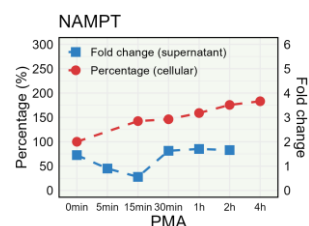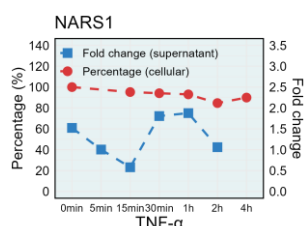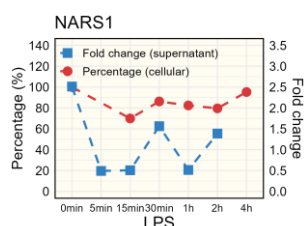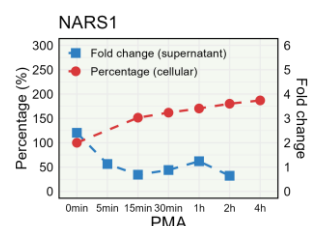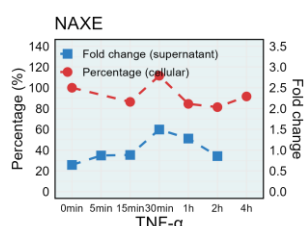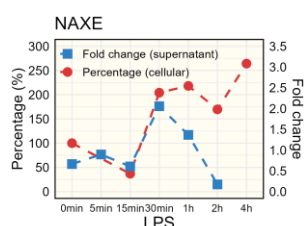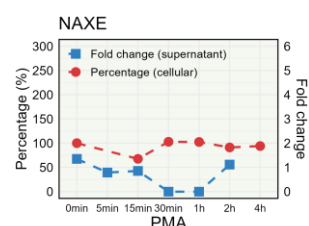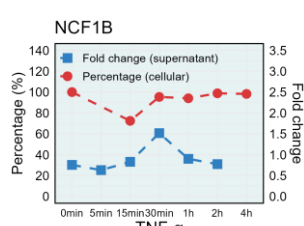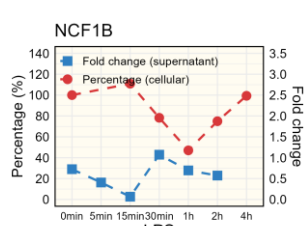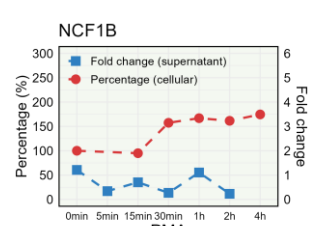

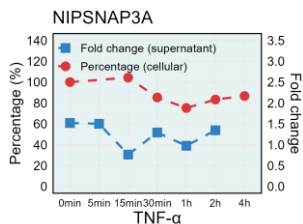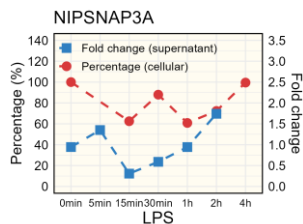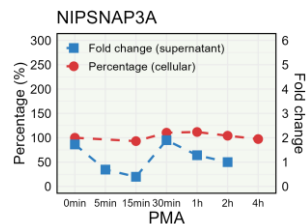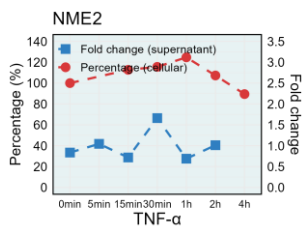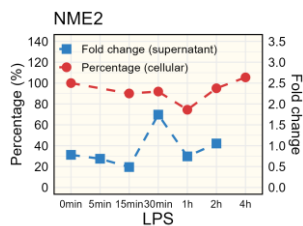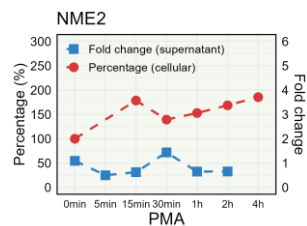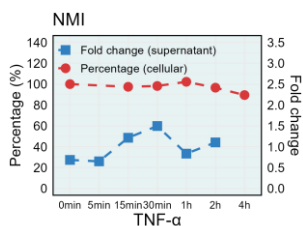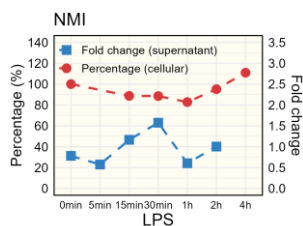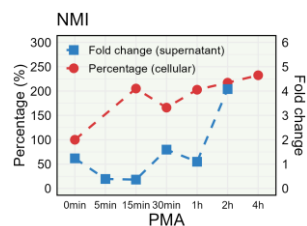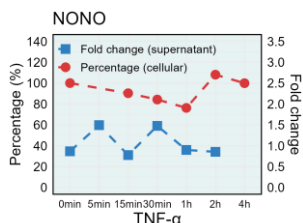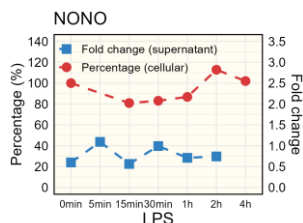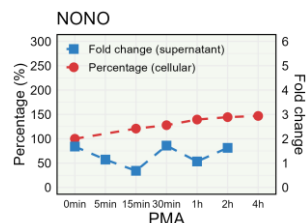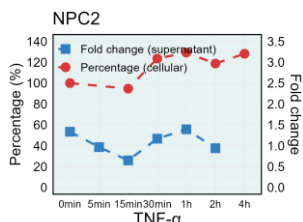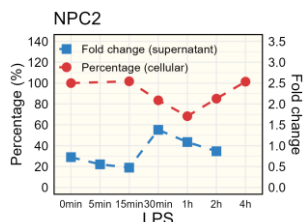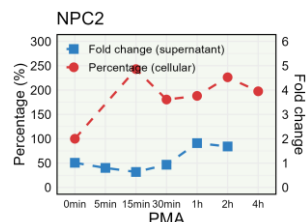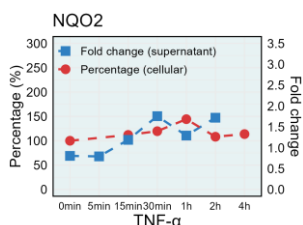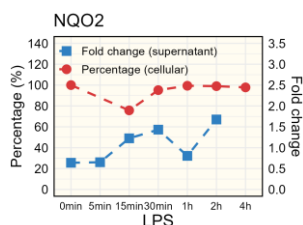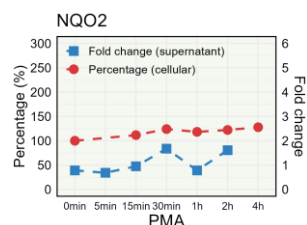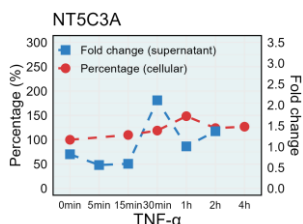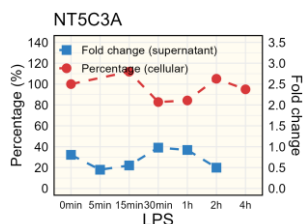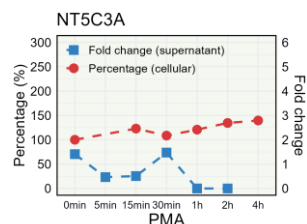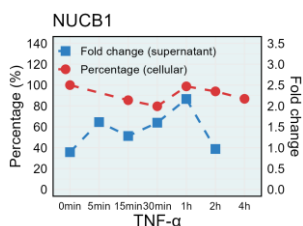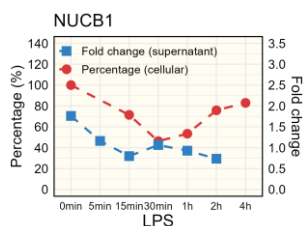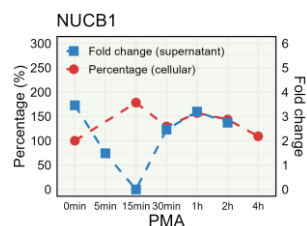

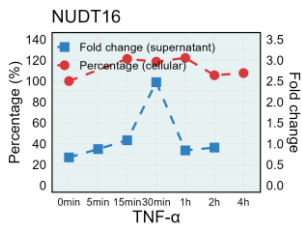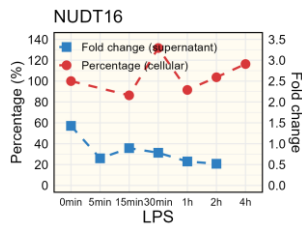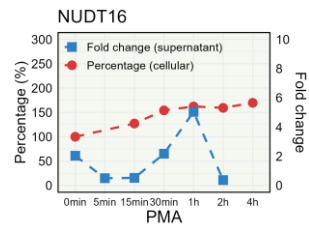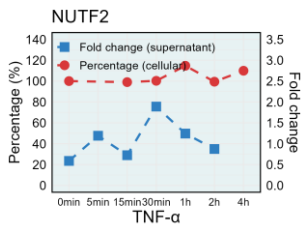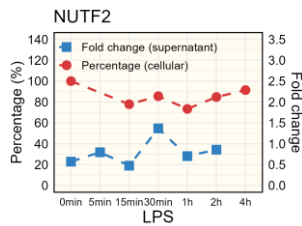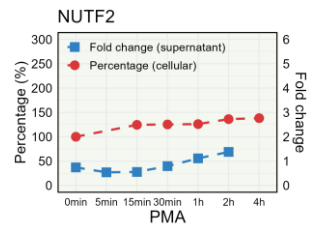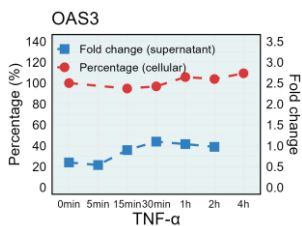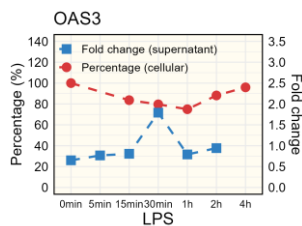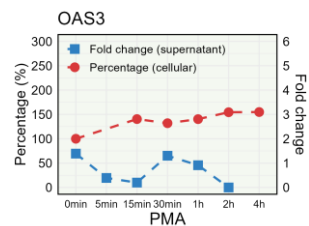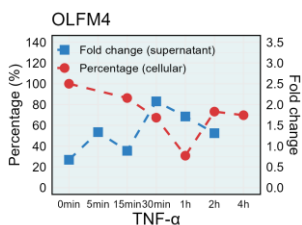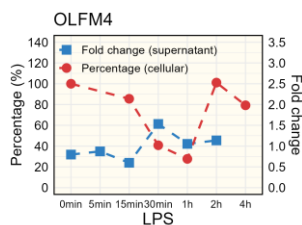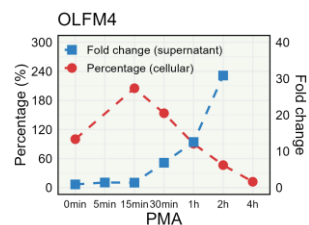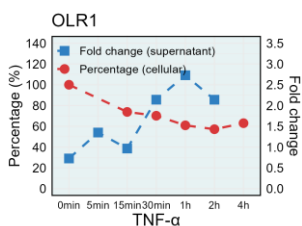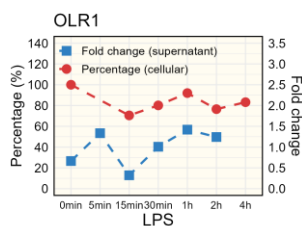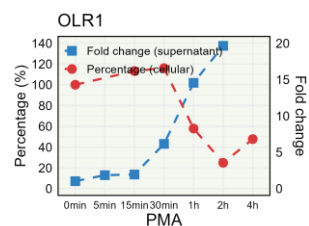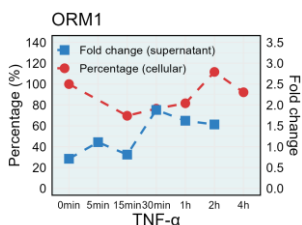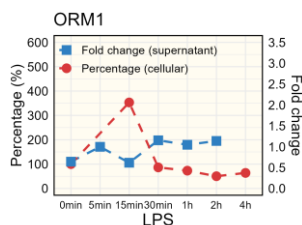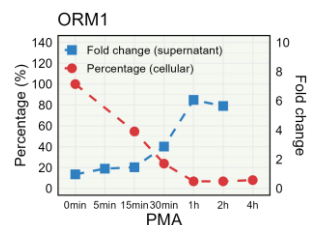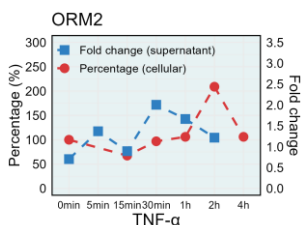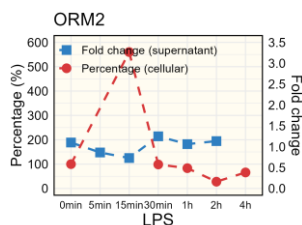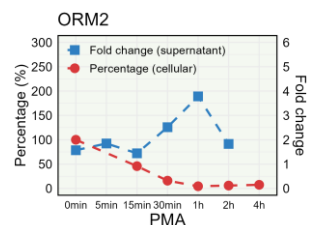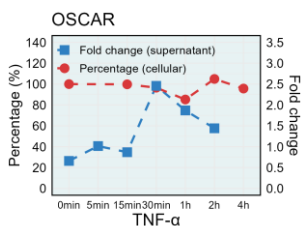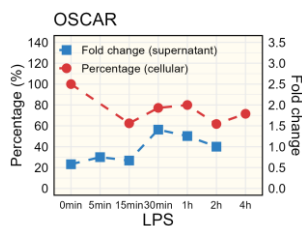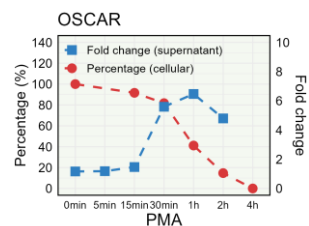

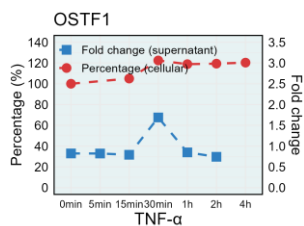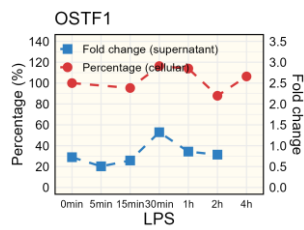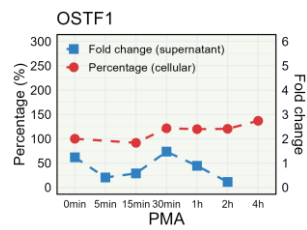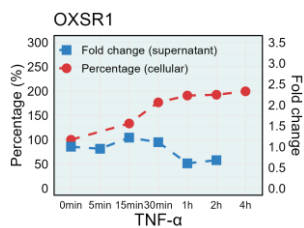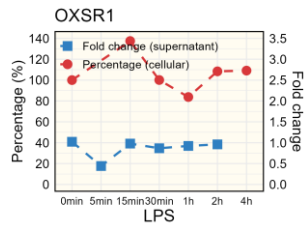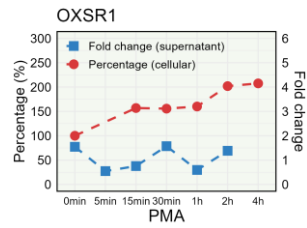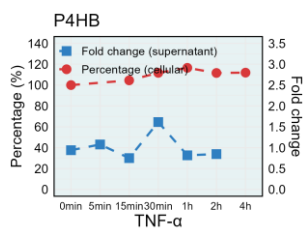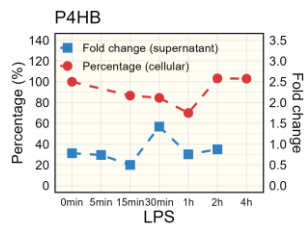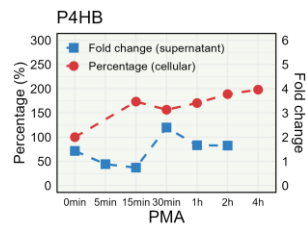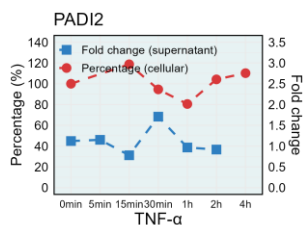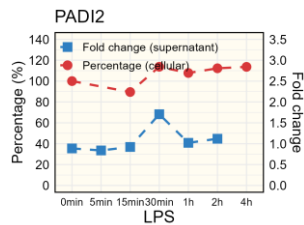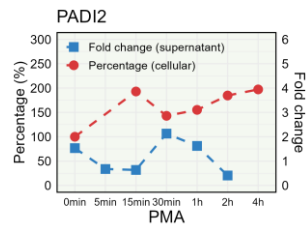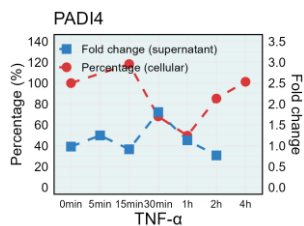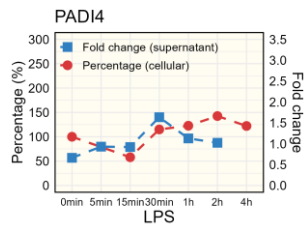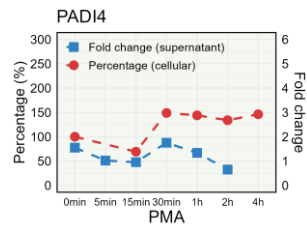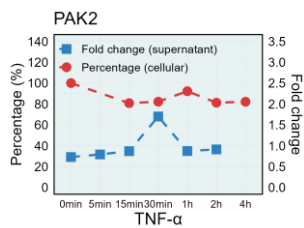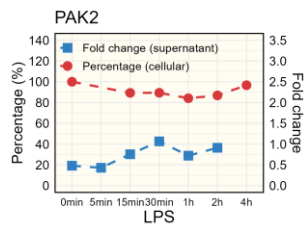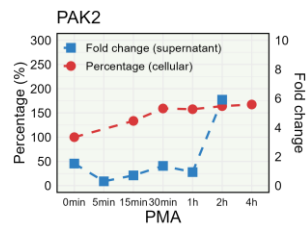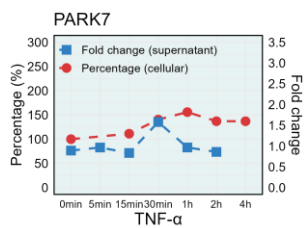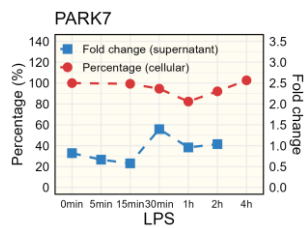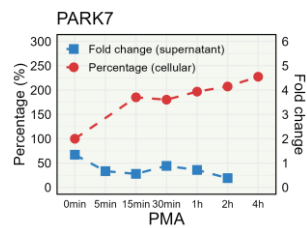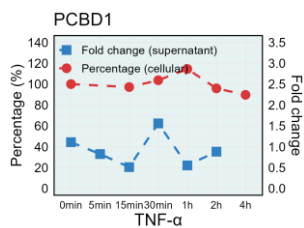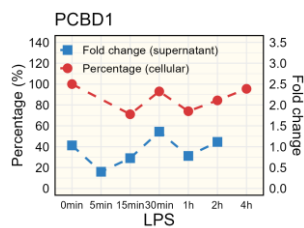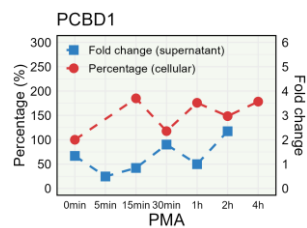

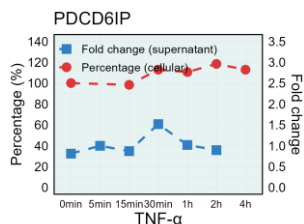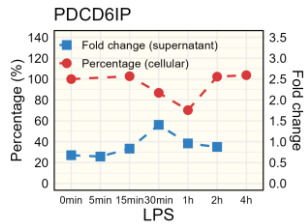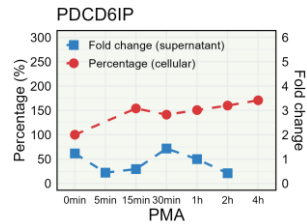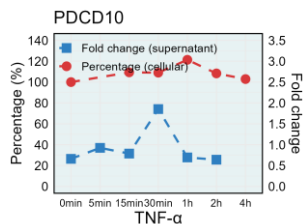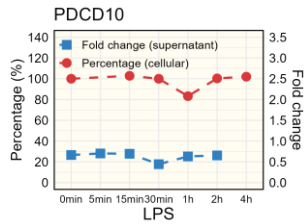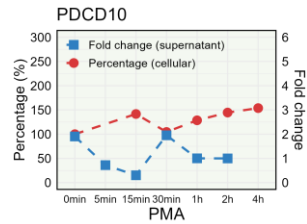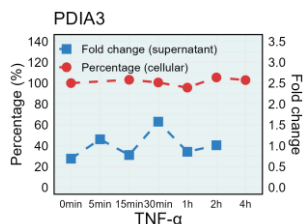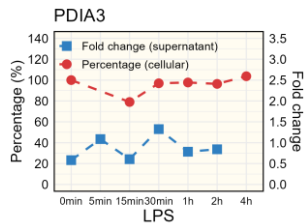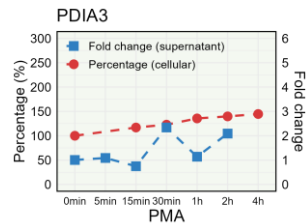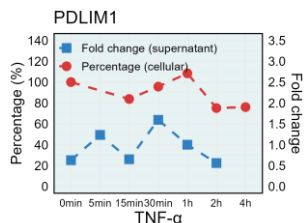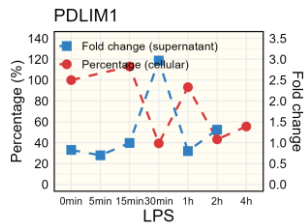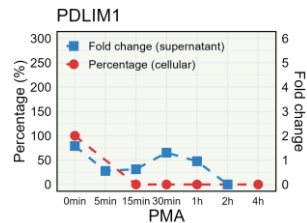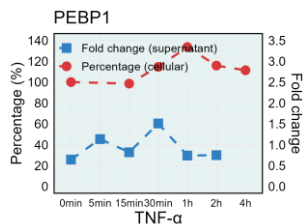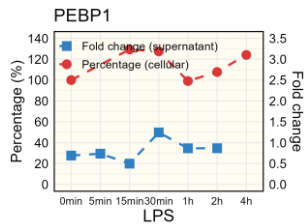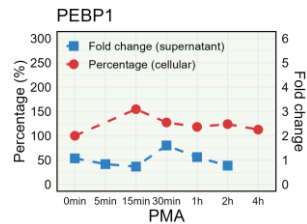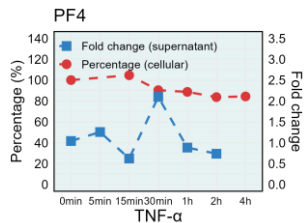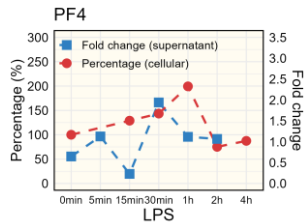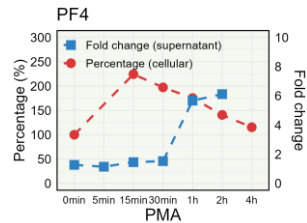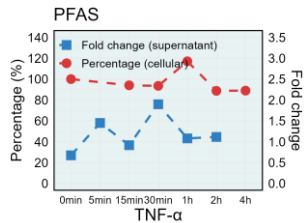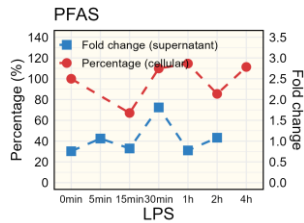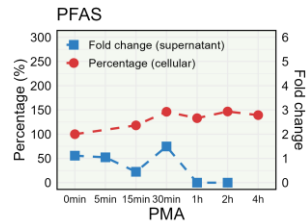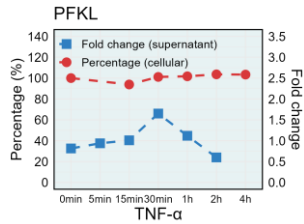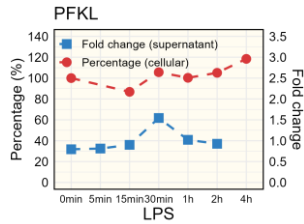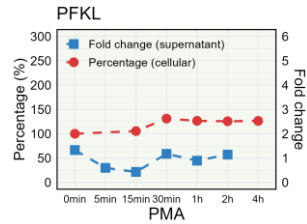

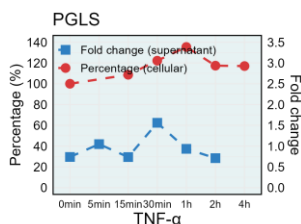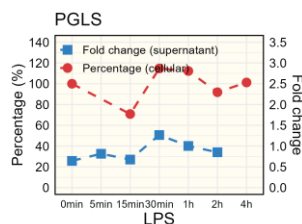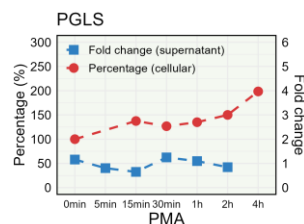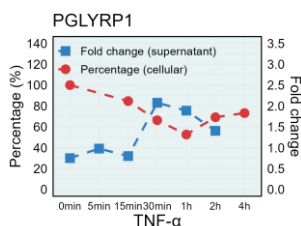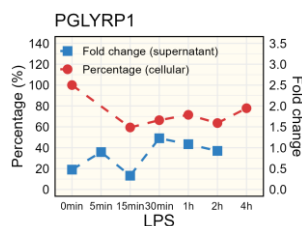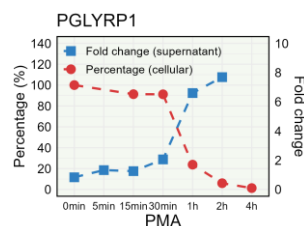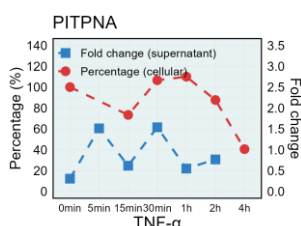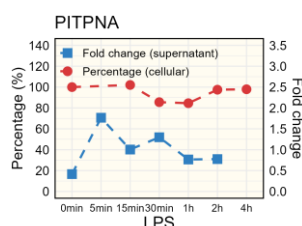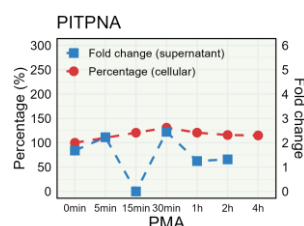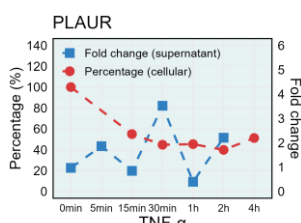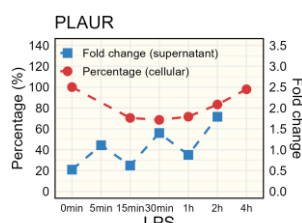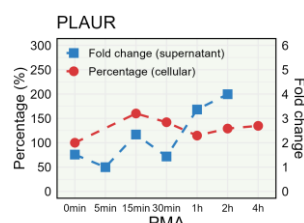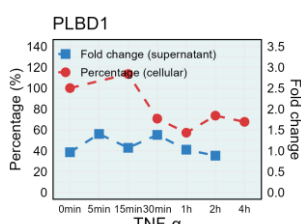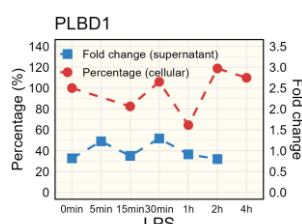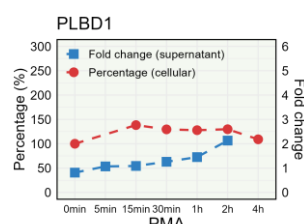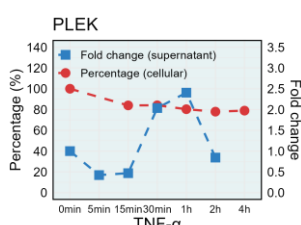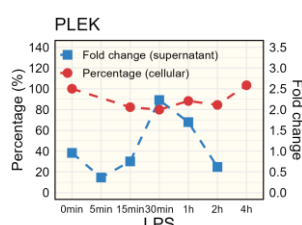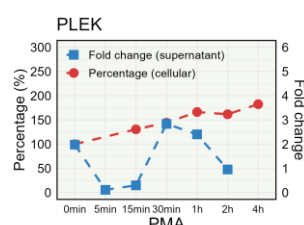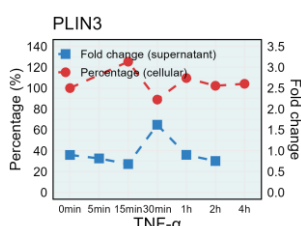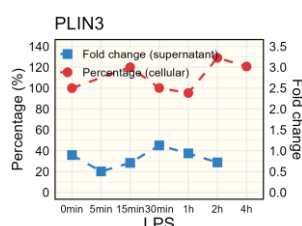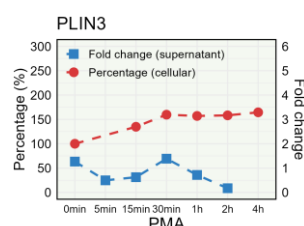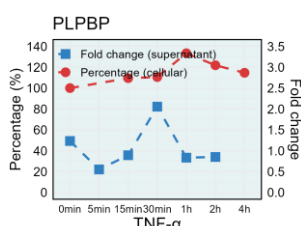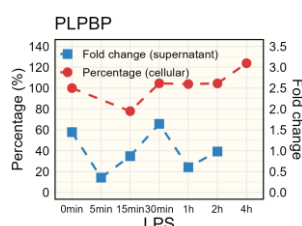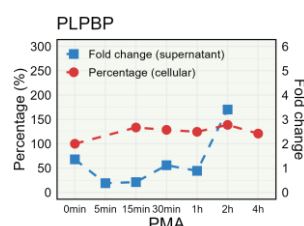

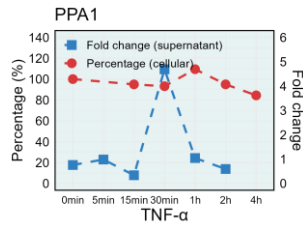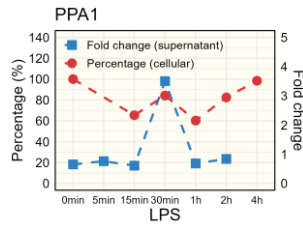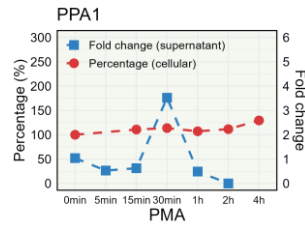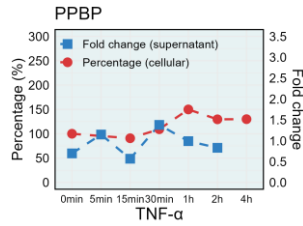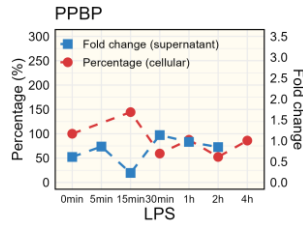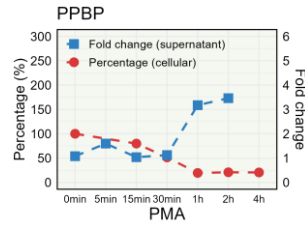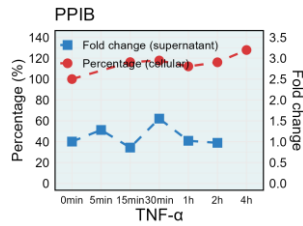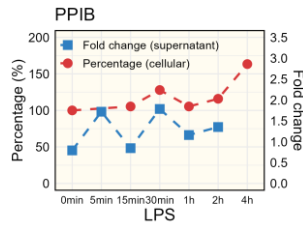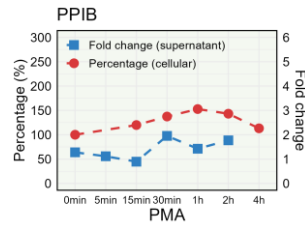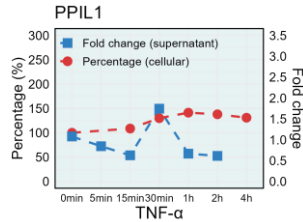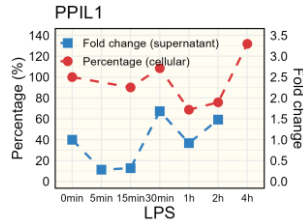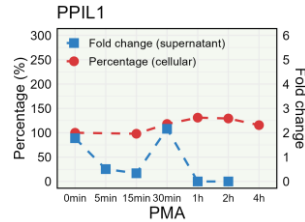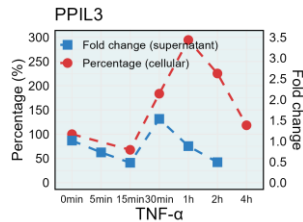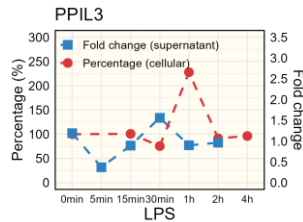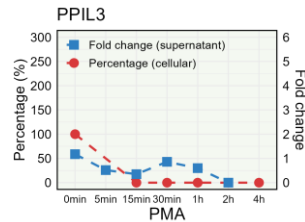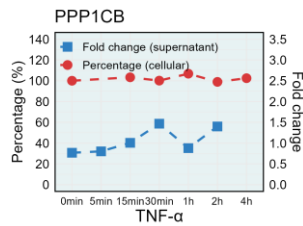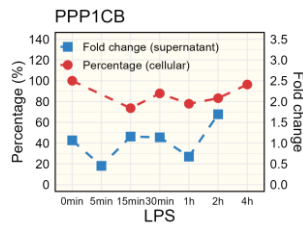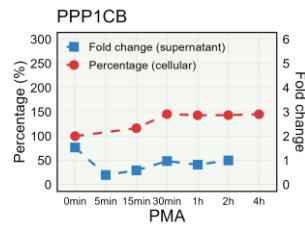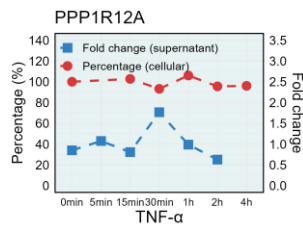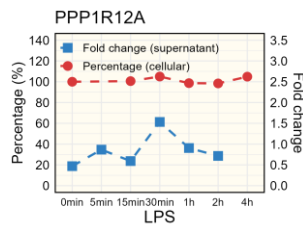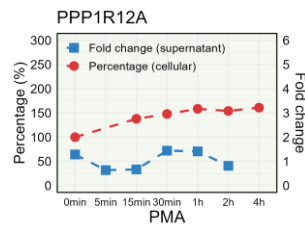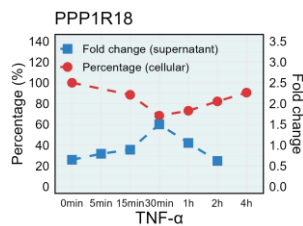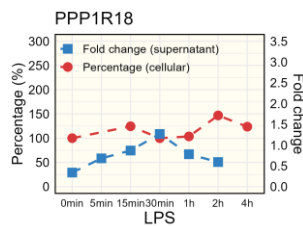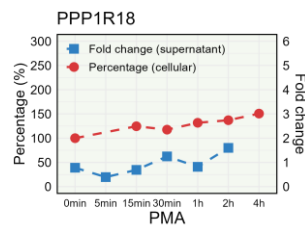

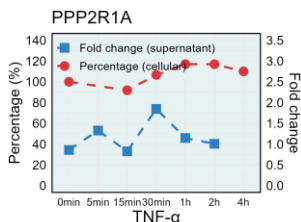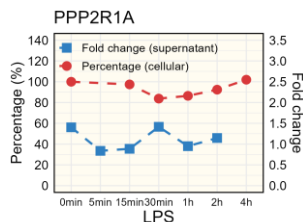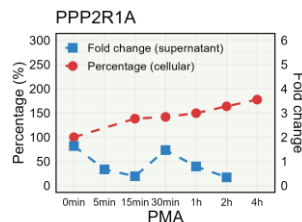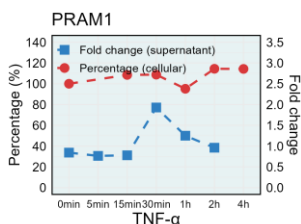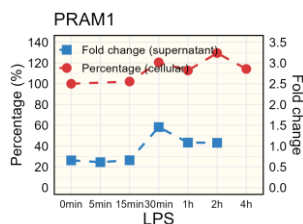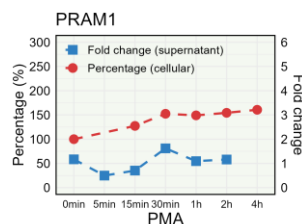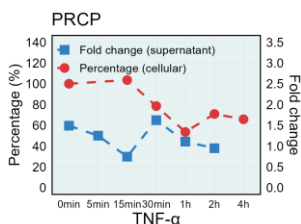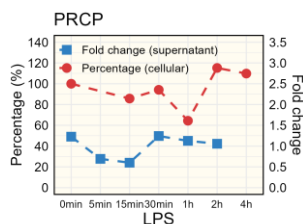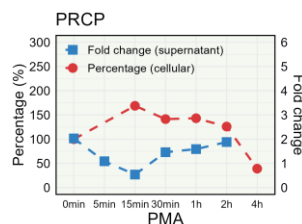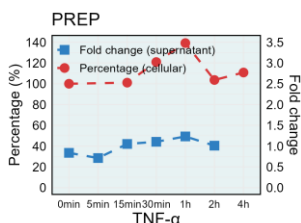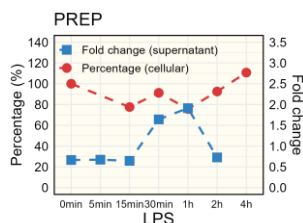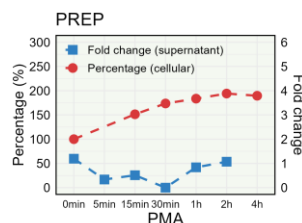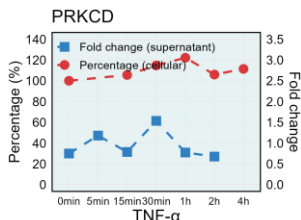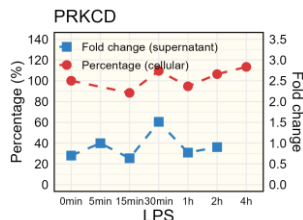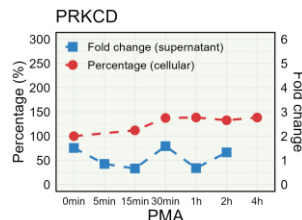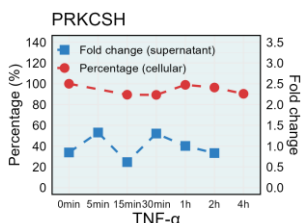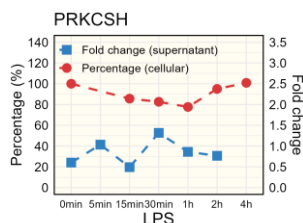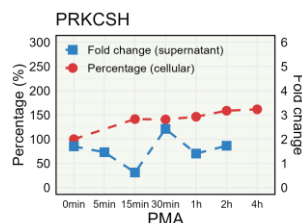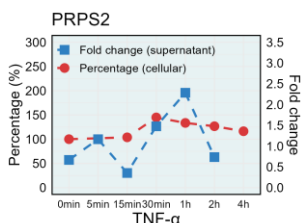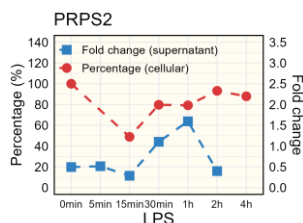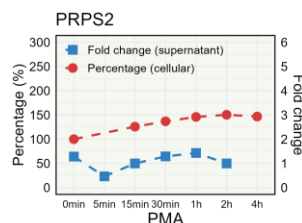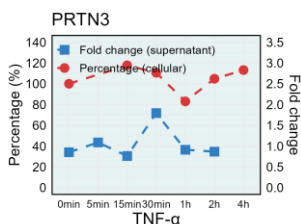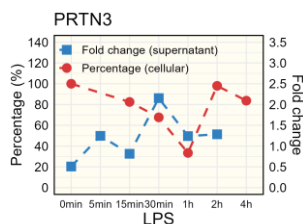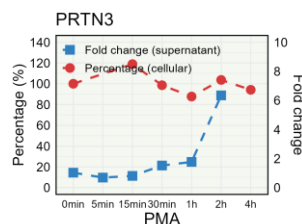

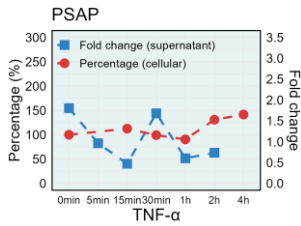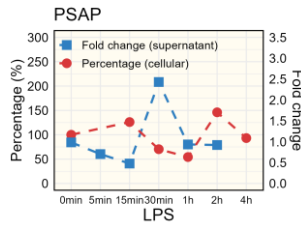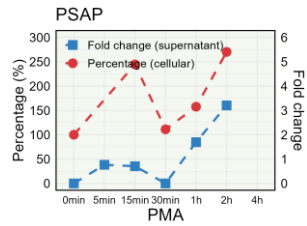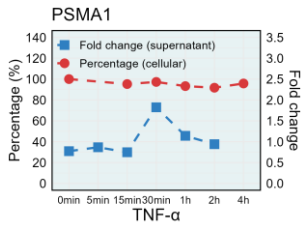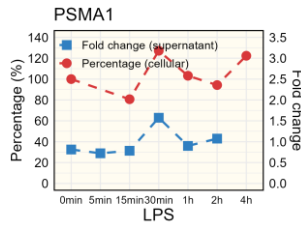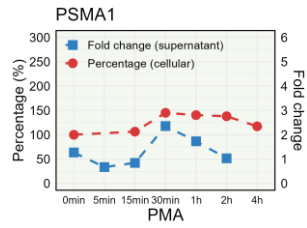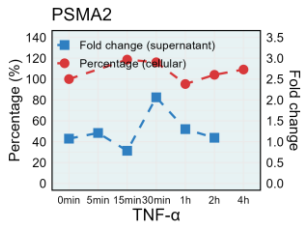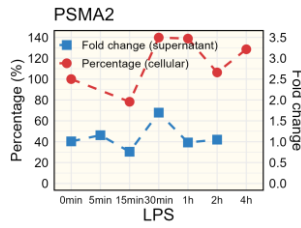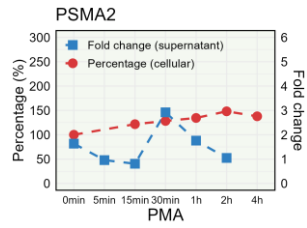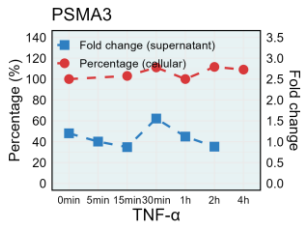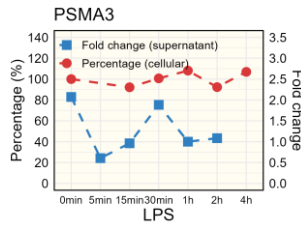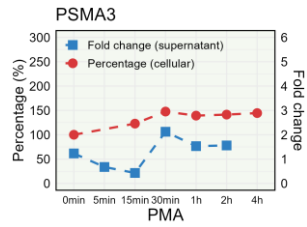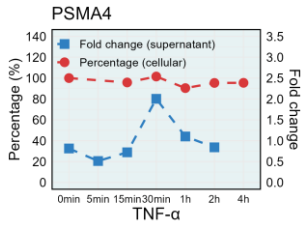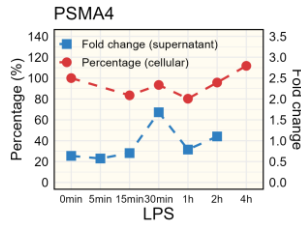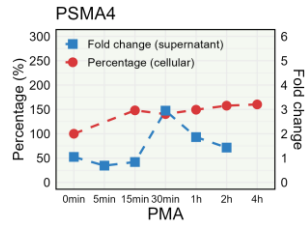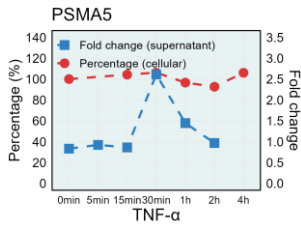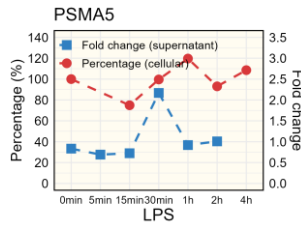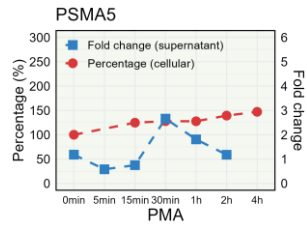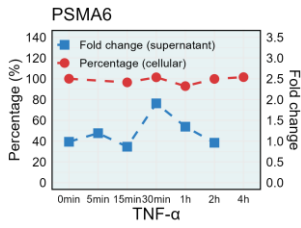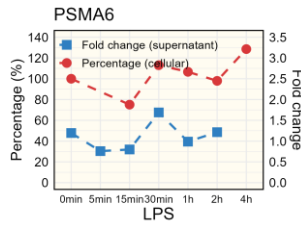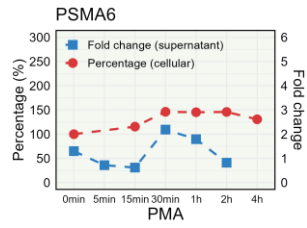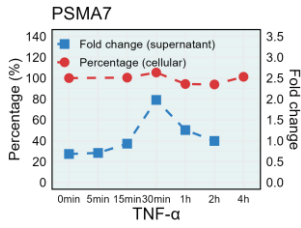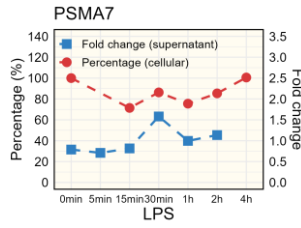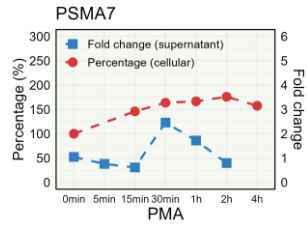

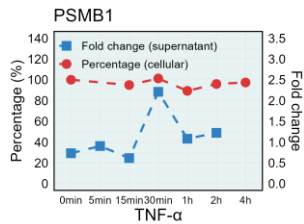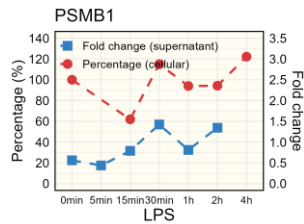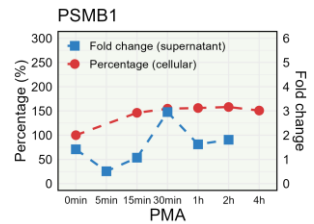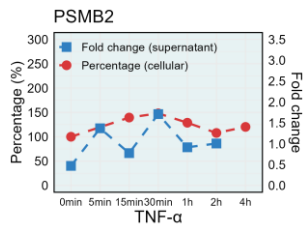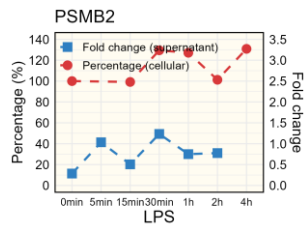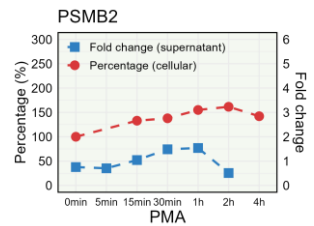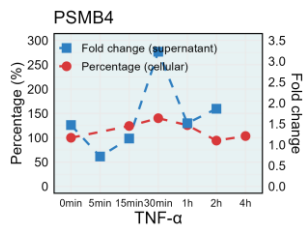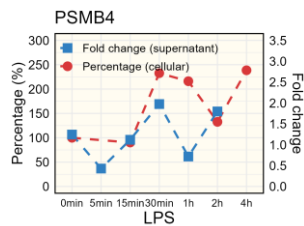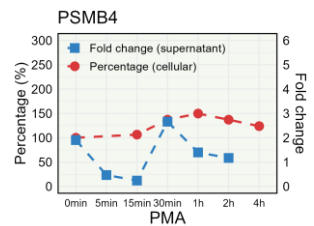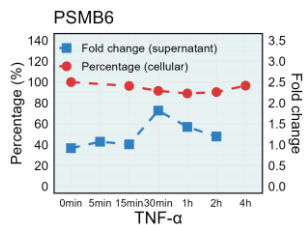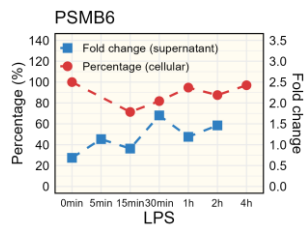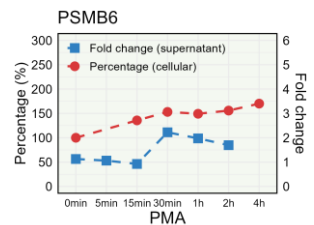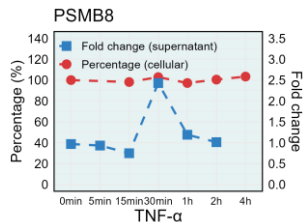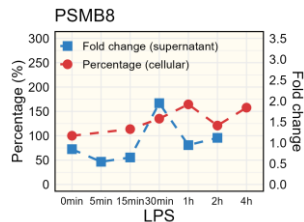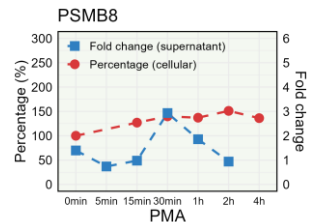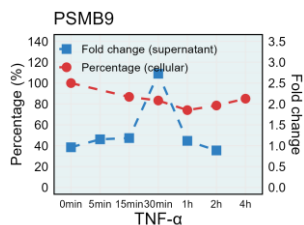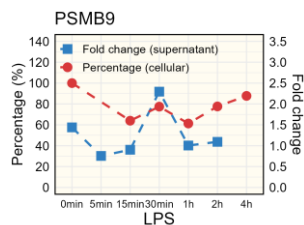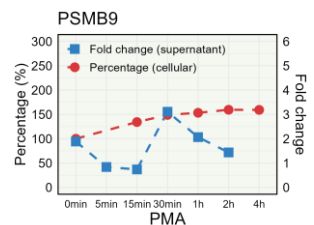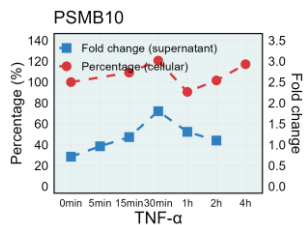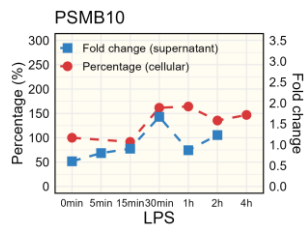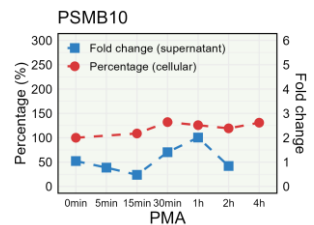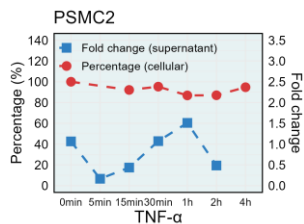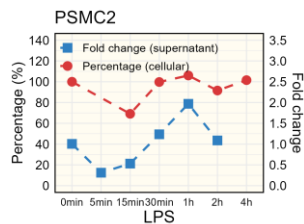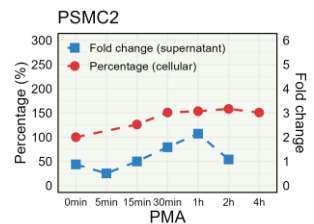

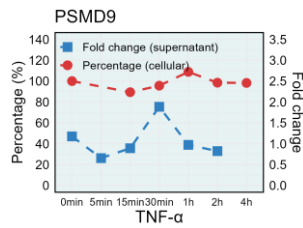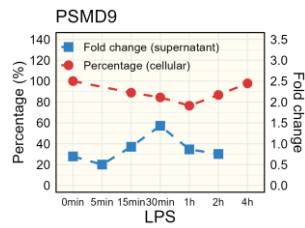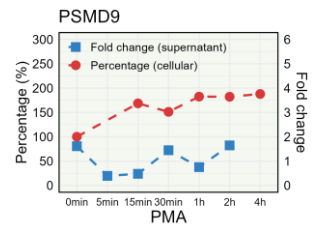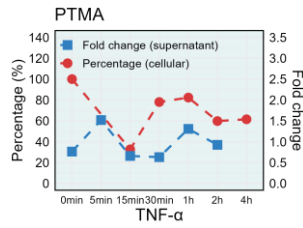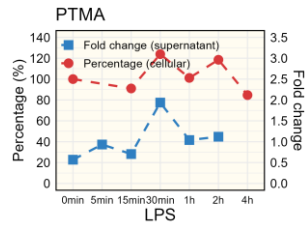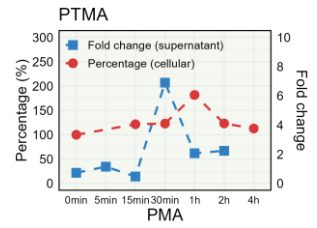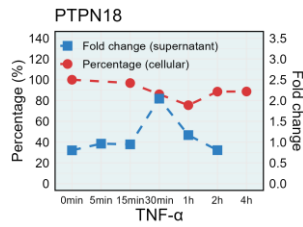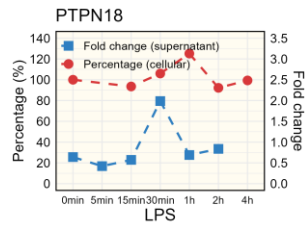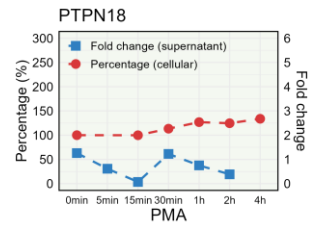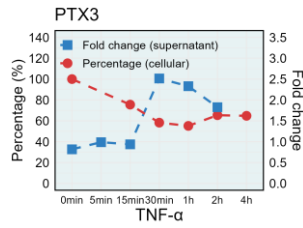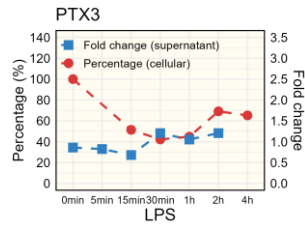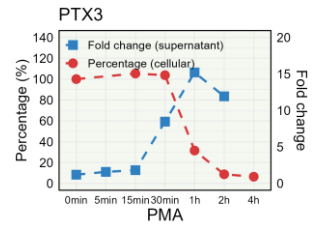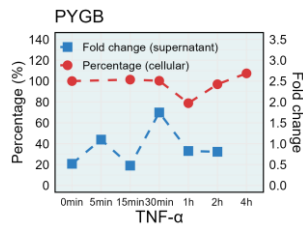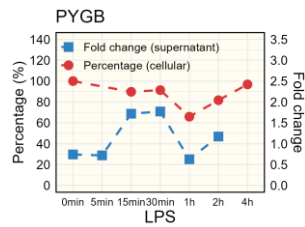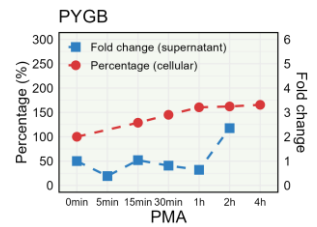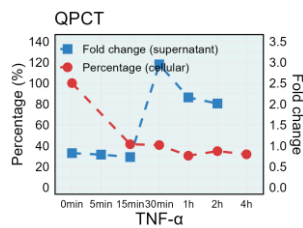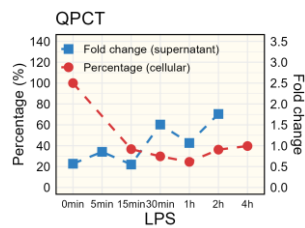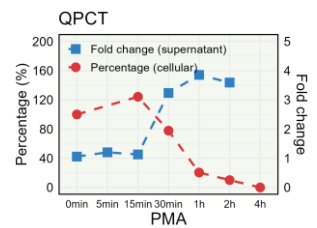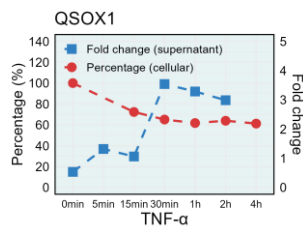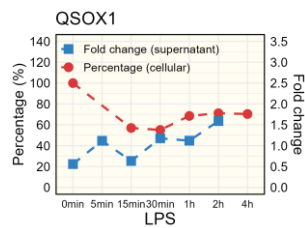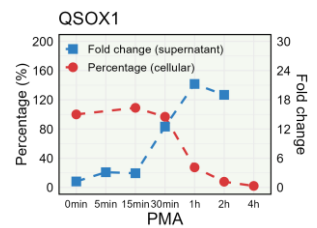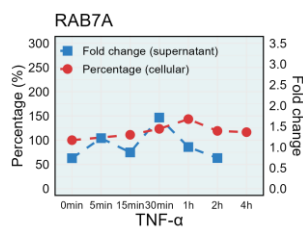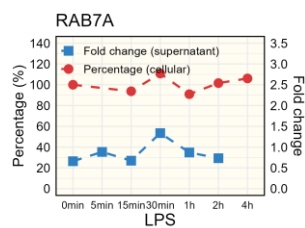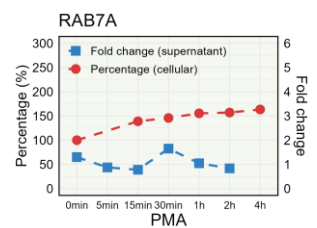

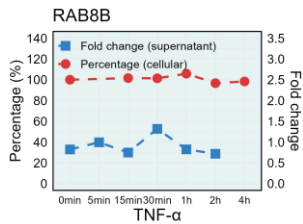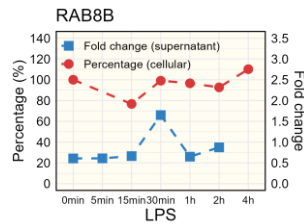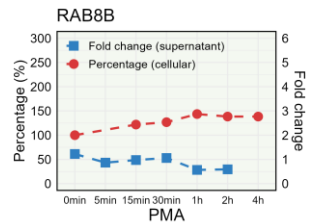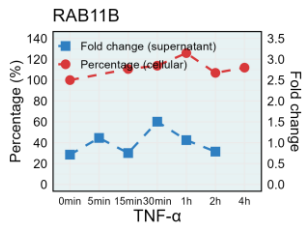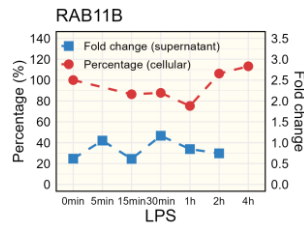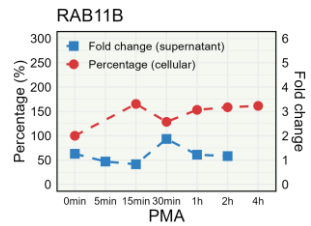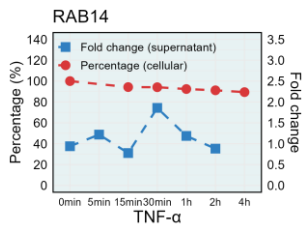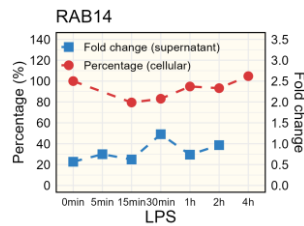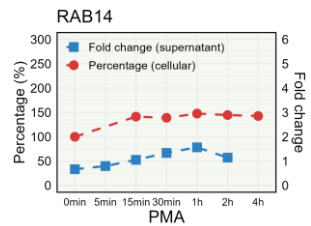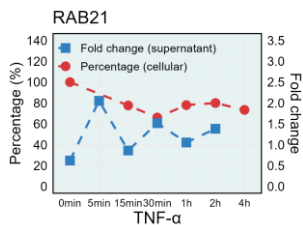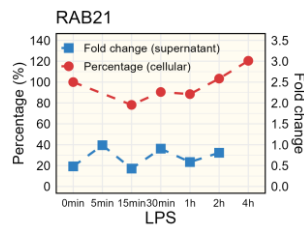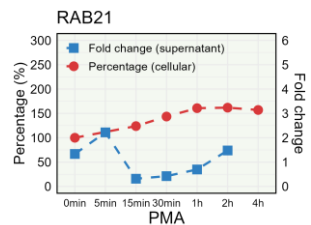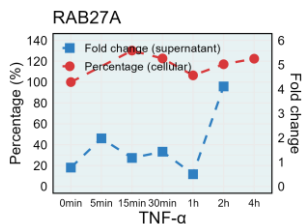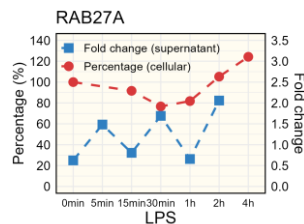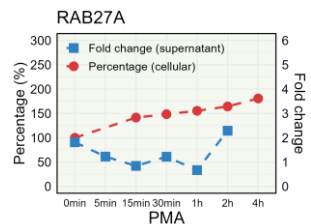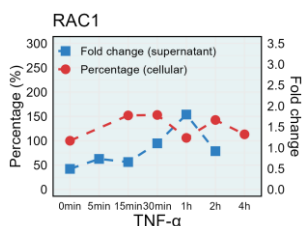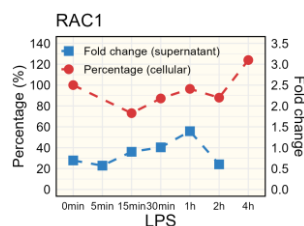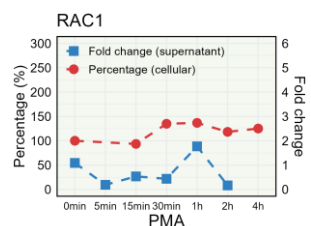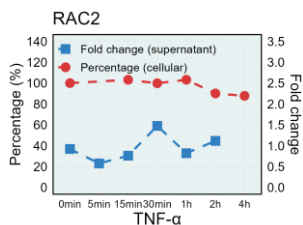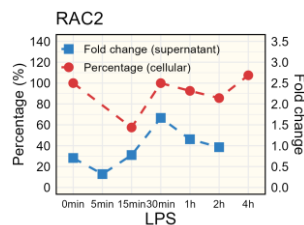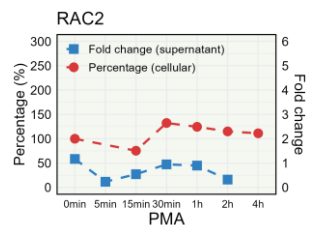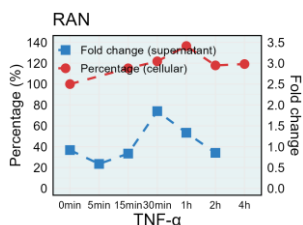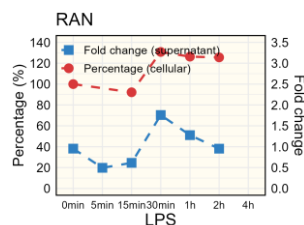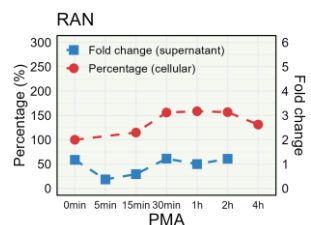

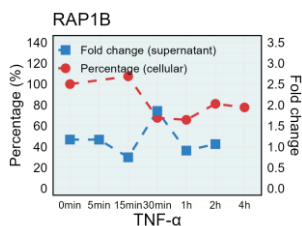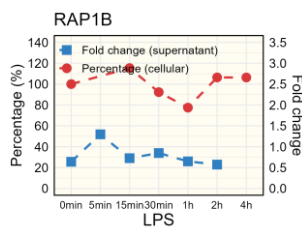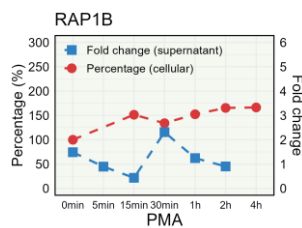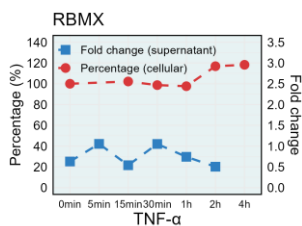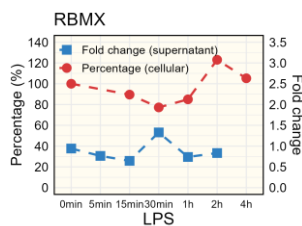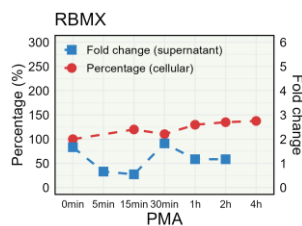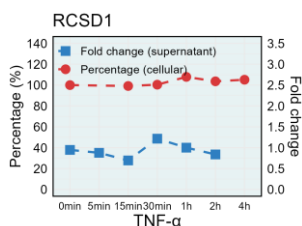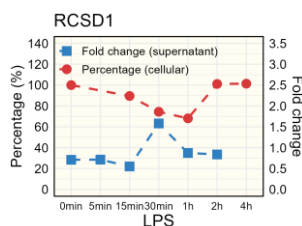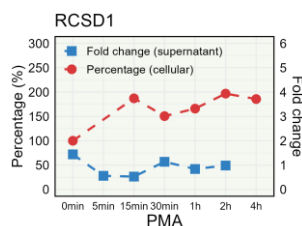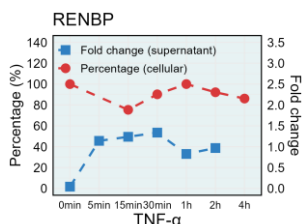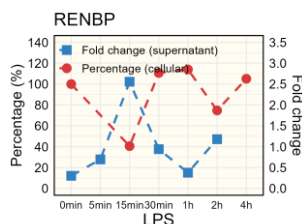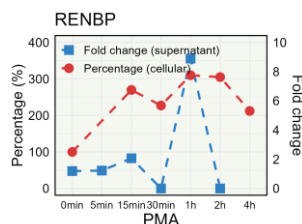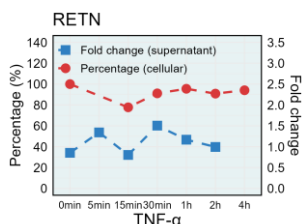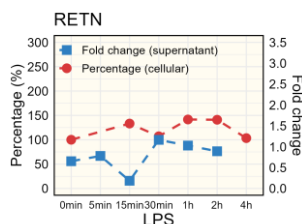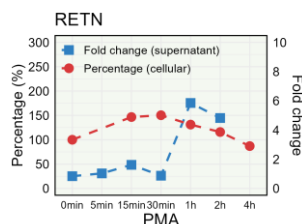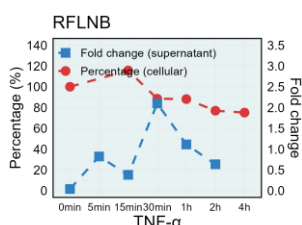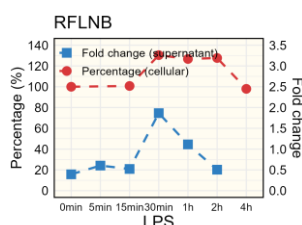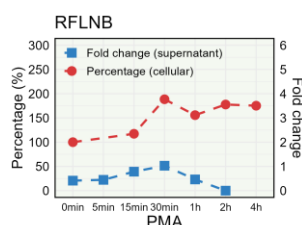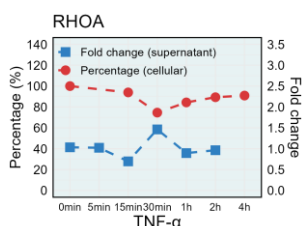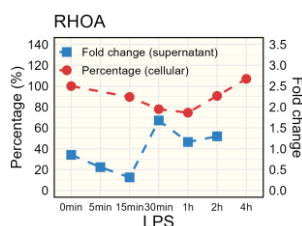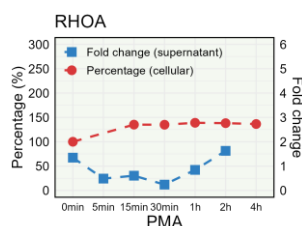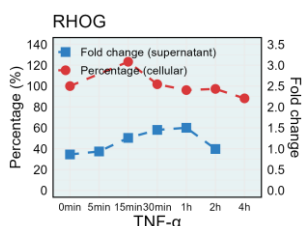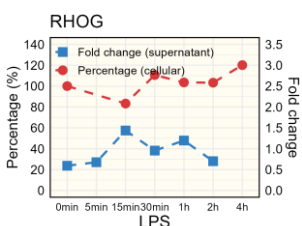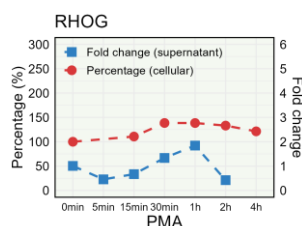

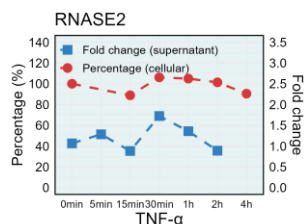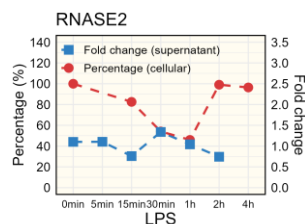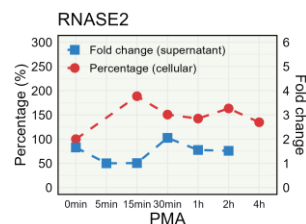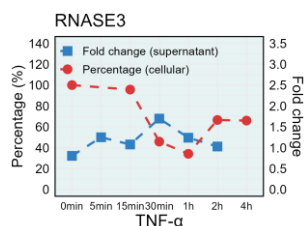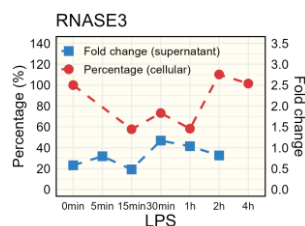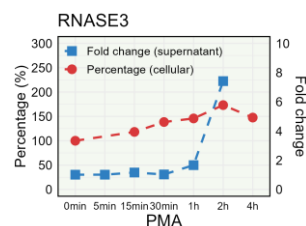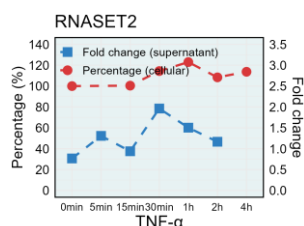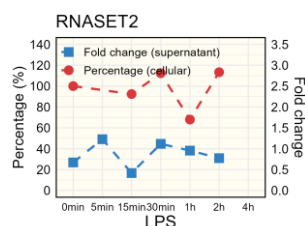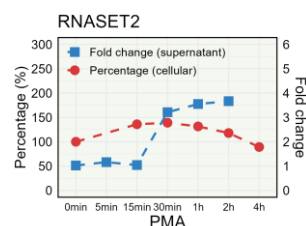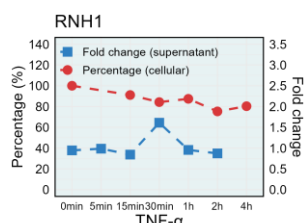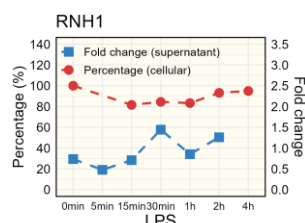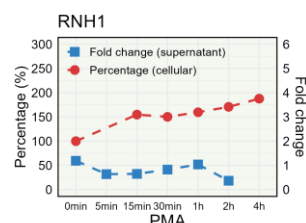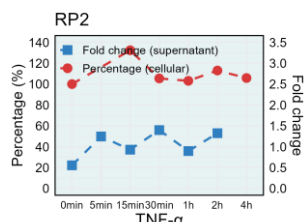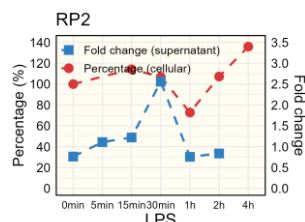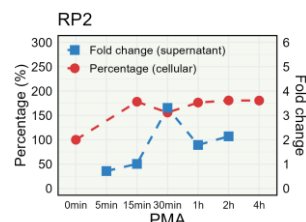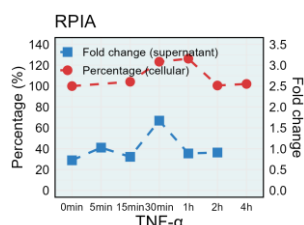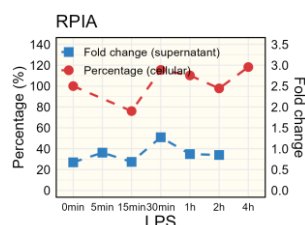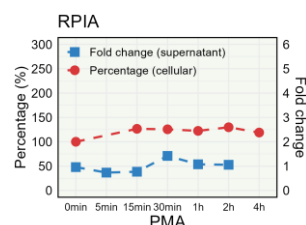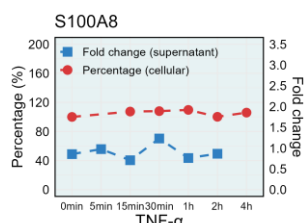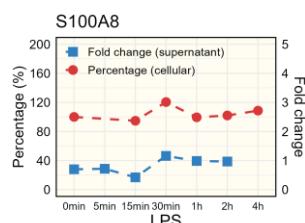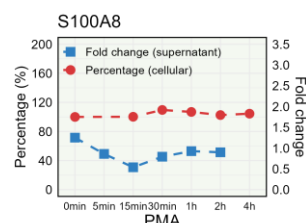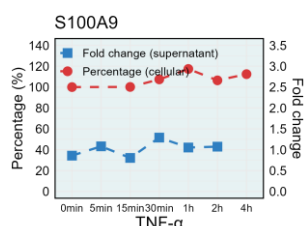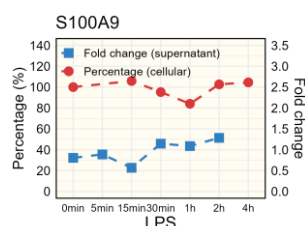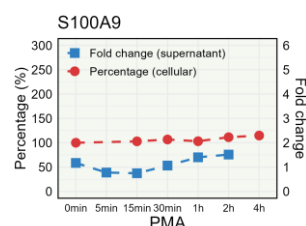

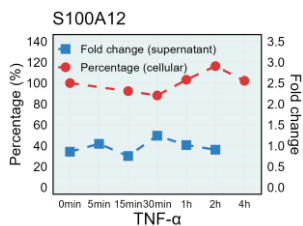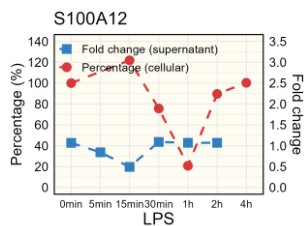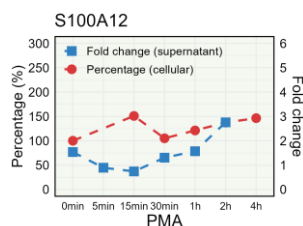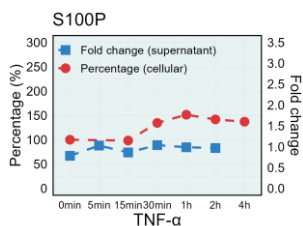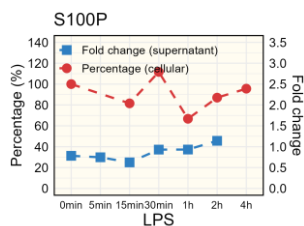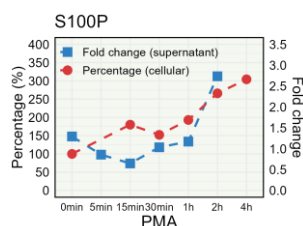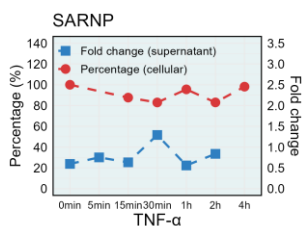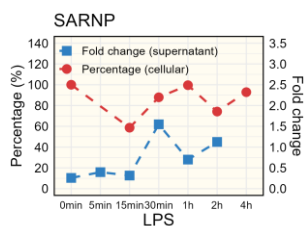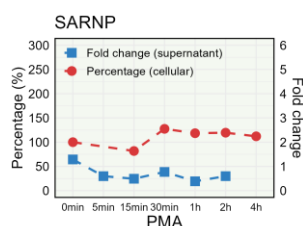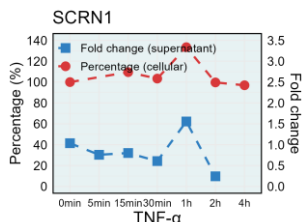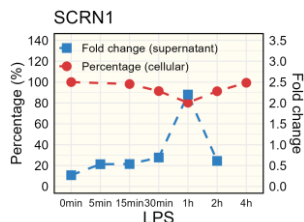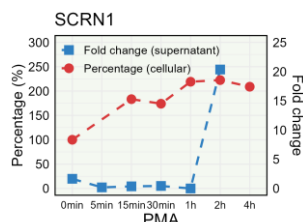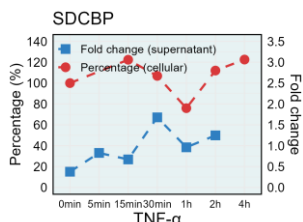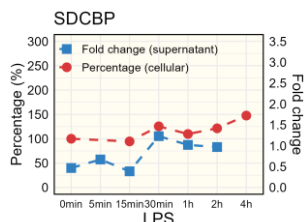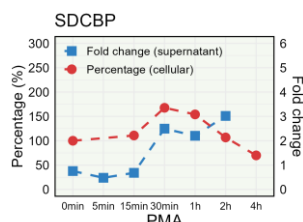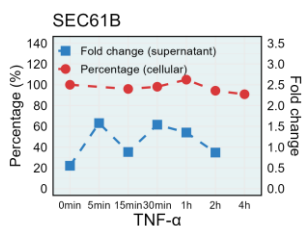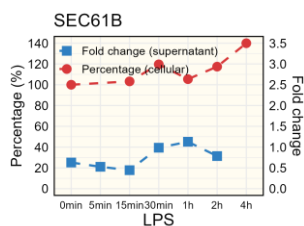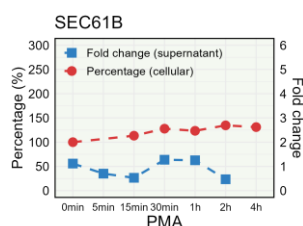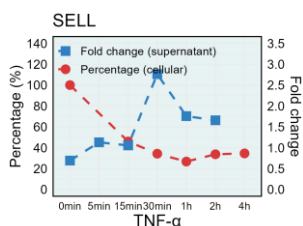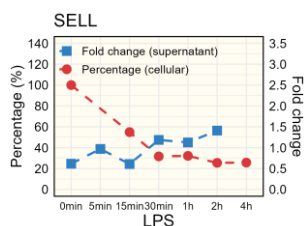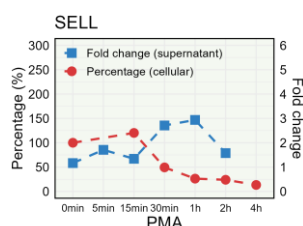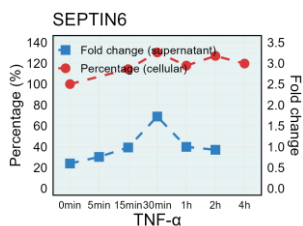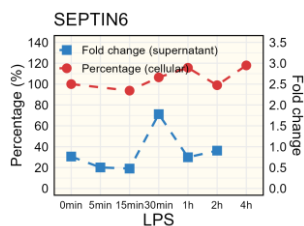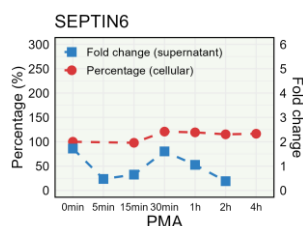

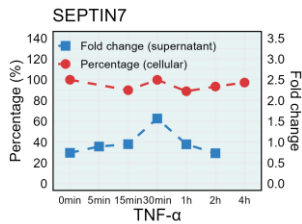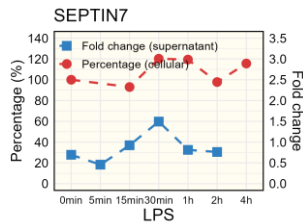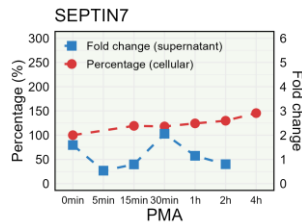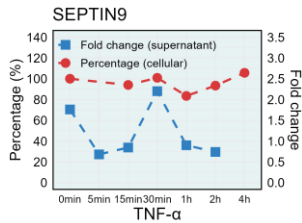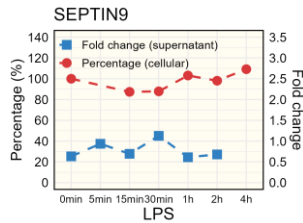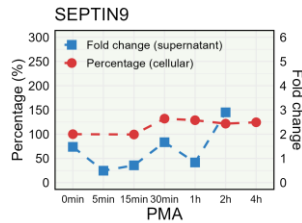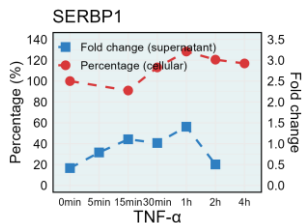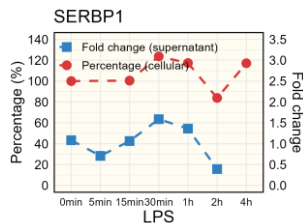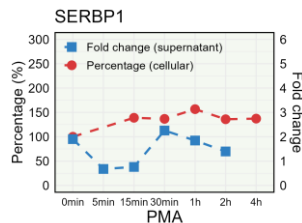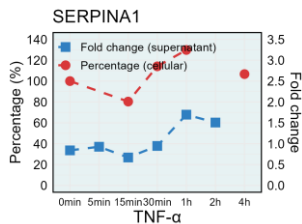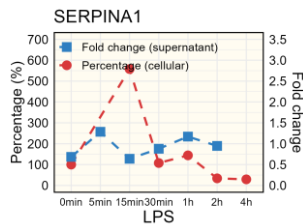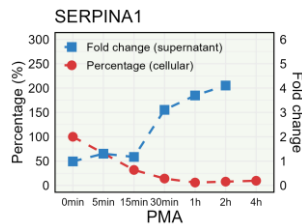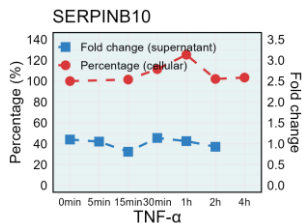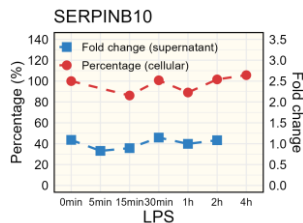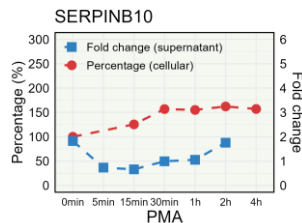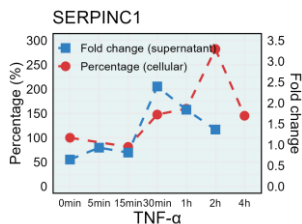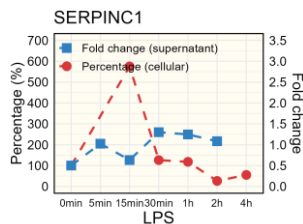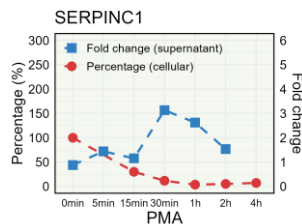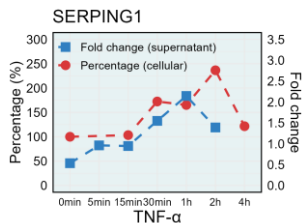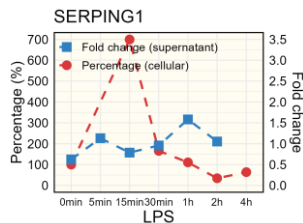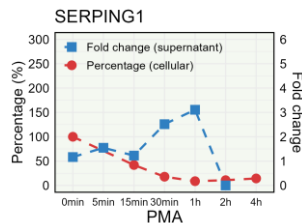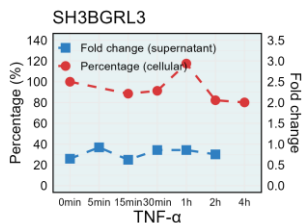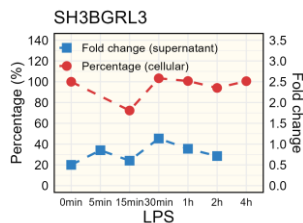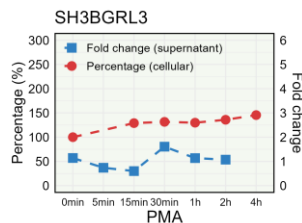

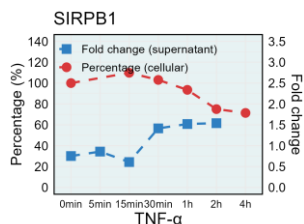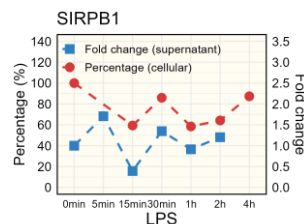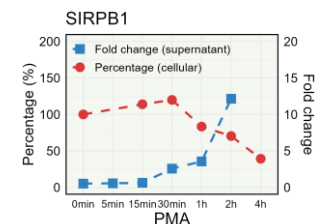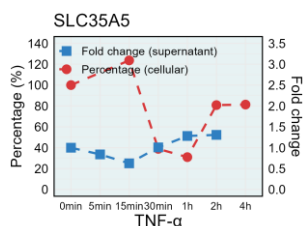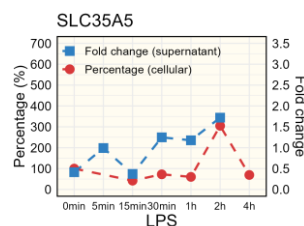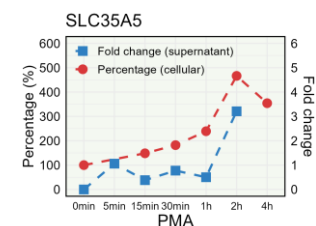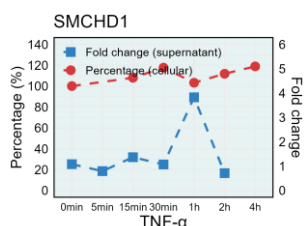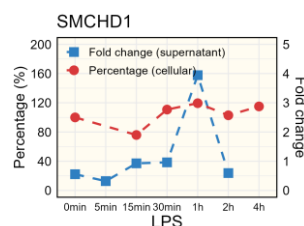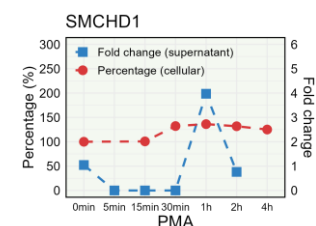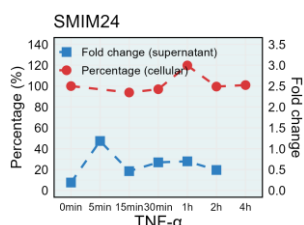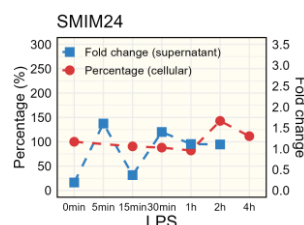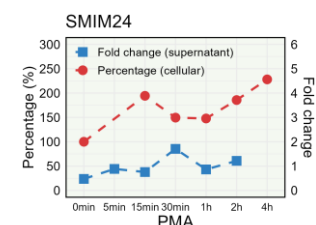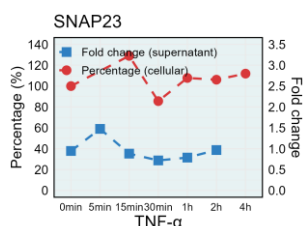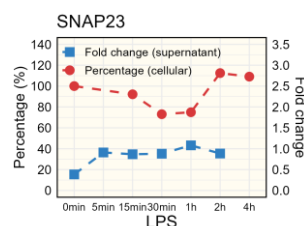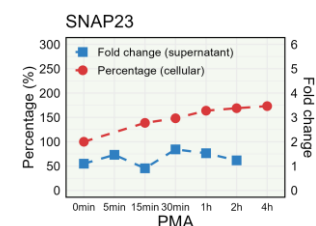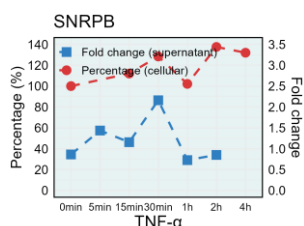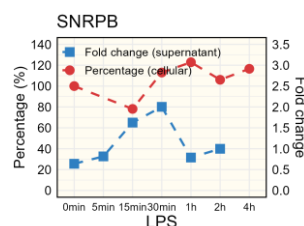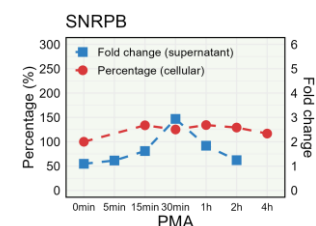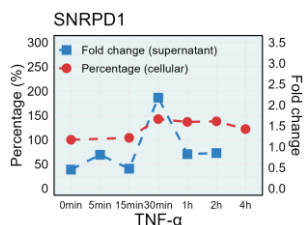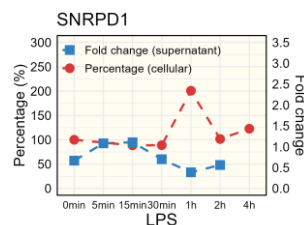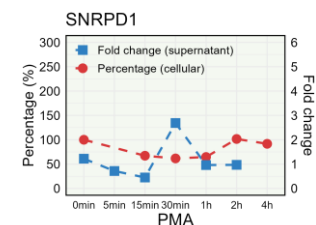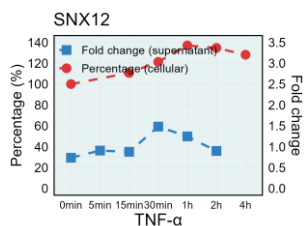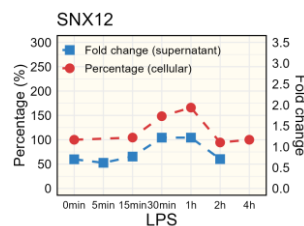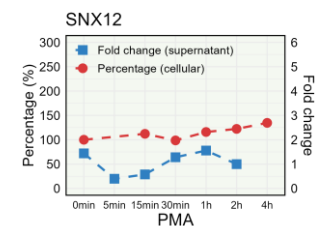

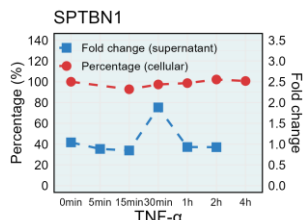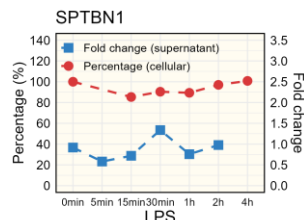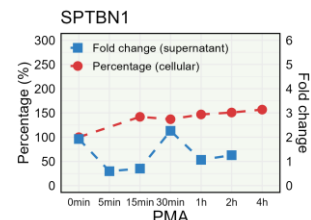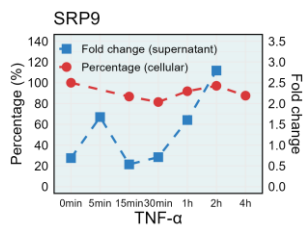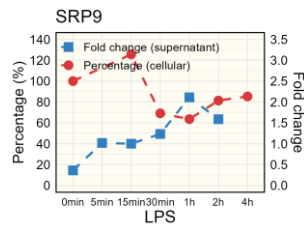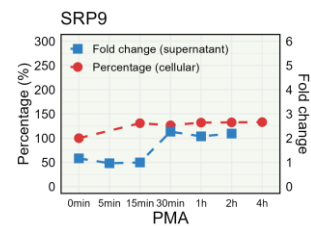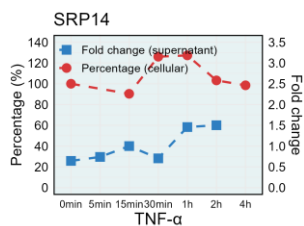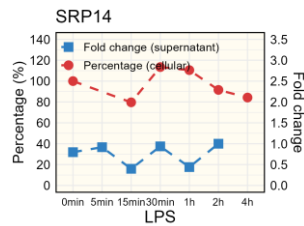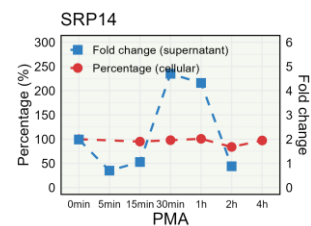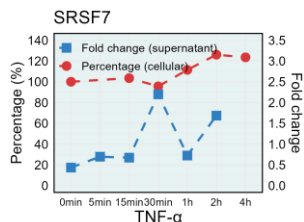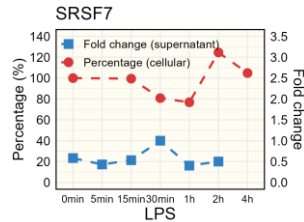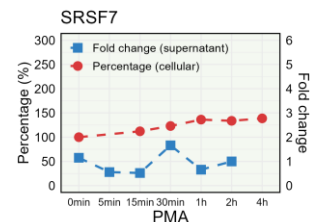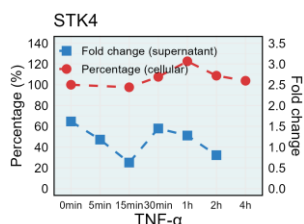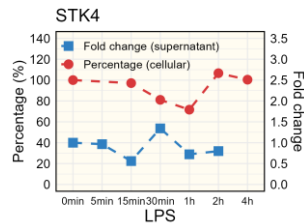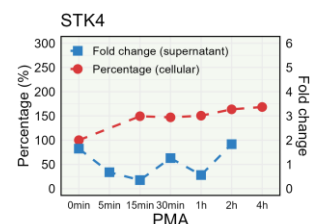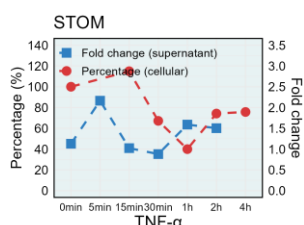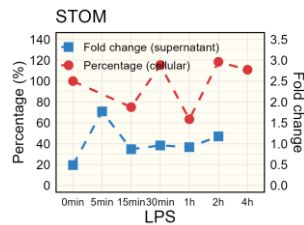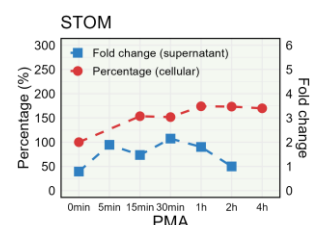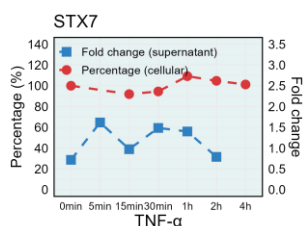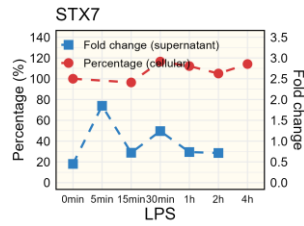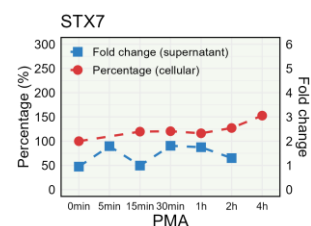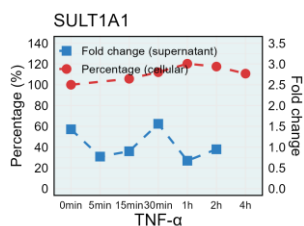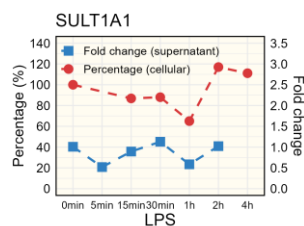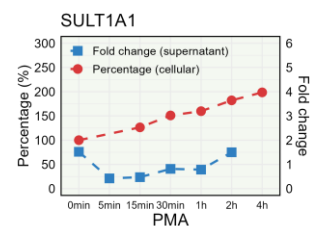

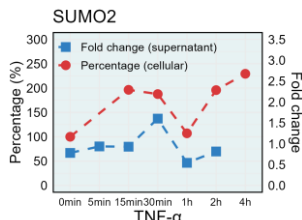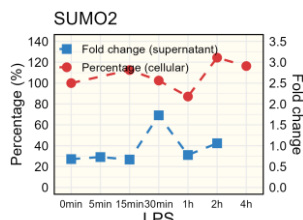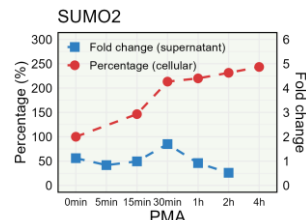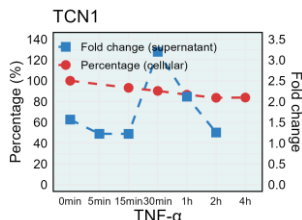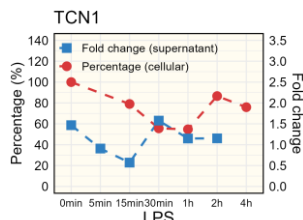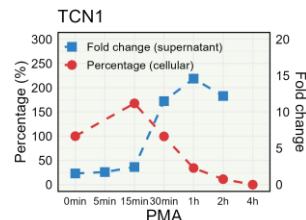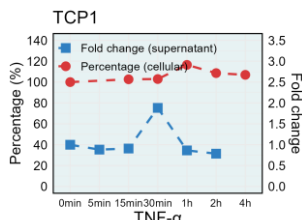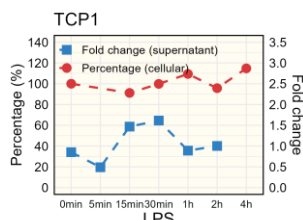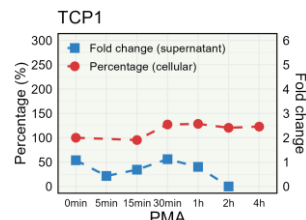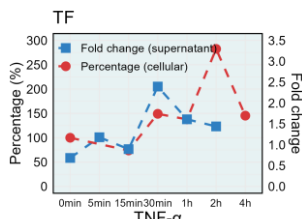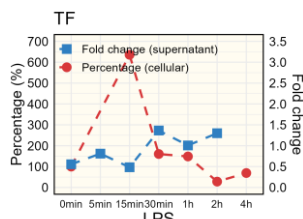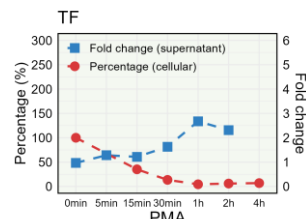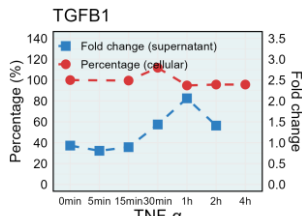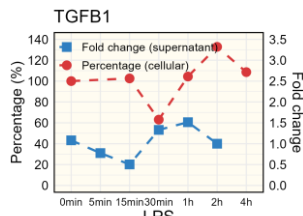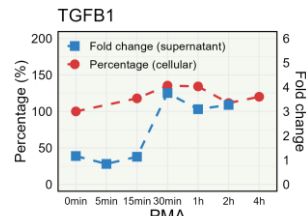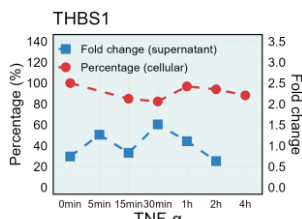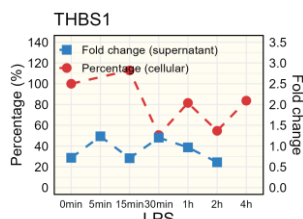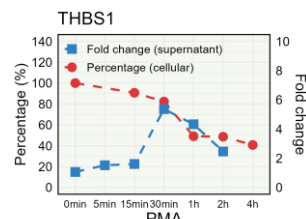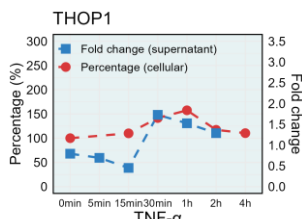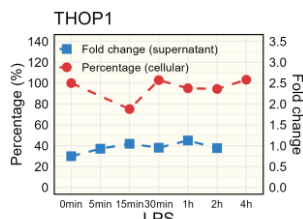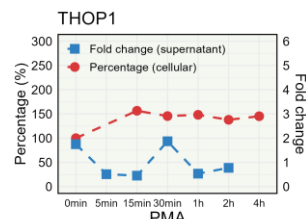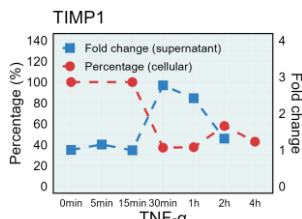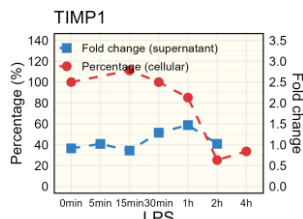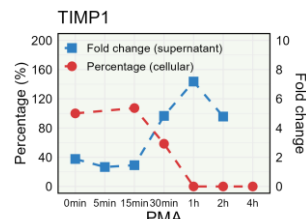

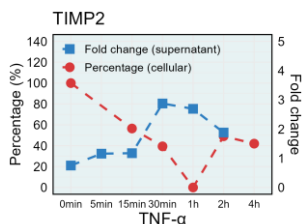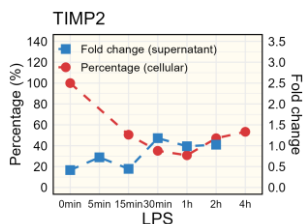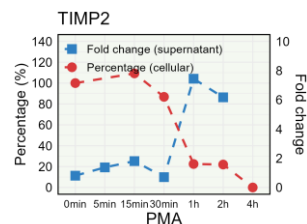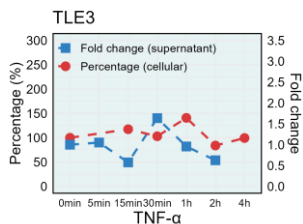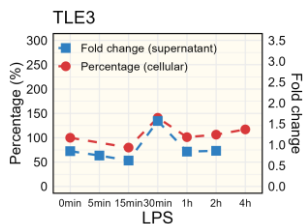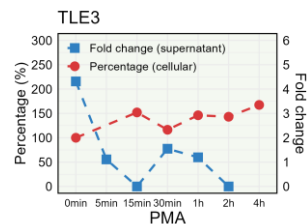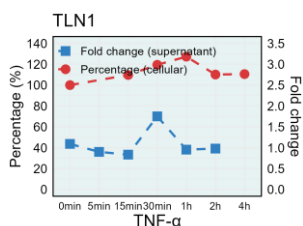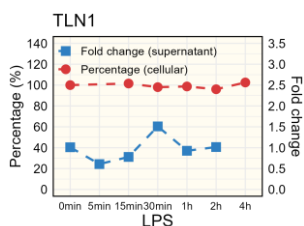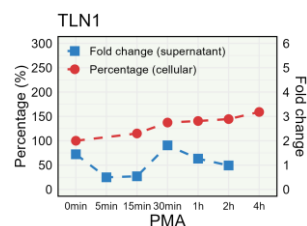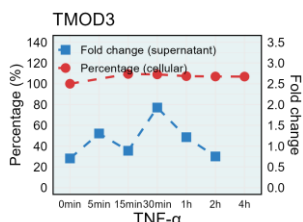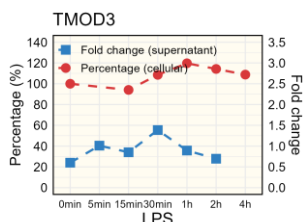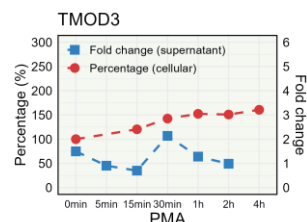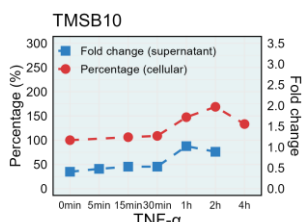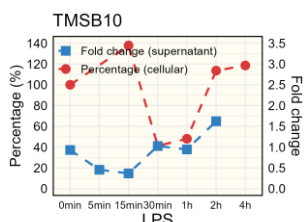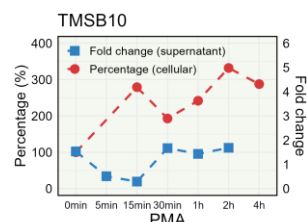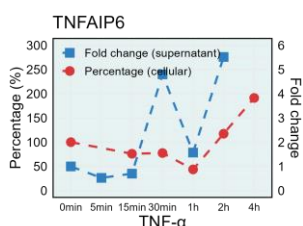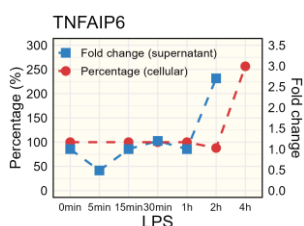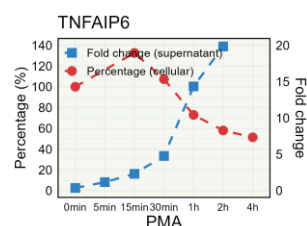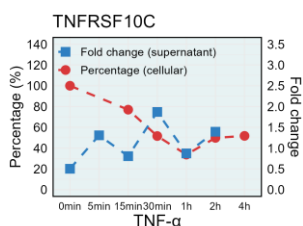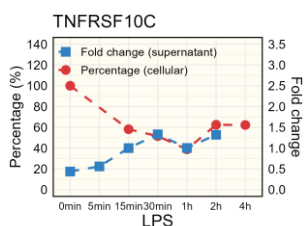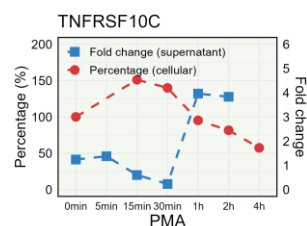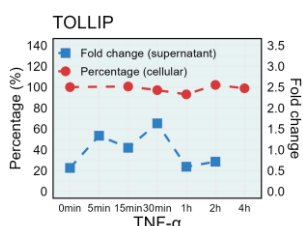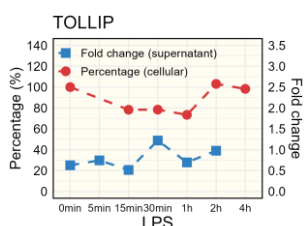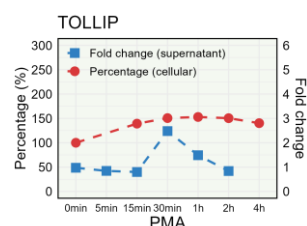

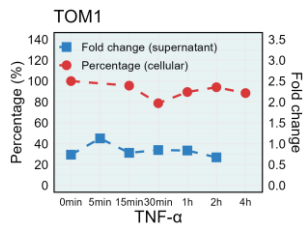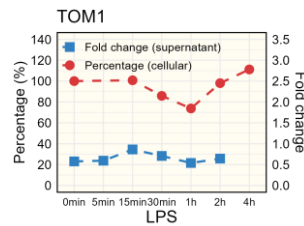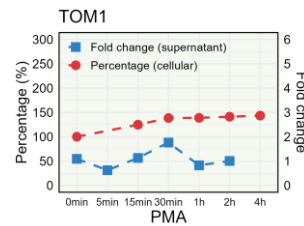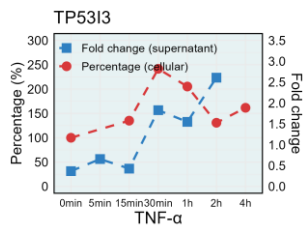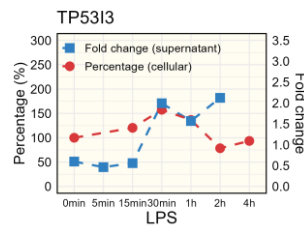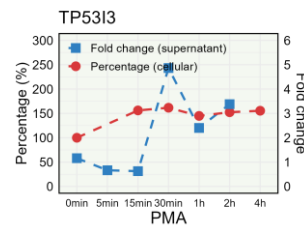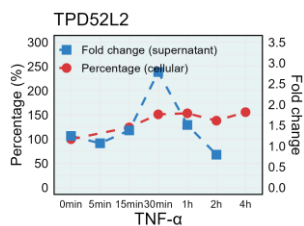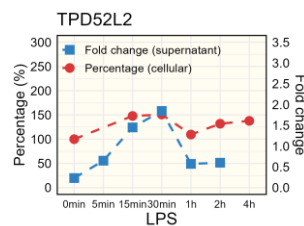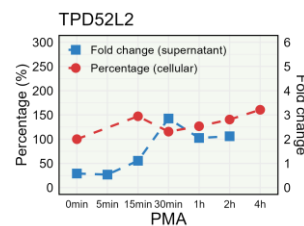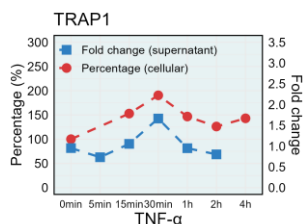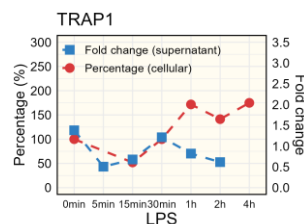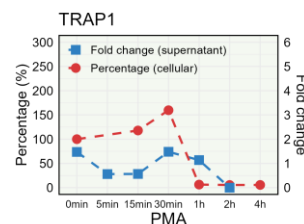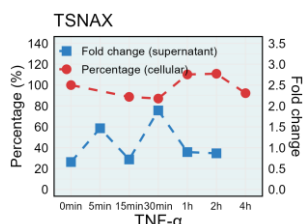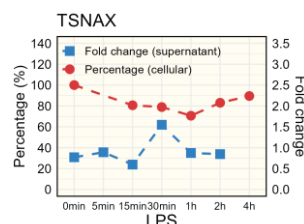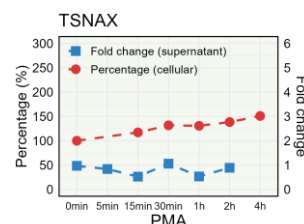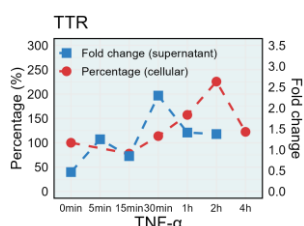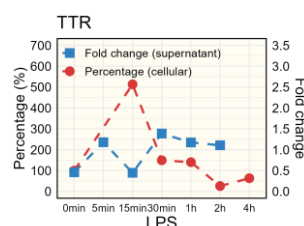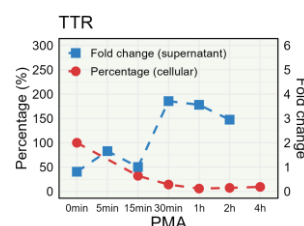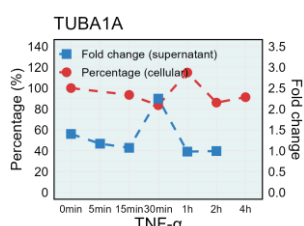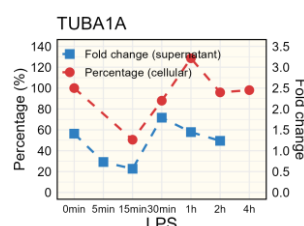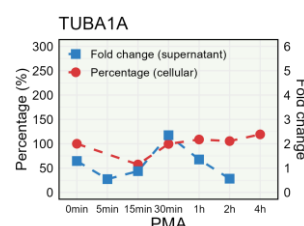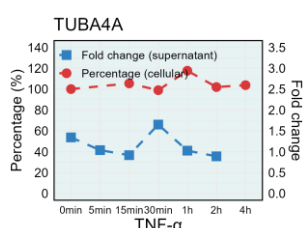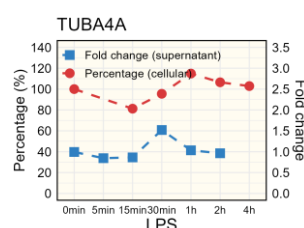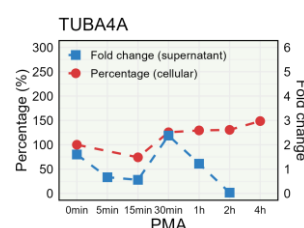

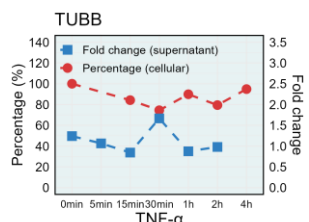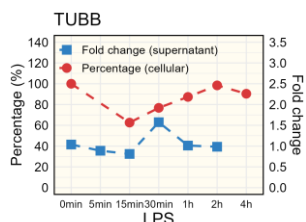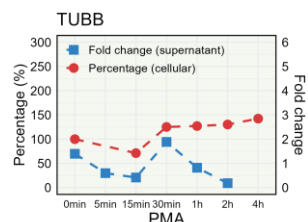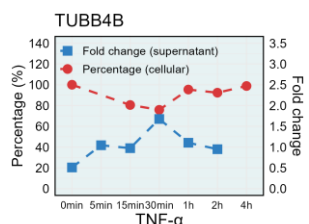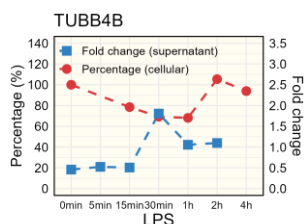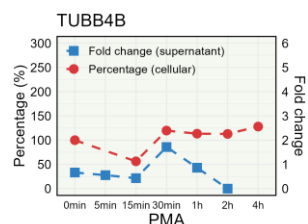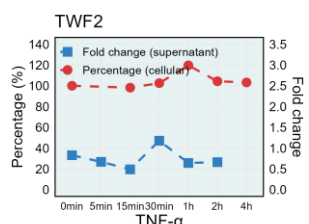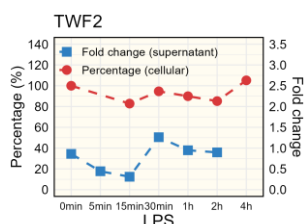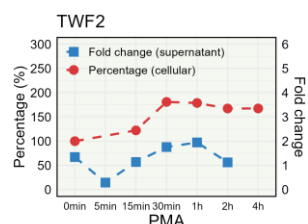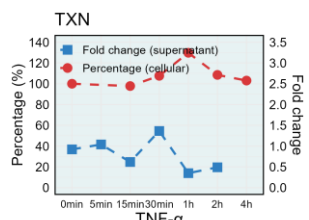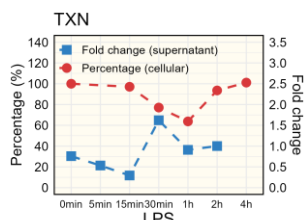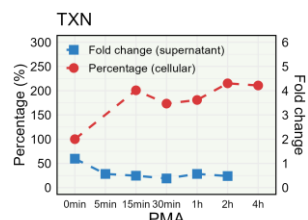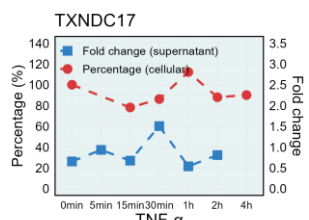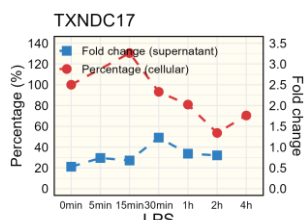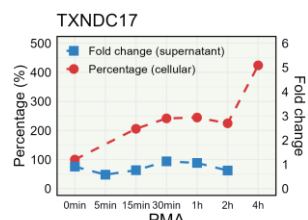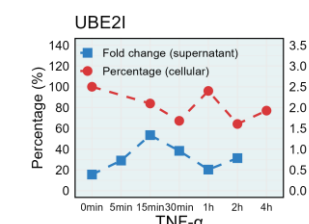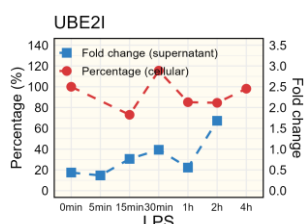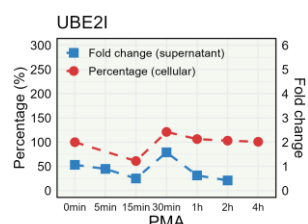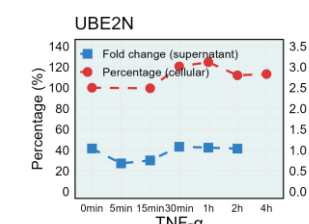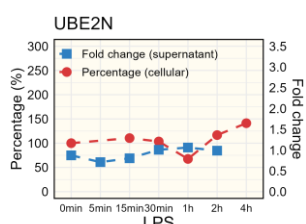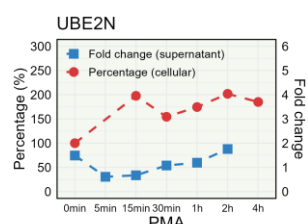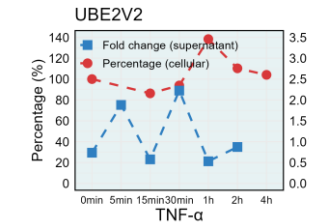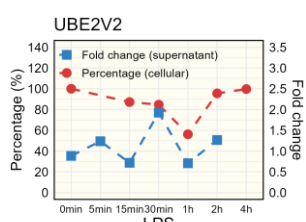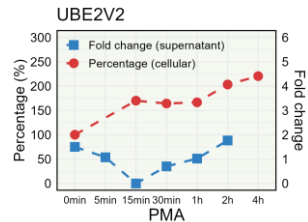

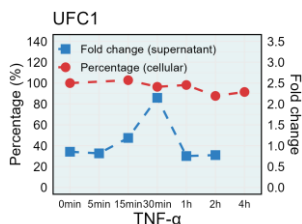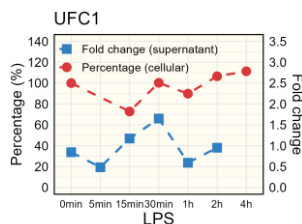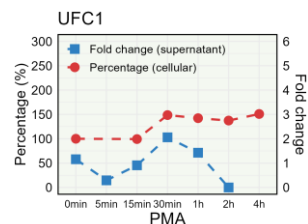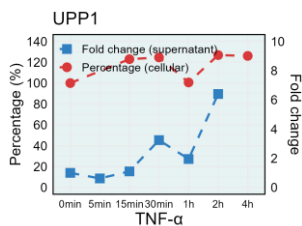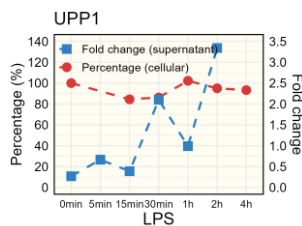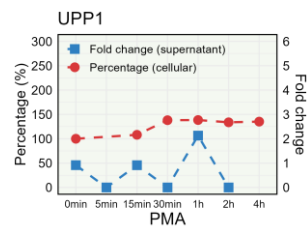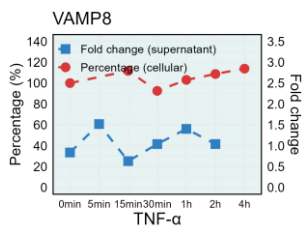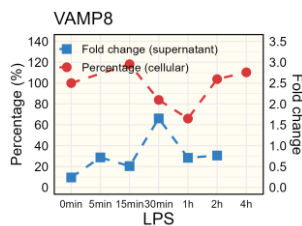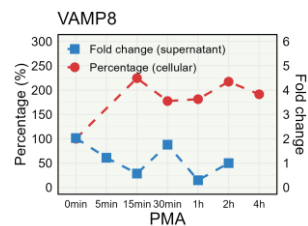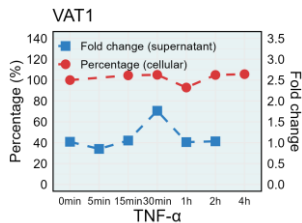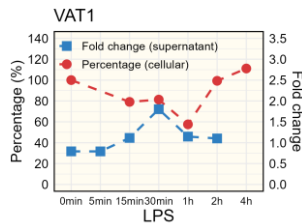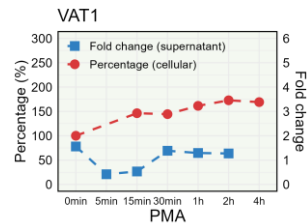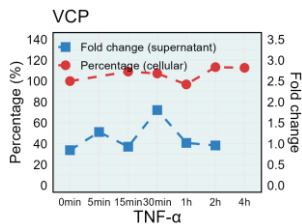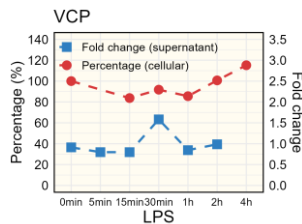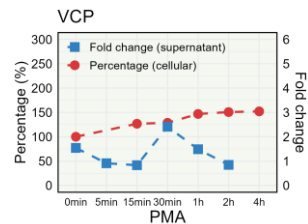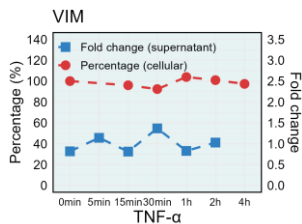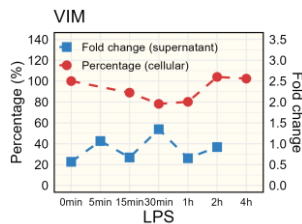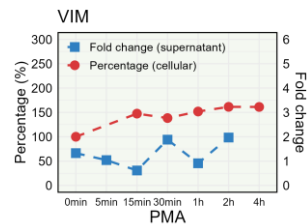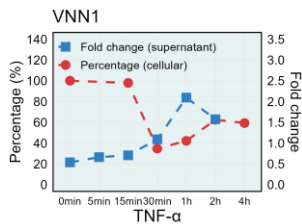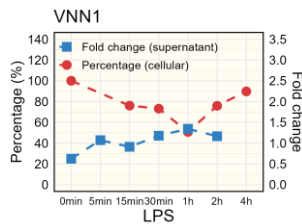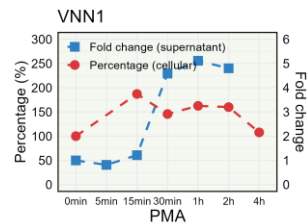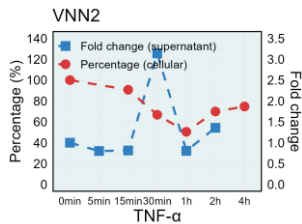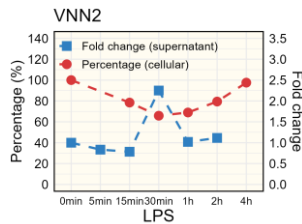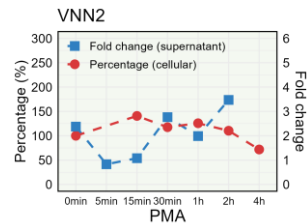

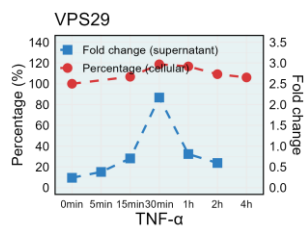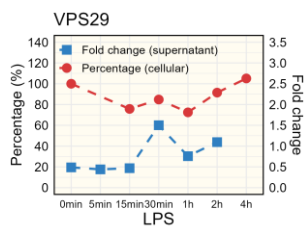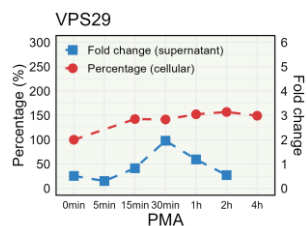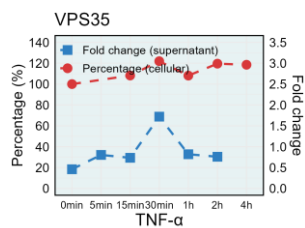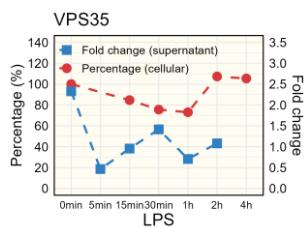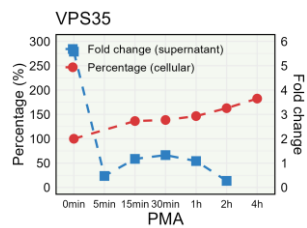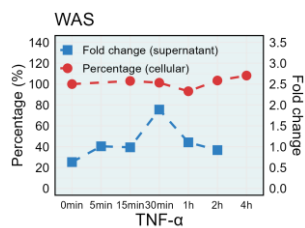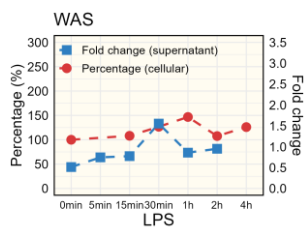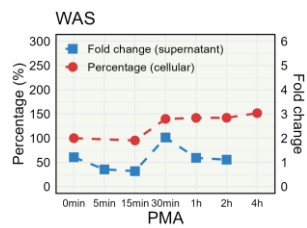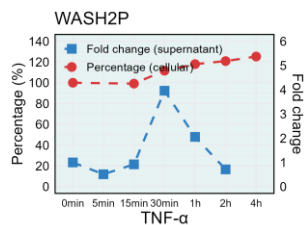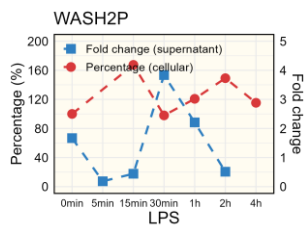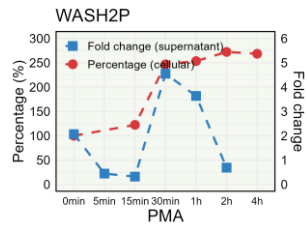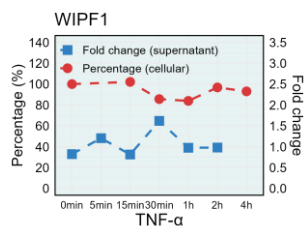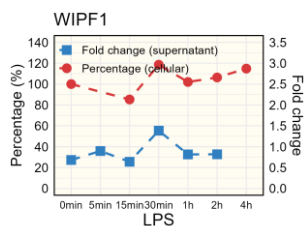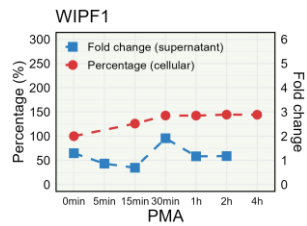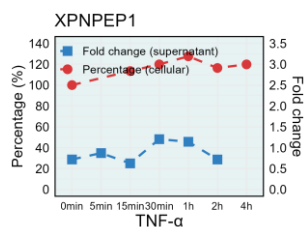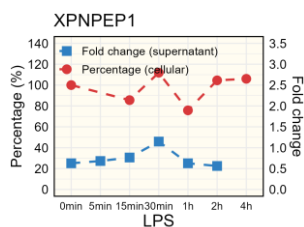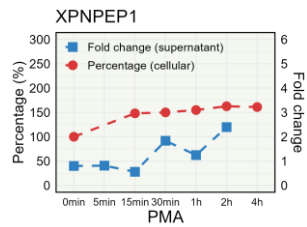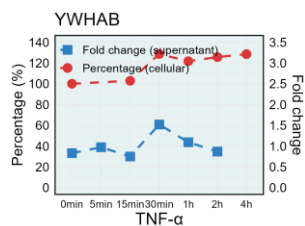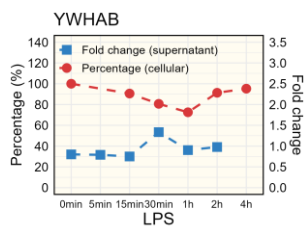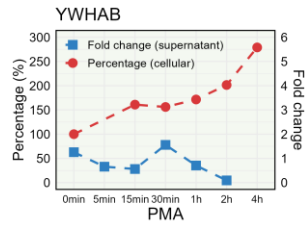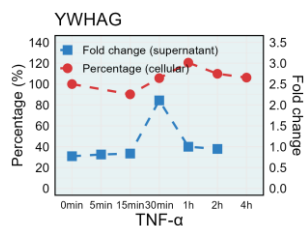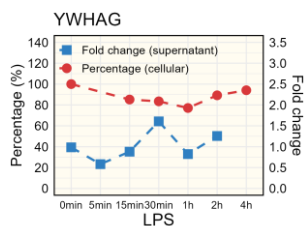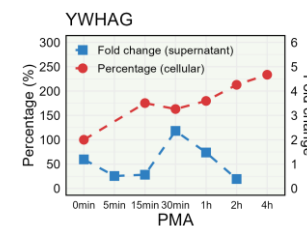

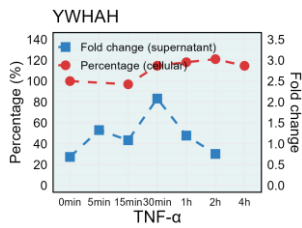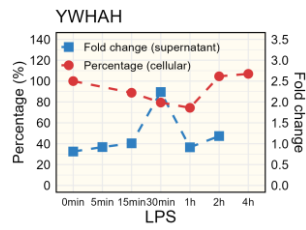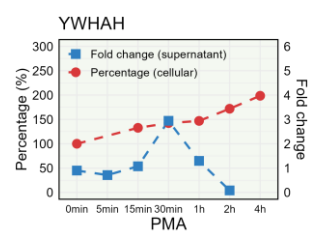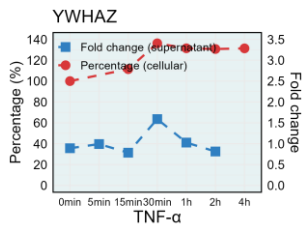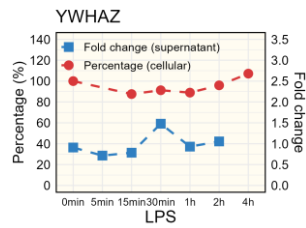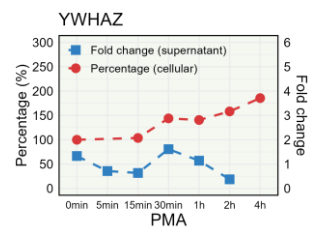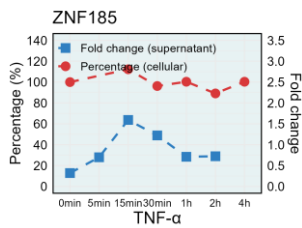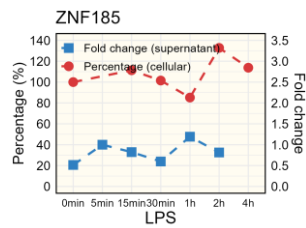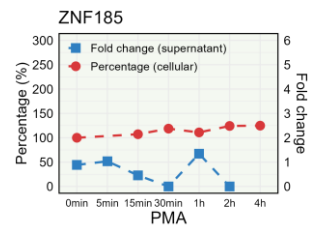

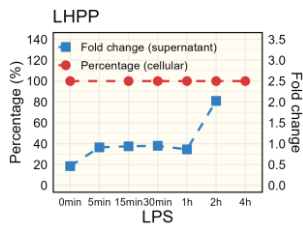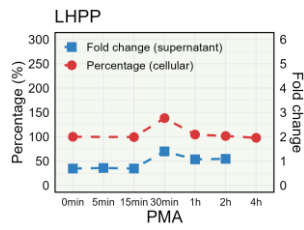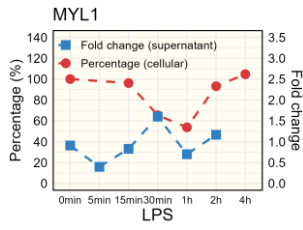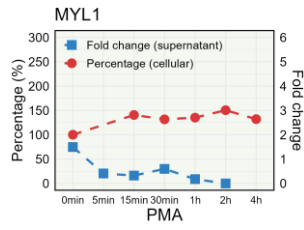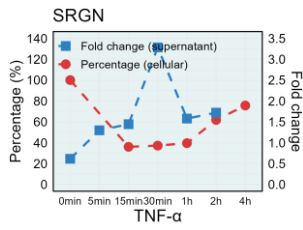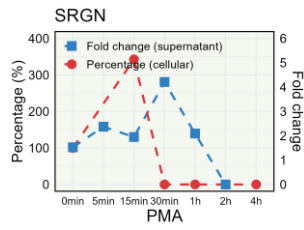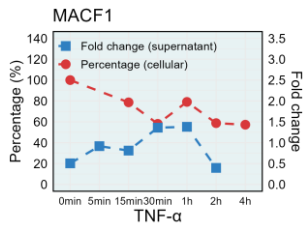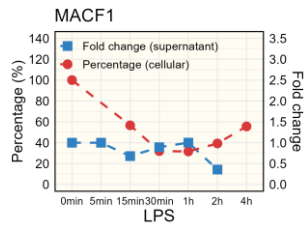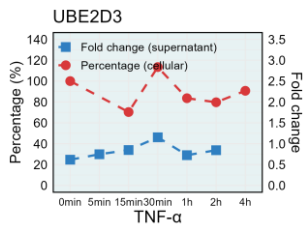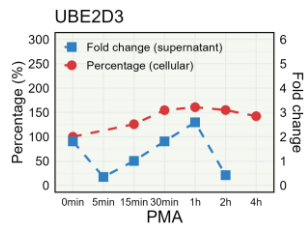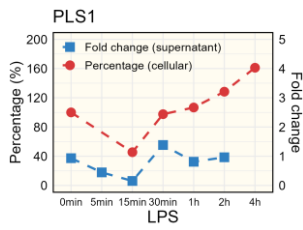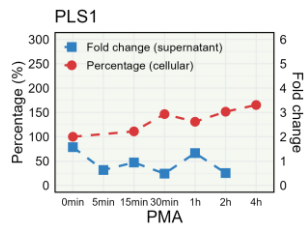

Supplement: Supplemental Dataset [file mmc10.pdf]
