# Supplementary material for: Proteomic Portrait of Degranulation Program in Human Circulating Neutrophils Upon Multi-Inflammatory and Infectious Activation
Source: Mol Cell Proteomics. 2025 Sep 29;25(1):101078. doi: 10.1016/j.mcpro.2025.101078 (PMC12804025; doi:10.1016/j.mcpro.2025.101078)
Supplement: Supplemental Material [file mmc11.docx]

**Supplemental Material**

Proteomic portrait of degranulation program in human circulating neutrophils upon multi-inflammatory and infectious activation

Ying Hua^1^, Ziqi Zhou^1^, Can Zhang^1^, Hai Fang^2^, Mingxing Wu^3^, Zhiyong Chen^4^, Xin Ku^1*^, Wei Yan^1*^

^1^ Key Laboratory of Systems Biomedicine (Ministry of Education), Shanghai Center for Systems Biomedicine, State Key Laboratory of Medical Genomics, Shanghai Jiao Tong University, 800 Dongchuan Road, Shanghai 200240, China.

^2^ Shanghai Institute of Hematology, State Key Laboratory of Medical Genomics, National Research Center for Translational Medicine at Shanghai, Ruijin Hospital, Shanghai Jiao Tong University School of Medicine, Shanghai 200025, China.

^3^ State Key Laboratory of Ophthalmology, Zhongshan Ophthalmic Center, Sun Yat-sen University, Guangdong Provincial Key Laboratory of Ophthalmology and Vision Science, Guangdong Provincial Clinical Res CN, Guangdong, China.

^4^ Department of Rheumatology & Immunology, Shanghai Jiao Tong University School of Medicine Affiliated Sixth People’s Hospital, 600 Yishan Road, Shanghai 200233, China.

*Address correspondence to: Wei Yan, E-mail: [weiyan_99@uwalumni.com](mailto:weiyan_99@uwalumni.com), [weiyan@sjtu.edu.cn](mailto:weiyan@sjtu.edu.cn); Xin Ku, E-mail: [xinku@sjtu.edu.cn](mailto:xinku@sjtu.edu.cn)

**Supplemental Figure legends**

**Supplemental Figure 1. The isolation of human blood neutrophils.**

**(A)** Separation of neutrophils from human blood by density gradient centrifugation method using Polymorphprep solution. **(B)** Flow cytometry showing high neutrophil purity (>95 %) after isolation. **(C)** Neutrophils are on the second buffy coat layer. The Wright-Giemsa stain revealed 2-5 segments in neutrophil nuclear morphology. **(D)** Line plot showing the temporal expression patterns of canonical granule proteins MMP9, MMP8 and PGLYRP1 in the Poly(I:C) treated neutrophils.

**Supplemental Figure 2. Stimulus-induced degranulation profiles in activated neutrophils**

Stimulus-specific degranulation signatures. Complete list of granule proteins released under: TNF-α treatment (148 proteins); LPS treatment (81 proteins); PMA treatment (147 proteins).

**Supplemental Figure 3. Temporal expression patterns of granule proteins in TNF-α, LPS and PMA stimulated neutrophils.**

**(A)** Line graph showing the cellular and supernatant expression patterns of granule proteins QPCT and OLFM4 in TNF-α condition (blue background), LPS condition (yellow background), and PMA condition (green background). **(B)** AZU1 and ELANE expression in the cellular proteome and supernatant proteome. **(C)** S100A9 expression in the cellular proteome and supernatant proteome.

**Supplemental Figure 4. Cellular and secreted patterns of classical marker proteins in primary granules, secondary granules, tertiary granules and secretory vesicles.**

1. Line graph showing the cellular and supernatant expression patterns of primary granule marker proteins AZU1, ELANE, MPO and PRTN3 in PMA condition. (Left: cellular patterns; Right: secreted patterns)
2. Line graph showing the cellular and supernatant expression patterns of secondary granule marker proteins LCN2, LTF, CHI3L1, CRISP3, MMP8, OLFM4, TCN1 and LRG1 in PMA condition.
3. Line graph showing the cellular and supernatant expression patterns of tertiary granule marker proteins FCN1, FGL2, MMP9, CAMP, CHIT1, CD93 and PGLYRP1 in PMA condition.
4. Line graph showing the cellular and supernatant expression patterns of secretory vesicles marker proteins FCGR3B, CD14 and CD11b in PMA condition.

**Supplemental Figure 5. Colocalization of QSOX1, TIMP2 with known granule marker protein.**

Line graph showing the cellular and supernatant expression patterns of granule proteins QSOX1 **(A)** and TIMP2 **(B)** in TNF-α condition (blue background), LPS condition (yellow background), and PMA condition (green background). **(C)** Confocal analysis revealed that QSOX1 colocalized with LTF abundant granules and MMP9 abundant granules. **(D)** Confocal analysis revealed that TIMP2 has a clear colocalization with LTF abundant granules and MMP9 abundant granules.

**Supplemental Figure 6. Secretion pathway prediction for non-typical granule proteins in neutrophils**

Non-typical granule proteins were subjected to bioinformatic tools to predict the classical secretory pathways or non-classical pathways. The prediction results were shown in pie chart **(A-D)**. **(A)** SignalP prediction. **(B)** SecretomeP prediction. **(C)** TMHMM prediction. **(D)** Vesiclepedia prediction. **(E)** Gene Ontology (Biological Process and Molecular Function) analysis of the 177 predicted secreted proteins. (adj. p-value < 0.01).

**Supplemental Figure 7. Comparison of the MMP/ADAMs mediated shedding of CD18, OLR1 and CD177 in multiple conditions.**

**(A)** Histogram plot showing TNF-α induced CD18 shedding events could be inhibited by GM6001. While LPS and PMA induced CD18 shedding events could not be significantly inhibited by GM6001.

**(B)** TNF-α, LPS or PMA induced the increase of CD177 can be inhibited by GM6001 treatment.

**(C)** Histogram plot showing TNF-α and LPS induced CD177 shedding events could be inhibited by GM6001. While the PMA induced CD177 shedding events could not be significantly inhibited by GM6001.

**(D)** The different temporal-expression patterns of IFN-related genes in the neutrophil under different stimulation: ISG15, ISG20, IFI35, IFI44.

Y axis: relative percentage (%), the relative percentage of granule proteins in the cellular compartment was calculated by comparing the protein MS signals (label-free quantification [LFQ] intensity) between the stimulated neutrophil and the control neutrophil at baseline (0 minutes).

**(E)** The temporal expression of classical granule proteins in the supernatant of TNF-α/LPS/PMA treated neutrophils and PBS treated neutrophils(control): MMP9, MMP8, OLFM4, LCN2. Y axis: label-free quantification [LFQ] intensity.

**Supplemental Figure 8. Integrated framework of neutrophil releases its mediators upon inflammation.**

Schematic illustrating two distinct mechanisms: (left panel) granule lumen protein release (direct exocytosis) and (right panel) membrane protein shedding (proteolytic cleavage by MMPs/ADAMs). Created with BioRender.com.

Neutrophil release the granule lumen proteins and granule membrane proteins through different mechanisms. The granule lumen proteins are directly released into extracellular space along with the fusion of granule membrane with the plasma membrane. In parallel, granule membrane proteins are translocated to the cell surface. Three potential pathways could be involved in membrane protein secretion：(i) post-fusion cleavage: granule membrane proteins might be cleaved by intramembrane sheddase or soluble sheddase after membrane fusion and then release into the environment, (ii) pre-fusion cleavage: the granule membrane proteins might be cleaved on the granule prior to fusion, (iii) vesicle-mediated pathway: secreted through vesicle-mediated pathway followed by extracellular proteolytic processing.

**Supplemental tables and table legends**

**Supplemental Table1.** **Neutrophil proteome dataset.** Abundance data for all proteins quantified in the proteomics analysis of neutrophils under inflammatory and infectious activation, shown as normalized LFQ intensity. The number of unique peptides assigned to the protein were also listed.

**Supplemental Table 2. Co-regulated protein clusters in LPS/TNF-α/PMA/Poly（I:C） treated neutrophils.** The proteins in representative clusters identified by MFUZZ analysis. Related to Figure 2.

**Supplemental Table 3. The neutrophil secretome dataset.** The secretome dataset includes protein accessions, quantification values (LFQ intensity), number of unique peptides assigned to the protein.

**Supplemental Table 4.** **The neutrophil secreted proteins.** The released 468 proteins in the activated neutrophil from different conditions.

**Supplemental Table 5. Candidate granule-associated proteins identified by co-kinetic analysis with established granule markers.** Proteins were selected based on similarity (Pearson r > 0.7) to canonical granule markers in both cellular expression patterns and secretion patterns.

**Supplemental Table 6. The sequence coverage of MS/MS identified transmembrane proteins.** The membrane-bound types of released transmembrane proteins, the number of MS/MS identified extracellular/intracellular residues, and their extracellular sequence coverage (%), intracellular sequence coverage (%).

**Supplemental Table 7. Proteins with stable abundance across all tested stimulation conditions.**Proteins listed herein exhibited minimal change in secretion levels under all tested stimuli.

**Supplemental Table 8. The differential expression detection results by MS-DAP analysis.** Only proteins meeting the following criteria were retained for downstream differential analysis: proteins must meet peptide-level detection thresholds (peptides must have a good confidence score and have a quantitative value in all the samples per group); proteins with at least 2 peptides; proteins observed in 70% of samples per group. Proteins that did not meet the above criteria were excluded from statistical testing and lack calculated P-values and fold changes (FC) in the table.

**Supplemental dataset.** We depicted the temporal expression patterns for each neutrophil-released protein in different conditions. There are the collections of Line plots which display the dynamic changes of protein levels in cellular and supernatant data across different conditions. TNF-α condition (blue background), LPS condition (yellow background), and PMA condition (green background). Left Y axis: the relative percentage of protein in cellular proteome. Right Y axis: the fold change of proteins in supernatant. X axis: time post-stimulation.
